# Co-developed neck exercise (EPIC-Neck) versus usual exercise care for people with chronic non-specific neck pain: a UK multicentre randomised feasibility trial

**DOI:** 10.1136/bmjopen-2026-120571

**Published:** 2026-10-20

**Authors:** Jonathan Price, Alison Rushton, Natalie Ives, Marilyn Bradbury, Priti Parmar, Colin J Greaves, EPIC-Neck feasibility trial collaborators, Apurba Subedi, Hayley Alderton, Katherine Yeung, Longsun Yau, Paul Matthews, Rajvinder Shokkar

**Affiliations:** 1 Musculoskeletal Physiotherapy Services, Birmingham Community Healthcare NHS Foundation Trust, Aston, UK; 2 Research and Innovation, Birmingham Community Healthcare NHS Foundation Trust, Aston, UK; 3 School of Sport, Exercise and Rehabilitation Sciences, University of Birmingham College of Life and Environmental Sciences, Birmingham, UK; 4 School of Physical Therapy, Western University, London, Ontario, Canada; 5 Institute of Applied Health Research, University of Birmingham College of Medical and Dental Sciences, Birmingham, UK

**Keywords:** Exercise, Self-Management, Feasibility Studies, Behavior, Spine

## Abstract

**Objectives:**

To assess the feasibility of conducting a definitive randomised controlled trial of the EPIC-Neck progamme versus usual exercise care for chronic non-specific neck pain, specifically in relation to recruitment, retention, delivery fidelity, acceptability and contamination.

**Design:**

Multicentre, two-arm, parallel-group, individually randomised controlled feasibility trial with an embedded process evaluation.

**Setting:**

Three National Health Service musculoskeletal services in England.

**Participants:**

Adults (≥18 years) with chronic non-specific neck pain lasting >3 months.

**Interventions:**

Participants were randomised (2:1) to EPIC-Neck (an individualised, evidence-based, theory informed, co-designed exercise programme) or usual exercise care. Participants and outcome assessors were not blinded to allocation.

**Feasibility objectives:**

Progression was assessed using predefined green–amber–red criteria across five domains: (1) recruitment rate, (2) retention rate (percentage returning the neck disability index at 6 months), (3) delivery fidelity (audio-recorded appointments scored on a 0–5 Dreyfus competence scale where ≥3 means acceptable), (4) acceptability to participants and physiotherapists (1–5 acceptability questionnaires where ≥4 means acceptable and interviews analysed deductively using framework analysis against the theoretical framework of acceptability) and (5) contamination (presence of EPIC-Neck components in usual care) scored using the same 0–5 Dreyfus competency scale as delivery fidelity.

**Results:**

Between May 2024 and February 2025, 287 individuals were screened and 36 were randomised (24 EPIC-Neck, 12 usual care), yielding a mean recruitment rate of 4.5 participants/month (green). Retention at 6 months was 67% (24/36) (red). Among participants who completed their allocated treatment, the 6-month retention rate was 91% (22/24). Delivery fidelity was moderate (mean 2.4/5; amber), with only 14% of participants receiving EPIC-Neck at an acceptable level. Acceptability was generally positive, although interviews identified addressable concerns around intervention coherence (amber). Contamination was low (mean fidelity score for controls was 1.1/5; green).

**Conclusions:**

EPIC-Neck shows potential for evaluation in a definitive trial, but progression is conditional on future refinement of the intervention and study procedures to address the key barriers to progression: retention, delivery fidelity and intervention coherence.

**Trial registration number:**

ISRCTN81746901

**STRENGTHS AND LIMITATIONS OF THIS STUDY**

- This multicentre randomised feasibility trial used an embedded mixed-methods process evaluation, allowing integration of quantitative and qualitative data from participants and physiotherapists.
- The high proportion of audio-recorded appointments enabled a robust assessment of delivery fidelity and contamination using a structured scoring framework.
- Inclusive recruitment and consent procedures, including multilingual/video materials and interpreter support, were used to reduce barriers to participation.
- Physiotherapists delivered the intervention to relatively few participants, so some fidelity limitations may reflect early learning effects.
- Fidelity assessments were unblinded, and qualitative interviews were conducted by the intervention developer, creating potential for bias despite mitigation strategies.

## Background

Neck pain continues to pose a growing global health challenge. The age-standardised number of years lived with disability for neck pain has increased in recent years, rising by 0.12% per year from 2011 to 2016 and 0.42% per year from 2016 to 2019, despite advancements in healthcare.^1^ Although all types of neck pain contribute to this burden, chronic non-specific neck pain is particularly impactful because symptoms are associated with greater pain, disability, psychological distress, reduced work capacity and healthcare use than acute neck pain.^2–6^ Effective treatments are needed to reduce immediate and long-term impact.

Clinical guidelines and multiple systematic reviews support the use of exercise to improve chronic non-specific neck pain and disability.^7–15^ However, effects are often modest, diminish over time^10 16^ and may be limited by poor adherence.^10 17–20^ To address these limitations, the EPIC-Neck programme (Exercise Prescription Improved through Co-design) was co-developed with patients and physiotherapists using Intervention Mapping.^21^ EPIC-Neck differs from usual exercise care by integrating physiotherapist-prescribed exercise, guided by a biopsychosocial prescription framework with structured behaviour change strategies to support exercise effects and adherence.^21^

Before evaluating adherence, clinical effectiveness and cost-effectiveness of EPIC-Neck compared with usual exercise care, key uncertainties that could compromise a definitive trial needed to be addressed.^22^ The Medical Research Council recommends that complex interventions, such as EPIC-Neck, undergo feasibility testing before definitive evaluation.^22^ For EPIC-Neck, key uncertainties included whether adults with chronic non-specific neck pain could be recruited and retained in an exercise-versus-exercise trial, whether EPIC-Neck was acceptable to patients and physiotherapists, whether key components could be delivered as intended and whether the intervention remained sufficiently distinct from usual exercise care to avoid contamination between trial arms.

### Aims and objectives

Aim: To determine the feasibility of a definitive trial evaluating the clinical and cost-effectiveness of EPIC-Neck compared with usual exercise care for adults with chronic non-specific neck pain.

Primary feasibility objectives, aligned directly with the pre-specified traffic-light progression criteria (table 1), were to assess the following:

- Mean participant recruitment.
- 6-month retention.
- Delivery fidelity of key EPIC-Neck components.
- Presence of key EPIC-Neck components in usual exercise care (contamination).
- Acceptability of EPIC-Neck to patients and physiotherapists.

**Table 1:** Traffic light progression criteria

	Green	Amber	Red	Feasibility outcomes
Mean recruitment rate	≥3 participants per month	1–2 participants per month	≤1 participant per month	Green: 4.5 participants per month (95% CI 3.0 to 6.1)
Retention rate	≥80%	70%–79%	<70%	Red: 67%, 95% CI (49 81)
Delivery fidelity	No areas of concern (most items ≥3)	Minor problems (≥50% of components between 1.5 and 3.0)	Major problems (most components <1.5)	Amber: overall mean score 2.4 (SD 1.3, 95% CI 1.7 to 3.1); 53% of components scored 1.5–3
Contamination	No EPIC-Neck components present in usual care	Minor overlap	Major overlap	Green: mean checklist score 1.1/5 (SD 1.0, 95% CI 0.6 to 1.6); 1/17 components observed
Acceptability	Mean acceptability score of ≥4 and no concerns in interviews	Mean acceptability score three and/or minor concerns	Mean acceptability score ≤2 and/or major concerns	Amber: mean 3.9 (SD 1.0, 95% CI 3.7 to 4.1); interview concerns were addressable

Green: no areas of concern. Amber: minor modifications required before continuing to a definitive trial. Red: major modifications required before continuing to a definitive trial.

EPIC-Neck, Exercise Prescription Improved through Co-design.

Secondary feasibility objectives were as follows:

- Describe treatment engagement, including initiation, attendance, appointment time, treatment duration and completion.
- Assess safety.
- Describe characteristics of individuals who did and did not enrol.
- Assess completeness of planned definitive-trial outcome measures.

## Methods

### Reporting

This trial is reported in accordance with the Consolidated Standards of Reporting Trials (CONSORT) checklist for pilot/feasibility trials (online supplemental file 1) and the published protocol (online supplemental file 2).^23 24^

10.1136/bmjopen-2026-120571.supp1 Supplementary data

10.1136/bmjopen-2026-120571.supp2 Supplementary data

### Trial design

This was a multicentre, two-arm, parallel-group, individually randomised controlled feasibility trial. Participants were randomised in a 2:1 ratio to either the intervention group (EPIC-Neck programme) or usual exercise care. The feasibility trial evaluated outcomes important for deciding whether and how to progress to a definitive trial, including recruitment, retention, treatment engagement, acceptability, contamination, outcome-measure completion and safety. Embedded within the feasibility trial was a mixed-methods process evaluation, which included a multidimensional fidelity evaluation to understand EPIC-Neck training, delivery, receipt and enactment and to inform intervention and training refinements. This manuscript reports the feasibility outcomes relevant to progression to a definitive trial; the detailed multidimensional fidelity evaluation is reported separately.^25^ The trial was registered (ISRCTN81746901) and received ethical approval from South Central - Oxford B Research Ethics Committee (24/SC/0006).

### Participant eligibility, identification and consent procedures

Participants were recruited from National Health Service (NHS) community-based and secondary care musculoskeletal services. Recruitment was originally planned across two NHS sites as described in the protocol; however, one site withdrew during study set-up, and two additional NHS musculoskeletal services were subsequently recruited. Adults (≥18 years) with chronic (≥3 months) non-specific neck pain were eligible. Non-specific neck pain was defined as pain or discomfort in the neck and/or shoulder girdle, with or without referred arm pain, for which no specific cause could be identified.^26 27^ Individuals with recent physiotherapy within 6 months, previous cervical surgery, traumatic or specific neck pathology, neurological involvement or inability to follow trial procedures were excluded. Full eligibility criteria are reported in the protocol.^24^

NHS clinical staff identified potential participants from waiting lists or trial posters and provided a Participant Information Leaflet. Potential participants completed telephone eligibility screening and, if eligible, attended a face-to-face baseline assessment with a research physiotherapist. Written informed consent was obtained for the feasibility trial and qualitative interviews, eligibility was confirmed and baseline assessments were completed prior to randomisation. Trial materials were available in paper, electronic and video formats, with English or Urdu audio narration and professional interpreters supported screening and consent where required.^28 29^

### Randomisation

Randomisation was conducted via a secure web-based system (sealed envelope). The sequence was computer-generated using random permuted blocks (sizes 3 and 6) with a 2:1 allocation to EPIC-Neck. A 2:1 allocation ratio in favour of the intervention group was used to ensure sufficient intervention exposure and participant feedback to inform feasibility, acceptability and intervention refinement.^30^ Allocation was concealed through the automated system, which released the assignment only after baseline data entry by the research physiotherapist.

### Blinding

Blinding of treating physiotherapists was not possible owing to the nature of the intervention, but cross-contamination was minimised by physiotherapists delivering either EPIC-Neck or usual exercise care but not both. Participants and outcome assessors were not blinded in this feasibility trial. Blinding procedures will be considered where appropriate in the definitive trial.^31^

### Physiotherapist consent procedures

Physiotherapists at participating sites provided written informed consent to deliver either EPIC-Neck or usual exercise care and to participate in the process evaluation (audio-recorded appointments/interviews).^24^ Characteristics (age, sex, qualifications, years of experience and postgraduate training) were collected by self-report before training and were not independently verified.

### Interventions

#### EPIC-Neck (intervention arm)

Full details of the EPIC-Neck programme are published elsewhere.^21^ In brief, EPIC-Neck is a biopsychosocial, theory-informed intervention designed to support self-management of chronic neck pain through a home-based exercise programme. Physiotherapists prescribed exercises using a biopsychosocial framework to target participants’ physical, psychological and social needs (online supplemental file 3, figure S3-2). A behaviour change facilitation guide supported exercise engagement using techniques such as goal setting, self-monitoring, action planning, social support and self-efficacy enhancement, delivered through patient-centred communication (online supplemental file 3, figure S3-1).^32 33^ EPIC-Neck was delivered face-to-face by trained physiotherapists and supported by paper and digital patient-facing materials (workbook, exercise diary, exercise library and friends-and-family leaflet). Non-exercise treatments (eg, postural advice and manual therapy) were provided at the physiotherapist’s discretion. Discharge was agreed collaboratively based on goals and self-management ability.

10.1136/bmjopen-2026-120571.supp3 Supplementary data

##### Physiotherapist training

Physiotherapists delivering EPIC-Neck completed a 1-day face-to-face training combining lectures, role play and behavioural skills practice.^34 35^ Ongoing support included a training manual, crib sheets, optional booster sessions and peer/expert support.^34 36^ Audio-recordings of intervention delivery were used for fidelity evaluation, but real-time monitoring, feedback or delivery coaching were not provided during the trial.

#### Usual exercise care (control arm)

The usual exercise care group received physiotherapy exercises as per the physiotherapists’ standard practice. Physiotherapists were not given any additional instruction or training. Non-exercise treatments (eg, postural advice and manual therapy) were provided at the physiotherapist’s discretion.

### Outcomes

#### Feasibility progression criteria

Feasibility of progressing to a definitive trial was assessed across five pre-specified domains: recruitment, 6-month retention, delivery fidelity, contamination and acceptability. A traffic-light system with a priori thresholds was used to guide progression decision (table 1).^37^

#### Recruitment

The mean number of participants randomised per month was calculated across the active recruitment period.^38^ Thresholds (table 1) were based on the estimated time required to recruit the anticipated definitive trial sample size of 248 participants across five musculoskeletal services.^38^

#### Retention

Return of the Neck Disability Index (NDI) at 6 months was used as the retention criterion, selected as an indicator of likely follow-up at the planned 12-month primary endpoint in a definitive trial.^39 40^ Thresholds (table 1) were informed by guidance that ≥80% retention represents low risk of attrition bias and comparable musculoskeletal trials reporting approximately 70% retention.^41–46^

#### Delivery fidelity

All EPIC-Neck appointments were audio-recorded and coded against a pre-defined 17-item checklist of key EPIC-Neck components (online supplemental file 3, table S3-1).^47 48^ Each component was rated using a 0–5 Dreyfus delivery competence scale, where 0 indicated absent delivery, ≥3 acceptable delivery and 5 optimal delivery.^49^ Progression thresholds (table 1) were based on the distribution of checklist scores across components, with ≥3 used as the threshold for acceptable delivery, consistent with previous fidelity research using this approach.^48 50 51^

#### Contamination

The presence of EPIC-Neck components within usual exercise care was assessed using audio-recorded usual care appointments, coded against the same 17-item checklist and 0–5 Dreyfus competence scale used for EPIC-Neck delivery fidelity.^52 53^ Thresholds (table 1) reflected the extent to which EPIC-Neck components appeared in usual care and whether this threatened distinction between trial arms. Final progression judgements were made through discussion within the study team (JP, MB and CG), informed by the checklist scores. Interviews with usual-care physiotherapists were undertaken to further characterise usual exercise care and will be reported elsewhere.

#### Acceptability

EPIC-Neck acceptability to participants and physiotherapists was evaluated using a convergent mixed-methods approach informed by the Theoretical Framework of Acceptability (TFA).^54 55^ EPIC-Neck participants completed 3-month and 6 month questionnaires covering TFA domains of affective attitude, intervention coherence, perceived effectiveness, opportunity costs and self-efficacy. Items were rated on 1–5 Likert scales and interpreted at face value according to the questionnaire response anchors, where scores of 4–5 indicated positive acceptability, 3 indicated neutrality and 1–2 indicated negative acceptability.^54^ Qualitative acceptability was explored through semi-structured interviews with participants (post-discharge) and physiotherapists (end of trial), using patient and public involvement and engagement (PPIE)-informed topic guides mapped to all seven TFA domains. Interviews were conducted by telephone, video call or face-to-face and audio-recorded. Progression criteria (table 1) combined these quantitative thresholds with qualitative findings. Final progression judgements were made through discussion between JP and CG, informed by questionnaire scores and qualitative findings. Any disagreements were resolved through discussion with a third study team member.

#### Allocated treatment engagement

Allocated treatment engagement was described using initiation, attendance, did-not-attend appointments, total appointment time, treatment duration and treatment completion. Treatment initiation was defined as attendance at ≥1 appointment, and treatment completion as collaboratively agreed discharge. The total appointment time was calculated from available audio-recordings, and treatment duration as weeks from initial assessment to final attended appointment.

#### Inclusivity

Demographic characteristics of randomised and non-randomised individuals identified from screening logs were compared. Variables included age, sex, ethnicity, first language, interpreter need and deprivation, measured using Index of Multiple Deprivation (IMD) decile. The IMD is a composite measure of area-level deprivation in England, incorporating domains including income, employment, education, health, crime, housing and living environment, with deciles ranging from 1 (most deprived 10%) to 10 (least deprived 10%).

#### Feasibility of trial outcome measures

Planned definitive-trial outcome measures were collected at baseline and at 3 and 6 months post-randomisation to assess data collection feasibility and completeness. Baseline data were collected face to face, and follow-up questionnaires were completed by post, with telephone completion and interpreter support available where required. A £20 voucher was offered for returning all three questionnaires. Outcomes were selected based on existing literature, PPIE input and the EPIC-Neck logic model.^21^ The planned definitive trial primary outcome was the NDI, which is widely used with good psychometric properties and a minimum clinically important difference of 5.5 points.^56 57^ Secondary outcome measures include pain (Visual Analogue Scale (VAS)),^58^ health-related quality of life (EuroQol 5-Dimension 5-Level),^59^ mental well-being (Hospital Anxiety and Depression Scale),^60^ sleep quality (The Sleep Condition Indicator),^61^ specific activity limitations (Patient Specific Functional Scale (PSFS))^62^ and social/relationship impact (Relationships and Self-Worth domains of Assessment of Quality of Life 8D).^63 64^ Candidate mediators were pain beliefs (Single item Survey of Pain Attitudes Questionnaire),^65^ pain-related fear (Tampa Scale of Kinesiophobia-13),^66^ pain self-efficacy (Pain self-efficacy questionnaire)^67^ and night pain (VAS).^58^

### Data analysis

Quantitative data were analysed using IBM SPSS v25 and were primarily descriptive, using means, SD, ranges, proportions and 95% CIs, as appropriate. For delivery fidelity and contamination, two researchers (JP and MB) independently coded an initial sample of audio-recordings and iteratively calibrated scoring using further recordings until ≥75% of ratings were within 1 point.^50 68^ Remaining recordings were coded by JP. Fidelity and contamination scores were summarised descriptively at the component, participant and study levels. Treatment initiation, completion and discharge following non-attendance were summarised using the number randomised to each group as the denominator. Among participants attending ≥1 appointment, attendance, non-attendance, total appointment time and treatment duration were summarised descriptively. Demographic characteristics of randomised and non-randomised individuals were compared using Pearson’s χ2 tests (p < 0.05). As this was a feasibility trial, no inferential between-group comparisons were planned or undertaken for the planned definitive-trial outcome measures.^69^

Qualitative acceptability data were analysed using deductive framework analysis.^70 71^ Interviews were audio-recorded, transcribed verbatim and coded against TFA domains in NVivo 12 Plus (QSR International). Data were summarised in a domain-by-case matrix to compare patterns within and across cases for participants and physiotherapists separately. Minor (amber), major (red) or no (green) acceptability concerns were identified within each domain. All interviews and qualitative analysis were completed by JP, an experienced physiotherapist and lead developer of the EPIC-Neck programme. Reflexivity was addressed by acknowledging prior professional relationships with some physiotherapists and none with participants. To support analytic rigour and consistency, a subset of domain coding was reviewed within the research team. Quantitative and qualitative acceptability findings were triangulated in a domain-level table, with domain-level acceptability ratings combined to provide an overall judgement.

### Sample size

Target recruitment was 45 participants based on a precision calculation to estimate retention with sufficient accuracy to inform a future definitive trial. Assuming 80% retention in this feasibility trial, approximately 43 participants were required to estimate retention with a 95% CI of ±12%; this was rounded to 45 participants.^45 72^ No interim analyses or stopping rules were planned.

Semi-structured interviews were conducted with a purposive sample of 12 EPIC-Neck participants, sampling for variation in ethnicity, socio-economic deprivation and baseline neck disability to reflect potentially under-served groups and diverse pain experiences.^73–75^ All physiotherapists delivering the EPIC-Neck programme were invited to be interviewed.

### Patient and public involvement and engagement

The trial protocol, intervention development, outcome selection, inclusivity design, trial materials and dissemination were informed by a PPIE group (n=17). Two PPIE members were part of the Study Management Group. PPIE contributions are reported in more detail in online supplemental file 4 (Guidance for Reporting Involvement of Patients and the Public 2—Short Form).^76^

10.1136/bmjopen-2026-120571.supp4 Supplementary data

## Results

### Screening and recruitment

Recruitment took place between 29 May 2024 and 10 February 2025 (figure 1). Of the 287 individuals invited, 36 were randomised (24 EPIC-Neck; 12 usual exercise care), corresponding to an uptake rate of 12.5% (95% CI 8.7 to 16.4). Recruitment stopped before the planned target of 45 participants was reached because the available study time and funding period ended. At this time point, sufficient data had been collected to evaluate the pre-specified feasibility objectives.

**Figure 1:**
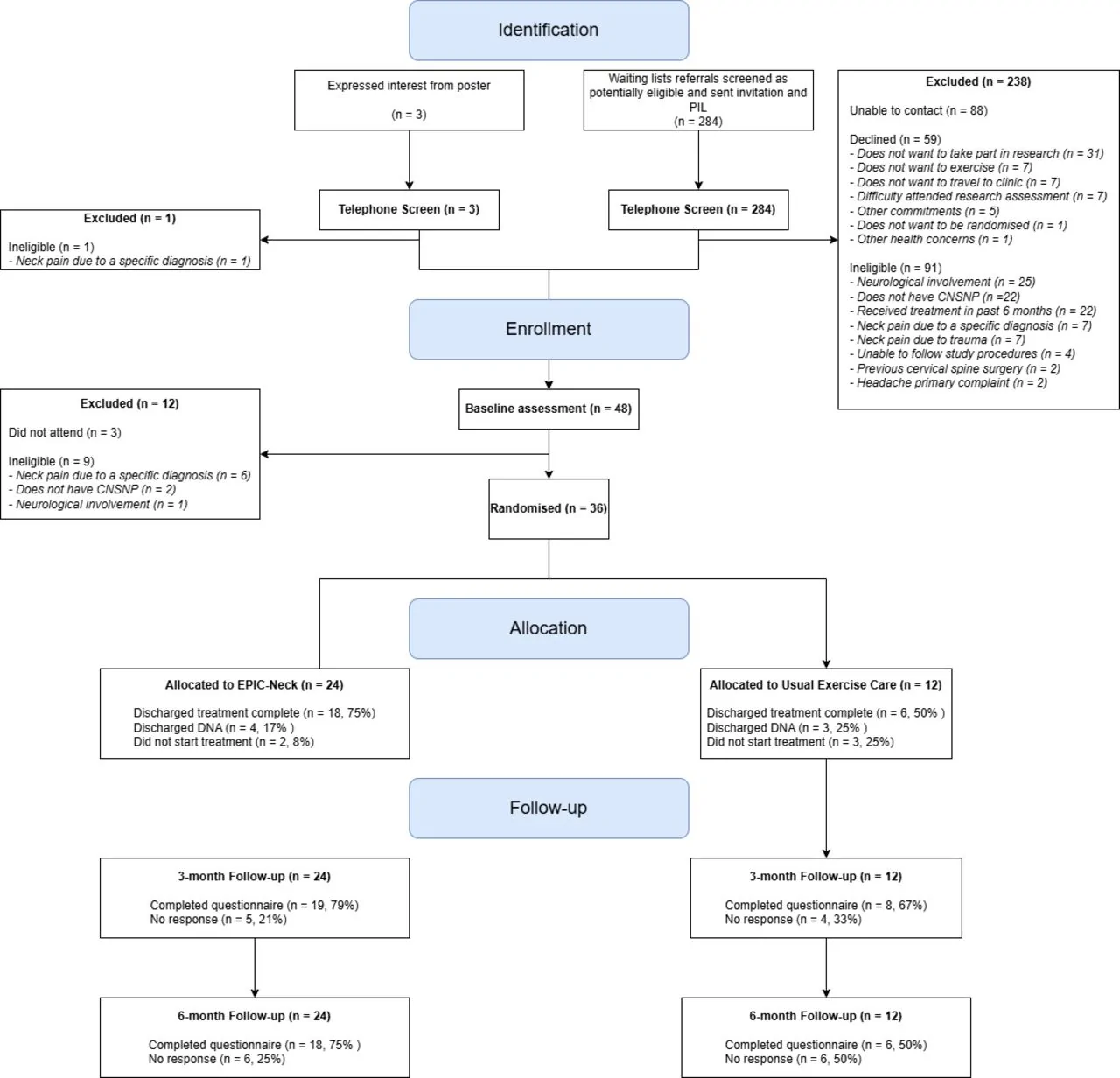
CONSORT flow diagram. CNSNP, chronic non-specific neck pain; DNA, did not attend; PIL, participant information leaflet.

### Participant characteristics

Participants had a mean age of 45.9 (range 19–82) years, with a similar number of males and females (table 2). Overall, 41.7% were of Asian or Asian British ethnic origin, and 72.2% lived in the most deprived IMD deciles.

**Table 2:** Baseline characteristics of the participating patients

Demographic characteristics	EPIC-Neck n=24		Usual exercise care n=12		Full sample n=36	
	n	%	n	%	n	%
Sex						
Female	11	45.8%	5	41.7%	16	44.4%
Male	13	54.2%	7	58.3%	20	55.6%
Age						
18–30	3	12.5%	3	25.0%	6	16.7%
31–40	7	29.2%	1	8.3%	8	22.2%
41–50	3	12.5%	3	25.0%	6	16.7%
51–60	7	29.2%	3	25.0%	10	27.8%
61+	4	16.7%	2	16.7%	6	16.7%
Ethnicity						
Asian or Asian British	9	37.5%	6	50.0%	15	41.7%
Indian	2	8.3%	1	8.3%	3	8.3%
Pakistani	7	29.2%	5	41.7%	12	33.3%
Black or black British	3	12.5%	2	16.7%	5	13.9%
African	1	4.2%	1	8.3%	2	5.6%
Caribbean	1	4.2%	0	0.0%	1	2.8%
Other	1	4.2%	1	8.3%	2	5.6%
Mixed	1	4.2%	1	8.3%	2	5.6%
White and black Caribbean	1	4.2%	1	8.3%	2	5.6%
White	10	41.7%	3	25.0%	13	36.1%
British	9	37.5%	3	25.0%	12	33.3%
Irish	1	4.2%	0	0.0%	1	2.8%
Other	1	4.2%	0	0.0%	1	2.8%
Arab	1	4.2%	0	0.0%	1	2.8%
IMD decile						
Most deprived (deciles 1–3)	19	79.2%	7	58.3%	26	72.2%
Middle (deciles 4–7)	4	16.7%	5	41.7%	9	25.0%
Least deprived (deciles 8–10)	1	4.2%	0	0.0%	1	2.8%
Marital status						
Living with partner	6	25.0%	1	8.3%	7	19.4%
Married/civil union	13	54.2%	5	41.7%	18	50.0%
Separated/divorced	1	4.2%	1	8.3%	2	5.6%
Unmarried (never married)	4	16.7%	5	41.7%	9	25.0%
Cohabiting status						
Cohabiting	24	100.0%	10	83.3%	34	94.4%
Living alone	0	0.0%	2	16.7%	2	5.6%
Neck pain location						
Left	11	45.8%	9	75.0%	20	55.6%
Right	11	45.8%	6	50.0%	17	47.2%
Middle	7	29.2%	7	58.3%	14	38.9%
Base of skull	2	8.3%	1	8.3%	3	8.3%

EPIC-Neck, Exercise Prescription Improved through Co-design-Neck; IMD, index of multiple deprivation.

### Feasibility objectives

#### Mean recruitment rate

The mean recruitment rate was 4.5 participants per month (95% CI 3.0 to 6.1; 36 participants over 8 months), exceeding the ‘green’ progression criterion (table 1).

#### Retention rate

At 6 months, 67% of participants returned the NDI questionnaire (24/36; 95% CI 49 to 81), meeting the ‘red’ criterion (table 1). Retention was 75% in EPIC-Neck (18/24) and 50% in usual exercise care (6/12) (figure 1). All 3-month non-responders were also non-responders at 6 months (9/9). Among 6-month non-responders, 41% (5/12) had not initiated treatment, and 41% (5/12) were discharged for non-attendance. Retention among treatment completers was high (22/24; 91%).

#### Delivery fidelity

Fidelity was scored for 76/80 EPIC-Neck appointments; four were excluded due to missing recordings (n=2) or a participant developing neurological symptoms (n=2). Overall mean fidelity was 2.4/5 (SD 1.3; 95% CI 1.7 to 3.1). Component scores ranged from 0.9 to 3.5, with 6/17 components (35%) delivered acceptably (≥3) and 9/17 (53%) scoring 1.5–<3.0 (table 3). Only 3/22 participants (14%) had a mean score of ≥3 (participant means 1.3–3.2). Based on the study-level mean and component-level distribution, delivery fidelity met the amber progression criterion (table 1).

**Table 3:** Delivery fidelity scores by component

Component	Mean score (SD)	
	EPIC-Neck	UEC
01 Agree outcome goals	2.0 (1.3)	0.9 (0.9)
02 Actively involve the patient	3.1 (0.7)	1.2 (0.4)
03 Improve motivation to perform exercise (PO1) – enhance perceived importance and safety	2.1 (1.0)	1.4 (0.6)
04 Improve motivation to perform exercise (PO1) – improve self-efficacy	3.5 (0.8)	3.0 (1.1)
05 Improve motivation to perform exercise (PO1) – engage social support	1.8 (1.1)	0.4 (0.5)
06 Set a plan to perform exercise (PO1)	2.3 (1.0)	1.0 (0.0)
07 Encourage self-monitoring	3.3 (0.9)	0.0 (0.0)
08 Review progress	2.7 (0.5)	1.6 (0.3)
09 Support problem solving	1.3 (0.7)	0.8 (0.5)
10 Introduce adapting and progressing (or regression) exercise (PO2)	1.6 (1.1)	0.2 (0.4)
11 Introduce flare-up exercise (PO3)	1.5 (1.0)	0.1 (0.3)
12 Introduce general exercise+/-secondary prevention exercise (PO4)	0.9 (0.9)	0.1 (0.3)
13 Evaluate self-management and discharge suitability	2.0 (1.2)	0.8 (0.9)
14 Identify short-term outcomes and exercise objectives	3.1 (0.8)	1.0 (0.0)
15 Home exercise programme – normalising movement	3.5 (0.7)	2.0 (1.0)
16 Home exercise programme – improving tolerance	2.7 (1.6)	2.2 (0.7)
17 Exercise review	3.0 (1.2)	2.2 (1.1)
	2.4 (1.3)	1.1 (1.0)

EPIC-Neck, Exercise Prescription Improved through Co-design-Neck; PO, performance objective; UEC, usual exercise care.

#### Contamination

Intervention content was scored for 29/32 usual exercise care appointments; three were excluded because of missing recordings. The mean checklist score was 1.1/5 (SD 1.0; range 0.6–1.5, 95% CI 0.6, 1.6), with 1/17 components (‘improve self-efficacy’) delivered in a manner consistent with EPIC-Neck (table 3). This met the ‘green’ contamination criterion (table 1).

#### Acceptability

Acceptability findings were similar at 3 and 6 months; therefore, only the 6-month findings are considered in the integrated acceptability assessment. Both 3-month and 6-month data are available in online supplemental file 5. Eighteen EPIC-Neck participants returned acceptability questionnaires (table 4). Mean scores met the pre-defined ≥4/5 threshold for affective attitude, intervention coherence, perceived effectiveness and self-efficacy. Opportunity costs, reflecting the extent to which doing the exercises interfered with other priorities, were neutral (mean 3.3, SD 1.0; 95% CI 2.8 to 3.9), and overall acceptability was 3.9 (SD 1.0; 95% CI 3.7 to 4.1).

10.1136/bmjopen-2026-120571.supp5 Supplementary data

**Table 4:** Acceptability summary

Domain	Patient questionnaire (n=18) mean (SD) (95% CI)	Patient interviews (n=12)	Physiotherapist interviews (n=5)	Integrated summary
Affective attitude	Green: 4.1 (0.8)(3.7 to 4.5)	Amber: satisfaction with individualised, targeted approach and associated benefits, though some expressed negative feelings about physical side effects and general exercise; overall, positive outcomes outweighed lack of enjoyment.	Green: enjoyed structured approach and behaviour change techniques	Amber: address patient concerns about physical side effects and attitudes to general exercise
Burden	NA	Amber: exercises that were complex or required specific space/positioning increased effort and were often omitted; time burden varied, with integration into daily routines reducing effort.	Amber: cognitive and time burden due to intervention depth, volume and changing practice which improved with exposure	Amber: address patient burden related to exercise duration and complex exercises and physiotherapist burden related to intervention depth and change in clinical practice.
Ethicality	NA	Amber: alignment with values on individualised, non-pharmacological management but misalignment for some with values around shared decision-making, dislike of routines and preference for interpersonal support over written or video resources.	Amber: alignment with individualised biopsychosocial patient-centred care beliefs but misaligned for some regarding goal setting and shared decision-making beliefs.	Amber: address patient misalignment with shared decision-making and resource preferences and physiotherapist misalignment with goal setting and shared decision-making.
Intervention coherence	Green: 4.1 (1.1)(3.6 to 4.7)	Red: patients understood basic exercise performance but had limited grasp of purpose, benefits and progression strategies; understanding of flare-up management and general exercise plans was inconsistent and rarely aligned with EPIC-Neck standards.	Amber: coherent with familiar components but low with new or more complex components—improved with practice but remained limited	Red: improve patients’ understanding of programme rationale, progression strategies and flare-up management and strengthen physiotherapists’ understanding of how to deliver newer or more complex components.
Opportunity costs	Amber: 3.3 (1.0)(2.8 to 3.9)	Amber: most reported no trade-offs, but some described minor sacrifices outweighed by benefits, whereas others de-prioritised exercise in favour of other activities.	Green: minimal trade-offs	Amber: address patient de-prioritisation of exercise
Perceived effectiveness	Green: 4.1 (1.2)(3.5 to 4.7)	Green: consistent benefits in pain, disability and function with sleep, mental well-being and relationships improvements noted where limited at baseline	Amber: overall perceived as effective with reservations regarding goal setting, social support and the patient resources	Amber: address physiotherapist concerns about goal setting, social support and patient resources.
Self-efficacy	Green: 4.1 (0.9)(3.7 to 4.5)	Amber: confident with basic exercises and self-managing flare-ups, but confidence declined with complexity, progression strategies and general exercise citing fear of harm	Amber: confident with familiar components, but low with new or more complex components—improved with practice but remained limited	Amber: address patient confidence with complex exercises, safety, progression strategies and general exercise and physiotherapist confidence with new or complex components.
Overall acceptability	Amber: 3.9 (1.0)(3.7 to 4.1)	–	–	Amber: broadly acceptable with addressable concerns particularly related to intervention coherence; further refinement and re-testing are required before a definitive trial.

Green: no areas of concern, mean acceptability score of ≥4 and no areas of concern identified in qualitative interviews. Amber: minor problems that require attention before continuing to a definitive trial, mean acceptability score 3 and/or minor problems identified in qualitative interviews. Red: major problems that require urgent attention before continuing to a definitive trial, mean acceptability score ≤2 and/or major problems identified in qualitative interviews.

EPIC_Neck, Exercise Prescription Improved through Co-design-Neck.

Interviews were completed with 12 participants and 5 physiotherapists (table 5). Qualitative analysis (table 4; full details are provided in online supplemental file 5) indicated no participant concerns about intervention effectiveness. Addressable concerns were identified in other TFA domains, particularly intervention coherence, defined here as participants’ understanding of the intervention and how it was intended to work. Participants showed limited understanding of the exercise rationale and performance objectives (exercise progression/regression, flare-up management and general exercise). Physiotherapists reported positive affective attitude and minimal opportunity costs but had addressable concerns around coherence and self-efficacy when delivering newer or more complex components.

**Table 5:** Characteristics of interviewed patients and physiotherapists

	*N*	%
Patient participants (n=12)		
Sex		
Female	4	33.3%
Male	8	66.7%
Age		
18–30	1	8.3%
31–40	3	25.0%
41–50	2	16.7%
51–60	3	25.0%
61+	3	25.0%
Ethnicity		
Asian or Asian British	**3**	**25.0%**
Indian	0	0.0%
Pakistani	3	25.0%
Black or black British	**2**	**16.7%**
African	0	0.0%
Caribbean	1	8.3%
Other	1	8.3%
Mixed	**1**	**8.3%**
White and black Caribbean	1	8.3%
White	**5**	**41.7%**
British	5	41.7%
Irish	0	0.0%
Other	**1**	**8.3%**
Arab	1	8.3%
IMD decile		
Most deprived (deciles 1–3)	10	83.3%
Middle (deciles 4–7)	2	16.7%
Least deprived (deciles 8–10)	0	0.0%
Neck pain related disability (NDI)		
No disability	2	16.7%
Mild disability	5	41.7%
Moderate disability	5	41.7%
Severe disability	0	0.0%
Complete disability	0	0.0%
Physiotherapists (n=5)		
Sex		
Female	3	60%
Male	2	40%
Age		
20–29	1	20%
30–39	1	20%
40–49	1	20%
50–59	2	40%
Highest qualification		
Bachelor’s degree	5	100%
Years experience treating chronic non-specific neck pain		
0–2	2	40%
3–5	0	0%
6–10	2	40%
11–20	1	20%
Postgraduate training areas		
Neck pain assessment and treatment	3	60%
Exercise prescription	3	60%
Behaviour change	2	40%
Psychologically informed practice	1	20%
Patient-centred communication techniques	3	60%

IMD, index of multiple deprivation; NDI, Neck Disability Index.

Integrated interpretation (table 4) showed that although questionnaires suggested good patient acceptability across most domains, interviews identified coherence gaps for both participants and physiotherapists. Although intervention coherence was identified as a substantial concern, the overall acceptability judgement reflected findings across all TFA domains rather than any single domain. Overall, EPIC-Neck was acceptable with addressable concerns, meeting the amber progression criterion.

#### Allocated treatment engagement

Treatment was not initiated by 2/24 EPIC-Neck participants (8%) and 3/12 usual exercise care participants (25%). Treatment was completed by 18/24 EPIC-Neck participants (75%) and 6/12 usual exercise care participants (50%). A further 4/24 EPIC-Neck participants (17%) and 3/12 usual exercise care participants (25%) initiated treatment but were discharged following non-attendance.

Among participants attending ≥1 appointment, both groups attended a mean of 3.6 appointments: EPIC-Neck, SD 1.4, range 1–7; usual exercise care, SD 2.6, range 1–9. Mean did-not-attend appointments were 0.7 in EPIC-Neck (SD 0.8, range 0–3) and 1.1 in usual exercise care (SD 1.3, range 0–3) groups. The mean total appointment time was 1 hour 55 min (SD 56 min, range 27 min to 4 hours 7 min) in the EPIC-Neck group and 1 hour 30 min (SD 52 min, range 26 min to 2 hours 42 min) in the usual exercise care group. The mean treatment duration was 15.9 (SD 8.7, range 0–31) weeks in the EPIC-Neck group and 11.3 (SD 10.0, range 0–29) in the usual exercise care group.

#### Safety

No exercise-related serious adverse events were reported. One participant experienced a temporary worsening of symptoms (related, non-serious), and one experienced transient syncope at work (unrelated, serious).

#### Inclusivity

No statistically significant differences were observed for any demographic characteristic between randomised and non-randomised individuals (table 6).

**Table 6:** Demographics randomised versus non-randomised

Demographic characteristic	Non-randomised (n=251)		Randomised (n=36)		P value (Pearson’s χ2 test)
	*n*	%	*n*	%	
Sex					p=0.110
Female	147	58.6%	16	44.4%	
Male	104	41.4%	20	55.6%	
Age					p=0.445
18–30	33	13.1%	6	16.7%	
31–40	46	18.3%	8	22.2%	
41–50	54	21.5%	6	16.7%	
51–60	47	18.7%	10	27.8%	
61+	71	28.3%	6	16.7%	
Ethnicity					p=0.549
Asian or Asian British	95	37.8%	11	30.6%	
Black or black British	16	6.4%	5	13.9%	
Mixed	5	2.0%	1	2.8%	
White	73	29.1%	11	30.6%	
Other	7	2.8%	0	0.0%	
Not known	55	21.9%	8	22.2%	
First language					p=0.264
English	84	33.5%	9	25.0%	
Other	50	19.9%	5	13.9%	
Not known	117	46.6%	22	61.1%	
Interpreter required					p=0.434
No	227	90.4%	34	94.4%	
Yes	24	9.6%	2	5.6%	
IMD decile					p=0.761
Most deprived (deciles 1–3)	193	76.9%	27	75.0%	
Middle (deciles 4–7)	46	18.3%	8	22.2%	
Least deprived (deciles 8–10)	12	4.8%	1	2.8%	

For consistency, demographic data were taken from the screening logs, as these could differ from self-reported baseline questionnaire data for randomised participants.

IMD, index of multiple deprivation.

#### Feasibility of trial outcome measures

Summary statistics (means, SDs, 95% CIs) for outcomes and candidate mediators are shown in table 7. The PSFS was not completed at baseline by two participants who reported no functional limitations. All other measures had 100% baseline return, and 75% and 67% return at 3 and 6 months, respectively. Return rates and item-level completeness are presented in online supplemental file 3-table S3-2.

**Table 7:** Descriptive summary of planned definitive trial outcome and candidate mediator measures

Outcome measure	EPIC-Neck			Usual exercise care		
	n	Mean (SD)	95% CI	n	Mean (SD)	95% CI
Planned definitive trial primary outcome measure						
Neck pain-related disability, NDI (0=best, 50=worst)						
Baseline	24	14.13 (7.12)	11.12 to 17.13	12	16.58 (8.84)	10.97 to 22.20
3 months	19	14.21 (10.97)	8.92 to 19.50	8	16.50 (10.30)	7.89 to 25.11
6 months	18	12.39 (12.79)	6.03 to 18.75	6	16.67 (12.47)	3.58 to 29.75
Planned definitive trial secondary outcome measures						
Neck pain, VAS (0=best, 10=worst)						
Baseline	24	5.63 (1.86)	4.84 to 6.41	12	5.67 (2.31)	4.20 to 7.13
3 months	19	4.42 (2.87)	3.04 to 5.81	8	4.25 (2.55)	2.12 to 6.38
6 months	18	3.50 (2.64)	2.19 to 4.81	6	4.00 (2.28)	1.61 to 6.39
Functional and activity impact, PSFS (0=worst, 10=best)						
Baseline	22	5.09 (1.77)	4.31 to 5.88	12	4.50 (2.43)	2.96 to 6.04
3 months	17	5.47 (2.48)	4.20 to 6.74	8	4.75 (2.55)	2.62 to 6.88
6 months	16	6.69 (2.65)	5.27 to 8.10	6	5.17 (2.93)	2.10 to 8.24
Sleep, SCI (0=worst, 32=best)						
Baseline	24	16.50 (6.49)	13.76 to 19.24	12	13.83 (6.39)	9.77 to 17.90
3 months	19	16.84 (9.55)	12.24 to 21.44	8	13.88 (8.11)	7.09 to 20.66
6 months	18	18.72 (9.89)	13.80 to 23.64	6	15.00 (6.60)	8.07 to 21.93
Mental well-being, HADS (0=best, 42=worst)						
Baseline	24	12.46 (7.87)	9.14 to 15.78	12	16.00 (10.59)	9.27 to 22.73
3 months	19	14.32 (10.26)	9.37 to 19.26	8	16.50 (14.28)	4.56 to 28.44
6 months	18	12.89 (10.36)	7.74 to 18.04	6	12.67 (12.34)	−0.28 to 25.62
Relationships and social function, AQoL (0=worst, 100=best)						
Baseline	24	74.63 (17.21)	67.36 to 81.89	12	70.75 (23.69)	55.70 to 85.80
3 months	19	71.42 (20.87)	61.36 to 81.48	8	67.13 (27.30)	44.30 to 89.95
6 months	18	75.56 (20.56)	65.33 to 85.78	6	68.83 (23.95)	43.70 to 93.97
Health-related quality of life, EQ-5D-5L (−0.594=worst, 1=best)						
Baseline	24	0.65 (0.24)	0.54 to 0.75	12	0.60 (0.26)	0.43 to 0.77
3 months	19	0.65 (0.32)	0.49 to 0.80	8	0.62 (0.22)	0.44 to 0.81
6 months	18	0.65 (0.36)	0.47 to 0.83	6	0.63 (0.20)	0.43 to 0.84
Potential measures of mediation						
Night pain, NPRS (0=best, 10=worst)						
Baseline	24	5.58 (2.75)	4.42 to 6.74	12	5.42 (2.35)	3.92 to 6.91
3 months	19	3.95 (2.84)	2.58 to 5.32	8	4.63 (2.56)	2.48 to 6.77
6 months	18	3.28 (2.87)	1.85, 4.70	6	4.83 (3.13)	1.55, 8.11
Pain self-efficacy, PSEQ (0=worst, 60=best)						
Baseline	24	40.29 (12.67)	34.94, 45.64	12	36.33 (14.30)	27.25, 45.42
3 months	19	44.53 (15.74)	36.94, 52.11	8	39.25 (13.95)	27.59, 50.91
6 months	18	47.11 (16.05)	39.13, 55.09	6	41.17 (15.82)	24.57, 57.77
Pain beliefs, SOPA (0=best, 28=worst)						
Baseline	24	14.71 (3.63)	13.18, 16.24	12	13.50 (4.15)	10.87, 16.13
3 months	19	13.95 (4.79)	11.64, 16.26	8	10.63 (3.38)	7.80, 13.45
6 months	18	13.72 (3.83)	11.82, 15.63	6	10.50 (2.43)	7.95, 13.05
Fear of movement, TSK-13 (13=best, 52=worst)						
Baseline	24	27.13 (8.46)	23.55, 30.70	12	28.58 (9.64)	22.46, 34.71
3 months	19	26.53 (9.77)	21.82, 31.23	8	29.50 (10.32)	20.87, 38.13
6 months	18	24.44 (9.43)	19.75, 29.13	6	31.17 (8.75)	21.98, 40.35

Table 7 reports descriptive statistics only to assess feasibility of outcome-measure collection, completeness and score distributions for a future definitive trial. No inferential between-group comparisons were planned or undertaken.

AQoL, Assessment of Quality-of-Life (relationships and self-worth domains only; EPIC-Neck, Exercise Prescription Improved through Co-design-Neck; EQ-5D-5L, EuroQol 5-Dimension 5-Level; HADS, Hospital Anxiety and Depression Scale (Anxiety and depression scores combined); NDI, Neck Disability Index; NPRS, Numeric Pain Rating Scale; PSEQ, Pain Self-Efficacy Questionnaire; PSFS, Patient-Specific Functional Scale; SCI, Sleep Condition Indicator; SOPA, Survey of Pain Attitudes Questionnaire (single item version); TSK, Tampa Scale of Kinesiophobia; VAS, Visual Analogue Scale.

### Progression to a definitive trial

Table 1 summarises feasibility findings against the pre-specified traffic-light criteria: green for recruitment and contamination, amber for delivery fidelity and acceptability, and red for retention. Overall, progression was considered feasible with modification, specifically to strengthen retention, delivery fidelity and intervention coherence.

## Discussion

This feasibility trial aimed to determine whether a definitive trial evaluating the clinical and cost-effectiveness of EPIC-Neck for chronic non-specific neck pain is feasible. The feasibility findings were mixed. Recruitment and contamination met the green progression criteria, indicating no major concerns in these domains. However, retention met the red criterion and delivery fidelity met the amber criterion, indicating important feasibility concerns that require resolution before a definitive trial. Acceptability to both patients and physiotherapists was broadly positive, but qualitative findings identified addressable concerns within the TFA domain of intervention coherence, particularly participants’ understanding of the intervention rationale and physiotherapists’ clarity around delivering newer or more complex components.

### Recruitment was feasible, but retention posed modifiable challenges

Despite not reaching the estimated sample size, 4–5 participants were randomised per month, exceeding the green progression criteria. In contrast, the 6-month retention rate was 67%. This is below the ≥80% target associated with lower risk of attrition bias and below the minimum 70% threshold pre-specified for progression, raising concerns about whether current retention strategies would be sufficient in a definitive trial.^41–45^

Standard retention strategies were used (linguistic support, pre-paid envelopes, reminders and financial incentives)^44 77 78^; while further optimisation may help (electronic and shorter questionnaires, increased incentives),^44 79^ our findings suggest that retention may be influenced by treatment allocation and engagement rather than by follow-up logistics. Retention differed by treatment allocation (50% usual exercise care vs 75% EPIC-Neck), and none of the participants who did not initiate treatment or were discharged for non-attendance returned follow-up measures. In contrast, treatment completers across both groups had 91% retention (22/24). Additionally, treatment completion was higher in EPIC-Neck (75%) than in usual exercise care (50%). Taken together, these patterns indicate engagement-related attrition and possible allocation-preference effects within this unblinded feasibility trial, reinforcing the need to consider participant blinding in the definitive exercise-versus-exercise trial to reduce allocation-related differences.^80 81^

### Intervention coherence and delivery are suboptimal

Although overall acceptability was broadly positive, there were clear concerns within the intervention coherence domain, particularly among participants. Many had limited understanding of the programme rationale and performance objectives. These coherence deficits align with lower delivery fidelity scores for the corresponding components (mean scores <3), suggesting that limited patient understanding may have been related, at least in part, to suboptimal physiotherapist delivery. Physiotherapists also reported reduced confidence and clarity when delivering newer or more complex EPIC-Neck components.

Addressing these issues is crucial before progressing to a definitive trial. Poor fidelity can reduce treatment effects,^82^ risk misinterpreting null findings^83 84^ and threaten internal validity.^85^ However, delivery fidelity should not be interpreted as a simple success/failure judgement. Although only 14% of participants received EPIC-Neck at an acceptable overall fidelity level, 35% of components were delivered acceptably, which means that some intended intervention mechanisms may still have been activated. Evidence from complex behavioural interventions also indicates that effectiveness may still be possible despite variable or moderate delivery fidelity, depending on which components are most critical to the intervention mechanism.^47 86 87^ Nevertheless, fidelity concerns remain substantial because lower-fidelity EPIC-Neck components included those intended to support long-term self-management, such as flare-up management and general exercise, which may plausibly influence long-term clinical outcomes in a definitive trial. These concerns are modifiable. Consistent with Medical Research Council guidance, EPIC-Neck and its training package can be refined and re-tested, with a focus on strengthening delivery fidelity and intervention coherence before progressing to a definitive evaluation.^22^

### EPIC-Neck was acceptable with concerns about intervention coherence and was distinct from usual exercise care

Despite concerns about intervention coherence preventing immediate progression to a definitive trial, EPIC-Neck was broadly acceptable across the remaining domains to both patients and physiotherapists. Additionally, the operationalisation of EPIC-Neck components (eg, behaviour change strategies and performance objectives) appears different from usual exercise care.^88^ This reduces the risk of contamination in a definitive trial diluting any between-group effect.^89–91^ Overall, EPIC-Neck appears acceptable and sufficiently distinct from usual exercise care but requires refinement and further fidelity testing before definitive evaluation.

### Future refinement before a definitive trial

Before progression to a definitive trial, key refinements are needed to optimise retention, delivery fidelity and intervention coherence. Retention strategies should address both questionnaire return and treatment engagement, as missing follow-up data appeared linked to treatment non-initiation and non-attendance. Future procedures could consider reducing questionnaire burden (eg, electronic completion or shorter questionnaires), increasing the financial incentive for completion and incorporating blinding procedures where feasible.^44 79–81^ Additional strategies to optimise delivery and intervention coherence should be designed collaboratively with patients and physiotherapists and informed by theory and existing evidence. Potential refinements include strengthening physiotherapist training through longer training duration, additional practice and regular monitoring and feedback.^92–96^ Intervention coherence may improve through better delivery, but additional strategies could include methods to check patient understanding during treatment, such as teach-back approaches.^97^

### Strengths and limitations

A key strength of this trial is the integration of quantitative and qualitative data, incorporating perspectives from both patients and physiotherapists, enabling a more comprehensive assessment of feasibility. A further strength is that 94% of appointments were audio-recorded, providing a robust assessment of delivery fidelity and contamination.^98^ Finally, efforts to minimise exclusion based on English language and age resulted in a diverse cohort (40% Asian; age range 19–82 years), enhancing external validity by better reflecting populations attending NHS musculoskeletal services.^29^

The target sample size was not reached, which limits the precision of feasibility estimates and means that the progression criteria and subgroup patterns should be interpreted with caution. The 2:1 allocation ratio increased intervention exposure and feedback but resulted in fewer usual exercise care participants, reducing the precision of usual-care estimates and limiting interpretation of between-group descriptive patterns. Participants and outcome assessors were not blinded, which may have introduced performance or detection bias, particularly for self-reported outcomes; blinding of outcome assessment will therefore be incorporated into the definitive trial where feasible. Physiotherapists delivered EPIC-Neck to relatively few patients; therefore, some fidelity issues may be overestimated and could improve with increased exposure and practice.^99 100^ Fidelity assessments were undertaken unblinded, which may have influenced scoring despite the use of a structured competence checklist. In addition, interviews were conducted by the intervention developer, whose role in EPIC-Neck development may have introduced social desirability and allegiance bias. The use of topic guides, reflective discussions and team-based interpretation helped mitigate this risk, but independent interviewing and analysis would strengthen future evaluations.

## Conclusion

The EPIC-Neck programme shows potential for evaluation in a definitive trial, but progression is conditional on further refinement. Recruitment and contamination findings were encouraging, and the intervention was broadly acceptable and distinct from usual exercise care. However, retention, delivery fidelity and intervention coherence require optimisation before definitive evaluation. Future work should refine the EPIC-Neck programme, training package and trial retention procedures, followed by targeted repeat testing of delivery fidelity, participant and physiotherapist understanding of the intervention and retention. Depending on the extent of these refinements, this may require a further feasibility or pilot study before progressing to a full-scale definitive trial.

## Supplementary Material

Reviewer comments

Author's manuscript

## Data Availability

Data are available upon reasonable request. Data sharing requests can be considered via contact with the corresponding author.
