# Supplementary material for: Co-developed neck exercise (EPIC-Neck) versus usual exercise care for people with chronic non-specific neck pain: a UK multicentre randomised feasibility trial
Source: BMJ Open. 2026 Oct 20;16(10):e120571. doi: 10.1136/bmjopen-2026-120571 (PMC13711528; doi:10.1136/bmjopen-2026-120571)
Supplement: Supplementary file 3 [file bmjopen-16-10-s003.pdf]

SUPPLEMENTARY FILE 3 – SUPPORTING INFORMATION

Figure S3-1 – EPIC-Neck behaviour change facilitation guide

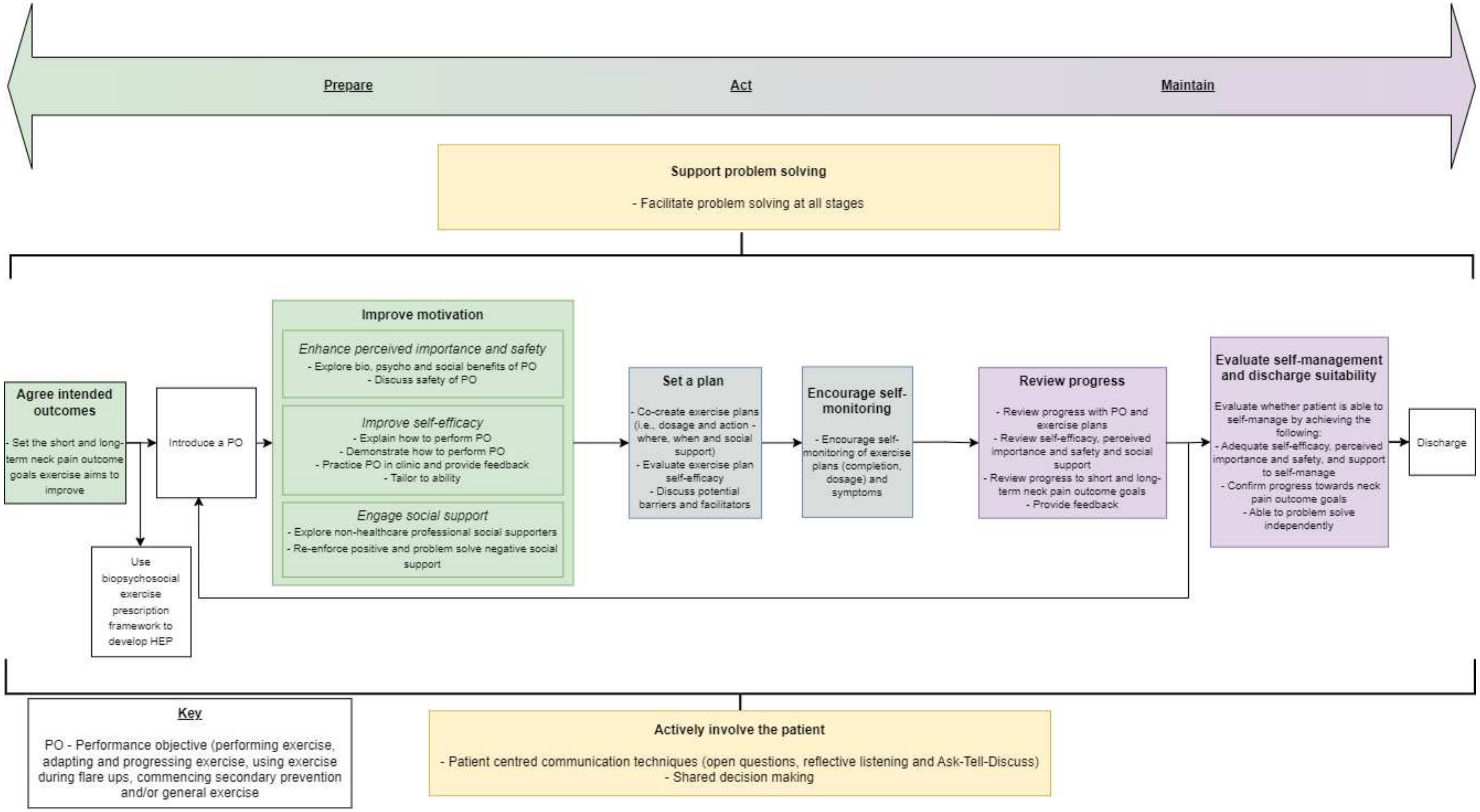

Price J, et al. BMJ Open 2026; 16:e120571. doi: 10.1136/bmjopen-2026-120571

Figure S3-2 – EPIC-Neck biopsychosocial exercise prescription framework

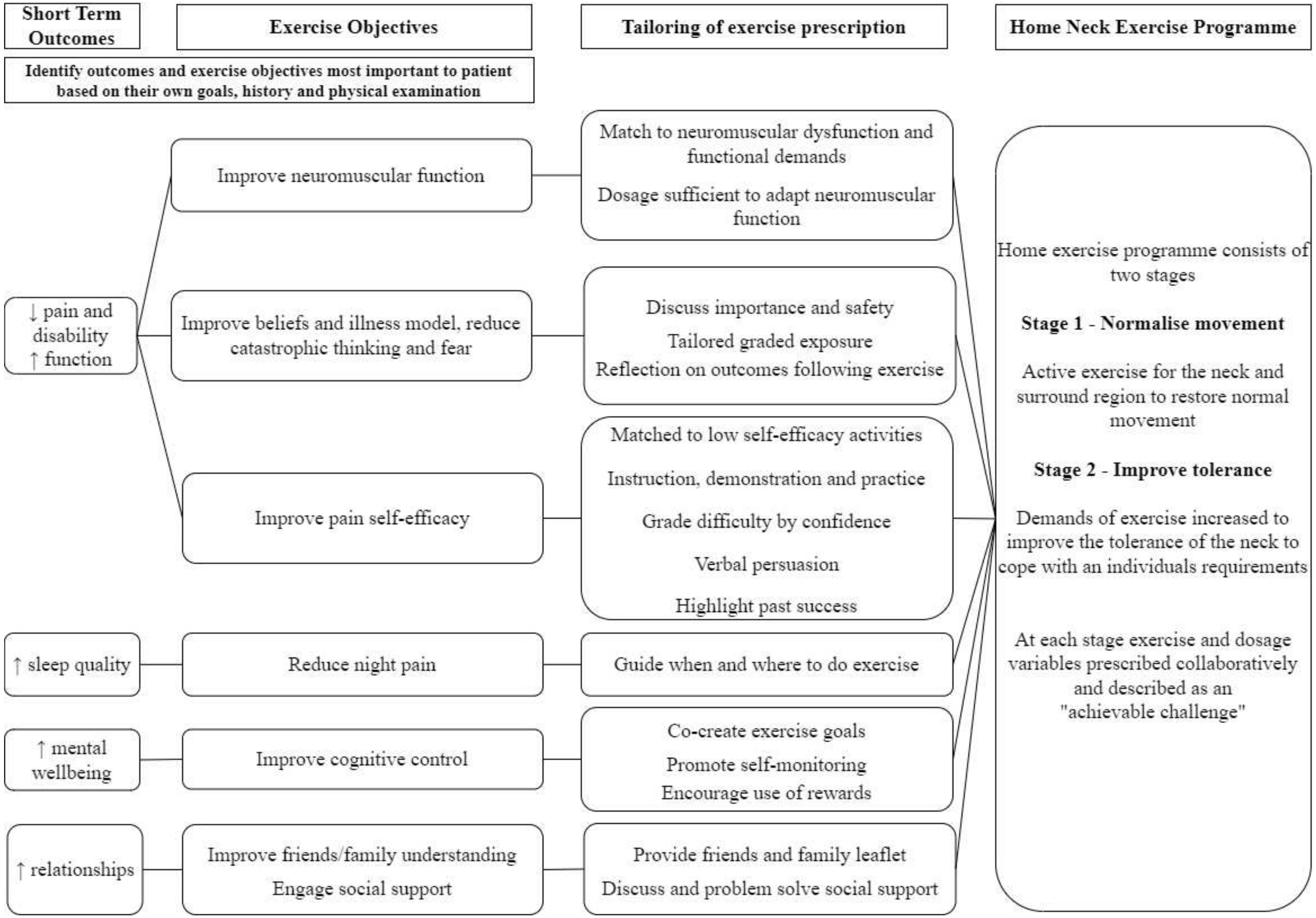

Table S3-1 – Delivery fidelity checklist

Interventional Techniques	Perfect - 5	Acceptable - 3	Scoring Notes
**<u>Intervention components to maximise engagement</u>**			
**Agree outcome goals:** The short and long-term neck pain outcome goals exercise aims to improve are determined *(Max score)*<br>***Notes:*** *Score 0 at follow-up if no new goals are set-up if no new goals are set*			
1. Set short (≤3 months) and long-term (12 months) outcome goals linked to current restrictions or limitations.	Goals verbally agreed for 3 and 12 months<br>Goals verbally and explicitly linked to patient's restrictions/limitations.<br>Goals set collaboratively using ASK-TELL-DISCUSS. DISCUSS is essential for a 5.	Goals verbally agreed and linked to restrictions/limitations.<br>Physio suggests goals before asking patient's preference.	Physio-suggested goals not explicitly linked to restrictions → max 2.<br>Goals not verbally agreed & explicitly linked → max 2.
2. Ensure outcome goals are measurable.	Goals are measurable and include clear targets (e.g., "walk 20 minutes without pain").	Goals are measurable but lack specific targets (e.g., "walk more").	
3. Encourage recording outcome goals in the workbook.	Physio physically shows the patient where in the workbook/wall chart to record the goals	Physio mentions recording but does not physically direct.	
**Actively involve the patient:** Patient centred communication style used throughout to encourage patients' active involvement in the appointment and decision making *(Mean score)*			
1. Predominant use of open questions to elicit information.	Predominant use of open questions.	Some open questions but reliance on closed questions.	Only 1–2 open questions → max 2.

Price J, *et al. BMJ Open* 2026; 16:e120571. doi: 10.1136/bmjopen-2026-120571

**Interventional Techniques**	**Perfect - 5**	**Acceptable - 3**	**Scoring Notes**
Closed questions are permitted where necessary.	Closed questions only when necessary.	Open question immediately followed by closed before patient responds.	
2. Use of Ask-Tell-Discuss techniques to help with information exchange. Needs to be all at the same time when discussing a concept/idea/barrier.	Full ASK-TELL-DISCUSS used in a single exchange.<br>ASK and DISCUSS prompt use open questions.	ASK-TELL used but no discussion.	TELL only → max 1.<br>DISCUSS prompted with closed question → max 4.
3. Use of reflective listening e.g., repetition, paraphrasing and reflecting feelings	Reflective listening immediately after important information is provided.<br>Includes summarisation at key points.	Summarisation/Reflective listening used intermittently, but key points may be missing.	
4. Shared decision making used throughout appointment	Open questions used to prompt patient's input in decision-making or to confirm a physio decision is acceptable (e.g., physio sets dosage and asked for patient opinion [open question])	Closed questions used to confirm acceptance of physio's decision. To score 3 must be used regularly throughout the appointment. Physio decision making based on information provided by patient throughout appointment or prior appointment.	Fully physio-led decisions (no patient input) → score 0.<br>1–2 closed-question confirmations ("is that ok?") → max 2.
**Improve motivation to perform exercise (PO1) - Enhance perceived importance and safety** - Physiotherapist helps to develop /enhance a patient's understanding of the importance and safety of performing exercise *(Max score)*			
1. Explore and seek to enhance the patient's perception of the bio, psycho, and social (relationships & social activities) benefits of	Uses ASK-TELL-DISCUSS to explore that patient's perception of bio, psycho and social benefits.	Physio states the bio, psycho and social benefits of exercise. All 3 essential to score a 3.	Only bio discussed → max 1.<br>Two of the three domains discussed → max 2.

Interventional Techniques	Perfect - 5	Acceptable - 3	Scoring Notes
performing exercise and of reducing neck pain using Ask-Tell-Discuss.	Discuss after telling is essential to score a 5.<br>Physio links benefits back to key findings from history or patient goals		
2. Discuss /explain the safety of performing exercise.	Use of ASK-TELL-Discuss to explore safety of exercise.<br>Discuss is essential to score a 5.	States that exercises are safe but does not explore patient concerns or thoughts in relation to safety.	
**Improve motivation to perform exercise (PO1) - Improve self-efficacy** - Physiotherapists uses techniques to help improve a patient's self-efficacy for performing exercise *(Mean score)*<br>***Notes:*** *Score NA if no new or modified exercises; NA if review identifies no issues.*			
1. Provide accurate verbal or written instruction on how to perform exercise.	Instructions clearly understandable from audio.<br>Exercises are explicitly part of programme and formally "set."	Instructions provided but may still be unclear from audio.	Audio quality prevents evaluation → score 3.
2. Physio to physically demonstrate how to perform exercise.	100% of prescribed exercises demonstrated by physiotherapist. Acceptable to use video to demonstrate exercises.	The majority (≥66%) of exercises prescribed demonstrated by the physiotherapist.	Printouts/static images used → max 2.
3. Patient practices exercise in clinic and physio provides feedback.	Patient practices all exercises (full dosage not required).<br>Detailed feedback provided with reinforcement or corrections.	Patient practices most exercises (≥66%).<br>Brief feedback given (e.g., "good job").	No feedback → max 2.

Interventional Techniques	Perfect - 5	Acceptable - 3	Scoring Notes
4. Tailor exercise to patients' ability and goals.	Exercise position/action linked to goal and physio explicitly states/demonstrates how<br>Exercises should be achievable for patients to perform.<br>Physio may change position/exercise if too difficult or too easy.<br>Sense checked with patient that exercise is suitable for ability through questioning (open or closed permitted)	Exercise appears reasonable for patients' ability. If states dosage to be manipulated to be a challenge, then score 3.<br>Must be practiced in clinic to score a 3.<br>Physio does not sense check with patient<br>Physio does not explicitly and verbally link to goals	No practice → score 0.<br>No practice but gives reps/adjustments as "challenge" → max 1.<br>No goals set → max 4.
5. Direct to Module 2 of the workbook for more detail.	Physio physically shows the patient the exercise library highlighting exercise within their programme.	Physio states there is an exercise library, and exercises can be found in there.	
**Improve motivation to perform exercise (PO1) - Engage social support** - Physiotherapists supports patient in engaging social support to assist with performing exercise programme *(Max score)*<br>***Notes:*** *Social-support discussion must relate to the exercise programme (not overall neck-pain management) → otherwise score 0.*			
1. Explore existing social support (both positive and negative).	Use of open questioning to explore what social support potentially exists to support with the exercise programme (can be when exercise prescription or at review appointments)	Use of closed questioning to explore social support with exercise (can be when exercise prescription or at review appointments)	

**Interventional Techniques**	**Perfect - 5**	**Acceptable - 3**	**Scoring Notes**
	Follow-up or further questioning is completed to elicit detail and full understanding of support to identify barriers or facilitators	Little to no follow-up questioning to understand barriers or facilitators	
2. Re-enforce positive social support. This may include informational/planning support, emotional support /approval, or practical support (e.g. helping the patient to make time for exercising).	Physio uses reflective listening skills to summarise positive social support with respect to exercise and re-enforces	Physio simply states that any support is positive, without the use of any reflective listening	No positive support identified → score NA.<br>Social support not evaluated → score 0.
3. Problem solves negative social support.	Physio uses reflective listening skills to summarise key points regarding the negative social support.<br>ASK-TELL-DISCUSS to facilitate problem solving<br>Discuss essential to score a 5<br>A menu of options provided when using TELL<br>Re-enforce positive solutions provided by patient using reflective listening techniques	Physio ASKs patient for solutions with how to improve social support (not how social support can help exercise).<br>Physio TELLs a solution. No options provided. No option to discuss. .	Physio only tells solutions → max 2.<br>DISCUSS prompted with a closed question → max 4.<br>No negative support identified → score NA.<br>Social support not evaluated → score 0.
4. Provide equipment as required.	5 if equipment given or patient has alternatives at	-	No equipment required → score NA.

Interventional Techniques	Perfect - 5	Acceptable - 3	Scoring Notes
	home (e.g., something to use as a weight [bag etc])		
5. Direct to the friends and family leaflet.	Physio physically shows the patient the friends and family leaflet and explains its benefits<br>If a social support is present physio provides them with the leaflet and explains its benefits	Physio states there is a friends and family leaflet. Benefits not explained.	Supporter present but not shown leaflet → max 4.
**Set a plan to perform exercise (PO1)** - Physiotherapist assists patient with planning exercise (i.e., dosage and action plans) *(Max score)*<br>***Notes:*** *Score NA if no new or modified exercises; NA if review identifies no issues.*			
1. Make an exercise dosage plan including frequency, reps, sets, position, and intensity of effort.	Dosage clearly articulated and clearly understood from audio-recordings<br>Minimum of frequency, reps, sets described.	Dosage given but may be unclear or provided in a fragmented way.<br>Only 2/3 of frequency, reps, sets are described	Only one dosage element provided → max 2.<br>Dosage written but not fully discussed → max 3.<br>Modification/progression of existing exercises only → score NA.
2. Co-create an exercise action plan with the patient, including where, when, and how social support will be engaged (either verbally or written).	ASK-TELL-DISCUSS used to outline where, when and how social support will be used<br>Discuss is essential for a 5.	Physio states, where, when and how social support will be used<br>No ASK or DISCUSS	Where/when/social support not all covered → max 2–3 (depending on detail).<br>Patient suggests only a single item with little physio reinforcement → score 1.

Price J, *et al. BMJ Open* 2026; 16:e120571. doi: 10.1136/bmjopen-2026-120571

Interventional Techniques	Perfect - 5	Acceptable - 3	Scoring Notes
3. Evaluate self-efficacy with completing exercise dosage and action plans.	Open questioning use to evaluate confidence in completing the exercise programme and plan/Uses a 0-10 scale	Uses a closed question to evaluated confidence	Only brief closed-question check (“is that ok?”) → max 2.<br>Confidence not asked explicitly → max 2.
4. Discuss potential barriers and facilitators (can be completed at any appointment).	Use physios open questioning to identify any potential barriers to completing exercise.<br>Further detail and questioning used to understand barrier/facilitators.<br>Reflective listening techniques used to summarise and highlight key barriers<br>If problems identified, problem solving scored elsewhere (Problem solving).	Physio uses closed questioning to identify any potential barriers/facilitators.<br>Reflective listening techniques used but maybe weak<br>If problems identified, problem solving scored elsewhere (Problem solving)	Pre-emptive exploration of barriers/facilitators only; actual barriers after implementation in “Reviewing progress & Problem solving”<br>Closed-question exploration → max 3.<br>Patient offers barrier/facilitator but not acknowledged → max 2.
5. Direct to workbook to record exercise dosage goals and exercise action plan.	Physio physically shows the patient where to record their exercise programme and dosage plan (can be exercise diary) AND action plan.	Physio states there is space for them in the resources to record their exercise dosage and action plan. Action plan essential for a 3.	Score recording of completion in “Self-monitoring”
**Encourage self-monitoring (PO1)** - Physiotherapist encourages patient to self-monitor exercise *(Max score)*<br>***Notes:*** *Diary review scored under “Review progress”.*			

Interventional Techniques	Perfect - 5	Acceptable - 3	Scoring Notes
1. Discuss the benefits of self-monitoring exercise completion, dosage, and symptoms (gives feedback on progress, provides a basis for discussion with the PT at the next appointment).	Physio uses ASK-TELL-DISCUSS to explore the benefits of self-monitoring<br>Explicitly links to goals or exercise objectives/functional limitations	Physio explicitly states the benefits of self-monitoring. Needs to be more than "it's a good idea".	
2. Direct to workbook (exercise diary/wall chart/app) for self-monitoring.	Physio physically shows the patient where to record their exercise completion and explains how to use it.	Physio states there is space for them in the resources to record their exercise completion	"There is space to record" only (no physical direction) → max 3.<br>Encouraging use (but no physical showing) → max 4.
**Review progress** - Physiotherapist reviews progress *(Mean score)*<br>***Notes:*** *Scored as NA for initial appointment; Understanding progress only → actual barriers/facilitators are scored under Support problem solving.*			
1. Review of short and long-term neck pain outcomes (the initial outcome goals set and any other benefits or harms noticed by the patient).	Open questioning to elicit changes in patient symptoms and goals.<br>Detail/follow-up questions to explore/understand changes (or harms)<br>Reflective listening used to highlight key changes	Closed questioning used to elicit changes<br>Weak or limited follow-up to gain understanding of changes/progress<br>No reflective listening	
2. Review of performance objectives and exercise plans.	Open questioning to elicit progress with exercise and other performance objectives (once introduced).	Closed questioning to elicit progress/compliance with exercise and other performance objectives (once introduced).	PO must be evaluated at the next session after it was introduced → needed for score 3.

Interventional Techniques	Perfect - 5	Acceptable - 3	Scoring Notes
	Detail/follow-up questions asked to understand detail behind how exercise or performance objectives are being performed/engaged with/understand exercise.<br>Reflective listening used to highlight key changes and any potential barriers/positives stuff	Short or minimal questioning to understand detail behind how exercise or performance objectives are being performed/engaged with/understand exercise.<br>No reflective listening	
3. Review motivation (importance, safety, self-efficacy and social support)	Open questioning to review understanding of benefits and safety of performance objectives/exercise<br>Open questioning (or a 0-10 scale) to review confidence in completing performance objectives/exercise<br>Open questioning to review completion of action plan and social support<br>Reflective listening to summarise and highlight any key issues/re-enforce positive changes	Close questioning used and only 2/3 explored out of<br>1. Benefits/safety<br>2. Confidence<br>3. Action plan/social support<br>Limited reflective listening	
4. Provide feedback on progress with neck pain outcome goals and performance objectives.	Uses a formal summary to recap.<br>Progress with symptoms/goals,	Absence of a formal summary<br>Physio states someone is doing well or not doing well	

Interventional Techniques	Perfect - 5	Acceptable - 3	Scoring Notes
	POs/exercise and motivation factors all mentioned.<br>Strong reflective listening that highlights and emphasises key findings (both positive and negative)	(symptoms/goals AND exercise/POs).<br>Misses feedback on motivational factors<br>Weak reflective listening with weak highlighting of key findings (both positive and negative)	
**Support problem solving** - Physiotherapist supports problem solving throughout intervention delivery. Most commonly used when reviewing progress *(Mean score)*<br>***Notes:*** *Initial appointment = NA unless barriers raised; NA if "Review Progress" item 2 scores ≥3 and no issues reported with exercise*			
1. Discuss barriers to exercise or performance objective completion.	If when reviewing progress issues with exercise/PO identified, barriers to completion explored using open questioning.<br>Good follow-up questioning to gain depth to understanding of all barriers<br>Strong reflective listening skills	If when reviewing progress issues with exercise/PO identified, barriers to completion explored using closed questioning.<br>Limited depth to understanding barrier(s). May only explore one barrier.<br>No reflective listening	If issues with PO/exercise plan are unknown due to Reviewing Progress Point 2 → max 3.<br>Patient mentions barrier but physio does not follow up → max 2.
2. Facilitate problem solving.	Uses ASK-TELL-DISCUSS to facilitate problem solving<br>Discuss essential to score a 5<br>A menu of options provided when using TELL	Physio ASKs patient for solutions.<br>Physio TELLs a solution. No options provided. No option to discuss.	Problem-solving relating to flare-ups → score in PO3.<br>Physio stating a single solution and patient challenges/declines → max 1<br>DISCUSS prompted with closed question → max 4.

Interventional Techniques	Perfect - 5	Acceptable - 3	Scoring Notes
	Re-enforce positive solutions provided by patient using reflective listening techniques		
3. Direct to FAQ of the workbook.	Physio physically shows the patient the FAQ sections and used to facilitate discussion.	Physio states there is an FAQ section and states it is to support problem solving.	
**Introduce adapting and progressing (or regression) exercise (PO2)** - Physiotherapist discusses adapting and progressing exercise *(Max score)*			
1. Discuss the bio, psycho, and social (relationships & social activities) benefits and safety of adapting and progressing (or regressing) exercise.	Physio uses ASK-TELL-DISCUSS to explore that patient's perception of bio, psycho and social benefits of progressing or regressing exercise. Discuss after telling is essential to score a 5.<br>Physio links benefits back to key findings from history or patient goals<br>Use of ASK-TELL-Discuss to explore safety of exercise.<br>Discuss is essential to score a 5.	Physio states the bio, psycho and social benefits of progress or regressing exercise. All 3 essential to score a 3.<br>Physio states the exercises are safe to complete. No use of ASK-TELL-DISCUSS	Only biological benefits discussed → max 1.<br>Two of three domains discussed → max 2.
2. Instruct and demonstrate options of how to adapt and progress (or regress) exercise.	Verbal instructions must be clear enough to be understood the exercise via audio-recording.<br>Demonstrates any changes that reasonably require a	Instructions for progression only.<br>Verbal instruction provided, may still be unclear from audio-recording.	Verbal instruction unclear (from audio) → score 3.<br>Demonstration not required when only reps/weight change (already loaded).

Interventional Techniques	Perfect - 5	Acceptable - 3	Scoring Notes
	demonstration (e.g., changing position, or adding a weight to an unweighted exercise)	Demonstration not provided when reasonably could have been (e.g., no weight to dumbbell)	
3. Co-create a tailored plan to patients' ability and goals, outlining how and when to adapt and/or progress exercise, and how they can use social support for support (either verbally or written).	Plan clearly linked to ability and goals<br>Physio provides options of how to progress/regress exercise and patient choose which they prefer.<br>Physio articulates when they need to adapt exercise (use of achievable challenge acceptable but not essential).<br>ASK-TELL-DISCUSS used to explore how social support can be used	Plans unclear how linked to ability or goals<br>Physio states how to progress or regress exercise (no patient choice)<br>Physio articulates when they need to adapt exercise (use of achievable challenge acceptable but not essential).<br>Social support not discussed	"How" here is the co-creation of a plan using options to try and deciding. The instructions of how are scored above.<br>Same plan described for only one exercise without generalisation → max 3.
4. Discuss potential barriers (including self-efficacy) and facilitators to adapting or progressing exercise (can be completed at any stage).	Use physios open questioning to identify any potential barriers to progressing/regressing exercise.<br>Further detail and questioning used to understand barrier/facilitators.<br>Reflective listening techniques used to	Physio uses closed questioning to identify any potential barriers/facilitators.<br>No follow-up questioning<br>Reflective listening techniques used to summarise and highlight key barriers<br>If problems identified, problem solving scored elsewhere (Problem solving)	Pre-emptive exploration of barriers/facilitators only; actual barriers after implementation in "Reviewing progress & Problem solving"

Interventional Techniques	Perfect - 5	Acceptable - 3	Scoring Notes
	summarise and highlight key barriers<br>If problems identified, problem solving scored elsewhere (Problem solving).		
5. Practice adapting and/or progressing exercise in clinic and provide feedback.	Patient practices the modifications to progress/ regression (includes repetition changes)<br>Detailed feedback provided either using either positive re-enforcement if done well, or to correct technique	Patient practices any modifications.<br>Feedback is brief (e.g., well done, good)	Only brief/non-specific feedback (“good”, “well done”) → max 3.
6. Direct to Module 3 of the workbook for more detail.	Physio physically shows the patient the relevant module and uses it as a guide to discuss the performance objective.	Physio states there is a section in the workbook on the performance objective.	
**Introduce flare-up exercise (PO3)** - Physiotherapist discuss flare-ups and how they can be self-managed with exercise *(Max score)*			
1. Explore the benefits and safety of self-managing a flare-up with exercise.	Physio links benefits back to key findings from history or patient goals<br>Physio uses ASK-TELL-DISCUSS to explore that patient’s perception of the benefits of managing a flare-up with exercise. Discuss	Physio states any benefits of managing a flare-up with exercise.<br>Physio states that managing a flare-up with exercise independently is safe to complete. No use of ASK-TELL-DISCUSS	

Interventional Techniques	Perfect - 5	Acceptable - 3	Scoring Notes
	after telling is essential to score a 5.<br>Use of ASK-TELL-Discuss to explore safety managing a flare-up independently with exercise. Discuss is essential to score a 5.		
2. Co-create a flare-up exercise plan including which exercises and when to seek healthcare professional support. Plan how to use social support (either verbally or written).	Uses open questioning to prompt problem solving for anticipated flare-ups.<br>Physio provides the options of 1. Regress 2. Flare-up programme 3. Restart old exercises (any other options do not score a 5)<br>Use of ASK-TELL-DISCUSS to explore social support. Discuss essential for a 5.<br>Physio states when to seek HCP support.	Physio states/tells a single option for flare-up exercise. May or may not be the 3 options in the workbook/to score a 3.<br>Physio states/tells how to engage social support	No non-HCP social support discussion → max 2.
3. Use any feedback when reviewing progress to reinforce self-efficacy (if a flare-up plan was implemented) or to problem-solve (if something didn't go according to plan).	Open questioning to explore how exercise was used the manage the flare-up<br>Use of reflective listening to highlight key barriers, or issues/Use of reflective listening to highlight what was done well	Closed questioning reliance to explore how exercise was used<br>No summaries or reflective listening.<br>Physio states how to problem-solve exercise for flare-ups	No flare-up between appointments → score NA.<br>Only non-exercise modalities discussed → max 2.<br>Reviewing progress/problem solving here is related to the success of managing a flare-up (outcome). Any barriers/facilitators to

Interventional Techniques	Perfect - 5	Acceptable - 3	Scoring Notes
	Re-enforce and praise any positive actions.<br>Use of ASK-TELL-DISCUSS to problem solve how exercise was implemented for the flare-ups		implementation of the exercise scored under Reviewing Progress/Problem Solving.
4. Direct to the Module 4 of workbook for more detail.	Physio physically shows the patient the relevant module and uses it as a guide to discuss the performance objective.	Physio states there is a section in the workbook on the performance objective.	
**Introduce general exercise +/- secondary prevention exercise (PO4)** - Physiotherapist encourages general exercises and discusses concept of secondary prevention exercise *(Max score)*			
1. Discuss bio, psycho, and social (relationships & social activities) benefits of secondary prevention and general exercise.	Physio states the pros and cons of secondary prevention exercise<br>ASK-TELL-DISCUSS to elicit views of bio, psycho and social benefits of general exercise. Discuss and all 3 domains essential for a 5.	Physio states the pros and cons of secondary prevention exercise<br>Physio states the bio, psycho and social benefits of general exercise.	Two of three domains discussed → max 2.
2. Set long-term general exercise goals.	Goals set that are measurable.<br>Can be behavioural (exercise 5 times per week) or outcome (lose weight)	Goals set that are not measurable<br>Can be behavioural (exercise 5 times per week) or outcome (lose weight)	

Price J, *et al. BMJ Open* 2026; 16:e120571. doi: 10.1136/bmjopen-2026-120571

Interventional Techniques	Perfect - 5	Acceptable - 3	Scoring Notes
3. Set a general exercise +/- secondary prevention exercise plan.	Collaborative decision between patient and physio as to whether to engage with secondary prevention exercise<br>ASK-TELL-DISCUSS to set an action plan of what, where, when and who can support general exercise. Discuss essential for a 5.<br>ASK-TELL-DISCUSS how to integrate secondary prevention exercise if opting in. Discuss essential for a 5.	Collaborative decision between patient and physio as to whether to engage with secondary prevention exercise<br>ASK-TELL to set an action plan of what and who can support general exercise (where and when not essential for a 3)<br>Physio states how to integrate secondary prevention exercise if opting in.	
4. Encourage self-monitoring.	Physio uses ASK-TELL-DISCUSS to explore the benefits of self-monitoring	Physio explicitly states they should self-monitoring general exercise	
5. Direct to Module 5 of the workbook for more detail.	Physio physically shows the patient the relevant module and uses it as a guide to discuss the performance objective.	Physio states there is a section in the workbook on the performance objective.	
**Evaluate self-management and discharge suitability** - Physiotherapists evaluates whether a patient is able to self-manage and therefore suitable for discharge *(Max score)*			
1. Initiate conversations regarding discharge.	Physio asks patient opinion on discharge using open questions.	Physio states they are ready for discharge	

Interventional Techniques	Perfect - 5	Acceptable - 3	Scoring Notes
2. Evaluate and determine adequate motivation (importance, safety, self-efficacy and social support) in using performance objectives.	Physio uses open questioning to confirm patient understand the benefits of all 4 performance objectives<br>Physio uses open questioning to confirm patient has adequate social support for exercise (not self-management of neck pain)<br>Physio uses open questioning to confirm self-efficacy with completing all 4 performance objectives	Closed questioning to confirm patient understands the benefits all 4 performance objectives<br>Closed questioning to confirm patient has adequate social support for exercise (not self-management of neck pain)<br>Closed questioning to confirm self-efficacy with all 4 performance objectives.	
3. Evaluate and confirm progress towards short and long-term neck pain outcome goals.	Physio explicitly asks patient to re-evaluate and confirm their progress towards their goals	Physio states patient is progressing towards goals (patient not asked)	No goals set but symptom changes used → max 3
4. Evaluate patient’s ability to review progress and problem solve independently post-discharge.	Physio uses open questions to hypothesis what patient would do if any exercise related problems occurred post-discharge<br>Patient answers appropriately.	Physio states the patient is able to problem solve independently	Specifically related to exercise, and use of exercise to manage symptoms.<br>Closed-question check (“can your problem-solve?”) → max 4.
**<u>Intervention components to maximise effectiveness</u>**			
**Identify short-term outcomes and exercise objectives (PO1)** - Use of history taking and physical examination to identify appropriate short-term outcomes and specific exercise objectives *(Max score)*			

Interventional Techniques	Perfect - 5	Acceptable - 3	Scoring Notes
1. Explore each potential short-term outcome (pain, disability/functional limitations, sleep, mental well-being and relationship impact) in history taking.	All domains explored with use of open questioning<br>Reflective listening used to highlight key findings within each domain<br>Summary used at the end of history to reflect each of the domains, and to highlight which domain(s) are a priority.	All domains explored with reliance on closed questioning	
2. Explore exercise objectives (fear/catastrophising and pain self-efficacy) for pain and disability.	All objectives explored with use of open questioning<br>Reflective listening used to highlight key findings within each objective<br>Summary used at the end of history to reflect each of the objective(s), and to highlight which if any are a priority.	All objectives explored with reliance on closed questioning	
3. Use physical examination techniques where required to identify and define exercise objective.	Clear and appropriate use of cervical, shoulder, scapular neuromuscular function examination articulating key findings to patient<br>Clear and appropriate use of graded exposure or self-efficacy examination where required	Basic neuromuscular function examination. May miss some areas or components<br>Brief use of graded exposure or self-efficacy examination where required	Neuro/myotomal testing only → max 2.<br>No cervical neuromuscular function evaluation → max 4.

Price J, *et al. BMJ Open* 2026; 16:e120571. doi: 10.1136/bmjopen-2026-120571

**Interventional Techniques**	**Perfect - 5**	**Acceptable - 3**	**Scoring Notes**
**Home exercise programme – Normalising movement (PO1)** - Home exercise programme prescribed *(Max score)*<br>***Notes:*** *Score NA on follow-up if previously prescribed and no new exercises.*			
1. Exercise programme utilises active exercise for the neck or surrounding regions aiming to restore movement that maybe dysfunctional due to any reason (if appropriate)	Appropriate exercises prescribed<br>Purpose of exercises clearly articulated as restoring normal movement	Largely appropriate exercises prescribed<br>Purpose of exercises not articulated to patient	Passive stretches relied on → max 2.
2. Exercises tailored to goal, examination findings and exercise objectives	Exercises matched to goals<br>Exercises clearly and appropriately matched to examination findings<br>Exercises tailored to appropriate exercise objectives<br>Physio explicitly states how they are linked to goals and examination findings	Exercises matched to examination findings but not necessarily tailored to goals or exercise objectives.<br>Link between exercises and examination findings may not be explicitly clear or expert	No goals set → max 4.
3. Exercise and dosage prescribed collaboratively to be an “achievable challenge”.	Exercise dosage clearly set by physiotherapists<br>Exercise dosage reasonable to affect the desired target<br>Open questioning used to determine acceptability to patient<br>Benchmarked against achievable challenge	Exercise dosage set but potentially unclear<br>Exercise dosage may not be appropriate/effective for desired target<br>Some attempts to benchmark against “achievable challenge” or patients’ effort.	Fully self-directed (“achievable challenge” only) and not contextualised as fear/catastrophising → max 2.
**Home exercise programme – Improving tolerance (PO1)** - Home exercise programme prescribed *(Max score)*			

Price J, *et al. BMJ Open* 2026; 16:e120571. doi: 10.1136/bmjopen-2026-120571

**Interventional Techniques**	**Perfect - 5**	**Acceptable - 3**	**Scoring Notes**
Notes: *Score NA on follow-up if previously prescribed and no new exercises.*			
1. Exercise programme utilises active exercises for the neck or surrounding regions aiming to progressively improve the tolerance of the neck to cope with increasing demands that are not just physical (if appropriate)	Appropriate exercises prescribed<br>Purpose of exercises clearly articulated as improving tolerance	Largely appropriate exercises prescribed<br>Purpose of exercises not articulated to patient	
2. Exercises tailored to goal, examination findings and exercise objectives	Exercises matched to goals<br>Exercises clearly and appropriately matched to examination findings<br>Exercises tailored to appropriate exercise objectives<br>Physio explicitly states how they are linked to goals and examination findings	Exercises matched to examination findings but not necessarily tailored to goals or exercise objectives.<br>Link between exercises and examination findings may not be explicitly clear or expert	No goals set → max 4.
3. Exercise and dosage prescribed collaboratively to be an “achievable challenge”.	Exercise dosage clearly set by physiotherapists and reasonable to affect the desired target<br>Open questioning used to determine acceptability to patient	Exercise dosage set but potentially unclear or may not be appropriate/effective for desired target<br>Some attempts to benchmark against “achievable challenge” or patients’ effort.	Fully self-directed dosage (without physio guidance) → max 2.

**Interventional Techniques**	**Perfect - 5**	**Acceptable - 3**	**Scoring Notes**
	Benchmarked against achievable challenge		
**Exercise Review** (PO1) - At follow-up appointments exercises reviewed for appropriateness and modified, progressed or regressed as needed *(Mean score)*<br>***Notes:*** *Initial appointment = NA.*			
1. At follow-up appointments exercises reviewed for continued appropriateness, and modified, progression/regressed as needed	Physio evaluates appropriateness of exercise verbally<br>Patients instructed to demonstrate all exercises<br>Repeated or further physical examination used to confirm suitability of exercises.<br>Appropriate modifications made based on demonstration, subjective and physical exam findings. If no changes must state, no changes.	Physio evaluates appropriateness of exercises verbally only<br>Modification made based on history/subjective findings only. If not, changes must state, no changes.	Appropriateness not asked (verbally or via demonstration) → max 2.<br>At discharge: no review but patient states exercise OK → max 4.<br>Significant new exercise added at discharge but not practiced → max 1. Technique modification at discharge → score 5.

Price J, *et al. BMJ Open* 2026; 16:e120571. doi: 10.1136/bmjopen-2026-120571

Table S3-2 - Return rates and completeness

Outcome	Timepoint	Returned n (%)	Initial missing items (n)	Final missing items (n)	Comments
NDI	Baseline	36 (100%)	-	-	
	3-months	27 (75%)	11	0	Difficult reading text, not working
	6-months	24 (67%)	3	0	Non-drivers
VAS	Baseline	36 (100%)	-	-	
	3-months	27 (75%)	-	-	
	6-months	24 (67%)	-	-	
PSFS	Baseline	34 (94%)	-	-	Denied functional limitations
	3-months	25 (69%)	4	0	
	6-months	22 (61%)	-	-	
SCI	Baseline	36 (100%)	-	-	
	3-months	27 (75%)	-	-	
	6-months	24 (67%)	-	-	
HADS	Baseline	36 (100%)	-	-	
	3-months	27 (75%)	2	1	
	6-months	24 (67%)	-	-	
AQoL	Baseline	36 (100%)	-	-	
	3-months	27 (75%)	-	-	
	6-months	24 (67%)	-	-	
EQ-5D-5L	Baseline	36 (100%)	-	-	
	3-months	27 (75%)	-	-	
	6-months	24 (67%)	-	-	
VAS (Night)	Baseline	36 (100%)	-	-	
	3-months	27 (75%)	-	-	
	6-months	24 (67%)	-	-	
PSEQ	Baseline	36 (100%)	-	-	
	3-months	27 (75%)	1	1	
	6-months	24 (67%)	-	-	
SOPA	Baseline	36 (100%)	-	-	
	3-months	27 (75%)	2	0	
	6-months	24 (67%)	3	1	
TSK-13	Baseline	36 (100%)	-	-	
	3-months	27 (75%)	3	0	Wording unclear (Q5-6)
	6-months	24 (67%)	3	0	Wording unclear (Q5)

Footnote: Final missing items = number of items remaining missing after attempts to clarify with participants.

Abbreviations: AQoL - Assessment of Quality-of-Life (relationships and self-worth domains only; HADS - Hospital Anxiety and Depression Scale (Anxiety and depression scores combined); HADS-Anxiety and depression scores combined;
NDI – Neck disability index; PSEQ – Pain self-efficacy questionnaire; PSFS – Patient specific functional scale; SCI – Sleep condition indicator; SOPA – Survey of Pain Attitudes Questionnaire (single item version); TSK - Tampa Scale of Kinesiophobia; VAS – Visual analogue scale

Price J, *et al. BMJ Open* 2026; 16:e120571. doi: 10.1136/bmjopen-2026-120571
