# Supplementary material for: Co-developed neck exercise (EPIC-Neck) versus usual exercise care for people with chronic non-specific neck pain: a UK multicentre randomised feasibility trial
Source: BMJ Open. 2026 Oct 20;16(10):e120571. doi: 10.1136/bmjopen-2026-120571 (PMC13711528; doi:10.1136/bmjopen-2026-120571)
Supplement: Supplementary file 4 [file bmjopen-16-10-s004.pdf]

# SUPPLEMENTARY FILE 4 – GRIPP2-SF

## 1. Aim of PPIE

The aim of patient and public involvement and engagement (PPIE) was to ensure that the EPIC-Neck feasibility trial, including the intervention and embedded process evaluation, was acceptable, relevant, inclusive, and practical for patients with chronic neck pain. PPIE was used to inform intervention development, trial design, participant-facing materials, recruitment and retention strategies, outcome selection, inclusivity processes, interpretation of findings, and dissemination planning. PPIE also aimed to ensure that the study reflected what mattered to patients, including the acceptability of the intervention, the burden of participation, and how findings should be communicated to patient and public audiences.

## 2. Methods of PPIE

### Recruitment and composition

Participants for the PPIE group were recruited through clinic advertisements, social media, community signposting, and word-of-mouth. This helped achieve diversity in age, gender, ethnic background, and activity levels. We made targeted efforts to reach South Asian communities to reflect the population of Birmingham. All contributors were compensated for their time, and travel and parking costs were covered.

### Format and facilitation

Workshops were held in person and facilitated by the lead author (JP), supported by clinicians, academics, and researchers experienced in PPIE and focus-group facilitation. Sessions used participatory activities (e.g., walking brainstorms, dot voting, round-robin discussions, practical tasks). Each workshop began with a brief recap to help maintain continuity for people who could not attend every session.

### Ensuring meaningful involvement

Facilitators ensured all contributors, including quieter members, had space to speak. PPIE involvement spanned the whole project: multiple workshops during intervention development, one workshop before the trial began, ongoing oversight during the study, and a final workshop focused on interpretation and dissemination. Two PPIE contributors sat on the Study Management Group.

## 3. Results of PPIE Contribution

### Intervention development

- PPIE contributions to intervention development are reported in the intervention development paper.

### Study design

- Highlighted the need to describe “usual exercise care”.

### Participant communication and recruitment

- Co-developed the Participant Information Leaflets (PILs) and invitation materials (wording, layout, accessibility, cultural relevance).
- Suggested video-based PILs in English and Urdu, rather than written translations, to reduce literacy barriers.



Price J, et al. BMJ Open 2026; 16:e120571. doi: 10.1136/bmjopen-2026-120571

- Provided suggestions to improve recruitment processes when initial uptake was low, including the use of NHS NoReply SMS messages as an additional route to support participant invitation and engagement.
- Advised on ethnicity categories, recommending detailed categorisation aligned with UK census format.

### Measures and data collection

- Reviewed and refined questionnaires:
    - added open-text boxes,
    - reduced length,
- Aiding in the selection of an outcome measure to assess relationship impact
- Reviewed interview topic guides: confirmed the length was appropriate and helped refine question wording, particularly around eliciting participants' understanding of the exercise programme.
- Suggested removing the domains of ethicality and burden from quantitative questionnaire as ethicality didn't feel relevant, while burden had crossover with opportunity costs
- Discussed acceptability of audio-recording physiotherapy appointments; the group felt this was acceptable if clearly explained as recording the physiotherapist, not the patient.

### Study oversight and participant engagement

- Contributed to participant newsletters (study progress updates and reminders), which supported questionnaire return.
- Provided feedback at interim stages and helped identify areas requiring modification during the study.

### Dissemination

- Contributed to the development of dissemination principles for patient and public audiences (PUCA framework).

## 4. Discussion and conclusions

PPIE input directly led to:

- more acceptable and accessible study materials (PILs, questionnaires, topic guides, video PILs);
- more culturally detailed demographic categorisation;
- improved recruitment strategy and participant retention;
- practical guidance for future dissemination approaches.

A summary of PPIE contributor characteristics is shown in the table below.

| | | N | % |
|---|---|---|---|
| Age | | | |
| | 30-39 | 7 | 41% |
| | 40-49 | 4 | 24% |
| | 50-59 | 1 | 6% |
| | 60-69 | 4 | 24% |
| | 70+ | 1 | 6% |

| | | | |
|---|---|---|---|
| Gender | | | |
| | Female | 10 | 59% |
| | Male | 7 | 41% |
| Ethnic Background | | | |
| | White British | 8 | 47% |
| | White Other | 1 | 6% |
| | Asian Pakistani | 3 | 18% |
| | Asian Indian | 3 | 18% |
| | Black African | 1 | 6% |
| | Black Caribbean | 1 | 6% |
| Index of Multiple Deprivation Decile | | | |
| | 1 (most deprived) | 5 | 29% |
| | 2 | 2 | 12% |
| | 3 | 2 | 12% |
| | 4 | 3 | 18% |
| | 5 | - | - |
| | 6 | 1 | 6% |
| | 7 | - | - |
| | 8 | - | - |
| | 9 | 3 | 18% |
| | 10 (least deprived) | 1 | 6% |
| Physical Activity Levels | | | |
| | Inactive | 6 | 40% |
| | Moderately Inactive | 2 | 13% |
| | Moderately Active | 4 | 27% |
| | Active | 3 | 20% |

## 5. Reflections / Critical Perspectives

PPIE involvement was extremely valuable and enhanced the acceptability, relevance, and quality of the feasibility trial. However,

- The group were highly engaged and motivated; their experiences may not fully represent patients who struggle with engagement
- Views within the group were sometimes conflicting, requiring careful negotiation and prioritisation.
- Enthusiasm occasionally led to a large number of suggestions, and facilitators needed to balance ambition with methodological and resource constraints.

Overall, PPIE made a substantial contribution to shaping a more patient-centred evaluation and improving the design, conduct, interpretation, and dissemination of the study.



Price J, *et al. BMJ Open* 2026; 16:e120571. doi: 10.1136/bmjopen-2026-120571
