# Supplementary material for: Co-developed neck exercise (EPIC-Neck) versus usual exercise care for people with chronic non-specific neck pain: a UK multicentre randomised feasibility trial
Source: BMJ Open. 2026 Oct 20;16(10):e120571. doi: 10.1136/bmjopen-2026-120571 (PMC13711528; doi:10.1136/bmjopen-2026-120571)
Supplement: Supplementary file 5 [file bmjopen-16-10-s005.pdf]

BMJ Publishing Group Limited (BMJ) disclaims all liability and responsibility arising from any reliance placed on this supplemental material which has been supplied by the author(s)

SUPPLEMENTARY FILE 5 – ACCEPTABILITY DATA

EPIC-Neck participant acceptability questionnaire findings at 3 and 6 months

	3-months			6-months		
**Domain**	**n**	**mean**	**SD**	**n**	**mean**	**SD**
Affective attitude	19	4.00	0.82	18	4.06	0.80
Intervention coherence	19	4.16	0.76	18	4.11	1.08
Opportunity costs	19	3.26	1.28	18	3.33	1.03
Perceived effectiveness	19	3.95	1.03	18	4.06	1.21
Self-efficacy	19	3.63	1.00	18	4.09	0.90
**Overall**	**19**	**3.80**	**1.02**	**18**	**3.93**	**1.04**

Note: Overall acceptability was calculated as the mean across the five questionnaire domains.

Abbreviations: SD – Standard deviation

Price J, *et al. BMJ Open* 2026; 16:e120571. doi: 10.1136/bmjopen-2026-120571

Acceptability framework matrix

Physiotherapist

**Physiotherapist** years treating neck pain	**A: Affective Attitude**	**B: Burden**	**C: Ethicality**	**D: Intervention Coherence**	**E: Opportunity Costs**	**F: Perceived Effectiveness**	**G: Self-Efficacy**
**1: PT1 - 6-10 years' experience**	**Mixed:**<br><br>Initially surprised ("wow") which improved overtime	**Moderate:**<br><br>Time-consuming initially - but improved with practice<br><br>High cognitive effort due to restructure and/or change practice – but improved over time<br><br>Low cognitive effort with some elements (achievable challenge, relationship discussions, sleep tailoring)	**Silence:**	**Partial:**<br><br>Overall: Initially low coherence due to differences from usual practice; improved over time but still partial.<br><br>Coherence better for components that are already part of routine practice<br><br>Conceptual: Understands framework, goal setting, flare-up concepts, and exercise prescription process; unclear on pain self-efficacy and fear assessment and treatment, mental well-being tailoring, and relationship impact. Recognises importance of problem-solving but unsure how to apply. Understands framework facilitates individualisation<br><br>Procedural: Improved skill in exercise selection and scapular work; struggles with motor control assessment/progression and using workbook/app. History-taking aligns with routine practice.<br><br>Other: Structure and patient resources supported understanding and delivery; adoption into practice because it makes conceptual sense; time to deliver reduced as understanding improved.	**Silence:**	**Mixed:**<br><br>Overall: Thinks EPIC-Neck is effective; helpful for complex patients.<br><br>Effective components: Structured approach; individualised goals and exercise prescription; communication strategies; collaborative problem-solving.<br><br>Less effective: Patient resources not well engaged with (potential value but current form ineffective).<br><br>Other: Adopted framework elements into routine practice; feels approach works for non-CNSNP patients.	**Mixed:**<br><br>Initially low confidence which improved with practice, feedback and discussion with peers<br><br>Confidence still not as high as should be<br><br>Low confidence with exercise prescription for fear & pain self-efficacy exercise objectives, progressing deep neck flexor exercises, when objective findings conflicted subjective<br><br>More confident with components similar to usual practice and discussing relationship impact<br><br>Confidence improved with repeated exposure and by integrating additional or more detailed components into existing practice routines.

Price J, *et al. BMJ Open* 2026; 16:e120571. doi: 10.1136/bmjopen-2026-120571

**Physiotherapist** years treating neck pain	**A: Affective Attitude**	**B: Burden**	**C: Ethicality**	**D: Intervention Coherence**	**E: Opportunity Costs**	**F: Perceived Effectiveness**	**G: Self-Efficacy**
**4: PT2 - 11-20 years' experience**	**Positive:**<br><br>Liked the structure	**Moderate:**<br><br>Time-consuming to complete additional tasks (setting up app) and communication strategies - but felt would improve with better understanding and skill<br><br>High cognitive effort due to volume of intervention, restructure and/or change practice (e.g., exercise tailoring for non-pain disability, goal setting) - but improved over time<br><br>High emotional effort (stressful) – but improved over time<br><br>Forgetfulness reduced over time	**Silence:**	**Partial:**<br><br>Overall: Initially uncertain but realised similarities to routine practice; understanding improved with exposure.<br><br>Coherence better for components that are already part of routine practice<br><br>Conceptual: Understands framework and role of non-pain disability outcomes; misunderstood education element in workbook; unclear on goal setting and problem-solving concepts.<br><br>Procedural: Delivery improved over time but still forgets elements; lacks skill in goal setting and problem-solving; struggled with app setup. understands how to deliver pain/disability components better than psychosocial aspects.<br><br>Other: Wanted feedback to support learning; Would forget elements due to lack of understanding/engraining part of routine practice.	**Low:**<br><br>Deferred note-writing to prioritise patient communication; improved with practice.	**Mixed:**<br><br>Overall: Thinks EPIC-Neck is effective.<br><br>Effective components: Structure for exercise prescription; patient resources (motivation/accountability); communication strategies; discussing benefits.<br><br>Less effective: App—patients prefer paper resources.<br><br>Other: Wants more education elements; integrated communication strategies and broader psychosocial focus (mental well-being, sleep, relationships) into routine practice.	**Mixed:**<br><br>Initially low confidence which improved with practice<br><br>Low confidence with goal setting
**5: PT3 - 0-2 years' experience**	**Positive:**<br><br>Enjoyed behaviour change techniques (action planning, communication techniques), prog/regress discussion. Found it useful and enjoyable	**High:**<br><br>Time-consuming to complete additional tasks (setting up app)<br><br>High cognitive effort due to depth of components, restructure and/or change practice - but improved over time<br><br>High cognitive effort when patient was complex or language barriers<br><br>High cognitive burden due to increased self-reflection<br><br>High emotional effort (anxiety, felt tiring) – but	**Mixed:**<br><br>Aligns with values of individualised, feasible exercise prescription. 'Achievable challenge' concept supports patient-centred care and accessibility.<br><br>Values addressing mental well-being in patient care, especially for chronic pain, and sees EPIC-Neck as supporting this focus despite limited familiarity.<br><br>Expressed a values conflict: sometimes usual practice prioritises physiotherapist-led decisions, whereas	**Partial:**<br><br>Overall: Understanding improved but remains incomplete; finds intervention different from usual care.<br><br>Coherence better for components that are already part of routine practice<br><br>Conceptual: Understands engagement, achievable challenge, and some psychosocial concepts (sleep, pain disability); lacks clarity on mental well-being, relationship impact, and tailoring goals. Lacks understanding of how some	**Silence:**	**Mixed:**<br><br>Overall: Thinks EPIC-Neck is effective and improves quality of care.<br><br>Effective components: Structured exercise prescription; scapular work; achievable challenge (engagement, self-monitoring, dosing); communication strategies; patient resources; mental well-being evaluation; flare-up discussions; behaviour change components.<br><br>Less effective: Relationship/social support discussions; framework not	**Mixed:**<br><br>Initially low confidence which improved with practice<br><br>Confidence still not as high as should be<br><br>Felt confidence with exercise prescription improved generally, and using the biopsychosocial exercise prescription framework to individualise exercise<br><br>Low confidence with motor control examination and exercise particular craniocervical flexion, exercise for mental wellbeing and relationship impact.

**Physiotherapist** years treating neck pain	**A: Affective Attitude**	**B: Burden**	**C: Ethicality**	**D: Intervention Coherence**	**E: Opportunity Costs**	**F: Perceived Effectiveness**	**G: Self-Efficacy**
		improved over time<br><br>Cognitive effort with delivery varied depending on emotional/cognitive capacity baseline for the day<br><br>Modified delivery to reduce cognitive effort (avoided in depth discussion, steered patient toward responses that reduce cognitive effort)	EPIC-Neck promotes collaboration and patient autonomy, which challenges existing habits.	exercises work<br><br>Procedural: Better with exercise selection. Better at delivering motor control, scapular, and resistance exercises; struggles with goal setting and tailoring exercises to goals; low skill with biofeedback unit.<br><br>Other: Believes more exposure would improve coherence; understands importance of individualisation but finds applying framework challenging / doesn't understand how to apply framework for low-complexity or resistant patients.		suitable for all patients (e.g., those seeking quick pain relief or who didn't understand EPIC-Neck); goal setting sometimes unnecessary or obstructive.<br><br>Other: Selectively modifies intervention based on patient needs; adopted scapular work and communication strategies into routine practice. Transferable for non-CNSNP patients	More confident with components similar to usual practice (tailoring exercise for pain/disability and sleep)
**2: PT5 - 6-10 years' experience**	**Positive:**<br><br>Liked the patient resources. thinks everything is of value	**High:**<br><br>Time-consuming due to volume, and depth/complexity of some elements (goal setting, problem solving)<br><br>High cognitive effort due to volume of intervention components - but felt it would improve with time and practice<br><br>High cognitive effort with collaborative components if patients were resistant or didn't understand (problem solving, goal setting)<br><br>Cognitive effort with delivery varied depending on emotional/cognitive capacity baseline for the day	**Mixed:**<br><br>Strongly values Biopsychosocial approach<br><br>Personal discomfort and scepticism toward goal setting (conflict with intervention emphasis).	**Partial:**<br><br>Overall: Low understanding due to lack of practice;<br><br>Conceptual: Understands framework and BPS principles; grasps prioritisation and individualisation; partial understanding of goal setting and problem-solving concepts.<br><br>Procedural: Delivery challenging due to multiple components; better with elements aligned to usual practice; struggles with skill in goal setting and problem-solving.<br><br>Other: Recognises conceptual logic behind tailoring; coherence supported by structured framework. understanding how to deliver challenging due to multiple components / volume	**Moderate:**<br><br>Delivering SMART goals as intended requires more time than perceived value; sacrifices other potential activities.	**Mixed:**<br><br>Overall: Thinks EPIC-Neck is effective.<br><br>Effective components: Dedicating time to check understanding and behaviours; communication strategies.<br><br>Less effective: Workbook (low engagement); effectiveness depends on tailoring to patient factors; goal setting not always critical.<br><br>Other: Emphasises effectiveness requires tailoring for patient needs.	**Not confident:**<br><br>Low confidence due to a lack of exposure<br><br>Low confidence with components that were new or different to usual practice

Price J, *et al. BMJ Open* 2026; 16:e120571. doi: 10.1136/bmjopen-2026-120571

**Physiotherapist** years treating neck pain	**A: Affective Attitude**	**B: Burden**	**C: Ethicality**	**D: Intervention Coherence**	**E: Opportunity Costs**	**F: Perceived Effectiveness**	**G: Self-Efficacy**
**3: PT6 - 0-2 years' experience**	**Silence:**	**Moderate:**<br><br>Time-consuming (collaborative working, depth and breadth of content)<br><br>Cognitive effort with delivery varied depending on emotional/cognitive capacity baseline for the day<br><br>Modified delivery to reduce cognitive effort (just provided resources without talking about it, problem solved for patients)	**Aligned:**<br><br>Denies any conflict with beliefs	**Partial:**<br><br>Overall: Understanding and skill improved over time but still variable across components.<br><br>Coherence better for components that are already part of routine practice<br><br>Conceptual: Understands collaborative approach, reducing reliance on physio-led prescription, and exercise tolerance concepts; lacks clarity on general exercise and flare-up rationale; partial understanding of social support.<br><br>Procedural: Better with assessment process and some exercises (e.g., 4-point kneeling); struggles with delivering collaborative/problem-solving approaches and engaging social support; scapular work less familiar.<br><br>Other: Feels coherence would improve further with practice; acknowledges importance of concepts even when skill is lacking.	**Low:**<br><br>Reports being able to implement non-EPIC-Neck interventions	**Mixed:**<br><br>Overall: Thinks EPIC-Neck is effective.<br><br>Effective components: Collaborative approach for exercise; shared decision-making; flare-up discussions; general exercise advice; problem-solving for accountability.<br><br>Less effective: Tailoring required—some patients resist collaboration/problem-solving; lack of engagement with resources; social support discussions not universally effective; skipped elements deemed irrelevant.<br><br>Other: Adopted communication style and some exercises into usual practice; effectiveness depends on delivery skill.	**Mixed:**<br><br>Initially low confidence which improved with practice<br><br>Confidence still not as high as should be<br><br>Low confidence with scapular motor control assessment and exercise prescription

Price J, *et al. BMJ Open* 2026; 16:e120571. doi: 10.1136/bmjopen-2026-120571

**Physiotherapist** years treating neck pain	**A: Affective Attitude**	**B: Burden**	**C: Ethicality**	**D: Intervention Coherence**	**E: Opportunity Costs**	**F: Perceived Effectiveness**	**G: Self-Efficacy**
**6: Cross physiotherapist summary**	**Positive:**<br><br>Most physiotherapists enjoyed delivering EPIC-Neck, valuing the overall approach, structured format, patient resources, and behaviour change techniques. Some described positive feelings such as satisfaction and enjoyment when using action planning or communication strategies. Initial reactions included surprise which improved overtime.	**Moderate:**<br><br>Initially increased cognitive and time burden due to intervention depth, volume and restructuring of usual practice. This generally improved with experience. Burden was greater when emotional or cognitive capacity was reduced or patients were complex or challenging. Some physiotherapists modified delivery to reduce effort (modifying intervention, refraining from app set up).	**Mixed:**<br><br>Two participants provided no ethicality-related data, and one explicitly reported no ethical concerns. Among those who did comment, EPIC-Neck generally aligned with their values, particularly around biopsychosocial, patient-centred, and individualised care, with one participant also valuing its attention to mental wellbeing. Value tensions were noted by two physiotherapists: one expressed low personal value and scepticism toward goal setting, and another felt that the emphasis on shared decision-making challenged their usual physiotherapist-led approach.	**Partial:**<br><br>Initially low across physiotherapists, coherence improved with exposure and practice but remained partial and varied by component and individual. Understanding was strongest for elements already aligned with usual practice, yet overall coherence was still limited at the end of the study.<br><br>Components broadly understood included the conceptual basis of the biopsychosocial exercise prescription framework and its goal of individualised, targeted exercise. Communication strategies were generally understood conceptually, though skill in delivery varied.<br><br>Mixed conceptual understanding was reported for goal setting, problem solving, and social support engagement, with procedural skill for these consistently lacking. Some physiotherapists struggled to apply the framework for individualisation—either omitting elements before assessment (e.g., mental well-being) or applying all components equally to non-complex patients.<br><br>Understanding and skill in exercise selection improved over time, but challenges persisted in evaluating non-pain disability outcomes and prescribing or tailoring exercises accordingly. Motor control exercises were frequently highlighted as difficult to understand and deliver.<br><br>Patient resources were	**Low:**<br><br>Few opportunity costs were noted. One physiotherapist deferred note-writing to prioritise patient communication, and another felt that delivering SMART goals consumed time that could have been spent on components perceived as more valuable. One physiotherapist was able to also implement other interventions.	**Mixed:**<br><br>Overall, physiotherapists perceived EPIC-Neck as effective for improving patient outcomes and engagement.<br><br>Key components viewed as beneficial: A structured approach to exercise prescription, enabling individualised programmes. Communication strategies and collaborative problem-solving. Concepts such as achievable challenge and appropriate exercise dosing.<br><br>Areas of variability: Goal setting, flare-up discussions, and social support conversations were seen as helpful by some but unnecessary or less relevant for others. Patient resources showed mixed engagement: some found them motivating, others did not use them. The app was generally declined by patients, and resources need redesign to be more effective.<br><br>Factors influencing effectiveness: Success depends on clinician skill and tailoring to patient needs. Elements were less effective for patients resistant to collaborative approaches or seeking quick pain relief, and for those unwilling to engage in deeper discussions (e.g., mental well-being, social support). Physiotherapists often adapted the framework selectively based in clinical reasoning.<br><br>Many adopted components (e.g., communication style, exercises, scapular work) into routine practice. Several felt the approach could be	**Mixed:**<br><br>Confidence in delivering EPIC-Neck was generally low at the outset but improved with exposure and practice. By the end of the study, most physiotherapists still felt their confidence was not at the level needed for comfortable routine delivery, citing a need for further practice. Confidence was higher for components similar to usual practice and lower for new or more complex elements. Areas of persistent low confidence included goal setting, motor control assessment and exercise, and exercises targeting pain self-efficacy, fear, mental well-being, and relationship impact.

**Physiotherapist** years treating neck pain	**A: Affective Attitude**	**B: Burden**	**C: Ethicality**	**D: Intervention Coherence**	**E: Opportunity Costs**	**F: Perceived Effectiveness**	**G: Self-Efficacy**
				viewed as helpful for supporting understanding, but physiotherapists noted the need for more time and practice to integrate them effectively; the app was problematic for at least one physiotherapist. The number of components made delivery cognitively demanding. Improved coherence led to selective adoption of certain elements (e.g., scapular work, communication strategies) into routine practice.		applied beyond CNSNP patients.	

Abbreviations: CNSNP = Chronic non-specific neck pain

Price J, *et al. BMJ Open* 2026; 16:e120571. doi: 10.1136/bmjopen-2026-120571

Participant

**Participant**<br>Age, ethnicity, IMD, NDI	**A: Affective Attitude**	**B: Burden**	**C: Ethicality**	**D: Intervention Coherence**	**E: Opportunity Costs**	**F: Perceived Effectiveness**	**G: Self-efficacy**
**1: P01: 60+, White, Low IMD, Mild disability**	**Silence:**	**Silence:**	**Misaligned:**<br>Felt many elements of the programme didn't apply to them due to their lower pain levels, questioning appropriateness. Also expressed belief that the programme should include more traditional physiotherapy.	**Partial:**<br>**PO1:** Knew which exercises to do and how but had limited understanding of dosage. Felt exercises were safe<br>**PO2:** Understands increased repetitions or time to progress<br>**PO3:** Reports if they had a flare-up, they would re-start exercises<br>**PO4:** Not doing general exercise. Wants to restart but no suitable action plan to overcome barriers. Some understanding of the benefits. The patient reports discontinuing exercise because they feel better. There is no mention of weighing the pros and cons of continuing versus stopping exercise	**Silence:**	**Effective:**<br>**Overall:** Felt effective:<br>**Pain/disability/function:** improved pain, postured and ROM. Improved driving<br>**Sleep:** Improved<br>**Mental well-being:** No perceived effectiveness – felt needed to be more general exercise approach for mood changes<br>**Relationship:** No perceived effectiveness	**Mixed:**<br>**PO1:** Denies any confidence issues with exercises<br>**PO2:** Silence<br>**PO3:** Silence<br>**PO4:** Expresses low confidence in starting general exercise
**2: P02: 50s, Black, Low IMD, Mild disability**	**Positive:**<br>Surprised by the benefit. Pleased that exercises were targeted and individualised. Enjoys doing the exercises as they are beneficial. Positive attitudes towards resources	**Low:**<br>**Physical:** Increased burden due to temporary aggravation of symptoms.<br>**Time:** No time burden<br>**Logistical:** able to fit exercises into daily activities.<br>**Cognitive:** Low burden from resources as they were easy and concise to read.	**Aligned:**<br>Valued the targeted exercise approach as it aligned with their preference for non-medication pain management and their personal value of staying active. Felt it supported their identity as an active person and helped break up sedentary time.	**Partial:**<br>**PO1:** Generally demonstrated understanding of most exercises, although technique was challenging for one exercise. Showed awareness of dosage. Describes a limited understanding of some of the bio and psycho benefits. No social benefits. Understands individualisation concept. Low understanding regarding safety.<br>**PO2:** Understands increased repetitions or time to progress. Understands concept of progressing exercise to make achievable challenge<br>**PO3:** Understanding is inconsistent. The participant acknowledges having an exercise programme for flare-ups but says they would "just stop" exercise during a flare-up, contrary to protocol.<br>**PO4:** The patient reports	**Silence:**	**Effective:**<br>**Overall:** Overall effective. Initially sceptical but experienced benefits<br>**Pain/disability/function:** Improved pain. No changes strength or ROM. Improved confidence and reduced fear.<br>**Sleep:** Improved<br>**Mental well-being:** No benefits, but this was not a concern<br>**Relationship:** No benefits, but this was not a concern	**Mixed:**<br>**PO1:** Some reduced confidence with exercises that cause increases in pain. Concerned that exercises may make symptoms worse.<br>**PO2:** Low confidence progressing. Fear of causing damage / making pain worse<br>**PO3:** Silence<br>**PO4:** Expresses low confidence with general exercise for fear of causing damage/worsening pain. Some concern over body building tolerance to exercise long term.

Price J, et al. BMJ Open 2026; 16:e120571. doi: 10.1136/bmjopen-2026-120571

**Participant** Age, ethnicity, IMD, NDI	**A: Affective Attitude**	**B: Burden**	**C: Ethicality**	**D: Intervention Coherence**	**E: Opportunity Costs**	**F: Perceived Effectiveness**	**G: Self-efficacy**
				planning to continue with exercise but no mention of weighing the pros and cons of continuing versus stopping exercise			
**3: P03: 30s, White, Low IMD, Moderate disability**	**Mixed:** While not performing general exercise (PO4) yet, they describe negative feelings about experiences starting general exercise (hate it at first, hurts, embarrassing) which they acknowledge later become positive	**Low:** **Time:** Low burden; exercises done on autopilot during other activities. **Logistical:** able to integrate exercises into daily activities **Cognitive:** Some effort to remember exercises. **Other Notes:** Felt overall exercises were manageable.	**Silence:**	**Partial:** **PO1:** Reported a good understanding of how to do their exercises. No discussion around dosage. Felt exercises were safe. **PO2:** Understands to do less to regress. Didn't know how to make harder **PO3:** The individual understands how to manage flare-ups and mentions they would resume exercises if a flare-up occurred. **PO4:** Not doing general exercise. Wants to restart but no suitable action plan to overcome barriers. The patient reports planning to continue with exercise but accepts they will eventually stop. No mention of weighing the pros and cons of continuing versus stopping exercise	**Silence:**	**Effective:** **Overall:** Felt effective **Pain/disability/function:** Gave short term relief. Didn't help cricked neck. Reduces tensions and tenseness. Improved self-efficacy in neck **Sleep:** Improved but appears unrelated to exercise **Mental well-being:** Improved but appears unrelated to exercise **Relationship:** Improved but appears unrelated to exercise	**Mixed:** **PO1:** Denies any confidence issues with exercises **PO2:** Silence **PO3:** Expresses confidence in managing flare-ups **PO4:** Expresses low confidence in starting general exercise. High confidence to continue with exercise
**4: P04: 50s, Asian, Low IMD, Moderate disability**	**Positive:** Felt exercises were good and overall experience was positive Liked they didn't require changing clothes or getting hot and sweaty and needing a shower Positive attitudes towards resources	**Moderate:** **Time:** Exercises integrated into sitting/daily activities were low burden **Logistical:** Able to integrate some exercises in daily activities. Those requiring space or floor setup were more burdensome and sometimes skipped **Physical:** Exercises interfering with comorbidities increased burden and sometimes skipped **Cognitive:** Completing exercise diary was burdensome due to frequency needed to match exercise completion. Stopped using the diary. **Other Notes:** Felt amount of exercise was achievable and acceptable.	**Aligned:** Valued the exercise approach as it aligned with their preference for non-medication pain management.	**Partial:** **PO1:** Generally demonstrated understanding of most exercises, although technique was challenging for one exercise (scapular protraction). No discussion around dosage. Low understanding of exercise purpose. Reports understanding the benefits but not describes bio, psycho or social benefits. Felt exercises were safe. **PO2:** Silence **PO3:** Reports if they had a flare-up, they would re-start exercises quickly **PO4:** Not doing general exercise. Wants to restart but no suitable action plan	**Silence:**	**Effective:** **Overall:** Overall effective **Pain/disability/function:** Improved stiffness, range of movement **Sleep:** Some improvement but not as much as desired **Mental well-being:** Improved mental well-being **Relationship:** Improved relationships at home	**Confident:** **PO1:** Denies any confidence issues with exercises **PO2:** Silence **PO3:** Silence **PO4:** Silence

**Participant**<br>Age, ethnicity, IMD, NDI	**A: Affective Attitude**	**B: Burden**	**C: Ethicality**	**D: Intervention Coherence**	**E: Opportunity Costs**	**F: Perceived Effectiveness**	**G: Self-efficacy**
**5: P05: 40s, Asian, Low IMD, Moderate disability**)	**Silence:**	**Moderate:**<br>**Time:** Exercises easy when time available but burden increased when limited.<br>**Logistical:** Space constraints varied throughout study with fluctuating burden. More space = less burden. Resource use burdensome due to misplacement and inability to maintain diary during home moves.<br>**Social:** Opted to do exercise away from family which wasn't always possible	**Mixed:**<br>Participant placed significant value on autonomy—a perspective influenced in part by their educational experiences. At first, they struggled with shared decision-making but eventually embraced it through self-motivation and increased understanding.	**Partial:**<br>**PO1:** Reported a good understanding of how to do their exercises. No understanding of achievable challenge dosage. Low understanding of exercise purpose. Describes a limited understanding of some of the bio benefits. No psycho, social benefits. Conflicting understanding of individualisation concept. Low understanding regarding safety.<br>**PO2:** Didn't understand why they needed to progress. Didn't understand it was safe to do.<br>**PO3:** Describes seeking GP support as first line strategy and then continuation with exercise<br>**PO4:** Not doing general exercise. Wants to restart but no suitable action plan to overcome barriers. Unclear understanding of what to do with exercise. No mention of weighing the pros and cons of continuing versus stopping exercise	**High:**<br>Busy schedule and need to rest outweighs priority of exercise, so some exercise session missed or skipped	**Effective:**<br>**Overall:** Overall effective but took time<br>**Pain/disability/function:** reduced medication/ not using medication, reduced pain. No changes in strength. Improved headaches. Improved confidence and reduced fear. Improved range of movement<br>**Sleep:** Improved but appears unrelated to exercise<br>**Mental well-being:** Improved<br>**Relationship:** Improved	**Mixed:**<br>**PO1:** Some reduced confidence with exercises that cause increases in pain. Concerned that exercises may make symptoms worse. Improved over time. Low confidence with trying new exercises not prescribed by physio.<br>**PO2:** Low confidence progressing. Fear of causing damage / making pain worse<br>**PO3:** Silence<br>**PO4:** Expresses low confidence in starting general exercise. Fear of causing damage / worsening pain.
**6: P06: 60+, Black, Low IMD, No disability**	**Silence:**	**High:**<br>**Time:** Exercises easy when time available but burden increased when limited.<br>**Logistical:** Reported high burden of integrating exercises into a routine or alongside daily activities as it felt "regimented" so didn't do it. Improved when utilising visual prompts to do exercise rather than a time dictated routine. Also avoided general exercises as again didn't want a routine or "regimented" way of life. Limited space to store resources for easy access and use.<br>**Physical:** Temporary increases in pain which was modified with changes in dosage.	**Mixed:**<br><br>Aligns with their general value of health and exercise but misaligns with their preference for autonomy and non-regimented routines.	**Partial:**<br>**PO1:** Generally demonstrated understanding of most exercises, although technique was challenging for one exercise (banded cervical retraction). No discussion around dosage. Low understanding of exercise purpose. Describes a limited understanding of some of the bio benefits. No psycho, social benefits. Initially low understanding of safety which improved.<br>**PO2:** Understood increasing reps. Unclear understanding of why. Didn't understand it was safe to do.<br>**PO3:** Unable to recall instructions; reports both stopping and continuing exercise without changes.	**Silence:**	**Effective:**<br>**Overall:** Overall effective but took time<br>**Pain/disability/function:** Reduced pain, improved movement, improved strength<br>**Sleep:** Improved<br>**Mental well-being:** No benefits, but this was not a concern<br>**Relationship:** Improved	**Mixed:**<br>**PO1:** Some reduced confidence with more complex exercises. Some concern with exercises that cause increases in pain which improved over time<br>**PO2:** Silence<br>**PO3:** Expresses low confidence in managing flare-ups<br>**PO4:** Expresses low confidence in starting general exercise. Fear of causing damage / worsening pain. Expresses confidence with continuing exercise

Price J, *et al. BMJ Open* 2026; 16:e120571. doi: 10.1136/bmjopen-2026-120571

**Participant** Age, ethnicity, IMD, NDI	**A: Affective Attitude**	**B: Burden**	**C: Ethicality**	**D: Intervention Coherence**	**E: Opportunity Costs**	**F: Perceived Effectiveness**	**G: Self-efficacy**
		**Cognitive:** High burden from resources as felt too much information in one go and felt physio input was sufficient.		**PO4:** Reports not wanting to start general exercise. The patient reports planning to continue with exercise but no mention of weighing the pros and cons of continuing versus stopping exercise			
**7: P07: 20s, Asian, Low IMD, Moderate disability**	**Silence:**	**Silence:**	**Silence:**	**Partial:**<br>**PO1:** Reported a good understanding of how to do their exercises. No discussion around dosage.<br>**PO2:** Silence<br>**PO3:** Describes seeking GP support as first line strategy and then continuation with exercise<br>**PO4:** The patient reports planning to continue with exercise but no mention of weighing the pros and cons of continuing versus stopping exercise	**Silence:**	**Effective:**<br>**Overall:** Overall effective. Initially sceptical but experienced benefits<br>**Pain/disability/function:** Reduced pain, improved posture, less cracking. Less fear and improved confidence.<br>**Sleep:** Improved but appears unrelated to exercise<br>**Mental well-being:** Improved<br>**Relationship:** Not discussed	**Confident:**<br>**PO1:** Silence<br>**PO2:** Silence<br>**PO3:** Silence<br>**PO4:** Expresses confidence in continuing with exercise
**8: P08: 50s, Mixed, Mid IMD, Mild disability**	**Mixed:**<br>Didn't enjoy how the exercises initially made them feel and extra strain it was placing on other joints<br>Positive attitudes towards resources	**Moderate:**<br>**Time:** Time consuming especially with increased repetitions<br>**Physical:** Interfered with comorbidities requiring change of exercises.<br>**Logistical:** Exercises that were able to be integrated into activities of daily living were performed with low burden<br>**Social:** Some effort required to maintain privacy at home and overcome initial embarrassment when exercising in public.<br>**Other Notes:** Overall reported as tolerable	**Mixed:**<br>Aligns with their value for individualised treatment that adapts to their needs. Conflict arose because the time-consuming nature of some exercises clashed with their lifestyle.	**Partial:**<br>**PO1:** Reported a good understanding of how to do their exercises. Showed awareness of dosage. Low understanding of exercise purpose. Describes a good understanding of some of the bio benefits. No psycho, social benefits.<br>**PO2:** Understood increasing reps/ decreasing reps, holding it for longer. Understood concept of progress to make it an achievable challenge. Build up strength in muscles<br>**PO3:** Reports lowering dosage, then increasing it as symptoms improved, and using a workbook to guide decisions.<br>**PO4:** Already going to the gym anyway. Some understanding of the benefits. The patient reports planning to continue with exercise. Some understanding as to benefits but no mention of weighing the pros and cons of	**High:**<br>Work and long commute created substantial time constraints; participant prioritised personal care and rest over exercises, leading to skipped sessions.	**Effective:**<br>**Overall:** Overall effective but took time<br>**Pain/disability/function:** Improved pain, strength, ability to work and sit, improved concentration<br>**Sleep:** Improved<br>**Mental well-being:** Improved<br>**Relationship:** Improved	**Mixed:**<br>**PO1:** Initially some low confidence doing exercise in public, but it improved. No concerns regarding safety. Low confidence with trying new exercises not prescribed by physio.<br>**PO2:** Silence<br>**PO3:** Expresses confidence in managing flare-ups<br>**PO4:** Silence

Price J, et al. BMJ Open 2026; 16:e120571. doi: 10.1136/bmjopen-2026-120571

**Participant** Age, ethnicity, IMD, NDI	**A: Affective Attitude**	**B: Burden**	**C: Ethicality**	**D: Intervention Coherence**	**E: Opportunity Costs**	**F: Perceived Effectiveness**	**G: Self-efficacy**
				continuing versus stopping exercise			
**9: P09: 30s, White, Mid IMD, Moderate disability**	**Positive:** Liked the flexibility and individualised nature of the exercise	**Moderate:** **Logistical:** Low burden as could be applied flexibly and most integrated into activities of daily living or routines. Increase burden with those unable to integrated into activities of daily living. Often skipped those that were not easily integrated **Cognitive:** Exercises requiring more concentration or with complex techniques required more effort and often skipped. **Social:** Reported no social burden and felt comfortable doing exercise in the office	**Mixed:** Aligns with their value for flexibility and shared decision making and use of goal setting. Valued the integration of social support but things we can do more. However, the use of patient resources misaligns with their dislike for written and video-based resources, which are part of the programme.	**Partial:** **PO1:** Generally demonstrated understanding of most exercises, although technique was challenging for one exercise with more complex technique. No discussion around dosage. Understood exercises were individualised to goals and functional requirements. Low understanding of safety. Understanding of bio and psycho and social benefits. **PO2:** No understanding. Didn't understand why they needed to progress. Didn't understand it was safe to do. **PO3:** Silence **PO4:** The patient reports planning to continue with exercise. Reports continuation will act as prevention. Limited understanding of benefits	**Low:** Able to integrate exercise into daily activities without trade-offs	**Effective:** **Overall:** Overall effective **Pain/disability/function:** Improved confidence and catastrophic thinking, **Sleep:** Improved **Mental well-being:** Improved via a mechanism of reduced pain and catastrophic thinking / fear **Relationship:** No benefits, but this was not a concern	**Mixed:** **PO1:** No confidence issues doing exercises in office. Low confidence with trying new exercises not prescribed by physio. Concerned that exercises may make symptoms worse / cause damage. **PO2:** Low confidence progressing. Fear of causing damage / making pain worse **PO3:** Silence **PO4:** Silence
**10: P10: 30s, Other, Low IMD, Mild disability**	**Positive:** Surprised by, and happy with the benefit. Liked the individualised nature	**High:** **Time:** Time consuming especially with increased repetitions. Improved with increased resistance/ reduced repetitions and splitting exercises up **Logistical:** Initially integrating exercise into a routine was effortful but improved with the use of visual prompts. Exercises not able to integrate into activities of daily living or requiring special space were more effortful and skipped. **Physical:** Fatigue often prevented exercise completion. **Cognitive:** Effort required to remember to do exercises improved with use of visual prompts **Other notes:** over time reports most exercises became manageable, but	**Silence:**	**Partial:** **PO1:** Reported a good understanding of how to do their exercises but struggled more with more complex exercises (4-point kneeling, craniocervical flexion supine). Showed awareness of dosage. Understood exercises were individualised. Describes a good understanding of some of the bio benefits. No psycho, social benefits. **PO2:** Understood increasing reps. Increasing resistance. Understood concept of progress to make it an achievable challenge. **PO3:** Describes using medication and then restarting exercises **PO4:** Not doing general exercise but wants to. Has some form of an action plan but not yet started. Some understanding of the benefits. The patient reports	**High:** Participant skipped exercises when faced with work or time constraints, indicating competing priorities override intervention engagement.	**Effective:** **Overall:** Overall effective. Initially sceptical but experienced benefits. Took time for benefits **Pain/disability/function:** Reduced pain, improved movement **Sleep:** No benefits, but this was not a concern **Mental well-being:** Improved **Relationship:** Improved	**Confident:** **PO1:** Silence **PO2:** Expresses high confidence in progressing exercise **PO3:** Silence **PO4:** Expresses confidence in general exercise.

Price J, *et al. BMJ Open* 2026; 16:e120571. doi: 10.1136/bmjopen-2026-120571

**Participant**<br>Age, ethnicity, IMD, NDI	**A: Affective Attitude**	**B: Burden**	**C: Ethicality**	**D: Intervention Coherence**	**E: Opportunity Costs**	**F: Perceived Effectiveness**	**G: Self-efficacy**
		time-consuming ones were eventually discontinued.		planning to continue with some exercise. Limited understanding of benefits and pros/cons.			
**12: P11: 40s, White, Low IMD, Mild disability**	**Silence:**	**Low:**<br>**Time:** Lack of time on travel days but overall minimal<br>**Logistical:** Integrating exercises into activities of daily living or into existing routines reduced effort. Some space constraints managed by exercising after family went to bed.<br>**Social:** Minor social consideration (banter from son) but not a barrier.<br>**Other notes:** overall felt little burden	**Silence:**	**Partial:**<br>**PO1:** Reported a good understanding of how to do their exercises. Showed awareness of dosage. No understanding of exercise purpose. Reports not understanding the benefits. Initially low understanding of safety which improved.<br>**PO2:** Understood increasing reps. Unclear understanding of why. Didn't understand it was safe to do.<br>**PO3:** Describes continuing with exercise and also using workbook to guide decision making<br>**PO4:** Not doing general exercise. Wants to restart but no suitable action plan to overcome barriers. Some understanding of the benefits. The patient reports planning to continue with some exercise. Limited understanding of benefits and pros/cons.	**High:**<br>Long-distance travel for child's activities created substantial time constraints; participant prioritised family commitments over exercises, leading to skipped sessions.	**Effective:**<br>**Overall:** Overall effective. Initially sceptical but experienced benefits. Took time for benefits<br>**Pain/disability/function:** Reduced pain, improved movement, carrying shopping and reaching<br>**Sleep**: Improved<br>**Mental well-being:** Improved<br>**Relationship:** No benefits, but this was not a concern	**Confident:**<br>**PO1:** Initially worried exercises would cause damage / make things worse. Improved over time.<br>**PO2:** Silence<br>**PO3:** Expresses confidence managing flare-ups<br>**PO4:** Silence
**11: P12: 80+, White, Low IMD, No disability**	**Mixed:**<br>Happy with the benefits and clarity. Exercises not enjoyable per se but acceptable because of the benefits gained. Satisfaction of using patient resources	**Low:**<br>**Time:** Time consuming especially with increased repetitions or number of exercises. Improved with splitting exercises up<br>**Logistical:** Integrating exercises into activities of daily living or into existing routines reduced effort<br>**Cognitive:** Effort required to remember exercises, dosage and education	**Silence:**	**Partial:**<br>**PO1:** Reported a good understanding of how to do their exercises. Showed awareness of dosage. Low understanding of exercise purpose. Describes a good understanding of some of the bio benefits. No psycho, social benefits.<br>**PO2:** Understood increasing reps.<br>**PO3:** Reports if they had a flare-up, they would re-start exercises<br>**PO4:** Already engaging with general exercise anyway. The patient reports planning to discontinue exercise if they feel better. There is no mention of weighing the pros	**Moderate:**<br>Sacrifices leisure time, but considered trade-off acceptable due to benefits	**Effective:**<br>**Overall:** Overall effective.<br>**Pain/disability/function:** Reduced pain, improved movement<br>**Sleep:** No benefits, but this was not a concern<br>**Mental well-being:** No benefits, but this was not a concern<br>**Relationship:** No benefits, but this was not a concern	**Confident:**<br>**PO1:** Denies any confidence issues with exercises<br>**PO2:** Neutral confidence in progressing<br>**PO3:** Silence<br>**PO4:** Silence

Price J, *et al. BMJ Open* 2026; 16:e120571. doi: 10.1136/bmjopen-2026-120571

**Participant** Age, ethnicity, IMD, NDI	**A: Affective Attitude**	**B: Burden**	**C: Ethicality**	**D: Intervention Coherence**	**E: Opportunity Costs**	**F: Perceived Effectiveness**	**G: Self-efficacy**
				and cons of continuing versus stopping exercise			
**13: Cross-participant summary**	**Mixed:** Among participants, affective attitudes towards the exercise programme were notably absent for many. Several individuals did not express strong emotions regarding their participation. However, for those who did, the primary association was with the tangible benefits they experienced; these positive outcomes tended to outweigh any lack of enjoyment associated with undertaking the exercises. A number of participants reported being surprised by the improvements they observed. There was a general sense of satisfaction with the individualised and targeted approach, as well as appreciation for the patient resources provided. Despite these positive aspects, some participants did express negative feelings. These included concerns about how exercise could make them feel physically, the potential burden on other joints, and negative associations with general exercise based on previous experiences.	**Moderate:** **Cognitive:** Cognitive effort varied among participants. Some had difficulty remembering to do the exercises. More cognitive effort was described for those that required greater concentration or involved more complex techniques, which were often skipped. The mental effort needed for patient resources also differed: some participants found them burdensome due to their length or the frequency with which they had to be completed (such as an exercise diary), while others considered them easy and straightforward to use. **Physical:** Some individuals reported exerting extra effort as a result of heightened neck pain, additional musculoskeletal discomfort, or complications arising from other comorbidities. At times, these exercises were omitted. **Social:** Some reported additional effort to do the exercises away from family members (in private). Some reported initial effort to overcome embarrassment when exercising in public while others felt this did not require additional effort. **Time:** Participants commonly reported on exercise duration. Some found it manageable when integrated with other activities, while others felt it became time-consuming as repetitions increased. Increasing resistance allowed one participant to cut down on repetitions and time. The perceived time burden also varied depending on other commitments.	**Mixed:** Participants valued individualised and tailored exercise, aligning with beliefs about personalisation and non-pharmacological management. Conflicts arose when programme elements clashed with personal or cultural values—such as dislike of routines, and preference for interpersonal support over written or video resources. One participant questioned appropriateness, feeling the programme was designed for people with more severe symptoms than their own.	**Incoherent:** **PO1:** Most participants understood how to do their exercises but had more difficulty with complex or resisted ones. Dosage was discussed less, and while some could describe a plan, it was sometimes unclear. Overall, there was limited understanding of the purpose and benefits of exercise. When people did express some awareness, it focused mainly on biological advantages like pain reduction and increased strength. Understanding of the safety of exercise varied. **PO2:** Most recognized that adding repetitions made the exercise tougher; few remembered other strategies. Responses varied among participants. Some were unclear about the rationale or believed it might be unsafe or potentially cause more harm. Others demonstrated an understanding that the exercise was intended to present an achievable challenge, supporting ongoing physiological improvements. **PO3:** There was inconsistent understanding of using exercise to manage flare-ups. Some participants restarted or adjusted exercise routines, others continued without changes, while some prioritized GP consultations or medication first. **PO4:** Understanding of a general exercise plan and the benefits was mixed. Most participants reported motivation to engage with general exercise, but none were able to describe a suitably tailored action plan.	**Moderate:** There was no evidence of reported trade-offs for many participants while some described minor sacrifices that were outweighed by the perceived benefits. Multiple participants described prioritising other activities over engagement with exercise.	**Effective:** All participants considered their exercise program effective, reporting improvements in pain, disability, and function. Many noticed better sleep, mental well-being, and relationships, though these benefits depended on whether they had existing issues. Some were initially doubtful about the program's effectiveness but observed improvements over time. Participants also noted that it took several weeks before the exercises became truly effective.	**Mixed:** **PO1:** Confidence varied between participants. Most had confidence doing their exercises, but some reported lower confidence where the exercise were more complex, or where there were concerns, they would cause more damage / make their pain worse. For some confidence improved over time. **PO2:** There was a silence of data for many participants. Where data was present there was multiple findings of low confidence citing fear of causing damage or making pain worse. Some participants described high or neutral confidence. **PO3:** There was a silence of data for many participants. Where data was present all participants reported confidence in self-managing flare-ups with exercise. **PO4:** There was a silence of data for many participants. Where data was present all but one participant reported a lack of confidence with engaging in general exercise citing common general exercise barriers or fear of causing damage or making symptoms worse. There were multiple reports of being confidence in continuing with physiotherapist prescribed neck exercise.

**Participant** Age, ethnicity, IMD, NDI	**A: Affective Attitude**	**B: Burden**	**C: Ethicality**	**D: Intervention Coherence**	**E: Opportunity Costs**	**F: Perceived Effectiveness**	**G: Self-efficacy**
		**Logistical:** Participants who successfully incorporated exercise into existing routines or activities of daily living generally reported minimal burden. However, one participant noted that integrating exercise into a routine felt “regimented” and resulted in increased burden. In contrast, exercises that could not be integrated into daily activities, or that required specific space or positioning, were described as more demanding and were sometimes avoided. The need to find additional space for exercise or store patient resources was occasionally mentioned as requiring extra effort. Additionally, some patient resources were reportedly misplaced.<br>Burden was reduced by using visual prompts, splitting exercises and adjusting repetitions/resistance.<br>Despite people reporting burden – they still reported that the exercises were manageable and acceptable.		Some participants were able to report a basic understanding of the benefit of general exercise (improve strength, feel good). There was generally a lack of an understanding or conceptual framework regarding the advantages and disadvantages of continuing with secondary prevention.			

Abbreviations: IMD = Index multiple deprivation; NDI = Neck Disability Index; ROM = Range of movement; PO1 = Performing physiotherapist prescribed exercise; PO2 = Adapting, progressing or regressing exercise independently; PO3 = self-managing flare-ups with exercise; PO4 = engaging in general exercise ± secondary prevention exercise

Price J, *et al. BMJ Open* 2026; 16:e120571. doi: 10.1136/bmjopen-2026-120571

Acceptability narrative description with quotes

Affective attitude

There were mixed attitudes towards EPIC-Neck. Patients' affective attitude towards EPIC-Neck was generally positive at 6 months (mean = 4.06, SD = 0.80, 95% CI 3.66–4.45). A total of 83% (15/18) rated affective attitude as acceptable (scores 4–5).

Interview findings partially complemented questionnaire results. Patients generally expressed positive attitudes towards EPIC-Neck, highlighting satisfaction with the individualised and targeted nature of their exercise programme and appreciation for supporting resources. For many, affective attitudes were neutral or absent, but when present, positive feelings were often linked to tangible benefits and unexpected improvements. Some participants reported negative emotions related to physical discomfort, concerns about burden on other joints, and prior negative experiences with general exercise. Overall, perceived benefits tended to outweigh any lack of enjoyment.

> *"I actually found them to be erm very good at strategically hitting the pain…I was quite surprised at how very simple movements that actually hit that pain and I was surprised that it was exercise that was being used to treat the pain to be honest with you, I really didn't want to have to ingesting pills to fight pain and flare-ups, so the idea of exercising the pain away was really great" P02: 50s, Black, Low IMD, Mild disability*

> *"I didn't particularly enjoy how it made me feel…getting up and down from the floor, I'm getting on a bit and my knees don't work." P08: 50s, Mixed, Mid IMD, Mild disability*

> *"I wouldn't say enjoyable, I'd rather I didn't have to do them, but I realise that there is going to be benefit and so I've done them." P12: 80+, White, Low IMD, No disability*

Physiotherapists had positive attitudes towards EPIC-Neck, valuing the overall structured approach and use of behaviour change techniques. Initial reactions of surprise improved with increased knowledge and confidence in delivery.

> *"So, I think overall it's been a positive experience…I'm a massive fan of the behaviour change stuff." PT3 - 0-2 years' experience*

Overall, there were minor concerns regarding affective attitude; steps should be taken to address patient concerns about physical side effects and attitudes towards general exercise prior to a definitive trial.

Burden

EPIC-Neck results in moderate burden. Patients reported moderate cognitive, physical, social, time and logistical burden as a result of their exercise programme. Cognitive effort varied but was increased for exercises that were more complex. Physical burden was increased due to increases in neck pain or additional discomfort due to comorbidities. Social burden varied with some describing effort to overcome embarrassment when exercising in public while others felt this did not require additional effort. Most patients reported exercise duration as a burden but found it manageable when they were able to integrate exercise into other activities or existing routines. Burden increased when they were unable integrate exercise due to

specific space or positioning requirements, or with increased duration with increasing repetitions. Overall, burden was reduced by strategies such as visual prompts, splitting exercises, and adjusting repetitions or resistance.

> *"The ones in the sitting position…we are sitting around most of the day and it's taking a couple of minutes and just, you know shrugging the shoulders up and down or you know, moving your head around. Those have been the easiest…the one on all fours, that was a little bit um… [pause] tricky…if you've got the space and your mat is always out it's easy but when you have to say okay, let's go upstairs, let's get the mat out, let's put it down, where should I put it? It's like I'll do it later. That's when later never comes in [laugh]" P04: 50s, Asian, Low IMD, Moderate disability*

> *"I started thinking Okay, I can't spend 20 minutes to do the exercises" P10: 30s, Other, Low IMD, Mild disability*

Physiotherapists reported moderate burden delivering EPIC-Neck, driven by intervention depth, volume, and large changes to usual practice, although the burden began to reduce with practice and improved skill. Burden was higher with complex patients, and some physiotherapists modified delivery to reduce effort.

> *"Initially I found it quite difficult purely because I was changing the way that I assess somebody totally. It was a totally different way of thinking and questioning…but after a few attempts it became a bit more fluent and a bit easier to do." PT3 - 0-2 years' experience*

Overall, there were minor concerns regarding burden; steps should be taken to address patient burden related to exercise duration and complex exercises, and physiotherapist burden related to intervention depth and change in clinical practice prior to a definitive trial.

Ethicality

There was mixed alignment between EPIC-Neck and patient and physiotherapists beliefs and values. Patients had mixed views on EPIC-Neck's alignment with personal values and beliefs. Many valued the individualised and tailored approach, which aligned with their preference for personalisation and non-pharmacological management. However, conflicts arose when programme elements clashed with personal or cultural values—such as dislike of structured routines, and preference for interpersonal support over written or video resources. One participant questioned appropriateness, feeling the programme was designed for people with more severe symptoms than their own.

> *"I'm not the person who's like oh I'm in pain and I'll start taking pills, I'd rather do the exercises. I'm not a pill person" P04: 50s, Asian, Low IMD, Moderate disability*

> *"I'm not being funny but its literature. People don't read it now… it's a leaflet …people pick it up and put it to the side …people will take it out of grace…the written stuff personally that doesn't work for me" P09: 30s, White, Mid IMD, Moderate disability*

Physiotherapists had mixed views on EPIC-Neck alignment with personal and professional values. The individualised biopsychosocial patient centred approach of

the EPIC-Neck programme aligned with their beliefs, but misalignment was found for some in relation to goal-setting and shared decision making.

> *"I'm not a goal driven person…there massively time consuming…it does give you structure in the things you need to be doing, but it doesn't help me massively in where I'm going next with patients, therefore it's an incentive to invest the time that it takes to get a goal established with a patient has used up more of my time than I might feel like it's being used elsewhere. I guess it's a degree of being unconvinced by it and the value in it." PT5 - 6-10 years' experience*

Overall, there were minor concerns regarding ethicality; steps should be taken to address patient misalignment with shared decision-making and resource preferences, and physiotherapist misalignment with goal-setting and shared decision-making prior to a definitive trial.

Intervention coherence

EPIC-Neck coherence was generally low. Patients rated their understanding of the programme rationale as acceptable at 6 months (mean = 4.11, SD = 1.08, 95% CI 3.57–4.65). The proportion rating coherence as acceptable was 89% (16/18).

Interview finding conflicted with questionnaire results. Patients demonstrated low intervention coherence overall. While most understood how to perform basic exercises, understanding reduced with complex or resisted exercise. Understanding of the underlying purpose, benefits and safety of exercise was limited, often restricted to biological outcomes such as pain reduction or strength gains. Progression strategies were recalled but restricted to repetition changes. Coherence was particularly weak for using exercise to manage flare-ups (PO3) and for general exercise plans +/- secondary prevention (PO4), where few participants could describe strategies aligned with EPIC-Neck standards.

> *"One exercise that was a bit difficult, was where you've got to sort of push yourself towards the wall, you had to keep your arms stretched and I couldn't do that one…I couldn't get my head around that one, I don't know which part I was missing that I couldn't do it the way he was showing me [laugh]… I did try, I did try but I just couldn't do that push the wall away and keep my arms straight and bring my shoulders together at the back, I couldn't do it…I shouldn't say this, but it was only the one attempt [laugh]" P04: 50s, Asian, Low IMD, Moderate disability*

Physiotherapists demonstrated partial coherence in understanding the purpose of EPIC-Neck and its delivery. Coherence was stronger for components aligned with usual practice but remained incomplete for new or complex elements. While physiotherapists understood the purpose of goal setting, problem-solving, and social support engagement, many lacked the knowledge and skills to apply these in practice. Delivery coherence was particularly low for evaluation and exercise prescription of non-pain disability outcomes and motor control exercises. Although understanding improved over time, gaps persisted; however, many physiotherapists selectively adopted components into routine care when they made conceptual sense.

> *"I think in terms of relationships and trying to get the family involved. Did I give exercises that aim to improve friends and family understanding and aim to*

> *improve social support? No…Maybe I didn't get it. How would an exercise improve a family members or friends understanding of it? I think I would incorporate family support with the relatives there...but may be a misunderstanding?" PT3 - 0-2 years' experience*

Overall, there were major concerns regarding coherence; steps should be taken to address low patient understanding of PO2–PO4 and safety/benefits of exercise, and physiotherapist understanding of delivery with new or complex components prior to a definitive trial.

Opportunity costs

Low to moderate trade-offs were found to engage with EPIC-Neck. Patients reported neutral to low interference with other priorities (mean = 3.33, SD = 1.03, 95% CI 2.82–3.85). The proportion rating opportunity costs as acceptable (≥4) was 56% (10/18).

This was partially complimented by interview findings. Many patients described no significant trade-offs, while others noted minor sacrifices that were outweighed by perceived benefits. However, several patients deprioritised exercise in favour of other activities, leading to omission.

> *"You could say all of them were a burden, couldn't you? If, if it's taking up your leisure time where you could be doing something else, which you would rather do then yes but, I see it as something totally acceptable because I want to get rid of this neck pain." P12: 80+, White, Low IMD, No disability*

> *"I don't really wake up with plenty of time before I have to do things. So typically, when I wake up, there's not much time before I need to be going and, you know, doing this or that, or this or that. So, when I tried to do in the morning, as soon as I woke up, I had to make sure that I wasn't in a rush for something or like I had any, any appointments, any work meetings, or any, you know, things I had to do." P10: 30s, Other, Low IMD, Mild disability*

EPIC-Neck required low trade-offs for physiotherapists. There was largely an absence of trade-offs reported other than one physiotherapist writing notes after appointments to prioritise communication, and another feeling goal setting detracted time from other treatment components perceived as more valuable.

Overall, there were minor concerns regarding opportunity costs; steps should be taken to address patient de-prioritisation of exercise.

Perceived effectiveness

Perceived effectiveness of EPIC-Neck was generally positive. Perceived effectiveness was generally positive (mean = 4.06, SD = 1.21, 95% CI 3.45–4.66), with 83% (15/18) rating perceived effectiveness as acceptable.

All participants considered their exercise program effective, reporting improvements in pain, disability, and function. Many noticed better sleep, mental well-being, and relationships, though these benefits depended on whether they had existing issues. Some were initially doubtful about the program's effectiveness but observed improvements over time. Participants also noted that it took several weeks before the exercises became truly effective.

Price J, *et al. BMJ Open* 2026; 16:e120571. doi: 10.1136/bmjopen-2026-120571

Physiotherapists reported mixed perceived effectiveness. Collectively they felt the overall approach was valuable and would result in more effective and engaging exercises. Specifically, components of communication strategies, collaborative programme solving and achievable challenging exercise dosing were specifically highlighted. Components they perceived to be of low value or effectiveness were goal setting and social support engagement. The patient resources were perceived as being valuable but in their current format had mixed engagement by patients. Physiotherapist felt their skill, and patient presentation modified the effectiveness of the intervention (e.g., less effective for patients resistant to collaborative approaches or seeking quick pain relief, and for those unwilling to engage in deeper discussions around mental well-being and social support). As such physiotherapists selectively adapted the framework based on clinical reasoning. Overall, the approach was viewed as an effective strategy demonstrated by physiotherapists adopting components into their usual care and suggesting the approach is suitable for a wider cohort of chronic musculoskeletal pain patients.

Overall, there were minor concerns regarding perceived effectiveness; steps should be taken to address physiotherapist concerns about goal setting, social support, and patient resources.

Self-efficacy

Mixed confidence was found with EPIC-Neck. Patients' confidence in completing their EPIC-Neck exercises was positive at 6 months (mean = 4.09, SD = 0.90, 95% CI 3.65–4.54). A total of 72% (13/18) rated self-efficacy as acceptable.

This was qualified by interview findings as patient confidence varied across performance objectives. Most participants felt confident performing basic exercises (PO1), but confidence declined for complex or resisted exercises and where fear of harm was present. Progression strategies (PO2) were associated with mixed confidence, with some participants reporting low confidence due to fear of causing damage or worsening pain. Confidence for flare-up management (PO3) was generally high among those who discussed it, while confidence for engaging in general exercise (PO4) was low for most, citing common barriers and fear of harm. Some participants reported confidence improving over time with practice.

Physiotherapists' self-efficacy for delivering the EPIC-Neck programme was mixed. Initially it was low but improved with exposure and practice. Confidence was higher for components that aligned to their routine practice. Components where physiotherapist confidence remained low in delivery were goal setting, motor control assessment and exercise, and exercises targeting pain self-efficacy, fear, mental well-being, and relationship impact.

Overall, there were minor concerns regarding self-efficacy; steps should be taken to address patient confidence with complex exercises, safety, progression strategies, and general exercise; and physiotherapist confidence with new or complex components prior to a definitive trial.

Price J, *et al. BMJ Open* 2026; 16:e120571. doi: 10.1136/bmjopen-2026-120571
