# Supplementary material for: Co-developed neck exercise (EPIC-Neck) versus usual exercise care for people with chronic non-specific neck pain: a UK multicentre randomised feasibility trial
Source: BMJ Open. 2026 Oct 20;16(10):e120571. doi: 10.1136/bmjopen-2026-120571 (PMC13711528; doi:10.1136/bmjopen-2026-120571)
Supplement: Supplementary file 6 [file bmjopen-2026-120571.draft_revisions.pdf]

BMJ Open is committed to open peer review. As part of this commitment we make the peer review history of every article we publish publicly available.

When an article is published we post the peer reviewers' comments and the authors' responses online. We also post the versions of the paper that were used during peer review. These are the versions that the peer review comments apply to.

The versions of the paper that follow are the versions that were submitted during the peer review process. They are not the versions of record or the final published versions. They should not be cited or distributed as the published version of this manuscript.

BMJ Open is an open access journal and the full, final, typeset and author-corrected version of record of the manuscript is available on our site with no access controls, subscription charges or pay-per-view fees (http://bmjopen.bmj.com).

If you have any questions on BMJ Open's open peer review process please email info.bmjopen@bmj.com

BMJ Open

Co-developed neck exercise (EPIC-Neck) versus usual exercise care for people with chronic non-specific neck pain: a multicentre randomised feasibility trial

Journal:	*BMJ Open*
Manuscript ID	bmjopen-2026-120571
Article Type:	Original research
Date Submitted by the Author:	02-Apr-2026
Complete List of Authors:	Price, Jonathan; Birmingham Community Healthcare NHS Foundation Trust, Musculoskeletal Physiotherapy Services; Birmingham Community Healthcare NHS Foundation Trust, Research and Innovation; University of Birmingham College of Life and Environmental Sciences, School of Sport, Exercise and Rehabilitation Sciences<br>Rushton, Alison; Western University, School of Physical Therapy<br>Ives, Natalie; University of Birmingham College of Medical and Dental Sciences, Institute of Applied Health Research<br>Bradbury, Marilyn; Birmingham Community Healthcare NHS Foundation Trust, Research and Innovation; University of Birmingham College of Life and Environmental Sciences, School of Sport, Exercise and Rehabilitation Sciences<br>Parmar, Priti ; Birmingham Community Healthcare NHS Foundation Trust, Research and Innovation<br>Greaves, Colin; University of Birmingham College of Life and Environmental Sciences, School of Sport, Exercise and Rehabilitation Sciences
Keywords:	Exercise, Self-Management, Behavior, Spine < ORTHOPAEDIC & TRAUMA SURGERY

SCHOLARONE MANUSCRIPTS

I, the Submitting Author has the right to grant and does grant on behalf of all authors of the Work (as defined in the below author licence), an exclusive licence and/or a non-exclusive licence for contributions from authors who are: i) UK Crown employees; ii) where BMJ has agreed a CC-BY licence shall apply, and/or iii) in accordance with the terms applicable for US Federal Government officers or employees acting as part of their official duties; on a worldwide, perpetual, irrevocable, royalty-free basis to BMJ Publishing Group Ltd ("BMJ") its licensees and where the relevant Journal is co-owned by BMJ to the co-owners of the Journal, to publish the Work in this journal and any other BMJ products and to exploit all rights, as set out in our licence.

The Submitting Author accepts and understands that any supply made under these terms is made by BMJ to the Submitting Author unless you are acting as an employee on behalf of your employer or a postgraduate student of an affiliated institution which is paying any applicable article publishing charge ("APC") for Open Access articles. Where the Submitting Author wishes to make the Work available on an Open Access basis (and intends to pay the relevant APC), the terms of reuse of such Open Access shall be governed by a Creative Commons licence – details of these licences and which Creative Commons licence will apply to this Work are set out in our licence referred to above.

Other than as permitted in any relevant BMJ Author's Self Archiving Policies, I confirm this Work has not been accepted for publication elsewhere, is not being considered for publication elsewhere and does not duplicate material already published. I confirm all authors consent to publication of this Work and authorise the granting of this licence.

For peer review only

TITLE PAGE

Co-developed neck exercise (EPIC-Neck) versus usual exercise care for people with chronic non-specific neck pain: a multicentre randomised feasibility trial

Internal Reference/Short Title: EPIC-Neck

IRAS number: 331102

Trial registration: ISRCTN81746901

AUTHORS

Jonathan Price[1,2,3*] - Jonathan.price2@nhs.net

Alison Rushton[4] - arushto3@uwo.ca

Natalie Ives[5] - n.j.ives@bham.ac.uk

Marilyn Bradbury[2,3] - marilyn.bradbury1@nhs.net

Priti Parmar[2] - priti.parmar2@nhs.net

Colin J Greaves[3] - c.j.greaves@bham.ac.uk

ABSTRACT

Objectives: To assess the feasibility of conducting a definitive randomised controlled trial evaluating the clinical and cost-effectiveness of the EPIC-Neck programme compared with usual exercise care for people with chronic non-specific neck pain.

For peer review only

For peer review only - http://bmjopen.bmj.com/site/about/guidelines.xhtml

Design: Multicentre, two-arm, parallel-group, individually randomised controlled feasibility trial with an embedded process evaluation.

Setting: Three National Health Service musculoskeletal services in England.

Participants: Adults (≥18 years) with chronic non-specific neck pain lasting >3 months.

Interventions: Participants were randomised (2:1) to EPIC-Neck (an individualised, evidence-based, theory-informed, co-designed exercise programme) or usual exercise care. Participants and outcome assessors were not blinded to allocation.

Feasibility objectives: Progression was assessed using predefined green–amber–red criteria across five domains: (1) recruitment rate; (2) retention rate (percentage returning the neck disability index at 6 months); (3) delivery fidelity (audio-recorded appointments scored on a 0-5 Dreyfus competence scale where ≥3 = acceptable); (4) acceptability to participants and physiotherapists (1-5 acceptability questionnaires where ≥4 = acceptable and interviews analysed deductively using framework analysis against the theoretical framework of acceptability); and (5) contamination (presence of EPIC-Neck components in usual care) scored using the same 0-5 Dreyfus competency scale as delivery fidelity.

Results: Between May 2024 and February 2025, 287 individuals were screened and 36 randomised (24 EPIC-Neck, 12 usual care), yielding a mean recruitment rate of 4.5 participants/month (green). Retention at 6 months was 67% (24/36) (red). Among participants who completed their allocated treatment, 6-month retention was 91% (22/24). Delivery fidelity was moderate (mean 2.4/5; amber), with only 14% of participants receiving EPIC-Neck at an acceptable level. Acceptability was generally

For peer review only - http://bmjopen.bmj.com/site/about/guidelines.xhtml

positive though interviews identified addressable concerns around intervention coherence (amber). Contamination was low (mean fidelity score for controls was 1.1/5; green).

Conclusions: Progression to a definitive trial is feasible with modifications to improve retention and strengthen delivery fidelity and patient understanding of key EPIC-Neck components. Further intervention refinement and repeat fidelity evaluation are recommended prior to definitive testing.

For peer review only

Trial registrations: ISRCTN81746901

Keywords: Neck pain, exercise, self-management, behaviour change, feasibility trial, acceptability, fidelity, complex intervention development

Strengths and limitations

- This multicentre randomised feasibility trial used an embedded mixed-methods process evaluation, allowing integration of quantitative and qualitative data from participants and physiotherapists.
- The high proportion of audio-recorded appointments enabled a robust assessment of delivery fidelity and contamination using a structured scoring framework.
- Inclusive recruitment and consent procedures, including multilingual/video materials and interpreter support, were used to reduce barriers to participation.
- Physiotherapists delivered the intervention to relatively few participants, so some fidelity limitations may reflect early learning effects.

- Fidelity assessments were unblinded, and qualitative interviews were conducted by the intervention developer, creating potential for bias despite mitigation strategies.

Word count: 4131

For peer review only

For peer review only - http://bmjopen.bmj.com/site/about/guidelines.xhtml

BACKGROUND

Neck pain continues to pose a growing global health challenge. The age-standardised number of years lived with disability for neck pain has increased in recent years, rising by 0.12% per year from 2011–2016 and 0.42% per year from 2016–2019, despite advancements in healthcare [1]. Projections suggest this burden will continue to rise, with rates expected to increase from 267 per 100,000 in 2019 to around 360 per 100,000 by 2044 [1]. Although all types of neck pain contribute to this burden, chronic non-specific neck pain is particularly impactful, representing around 60% of all neck pain cases seen in clinical practice and associated with higher pain, greater disability, and reduced work capacity compared with acute neck pain [2-6]. The impact of chronic non-specific neck pain is compounded by recurrent episodes of pain and disability, leading to substantial and repeated healthcare use [7]. Effective treatments are needed to reduce immediate and long-term impact.

Clinical guidelines and multiple systematic reviews support the use of exercise to improve chronic non-specific neck pain and disability [8-16]. However, short-term effects often fall below clinically meaningful thresholds, and the benefits diminish further in the long term [11 17]. Limited adherence may contribute to these outcomes, with at least one-third of individuals not adhering to prescribed programmes and adherence declining noticeably after six weeks [11 18-21]. These factors highlight the need for exercise programmes that maximise effectiveness and support sustained adherence.

To address these limitations, the EPIC-Neck programme was co-developed with patients and physiotherapists using Intervention Mapping [22]. It is an evidence-based,

For peer review only - http://bmjopen.bmj.com/site/about/guidelines.xhtml

theoretically informed, individualised programme that integrates behaviour change techniques and a structured exercise prescription framework to maximise clinical effectiveness and adherence [22].

Prior to evaluating the long-term clinical and cost-effectiveness of the EPIC-Neck programme, the Medical Research Council recommends complex interventions undergo feasibility testing to address uncertainties that could compromise a definitive trial [23]. For the EPIC-Neck programme, key uncertainties included its acceptability to patients and physiotherapists (which may influence adherence and engagement), delivery fidelity (which could dilute treatment effects), and the risk of contamination between intervention and control conditions.

Aims and objectives

Aim: To determine the feasibility of a definitive trial evaluating the clinical and cost-effectiveness of the EPIC-Neck programme compared with usual exercise care for adults with chronic non-specific neck pain.

Primary feasibility objectives, aligned directly with the prespecified traffic-light progression criteria (Table 1), were to:

- Assess mean participant recruitment to the trial.
- Determine participant retention at 6-month follow-up.
- Evaluate delivery fidelity of key EPIC-Neck components
- Identify the presence of key EPIC-Neck components in usual exercise care (contamination)
- Evaluate the acceptability of EPIC-Neck to patients and physiotherapists

For peer review only

For peer review only - http://bmjopen.bmj.com/site/about/guidelines.xhtml

113 Secondary feasibility objectives were to:

- 114 Describe allocated treatment engagement, including treatment initiation, 115 attendance, total appointment time, treatment duration, and completion.
- 116 Assess the safety of the EPIC-Neck programme.
- 117 Explore the demographics of individuals who do and do not enrol in the trial.
- 118 Assess completeness of outcome measures intended for the planned 119 definitive trial.

For peer review only

For peer review only - http://bmjopen.bmj.com/site/about/guidelines.xhtml

Table 1 - Traffic light progression criteria

	Green	Amber	Red	Feasibility outcomes
**Mean recruitment rate**	≥3 participants per month	1-2 participants per month	≤1 participants per month	**Green:** 4.5 participants per month, 95%CI [3.0, 6.1]
**Retention rate**	≥80%	70-79%	<70%	**Red:** 67%, 95% CI [49, 81]
**Delivery fidelity**	No areas of concern (most items ≥3)	Minor problems (≥ 50% of components between 1.5–3.0)	Major problems (most components < 1.5)	**Amber:** Overall mean score 2.4 (SD 1.3, 95% CI: 1.7, 3.1); 53% of components scored 1.5–3
**Contamination**	No EPIC-Neck components present in usual care	Minor overlap	Major overlap	**Green:** Mean checklist score 1.1/5 (SD 1.0, 95%CI: 0.6, 1.6); 1/17 components observed
**Acceptability**	Mean acceptability score of ≥ 4 and no concerns in interviews	Mean acceptability score 3 and/or minor concerns	Mean acceptability score ≤2 and/or major concerns	**Amber:** Mean 3.9 (SD 1.0, 95%CI: 3.7, 4.1); interview concerns were addressable

Green: No areas of concern
Amber: Minor modifications required before continuing to a definitive trial
Red: Major modifications required before continuing to a definitive trial
Abbreviation: CI: Confidence Interval; SD: Standard Deviation

For peer review only

For peer review only - http://bmjopen.bmj.com/site/about/guidelines.xhtml

METHODS

Reporting

This trial is reported in accordance with the Consolidated Standards of Reporting Trials checklist for pilot/feasibility trials (Supplementary File 1) and a published protocol (Supplementary File 2) [24 25].

Trial design

This was a multicentre, two-arm, parallel-group, individually randomised controlled feasibility trial with an embedded convergent mixed methods process evaluation. Participants were randomised in a 2:1 ratio, to either the intervention group (EPIC-Neck programme) or usual exercise care. The trial was registered (ISRCTN81746901) and received ethical approval from South Central - Oxford B Research Ethics Committee (24/SC/0006).

Participant eligibility, identification and consent procedures

Participants were recruited from National Health Service (NHS) community-based and secondary care musculoskeletal services. Adults (≥18 years) with chronic non-specific neck pain were eligible. Exclusions were recent physiotherapy, previous cervical surgery, traumatic or specific neck pathology, neurological involvement, or inability to follow trial procedures. See protocol for full eligibility criteria [25].

NHS clinical staff identified potential participants from waiting lists or trial posters and provided a Participant Information Leaflet (PIL). Potential participants completed a telephone eligibility screen and, if eligible, attended a face-to-face baseline assessment with a research physiotherapist. Written informed consent was obtained

For peer review only - http://bmjopen.bmj.com/site/about/guidelines.xhtml

for the feasibility trial and qualitative interviews, eligibility confirmed, and baseline assessments completed prior to randomisation. Trial materials were available in paper, electronic, and video formats, with audio narration in English or Urdu, and professional interpreters supported screening and consent where required [26 27].

Randomisation

Randomisation was conducted via a secure web-based system (Sealed Envelope). The sequence was computer-generated using random permuted blocks (sizes 3 and 6) with a 2:1 allocation to EPIC-Neck. Allocation was concealed through the automated system, which released the assignment only after baseline data entry by the research physiotherapist.

Blinding

Blinding of treating physiotherapists was not possible due to the nature of the intervention but cross-contamination was minimised by physiotherapists delivering either EPIC-Neck or usual exercise care, but not both. Participants and outcome assessors were not blinded, although blinding procedures will be implemented in the definitive trial.

Physiotherapist consent procedures

Physiotherapists at participating sites provided written informed consent to deliver either EPIC-Neck or usual exercise care and to take part in the process evaluation (audio-recorded appointments/interviews). Full details are reported elsewhere [25].

For peer review only

For peer review only - http://bmjopen.bmj.com/site/about/guidelines.xhtml

Interventions

EPIC-Neck (Intervention arm)

Full details of the EPIC-Neck programme are published elsewhere [22]. In brief, EPIC-Neck is a biopsychosocial, theory-informed intervention designed to support self-management of chronic neck pain through a home-based exercise programme. The programme integrates a structured behaviour change process with targeted exercise prescription to maximise both engagement and effectiveness.

To support long-term engagement, physiotherapists used a behaviour change facilitation guide (Supplementary File 3-Figure S3-1) to help participants achieve four sequential performance objectives (PO):

PO1) Perform physiotherapist prescribed neck exercises

PO2) Independently adapt and progress (or regress) neck exercise

PO3) Use neck exercise to self-manage flare-ups

PO4) Commence general exercise, with or without secondary prevention neck exercise.

Techniques to support these objectives included goal setting, self-monitoring, co-created action plans, social support, and strategies to enhance self-efficacy, delivered using patient-centred communication [28 29].

To prescribe maximally effective exercise, physiotherapists tailored exercises to each participant's physical, psychological, and social needs using a two-stage biopsychosocial exercise framework (Supplementary File 3-Figure S3-2). Exercise

For peer review only

For peer review only - http://bmjopen.bmj.com/site/about/guidelines.xhtml

selection and dosage were personalised based on key mediators such as pain self-efficacy, fear avoidance, and neuromuscular function.

Intervention Delivery

EPIC-Neck was delivered face-to-face by trained physiotherapists and supported by paper and digital patient-facing materials (workbook, exercise diary, exercise library, and friends-and-family leaflet). Non-exercise treatments (e.g. postural advice, manual therapy) were provided at the physiotherapist's discretion. Treatment was completed when physiotherapists and participants agreed on discharge based on progress towards goals, performance objectives achievement and the patient's problem-solving ability.

Physiotherapist training

Physiotherapists delivering EPIC-Neck completed a one-day, face-to-face training combining lectures, role play and behavioural skills practice [30 31]. Ongoing support included a training manual, crib sheets, optional booster sessions, and peer/expert support [30 32].

Usual exercise care (Control arm)

The usual exercise care group received physiotherapy exercises as per the physiotherapists standard practice. Physiotherapists were not given any additional instruction or training. Non-exercise treatments (e.g. postural advice, manual therapy) were provided at the physiotherapist's discretion.

For peer review only

For peer review only - http://bmjopen.bmj.com/site/about/guidelines.xhtml

Outcomes

Feasibility progression criteria

Feasibility of progressing to a definitive trial was assessed across five prespecified domains of uncertainty (recruitment, 6-month retention, delivery fidelity, contamination, and acceptability) using a traffic-light system (green: proceed; amber: proceed with modifications; red: major modifications required) with a priori thresholds (Table 1)[33].

Recruitment

Recruitment was assessed as the mean number of participants randomised per month, calculated from the total randomised over the period of active recruitment [34].

Retention

Retention was assessed as the proportion of randomised participants who returned the Neck Disability Index at 6 months. Six months was chosen as the feasibility timepoint because retention at this stage is expected to predict follow-up at the planned 12-month primary endpoint of a definitive trial [35 36].

Delivery fidelity

Delivery fidelity was evaluated by scoring the quality of physiotherapists' delivery of key EPIC-Neck components. All EPIC-Neck appointments were audio-recorded and coded against a pre-defined fidelity checklist covering 17 items outlining the key components of the EPIC-Neck programme (Supplementary File 3-Table S3-1)[37 38]. Each component was rated using a 0–5 Dreyfus delivery competence scale (0 =

For peer review only - http://bmjopen.bmj.com/site/about/guidelines.xhtml

For peer review only

absent, 3 = acceptable, 5 = optimal) [39]. A more detailed assessment of intervention fidelity informed by the National Institutes of Health-Behaviour Change Consortium (NIH-BCC) fidelity domains (training, delivery, receipt, enactment) will be reported separately [40].

Contamination

Contamination was assessed by examining the presence of EPIC-Neck components within usual exercise care. All usual exercise care appointments were audio-recorded and coded using the same 17-item fidelity checklist and 0–5 Dreyfus competence scale applied to the delivery fidelity evaluation of the EPIC-Neck group [41 42].

Acceptability

EPIC-Neck acceptability to participants and physiotherapists was evaluated using a convergent mixed-methods approach informed by the Theoretical Framework of Acceptability (TFA) [43 44]. Quantitative acceptability was assessed only for EPIC-Neck participants at 6-month follow-up using questionnaires covering TFA domains (affective attitude, intervention coherence, perceived effectiveness, opportunity costs, and self-efficacy) rated on 1–5 Likert scales (≥4 acceptable).

Qualitative acceptability was explored through semi-structured interviews with participants (post-discharge) and physiotherapists (end of trial), using patient and public involvement and engagement (PPIE)- informed topic guides mapped to all seven TFA domains. Interviews were conducted by telephone, video call or face-to-face and audio recorded.

For peer review only - http://bmjopen.bmj.com/site/about/guidelines.xhtml

252 Allocated treatment engagement

253 Allocated treatment engagement was described using treatment initiation,
254 attendance, total appointment time, treatment duration, and treatment completion.
255 Treatment initiation was defined as attendance at ≥1 treatment appointment.
256 Treatment completion was defined as discharge by collaborative agreement between
257 the participant and physiotherapist. Among participants who attended at least one
258 appointment, treatment attendance was described using the number of attended and
259 did not attend (DNA) appointments. Total appointment time was defined as the
260 cumulative duration of physiotherapist-participant face-to-face contact derived from
261 available audio-recordings. Treatment duration was defined as the time in weeks
262 from the initial assessment to the final attended appointment.

263 Inclusivity

264 To explore inclusivity, demographic characteristics of randomised and non-
265 randomised individuals identified from screening logs were compared. Variables
266 included age, gender, ethnicity, first language, interpreter need, and Index of Multiple
267 Deprivation (IMD) decile.

268 Feasibility of trial outcome measures

269 To assess the feasibility of data collection for a future definitive trial, planned trial
270 outcome measures were collected at baseline, 3- and 6-months post-randomisation.
271 Baseline data were collected face-to-face, and follow-up questionnaires were
272 completed by post, with telephone completion and interpreter support available
273 where required. A £20 voucher was offered to participants for returning all three
274 questionnaires. The outcomes were selected based on existing literature, PPIE input

For peer review only - http://bmjopen.bmj.com/site/about/guidelines.xhtml

275 and the EPIC-Neck logic model [22]. The planned definitive trial primary outcome is the
276 Neck Disability Index (NDI), which is widely used with good psychometric properties
277 and a minimum clinically important difference of 5.5 points [45 46]. Secondary outcome
278 measures include pain (Visual Analogue Scale (VAS)) [47]; health-related quality of life
279 (EQ-5D-5L)[48]; mental well-being (Hospital Anxiety and Depression Scale (HADS))[49];
280 sleep quality (The Sleep Condition Indicator)[50]; specific functional or activity
281 limitations (Patient Specific Functional Scale (PSFS)[51] and social and relationships
282 impacts (Relationships and Self-Worth domains of Assessment of Quality-of-Life
283 8D)[52 53]. Candidate mediators were pain beliefs (Single item Survey of Pain Attitudes
284 Questionnaire (SOPA))[54]; pain related fear (Tampa Scale of Kinesiophobia-13 (TSK-
285 13))[55]; pain self-efficacy (Pain self-efficacy questionnaire (PSEQ))[56]; night pain
286 (VAS)[47].

287 **Data analysis**

288 Quantitative data were summarised using means (SD), proportions, and 95%
289 confidence intervals as appropriate for recruitment, retention, delivery fidelity,
290 contamination, and quantitative acceptability scores (domain means and overall
291 acceptability mean) using IBM SPSS v25.

292 For the delivery fidelity and contamination objectives, two researchers (JP, MB)
293 independently coded an initial sample of recordings and iteratively calibrated their
294 scoring (using further recordings) until ≥75% of ratings were within one point. The
295 remaining recordings were then coded by a single researcher (JP)[57 58]. Fidelity and
296 contamination scores were summarised descriptively at component-, participant-,
297 and study-level using mean (SD).

For peer review only

For the allocated treatment engagement objective, the proportions of participants who initiated treatment, completed treatment, and were discharged following non-attendance were summarised descriptively using the number randomised to each group as the denominator. Among participants who attended at least one appointment, the number of attended appointments, the number of DNA appointments, total appointment time, and treatment duration were summarised using mean (SD) and range.

For the feasibility of planned definitive-trial outcome measures questionnaire return rates and data completeness were summarised as percentages at each time point. Outcome scores were summarised descriptively using mean (SD) and 95% confidence intervals at baseline and at 3- and 6-month follow-up. Missing data were reported for all time points. No between-group comparisons or subgroup/adjusted analyses were undertaken [59]. For the inclusivity objective, demographic characteristics of randomised and non-randomised individuals were compared using Pearson's chi-square tests ($p < 0.05$).

Qualitative acceptability data were analysed using deductive framework analysis [60] [61]. Interviews were audio-recorded, transcribed verbatim and coded against TFA domains. Data were then summarised using a domain-by-case matrix, enabling comparison within and across cases (NVivo 12 Plus, QSR International). Patterns of acceptability were interpreted within each TFA domain for participants and physiotherapists separately, identifying areas of minor (Amber), major (Red) or no (Green) acceptability concerns. All interviews and qualitative analysis were completed by JP, an experienced physiotherapist and lead developer of the EPIC-Neck programme. Reflexivity was addressed by acknowledging prior professional

For peer review only - http://bmjopen.bmj.com/site/about/guidelines.xhtml

relationships with some physiotherapists and none with participants. To support analytic rigour and consistency, a subset of domain coding was reviewed within the research team.

Quantitative and qualitative acceptability findings were triangulated using a domain-level table, aligning questionnaire and interview findings. Minor, major, or no acceptability concerns were described across data sources for each domain. These domain-level classifications were combined to provide an overall acceptability judgement.

Sample size

Target recruitment was 45 participants, to allow estimation of retention rates, assuming 80% retention with 95% CI ±12% [62 63]. No interim analyses or stopping rules were planned.

Semi-structured interviews were conducted with a purposive sample of 12 EPIC-Neck participants, sampling for variation in ethnicity, socioeconomic deprivation, and baseline neck disability to reflect potentially under-served groups and diverse pain experiences [64-66]. All physiotherapists delivering the EPIC-Neck programme were also invited to be interviewed.

Patient and public involvement and engagement

The trial protocol, intervention development, outcome selection, inclusivity design, trial materials and dissemination were informed by a PPIE group (n = 17). Two PPIE members were part of the Study Management Group. PPIE contributions are reported in more detail in Supplementary File 4 (GRIPP2-SF)[67].

For peer review only

For peer review only - http://bmjopen.bmj.com/site/about/guidelines.xhtml

RESULTS

Screening and recruitment

Recruitment took place between 29 May 2024 and 10 February 2025 (Figure 1). Of 287 individuals invited, 36 were randomised (24 EPIC-Neck; 12 usual exercise care), giving an uptake rate of 12.5% (95% CI 8.7–16.4). Recruitment stopped before the planned target of 45 participants was reached because the available study time and funding period ended. At the point recruitment stopped, sufficient data had been collected to evaluate the prespecified feasibility objectives.

Participant characteristics

Participants had a mean age of 45.9 years (range 19–82), with a similar number of males and females (Table 2). Overall, 41.7% were of Asian or Asian British ethnic origin, and 72.2% lived in the most deprived IMD deciles.

For peer review only - http://bmjopen.bmj.com/site/about/guidelines.xhtml

Table 2 – Patient participant baseline characteristics

Demographic characteristic	EPIC-Neck n=24		Usual Exercise Care n=12		Full sample n=36	
	n	%	*n*	%	*n*	%
**Gender**						
Female	11	45.8%	5	41.7%	16	44.4%
Male	13	54.2%	7	58.3%	20	55.6%
**Age**						
18-30	3	12.5%	3	25.0%	6	16.7%
31-40	7	29.2%	1	8.3%	8	22.2%
41-50	3	12.5%	3	25.0%	6	16.7%
51-60	7	29.2%	3	25.0%	10	27.8%
61+	4	16.7%	2	16.7%	6	16.7%
**Ethnicity**						
**Asian or Asian British**	9	37.5%	6	50.0%	15	41.7%
Indian	2	8.3%	1	8.3%	3	8.3%
Pakistani	7	29.2%	5	41.7%	12	33.3%
**Black or Black British**	3	12.5%	2	16.7%	5	13.9%
African	1	4.2%	1	8.3%	2	5.6%
Caribbean	1	4.2%	0	0.0%	1	2.8%
Other	1	4.2%	1	8.3%	2	5.6%
**Mixed**	1	4.2%	1	8.3%	2	5.6%
White & Black Caribbean	1	4.2%	1	8.3%	2	5.6%
**White**	10	41.7%	3	25.0%	13	36.1%
British	9	37.5%	3	25.0%	12	33.3%
Irish	1	4.2%	0	0.0%	1	2.8%
**Other**	1	4.2%	0	0.0%	1	2.8%
Arab	1	4.2%	0	0.0%	1	2.8%
**IMD decile**						
Most deprived (deciles 1-3)	19	79.2%	7	58.3%	26	72.2%
Middle (deciles 4-7)	4	16.7%	5	41.7%	9	25.0%
Least deprived (deciles 8-10)	1	4.2%	0	0.0%	1	2.8%
**Marital status**						
Living with partner	6	25.0%	1	8.3%	7	19.4%
Married/civil union	13	54.2%	5	41.7%	18	50.0%
Separated/divorced	1	4.2%	1	8.3%	2	5.6%
Unmarried (never married)	4	16.7%	5	41.7%	9	25.0%
**Cohabiting status**						
Cohabiting	24	100.0%	10	83.3%	34	94.4%
Living alone	0	0.0%	2	16.7%	2	5.6%
**Neck pain location**						
Left	11	45.8%	9	75.0%	20	55.6%

For peer review only - http://bmjopen.bmj.com/site/about/guidelines.xhtml

Right	11	45.8%	6	50.0%	17	47.2%
Middle	7	29.2%	7	58.3%	14	38.9%
Base of skull	2	8.3%	1	8.3%	3	8.3%

Abbreviations: IMD - Index of multiple deprivation

Feasibility objectives

Mean recruitment rate

Over 8 months, 36 participants were randomised, giving a mean recruitment rate of 4.5 participants per month (95% CI: 3.0, 6.1), which exceeded the "Green" progression criterion (Table 1).

Retention rate

At six months, 67% of participants returned the NDI questionnaire (24/36; 95% CI: 49, 81), meeting the "Red" progression criterion (Table 1). Retention was 75% in the EPIC-Neck group (18/24) and 50% in usual exercise care (6/12) (Figure 1). All participants missing at three months were also missing at six months (9/9). Among those not returning the six-month NDI, 41% (5/12) had not initiated treatment and 41% (5/12) were discharged for non-attendance. Retention among treatment completers (collaborative discharge) across both groups was high (22/24; 91%).

Delivery fidelity

Fidelity was scored for 76/80 EPIC-Neck appointments. Four appointments were excluded due to missing recordings (n=2) or a participant developing neurological symptoms (n=2). The overall mean fidelity score was 2.4 out of 5 (SD 1.3; 95% CI: 1.7, 3.1). Component scores across the 17-item checklist ranged from 0.9 to 3.5,

For peer review only

For peer review only - http://bmjopen.bmj.com/site/about/guidelines.xhtml

with 6/17 components (35%) delivered at an acceptable level (≥3) and 9/17 (53%) scoring 1.5–<3.0 (Table 3). Only 3/22 participants (14%) had a mean score ≥3 (participant means 1.3–3.2). Based on the study-level mean and component-level distribution, delivery fidelity met the Amber progression criterion (Table 1).

For peer review only

For peer review only - http://bmjopen.bmj.com/site/about/guidelines.xhtml

Table 3 – Delivery fidelity scores by component

	Mean Score (SD)	
**Component**	**EPIC-Neck**	**UEC**
01 - Agree outcome goals	2.0 (1.3)	0.9 (0.9)
02 - Actively involve the patient	3.1 (0.7)	1.2 (0.4)
03 - Improve motivation to perform exercise (PO1) - Enhance perceived importance and safety	2.1 (1.0)	1.4 (0.6)
04 - Improve motivation to perform exercise (PO1) - Improve self-efficacy	3.5 (0.8)	3.0 (1.1)
05 - Improve motivation to perform exercise (PO1) - Engage social support	1.8 (1.1)	0.4 (0.5)
06 - Set a plan to perform exercise (PO1)	2.3 (1.0)	1.0 (0.0)
07 - Encourage self-monitoring	3.3 (0.9)	0.0 (0.0)
08 - Review progress	2.7 (0.5)	1.6 (0.3)
09 - Support problem solving	1.3 (0.7)	0.8 (0.5)
10 - Introduce adapting and progressing (or regression) exercise (PO2)	1.6 (1.1)	0.2 (0.4)
11 - Introduce flare-up exercise (PO3)	1.5 (1.0)	0.1 (0.3)
12 - Introduce general exercise +/- secondary prevention exercise (PO4)	0.9 (0.9)	0.1 (0.3)
13 - Evaluate self-management and discharge suitability	2.0 (1.2)	0.8 (0.9)
14 - Identify short-term outcomes and exercise objectives	3.1 (0.8)	1.0 (0.0)
15 - Home exercise programme – Normalising movement	3.5 (0.7)	2.0 (1.0)
16 - Home exercise programme – Improving tolerance	2.7 (1.6)	2.2 (0.7)
17 - Exercise review	3.0 (1.2)	2.2 (1.1)
	2.4 (1.3)	1.1 (1.0)

Abbreviations: PO – Performance Objective; SD – Standard deviation; UEC – Usual Exercise Care

For peer review only - http://bmjopen.bmj.com/site/about/guidelines.xhtml

Contamination

Intervention content was scored for 29/32 usual exercise care appointments. Three appointments were excluded due to missing recordings. The mean checklist score was 1.1 out of 5 (SD 1.0; range 0.6–1.5, 95%CI: 0.6, 1.6), and only 1/17 component ('Improve self-efficacy') was delivered in a manner consistent with the EPIC-Neck protocol (Table 3). This indicates low contamination, meeting the "Green" progression criterion (Table 1).

Acceptability

Eighteen EPIC-Neck participants returned acceptability questionnaires (Table 5). Mean scores for each domain (affective attitude, intervention coherence, perceived effectiveness, and self-efficacy) met the pre-defined ≥4 out of 5 threshold; opportunity costs, reflecting the extent to which doing the exercises interfered with other priorities were neutral (mean 3.3, SD 1.0; 95% CI: 2.8, 3.9). The overall acceptability mean was 3.9 (SD 1.0; 95% CI: 3.7, 4.1).

Table 4 – Interviewed patient and physiotherapist characteristics

	n	%
**Patient participants (n = 12)**		
**Gender**		
Female	4	33.3%
Male	8	66.7%
**Age**		
18-30	1	8.3%
31-40	3	25.0%
41-50	2	16.7%
51-60	3	25.0%
61+	3	25.0%
**Ethnicity**		
**Asian or Asian British**	**3**	**25.0%**
Indian	0	0.0%

For peer review only - http://bmjopen.bmj.com/site/about/guidelines.xhtml

For peer review only

Pakistani	3	25.0%
**Black or Black British**	**2**	**16.7%**
African	0	0.0%
Caribbean	1	8.3%
Other	1	8.3%
**Mixed**	**1**	**8.3%**
White & Black Caribbean	1	8.3%
**White**	**5**	**41.7%**
British	5	41.7%
Irish	0	0.0%
**Other**	**1**	**8.3%**
Arab	1	8.3%
**IMD decile**		
Most deprived (deciles 1-3)	10	83.3%
Middle (deciles 4-7)	2	16.7%
Least deprived (deciles 8-10)	0	0.0%
**Neck pain related disability (NDI)**		
No disability	2	16.7%
Mild disability	5	41.7%
Moderate disability	5	41.7%
Severe disability	0	0.0%
Complete disability	0	0.0%
**Physiotherapist (n = 5)**		
**Gender**		
Female	3	60%
Male	2	40%
**Age**		
20-29	1	20%
30-39	1	20%
40-49	1	20%
50-59	2	40%
**Highest qualification**		
Bachelor's degree	5	100%
**Years experience treating chronic non-specific neck pain**		
0-2	2	40%
3-5	0	0%
6-10	2	40%
11-20	1	20%
**Postgraduate training areas**		
Neck pain assessment and treatment	3	60%
Exercise prescription	3	60%
Behaviour change	2	40%
Psychologically informed practice	1	20%
Patient-centred communication techniques	3	60%

Abbreviations: IMD – Index of multiple deprivation

For peer review only - http://bmjopen.bmj.com/site/about/guidelines.xhtml

Interviews were completed with 12 participants and 5 physiotherapists (Table 4). Qualitative analysis (Table 5; full details are in Supplementary File 5) indicated that no participant had concerns about intervention effectiveness. However, there were addressable concerns in other TFA domains, particularly intervention coherence, defined here as participants' understanding of the intervention and how it was intended to work. Participants showed limited understanding of the exercise rationale and the intended performance objectives (exercise progression/regression, flare-up management, and general exercise). Physiotherapists reported no concerns regarding their affective attitude or opportunity costs. They did describe addressable concerns across the remaining TFA domains, particularly around intervention coherence and self-efficacy when delivering newer or more complex components.

For peer review only

Table 5 – Acceptability summary

Domain	Patient Questionnaire* (n = 18) mean (SD) [95%CI]	Patient Interviews (n = 12)	Physiotherapist Interviews (n = 5)	Integrated summary
**Affective attitude**	**Green:** 4.0 (0.8) [3.7, 4.5]	**Amber:** Satisfaction with individualised, targeted approach and associated benefits, though some expressed negative feelings about physical side effects and general exercise; overall, positive outcomes outweighed lack of enjoyment.	**Green:** Enjoyed structured approach and behaviour change techniques	**Amber:** Address patient concerns about physical side effects and attitudes to general exercise
**Burden**	NA	**Amber:** Exercises that were complex or required specific space/positioning increased effort and were often omitted; time burden varied, with integration into daily routines reducing effort.	**Amber:** Cognitive and time burden due to intervention depth, volume and changing practice which improved with exposure.	**Amber:** Address patient burden related to exercise duration and complex exercises, and physiotherapist burden related to intervention depth and change in clinical practice.
**Ethicality**	NA	**Amber:** Alignment with values on individualised, non-pharmacological management, but misalignment for some with values around shared decision-making, dislike of routines, and preference for interpersonal support over written or video resources.	**Amber:** Alignment with individualised biopsychosocial patient centred care beliefs but misaligned for some regarding goal setting and shared decision-making beliefs.	**Amber:** Address patient misalignment with shared decision-making and resource preferences, and physiotherapist misalignment with goal-setting and shared decision-making.
**Intervention coherence**	**Green:** 4.1 (1.1) [3.6, 4.7]	**Red:** Patients understood basic exercise performance but had limited grasp of purpose, benefits, and progression	**Amber:** Coherent with familiar components, but low with new or more complex components	**Red:** Improve patients' understanding of programme rationale, progression strategies, and flare-up

		strategies; understanding of flare-up management and general exercise plans was inconsistent and rarely aligned with EPIC-Neck standards.	– improved with practice but remained limited	management, and strengthen physiotherapists' understanding of how to deliver newer or more complex components.
**Opportunity costs**	**Amber:** 3.3 (1.0) [2.8, 3.9]	**Amber:** Most reported no trade-offs, but some described minor sacrifices outweighed by benefits, while others de-prioritised exercise in favour of other activities.	**Green:** Minimal trade-offs	**Amber:** Address patient de-prioritisation of exercise
**Perceived effectiveness**	**Green:** 4.1 (1.2) [3.5, 4.7]	**Green:** Consistent benefits in pain, disability and function, with sleep, mental well-being, and relationships improvements noted where limited at baseline	**Amber:** Overall perceived as effective with reservations regarding goal setting, social support and the patient resources	**Amber:** Address physiotherapist concerns about goal setting, social support, and patient resources.
**Self-efficacy**	**Green:** 4.1 (0.9) [3.7, 4.5]	**Amber:** Confident with basic exercises and self-managing flare-ups, but confidence declined with complexity, progression strategies, and general exercise citing fear of harm	**Amber:** Confident with familiar components, but low with new or more complex components – improved with practice but remained limited	**Amber:** Address patient confidence with complex exercises, safety, progression strategies, and general exercise; and physiotherapist confidence with new or complex components.
**Overall acceptability**	**Amber:** 3.9 (1.0) [3.7, 4.1]	-	-	**Amber:** Broadly acceptable with addressable concerns, particularly related to intervention coherence; further refinement and re-testing are required before a definitive trial.

Green: No areas of concern, mean acceptability score of ≥ 4 and no areas of concern identified in qualitative interviews

Amber: Minor problems that require attention before continuing to a definitive trial, mean acceptability score 3 and/or minor problems identified in qualitative interviews

For peer review only - http://bmjopen.bmj.com/site/about/guidelines.xhtml

Red: Major problems that require urgent attention before continuing to a definitive trial, mean acceptability score ≤2 and/or major problems identified in qualitative interviews

Abbreviations: SD – Standard deviation

For peer review only

For peer review only - http://bmjopen.bmj.com/site/about/guidelines.xhtml

Integrated interpretation (Table 5) showed that while questionnaires suggested good patient acceptability across most domains, interviews revealed coherence gaps for both participants and physiotherapists. Collectively, the findings showed that EPIC-Neck was acceptable, but with addressable concerns, meeting the “Amber” progression criterion.

Allocated treatment engagement

Two EPIC-Neck participants (2/24; 8%) and three usual exercise care participants (3/12; 25%) did not initiate treatment. Treatment was completed, defined as discharge by collaborative agreement, by 18 EPIC-Neck participants (18/24; 75%) and six usual exercise care participants (6/12; 50%). A further four EPIC-Neck participants (4/24; 17%) and three usual exercise care participants (3/12; 25%) initiated treatment but were discharged following non-attendance in accordance with NHS Trust policy.

Among participants who attended at least one appointment, EPIC-Neck participants attended a mean of 3.6 appointments (SD 1.4, range 1–7) and had a mean of 0.7 DNA appointments (SD 0.8, range 0–3). Usual exercise care participants attended a mean of 3.6 appointments (SD 2.6, range 1–9) and had a mean of 1.1 DNA appointments (SD 1.3, range 0–3). Mean total appointment time was 1 hour 55 minutes (SD 56 minutes, range 27 minutes to 4 hours 7 minutes) in the EPIC-Neck group and 1 hour 30 minutes (SD 52 minutes, range 26 minutes to 2 hours 42 minutes) in the usual exercise care group. Mean treatment duration was 15.9 weeks (SD 8.7, range 0–31) in the EPIC-Neck group and 11.3 weeks (SD 10.0, range 0–29) in the usual exercise care group.

For peer review only

For peer review only - http://bmjopen.bmj.com/site/about/guidelines.xhtml

Safety

No exercise-related serious adverse events were reported. One participant experienced a temporary worsening of symptoms (related, non-serious), and another experienced a transient episode of syncope while at work (unrelated, serious).

Inclusivity

Demographic comparisons between randomised and non-randomised individuals showed no statistically significant differences (Table 6), implying that refusal to take part in the research study was not socially or ethnically patterned, with no significant biases in terms of age, gender or language.

Table 6 - Demographics randomised vs nonrandomised

**Demographic characteristic**	**Nonrandomised (n=251)**		**Randomised (n=36)**		P-value (from Pearson's Chi-Squared Test)
	n	%	*n*	%	
**Gender**					p = 0.110
Female	147	58.6%	16	44.4%	
Male	104	41.4%	20	55.6%	
**Age**					p = 0.445
18-30	33	13.1%	6	16.7%	
31-40	46	18.3%	8	22.2%	
41-50	54	21.5%	6	16.7%	
51-60	47	18.7%	10	27.8%	
61+	71	28.3%	6	16.7%	
**Ethnicity**					p = 0.549
Asian or Asian British	95	37.8%	11	30.6%	
Black or Black British	16	6.4%	5	13.9%	
Mixed	5	2.0%	1	2.8%	
White	73	29.1%	11	30.6%	
Other	7	2.8%	0	0.0%	
Not known	55	21.9%	8	22.2%	
**First language**					p = 0.264
English	84	33.5%	9	25.0%	
Other	50	19.9%	5	13.9%	

For peer review only

Not known	117	46.6%	22	61.1%	
**Interpreter required**					p = 0.434
No	227	90.4%	34	94.4%	
Yes	24	9.6%	2	5.6%	
**IMD decile**					p = 0.761
Most deprived (Deciles 1-3)	193	76.9%	27	75.0%	
Middle (Deciles 4-7)	46	18.3%	8	22.2%	
Least deprived (Deciles 8-10)	12	4.8%	1	2.8%	

Note – For consistency, demographic data has been taken from the screening logs – as the demographic data on screening logs can be different to what is self-reported on baseline questionnaires for randomised participants

Abbreviations: IMD – Index of multiple deprivation

Feasibility of trial outcome measures

Summary statistics (means, SDs, 95% CIs) for outcomes and candidate mediators are shown in Table 7. The PSFS was not completed at baseline by two participants who reported no functional limitations. All other measures had 100% baseline return, and 75% and 67% return at 3 and 6 months, respectively. Return rates and item-level completeness are in Supplementary File 3–Table S3-2.

Table 7 - Outcome measure data collection

Outcome Measure	EPIC-Neck			Usual Exercise Care		
	n	Mean (SD)	95%CI	n	Mean (SD)	95%CI
**Planned definitive trial primary outcome measure**						
Neck pain-related disability – NDI (0 = best, 50 = worst)						
Baseline	24	14.13 (7.12)	11.12, 17.13	12	16.58 (8.84)	10.97, 22.20
3-Month	19	14.21 (10.97)	8.92, 19.50	8	16.50 (10.30)	7.89, 25.11
6-Month	18	12.39 (12.79)	6.03, 18.75	6	16.67 (12.47)	3.58, 29.75
**Planned definitive trial secondary outcome measures**						
Neck pain – VAS (0 = best, 10 = worst)						
Baseline	24	5.63 (1.86)	4.84, 6.41	12	5.67 (2.31)	4.20, 7.13
3-Month	19	4.42 (2.87)	3.04, 5.81	8	4.25 (2.55)	2.12, 6.38
6-Month	18	3.50 (2.64)	2.19, 4.81	6	4.00 (2.28)	1.61, 6.39
Functional and activity impact – PSFS (0 = worst, 10 = best)						
Baseline	22	5.09 (1.77)	4.31, 5.88	12	4.50 (2.43)	2.96, 6.04
3-Month	17	5.47 (2.48)	4.20, 6.74	8	4.75 (2.55)	2.62, 6.88

For peer review only - http://bmjopen.bmj.com/site/about/guidelines.xhtml

6-Month	16	6.69 (2.65)	5.27, 8.10	6	5.17 (2.93)	2.10, 8.24
Sleep – SCI (0 = worst, 32 = best)						
Baseline	24	16.50 (6.49)	13.76, 19.24	12	13.83 (6.39)	9.77, 17.90
3-Month	19	16.84 (9.55)	12.24, 21.44	8	13.88 (8.11)	7.09, 20.66
6-Month	18	18.72 (9.89)	13.80, 23.64	6	15.00 (6.60)	8.07, 21.93
Mental wellbeing – HADS (0 = best, 42 = worst)						
Baseline	24	12.46 (7.87)	9.14, 15.78	12	16.00 (10.59)	9.27, 22.73
3-Month	19	14.32 (10.26)	9.37, 19.26	8	16.50 (14.28)	4.56, 28.44
6-Month	18	12.89 (10.36)	7.74, 18.04	6	12.67 (12.34)	-0.28, 25.62
Relationships and social function – AQoL (0 = worst, 100 = best)						
Baseline	24	74.63 (17.21)	67.36, 81.89	12	70.75 (23.69)	55.70, 85.80
3-Month	19	71.42 (20.87)	61.36, 81.48	8	67.13 (27.30)	44.30, 89.95
6-Month	18	75.56 (20.56)	65.33, 85.78	6	68.83 (23.95)	43.70, 93.97
Health related quality of life – EQ-5D-5L (-0.594= worst, 1 = best)						
Baseline	24	0.65 (0.24)	0.54, 0.75	12	0.60 (0.26)	0.43, 0.77
3-Month	19	0.65 (0.32)	0.49, 0.80	8	0.62 (0.22)	0.44, 0.81
6-Month	18	0.65 (0.36)	0.47, 0.83	6	0.63 (0.20)	0.43, 0.84
**Potential measures of mediation**						
Night pain – NPRS (0 = best, 10 = worst)						
Baseline	24	5.58 (2.75)	4.42, 6.74	12	5.42 (2.35)	3.92, 6.91
3-Month	19	3.95 (2.84)	2.58, 5.32	8	4.63 (2.56)	2.48, 6.77
6-Month	18	3.28 (2.87)	1.85, 4.70	6	4.83 (3.13)	1.55, 8.11
Pain self-efficacy – PSEQ (0 = worst, 60 = best)						
Baseline	24	40.29 (12.67)	34.94, 45.64	12	36.33 (14.30)	27.25, 45.42
3-Month	19	44.53 (15.74)	36.94, 52.11	8	39.25 (13.95)	27.59, 50.91
6-Month	18	47.11 (16.05)	39.13, 55.09	6	41.17 (15.82)	24.57, 57.77
Pain beliefs – SOPA (0 = best, 28 = worst)						
Baseline	24	14.71 (3.63)	13.18, 16.24	12	13.50 (4.15)	10.87, 16.13
3-Month	19	13.95 (4.79)	11.64, 16.26	8	10.63 (3.38)	7.80, 13.45
6-Month	18	13.72 (3.83)	11.82, 15.63	6	10.50 (2.43)	7.95, 13.05
Fear of movement – TSK-13 (13 = best, 52 = worst)						
Baseline	24	27.13 (8.46)	23.55, 30.70	12	28.58 (9.64)	22.46, 34.71
3-Month	19	26.53 (9.77)	21.82, 31.23	8	29.50 (10.32)	20.87, 38.13
6-Month	18	24.44 (9.43)	19.75, 29.13	6	31.17 (8.75)	21.98, 40.35

Abbreviations: AQoL - Assessment of Quality-of-Life (relationships and self-worth domains only; HADS - Hospital Anxiety and Depression Scale (Anxiety and depression scores combined); HADS- Anxiety and depression scores combined; NDI – Neck disability index; PSEQ – Pain self-efficacy questionnaire; PSFS – Patient specific functional scale; SCI – Sleep condition indicator; SD – Standard deviation; SOPA – Survey of Pain Attitudes Questionnaire (single item version); TSK - Tampa Scale of Kinesiophobia; VAS – Visual analogue scale

Progression to a definitive trial

Table 1 summarises feasibility findings against the prespecified traffic-light criteria:

Green for recruitment and contamination; Amber for delivery fidelity and

For peer review only - http://bmjopen.bmj.com/site/about/guidelines.xhtml

acceptability; Red for retention. Overall, progression was considered feasible with modification, specifically to strengthen retention, delivery fidelity, and intervention coherence.

DISCUSSION

This feasibility trial aimed to determine whether a definitive trial evaluating the clinical and cost-effectiveness of a newly developed, evidence-based, theoretically informed, and individualised programme for chronic non-specific neck pain (EPIC-Neck) is feasible. The findings show that a definitive trial is feasible but there are important concerns relating to participant retention, delivery fidelity and intervention coherence. However, the EPIC-Neck programme is broadly acceptable and demonstrably different from usual exercise care.

For peer review only

Recruitment was feasible, but retention posed modifiable challenges

Despite not reaching the estimated sample size, 4–5 participants were randomised per month, demonstrating effective recruitment strategies and exceeded the green progression criteria. In contrast, retention at 6-month was 67%. This is below the ≥80% needed to minimize attritions bias and below rates typically observed in clinical trials, raising concerns over retention strategies if carried forward to a definitive trial [63,68-71].

Standard retention strategies were used (linguistic support, pre-paid envelopes, reminders, financial incentives)[71-73] and while further optimisation may help (electronic and shorter questionnaires, increased incentives)[71,74] our findings suggest that retention maybe influenced by treatment allocation and engagement, rather than follow-up logistics. Retention differed by treatment allocation (50% usual exercise

For peer review only - http://bmjopen.bmj.com/site/about/guidelines.xhtml

care vs 75% EPIC-Neck) and none of the participants who did not start treatment or were discharged for non-attendance returned follow-up measures. In contrast, treatment completers across both groups had 91% retention (22/24). Additionally, treatment completion was higher in the EPIC-Neck (75% vs 50% usual exercise care). Taken together, these patterns indicate engagement-related attrition and possible allocation-preference effects within this unblinded feasibility trial, reinforcing the need for participant blinding in the definitive exercise-versus-exercise trial to reduce allocation-related differences [75 76].

Intervention coherence and delivery are suboptimal

Although overall acceptability was broadly positive, there were clear concerns within the intervention coherence domain, particularly among participants. Many had limited understanding of the programme rationale and performance objectives. These coherence deficits align with the lower delivery fidelity scores for the corresponding components (mean scores <3), suggesting that limited patient understanding was at least partly driven by suboptimal physiotherapist delivery. Physiotherapists also reported reduced confidence and clarity when delivering newer or more complex EPIC-Neck components.

Addressing these issues is crucial before progressing to a definitive trial. Poor fidelity can reduce treatment effects [77], risk misinterpreting null findings [78 79] and threaten internal validity [40]. This is particularly important where fidelity concerns relate to intervention components intended to support long-term self-management (such as the EPIC-Neck performance objectives) given that the primary outcome of a definitive trial would be long-term clinical outcomes. These concerns are, however,

For peer review only - http://bmjopen.bmj.com/site/about/guidelines.xhtml

518 modifiable. Consistent with Medical Research Council guidance, EPIC-Neck and its training package can be refined and re-tested, with a focus on strengthening delivery fidelity and intervention coherence before progressing to a definitive evaluation [23].

EPIC-Neck is broadly acceptable and distinct from usual exercise care

Despite concerns about intervention coherence preventing immediate progression to a definitive trial, EPIC-Neck was broadly acceptable across the remaining domains to both patients and physiotherapists. Additionally, the operationalisation of EPIC-Neck components (e.g., behaviour change strategies, performance objectives) appears different to usual exercise care [80]. This reduces the risk of contamination in a definitive trial diluting between-group effect [81-83]. Overall, EPIC-Neck is a novel and acceptable intervention, warranting refinement and further fidelity testing before definitive evaluation.

Strengths and limitations

A key strength of this trial is the integration of quantitative and qualitative data, incorporating perspectives from both patients and physiotherapists, enabling a more comprehensive assessment of feasibility. A further strength is that 94% of appointments were audio-recorded providing a robust assessment of delivery fidelity and contamination [84]. Finally, efforts to minimise exclusion based on English language and age resulted in a diverse cohort (40% Asian; age range 19–82), enhancing external validity by better reflecting populations attending NHS musculoskeletal services [27].

For peer review only - http://bmjopen.bmj.com/site/about/guidelines.xhtml

This trial also has limitations. Physiotherapists delivered the EPIC-Neck programme to relatively few patients; therefore, some fidelity issues may be overestimated and could improve with increased exposure and practice [85 86]. Fidelity assessments were undertaken unblinded, which may have influenced scoring despite use of a structured competence checklist. In addition, interviews were conducted by the intervention developer, raising the possibility of social-desirability bias, although the use of a topic guide and reflexive discussions helped mitigate this risk.

Conclusion

Progression to a definitive trial evaluating the clinical and cost-effectiveness of EPIC-Neck compared to usual exercise care is feasible, with modification to improve participant retention, delivery fidelity and intervention coherence. The EPIC-Neck intervention was broadly acceptable and different from usual exercise care. Future research should refine the EPIC-Neck programme and re-assess fidelity and retention rates prior to progressing to a definitive trial.

For peer review only - http://bmjopen.bmj.com/site/about/guidelines.xhtml

LIST OF ABBREVIATIONS

AQoL. Assessment of Quality-of-Life
CI. Confidence Interval
DNA. Did Not Attend
HADS. Hospital Anxiety and Depression Scale
IMD. Index of Multiple Deprivation
NDI. Neck disability index
NHS. National Health Service
NIH-BCC. National Institutes of Health-Behaviour Change Consortium
PIL. Participant Information Leaflet
PO. Performance Objectives
PPIE. Patient and Public Involvement and Engagement
PSEQ. Pain self-efficacy questionnaire
PSFS. Patient Specific Functional Scale
SCI. Sleep condition indicator
SD. Standard Deviation
SOPA. Survey of Pain Attitudes Questionnaire
TFA. Theoretical Framework of Acceptability
TSK. Tampa Scale of Kinesiophobia
UEC. Usual Exercise Care
VAS. Visual analogue scale

DECLARATIONS

Ethics approval and consent to participate

The study was registered with ISRCTN81746901 and received ethical approval from South Central - Oxford B Research Ethics Committee (24/SC/0006). Patient participants and physiotherapists gave informed consent to participate in the study before taking part.

Participant consent for publication

Not applicable.

For peer review only

Availability of data and materials

Data are available upon reasonable request. Data sharing requests can be considered via contact with the corresponding author.

Competing interests

The authors declare that they have no competing interests

Funding

Jonathan Price, Clinical Doctoral Research Fellow, NIHR302141 is funded by HEE/NIHR for this research project. The views expressed in this publication are those of the author(s) and not necessarily those of the NIHR, NHS or the UK Department of Health and Social Care.

The funders are not involved in trial design, data collection, management, analysis, writing of the report or the decision to submit this report for publication.

The study sponsor is Birmingham Community Healthcare NHS Foundation Trust (BCHC.researchinnovation@nhs.net).

Author Contributions

JP was the chief investigator and conceived and designed the study, was awarded the funding and drafted the manuscript. AR, NI, CG, PP contributed to the study design, provided specific content and edited the trial protocol and manuscript. MB contributed to data analysis. All authors have reviewed and approved the manuscript.

For peer review only

Collaborators

EPIC-Neck feasibility trial collaborators: Apurba Subedi (Birmingham Community Healthcare NHS Foundation Trust), Hayley Alderton (Berkshire Healthcare NHS Foundation Trust), Katherine Yeung (Birmingham Community Healthcare NHS Foundation Trust), Longsun Yau (Birmingham Community Healthcare NHS Foundation Trust), Paul Matthews (Nottingham University Hospitals NHS Trust), Rajvinder Shokkar (Berkshire Healthcare NHS Foundation Trust)

Acknowledgements

We thank the EPIC-Neck patient and public involvement and engagement group for their contributions.

Author details

[1]Musculoskeletal Physiotherapy Services, Birmingham Community Healthcare NHS Foundation Trust, Birmingham, United Kingdom

[2]Research and Innovation, Birmingham Community Healthcare NHS Foundation Trust, Birmingham, United Kingdom

[3]School of Sport, Exercise and Rehabilitation Sciences, College of Life and Environmental Sciences, University of Birmingham, Birmingham, United Kingdom

[4]School of Physical Therapy, Western University, Ontario, Canada

[5]Institute of Applied Health Research, College of Medical and Dental Sciences, University of Birmingham, Birmingham, United Kingdom

For peer review only

For peer review only - http://bmjopen.bmj.com/site/about/guidelines.xhtml

*Corresponding author

Email: Jonathan.price2@nhs.net

ORCID iDs

Jonathan Price: https://orcid.org/0000-0002-5505-1564

Alison Rushton: https://orcid.org/0000-0001-8114-7669

Natalie Ives: https://orcid.org/0000-0002-1664-7541

Marilyn Bradbury: https://orcid.org/0000-0001-6000-7229

Priti Parmar: https://orcid.org/0000-0003-4344-144X

Colin J Greaves: https://orcid.org/0000-0003-4425-2691

FIGURES

Figure 1 – CONSORT flow diagram. Abbreviations: CNSNP – Chronic non-specific neck pain; DNA – Did not attend; PIL – Participant information leaflet

SUPPLEMENTARY FILES

Supplementary file 1 – CONSORT for pilot and feasibility trials

Supplementary file 2 – Feasibility trial protocol ethics

Supplementary file 3 – Supporting information

Supplementary file 4 – GRIPP2-SF

Supplementary file 5 – Acceptability data

For peer review only

For peer review only - http://bmjopen.bmj.com/site/about/guidelines.xhtml

REFERENCES

1. Xu F, Zhang X, Yang M, et al. Magnitude, temporal trend and inequality in burden of neck pain: an analysis of the Global Burden of Disease Study 2019. *BMC Musculoskelet Disord* 2025;26(1):202. doi: 10.1186/s12891-025-08342-3 [published Online First: 20250227]

2. Myhrvold BL, Irgens P, Robinson HS, et al. Visual trajectory pattern as prognostic factors for neck pain. *Eur J Pain* 2020;24(9):1752-64. doi: 10.1002/ejp.1622 [published Online First: 20200804]

3. Horn ME, Fritz JM. Timing of physical therapy consultation on 1-year healthcare utilization and costs in patients seeking care for neck pain: a retrospective cohort. *BMC Health Serv Res* 2018;18(1):887. doi: 10.1186/s12913-018-3699-0 [published Online First: 20181126]

4. Hey HWD, Lim JXY, Ong JZ, et al. Epidemiology of Neck Pain and Its Impact on Quality-of-Life-A Population-Based, Cross Sectional Study in Singapore. *Spine (Phila Pa 1976)* 2021;46(22):1572-80. doi: 10.1097/BRS.0000000000004071

5. Irgens P, Myhrvold BL, Kongsted A, et al. The clinical course of neck pain: Are trajectory patterns stable over a 1-year period? *Eur J Pain* 2022;26(2):531-42. doi: 10.1002/ejp.1879 [published Online First: 20211103]

6. Hallman DM, Holtermann A, Dencker-Larsen S, et al. Are trajectories of neck-shoulder pain associated with sick leave and work ability in workers? A 1-year prospective study. *BMJ Open* 2019;9(3):e022006. doi: 10.1136/bmjopen-2018-022006 [published Online First: 20190320]

7. Carroll LJ, Hogg-Johnson S, van der Velde G, et al. Course and prognostic factors for neck pain in the general population: results of the Bone and Joint Decade 2000-2010 Task Force on Neck Pain and Its Associated Disorders. *Spine (Phila Pa 1976)* 2008;33(4 Suppl):S75-82. doi: 10.1097/BRS.0b013e31816445be

8. National Institute for Health and Care Excellence. Chronic pain (primary and secondary) in over 16s: assessment of all chronic pain and mangement of chronic primary pain. NICE Guideline NG193, 2021.

9. Bier JD, Scholten-Peeters WGM, Staal JB, et al. Clinical Practice Guideline for Physical Therapy Assessment and Treatment in Patients With Nonspecific Neck Pain. *Phys Ther* 2018;98(3):162-71. doi: 10.1093/ptj/pzx118

10. Blanpied PR, Gross AR, Elliott JM, et al. Neck Pain: Revision 2017. *J Orthop Sports Phys Ther* 2017;47(7):A1-A83. doi: 10.2519/jospt.2017.0302

11. Wilhelm MP, Donaldson M, Griswold D, et al. The Effects of Exercise Dosage on Neck-Related Pain and Disability: A Systematic Review With Meta-analysis. *J Orthop Sports Phys Ther* 2020;50(11):607-21. doi: 10.2519/jospt.2020.9155

12. de Zoete RMJ, Brown L, Oliveira K, et al. The effectiveness of general physical exercise for individuals with chronic neck pain: a systematic review of randomised controlled trials. *European Journal of Physiotherapy 2020;22(3):141-147* 2020

For peer review only - http://bmjopen.bmj.com/site/about/guidelines.xhtml

682 13. Garzonio S, Arbasetti C, Geri T, et al. Effectiveness of Specific Exercise for Deep
683 Cervical Muscles in Nonspecific Neck Pain: A Systematic Review and Meta-Analysis.
684 *Phys Ther* 2022;102(5) doi: 10.1093/ptj/pzac001

685 14. Martin-Gomez C, Sestelo-Diaz R, Carrillo-Sanjuan V, et al. Motor control using cranio-
686 cervical flexion exercises versus other treatments for non-specific chronic neck pain:
687 A systematic review and meta-analysis. *Musculoskelet Sci Pract* 2019;42:52-59. doi:
688 10.1016/j.msksp.2019.04.010 [published Online First: 20190420]

689 15. Li Y, Li S, Jiang J, et al. Effects of yoga on patients with chronic nonspecific neck pain: A
690 PRISMA systematic review and meta-analysis. *Medicine (Baltimore)*
691 2019;98(8):e14649. doi: 10.1097/MD.0000000000014649

692 16. Martini JD, Ferreira GE, Xavier de Araujo F. Pilates for neck pain: A systematic review
693 and meta-analysis of randomised controlled trials. *J Bodyw Mov Ther* 2022;31:37-44.
694 doi: 10.1016/j.jbmt.2022.03.011 [published Online First: 20220320]

695 17. Price J, Rushton A, Tyros V, et al. Expert consensus on the important chronic non-
696 specific neck pain motor control and segmental exercise and dosage variables: An
697 international e-Delphi study. *PLoS One* 2021;16(7):e0253523. doi:
698 10.1371/journal.pone.0253523 [published Online First: 20210701]

699 18. McLean SM, Klaber Moffett JA, Sharp DM, et al. A randomised controlled trial comparing
700 graded exercise treatment and usual physiotherapy for patients with non-specific
701 neck pain (the GET UP neck pain trial). *Man Ther* 2013;18(3):199-205. doi:
702 10.1016/j.math.2012.09.005 [published Online First: 20121022]

703 19. Villanueva-Ruiz I, Falla D, Saez M, et al. Manual therapy and neck-specific exercise are
704 equally effective for treating non-specific neck pain but only when exercise
705 adherence is maximised: A randomised controlled trial. *Musculoskelet Sci Pract*
706 2025;77:103319. doi: 10.1016/j.msksp.2025.103319 [published Online First:
707 20250321]

708 20. Dalager T, Welch A, O'Leary SP, et al. Clinically Relevant Decreases in Neck/Shoulder
709 Pain Among Office Workers Are Associated With Strength Training Adherence and
710 Exercise Compliance: Explorative Analyses From a Randomized Controlled Trial.
711 *Phys Ther* 2023;103(2) doi: 10.1093/ptj/pzac166

712 21. Zebis MK, Andersen CH, Sundstrup E, et al. Time-wise change in neck pain in response
713 to rehabilitation with specific resistance training: implications for exercise
714 prescription. *PLoS One* 2014;9(4):e93867. doi: 10.1371/journal.pone.0093867
715 [published Online First: 20140407]

716 22. Price J, Rushton A, Ives N, et al. Co-development of an evidence-informed, theoretically
717 driven exercise programme for people with chronic non-specific neck pain (the EPIC-
718 Neck programme - "Exercise Prescription Improved through Co-design"). *BMC*
719 *Musculoskelet Disord* 2025;26(1):689. doi: 10.1186/s12891-025-08918-z [published
720 Online First: 20250718]

721 23. Skivington K, Matthews L, Simpson SA, et al. A new framework for developing and
722 evaluating complex interventions: update of Medical Research Council guidance.
723 *BMJ* 2021;374:n2061. doi: 10.1136/bmj.n2061 [published Online First: 20210930]

For peer review only - http://bmjopen.bmj.com/site/about/guidelines.xhtml

24. Eldridge SM, Chan CL, Campbell MJ, et al. CONSORT 2010 statement: extension to randomised pilot and feasibility trials. *BMJ* 2016;355:i5239. doi: 10.1136/bmj.i5239 [published Online First: 20161024]

25. Price J, Rushton A, Ives N, et al. Co-designed neck exercise (EPIC-Neck) vs usual exercise care for people with chronic non-specific neck pain: protocol for a randomised feasibility study with process evaluation. *Pilot Feasibility Stud* 2025;11(1):26. doi: 10.1186/s40814-025-01608-6 [published Online First: 20250308]

26. NHS England. Increasing diversity in research participation: A good practice guide for engaging with underrepresented groups, 2023.

27. National Institute for Health Research. Improving inclusion of under-served groups in clinical reasearch: Guidance from INCLUDE Project, 2022.

28. Miller WR, Rollnick S. Motivational Interviewing, Second Edition: Preparing People for Change: Guilford Publications 2002.

29. Elwyn G, Durand MA, Song J, et al. A three-talk model for shared decision making: multistage consultation process. *BMJ* 2017;359:j4891. doi: 10.1136/bmj.j4891 [published Online First: 20171106]

30. To D, De Carvalho D, Pike A, et al. A qualitative study exploring perceived barriers and enablers to fidelity of training and delivery for an intervention to reduce non-indicated imaging for low back pain. *Chiropr Man Therap* 2023;31(1):6. doi: 10.1186/s12998-023-00480-6 [published Online First: 20230131]

31. Beneciuk JM, George SZ, Greco CM, et al. Targeted interventions to prevent transitioning from acute to chronic low back pain in high-risk patients: development and delivery of a pragmatic training course of psychologically informed physical therapy for the TARGET trial. *Trials* 2019;20(1):256. doi: 10.1186/s13063-019-3350-3 [published Online First: 20190506]

32. Craven K, Holmes J, Powers K, et al. Embedding mentoring to support trial processes and implementation fidelity in a randomised controlled trial of vocational rehabilitation for stroke survivors. *BMC Med Res Methodol* 2021;21(1):203. doi: 10.1186/s12874-021-01382-y [published Online First: 20211003]

33. Mellor K, Albury C, Dutton SJ, et al. Recommendations for progression criteria during external randomised pilot trial design, conduct, analysis and reporting. *Pilot Feasibility Stud* 2023;9(1):59. doi: 10.1186/s40814-023-01291-5 [published Online First: 20230415]

34. Price J, Rushton A, Tyros I, et al. Effectiveness and optimal dosage of exercise training for chronic non-specific neck pain: A systematic review with a narrative synthesis. *PLoS One* 2020;15(6):e0234511. doi: 10.1371/journal.pone.0234511 [published Online First: 20200610]

35. Littlewood C, Bateman M, Brown K, et al. A self-managed single exercise programme versus usual physiotherapy treatment for rotator cuff tendinopathy: a randomised controlled trial (the SELF study). *Clin Rehabil* 2016;30(7):686-96. doi: 10.1177/0269215515593784 [published Online First: 20150709]

For peer review only

For peer review only - http://bmjopen.bmj.com/site/about/guidelines.xhtml

36. Lamb SE, Williamson EM, Heine PJ, et al. Exercises to improve function of the rheumatoid hand (SARAH): a randomised controlled trial. *The Lancet* 2015;385(9966):421-29. doi: https://doi.org/10.1016/S0140-6736(14)60998-3

37. Frost J, Wingham J, Britten N, et al. Home-based rehabilitation for heart failure with reduced ejection fraction: mixed methods process evaluation of the REACH-HF multicentre randomised controlled trial. *BMJ Open* 2019;9(8):e026039. doi: 10.1136/bmjopen-2018-026039 [published Online First: 20190802]

38. Thompson TP, Lambert JD, Greaves CJ, et al. Intervention delivery fidelity assessment of a counseling-based intervention for promoting smoking reduction and increasing physical activity. *Health Psychol* 2018;37(7):627-37. doi: 10.1037/hea0000613 [published Online First: 20180430]

39. Dreyfus SE. The Five-Stage Model of Adult Skill Acquisition. *Bulletin of Science, Technology & Society* 2004;24(3):177-81. doi: 10.1177/0270467604264992

40. Borrelli B. The Assessment, Monitoring, and Enhancement of Treatment Fidelity In Public Health Clinical Trials. *J Public Health Dent* 2011;71(s1):S52-S63. doi: 10.1111/j.1752-7325.2011.00233.x

41. Luker JA, Craig LE, Bennett L, et al. Implementing a complex rehabilitation intervention in a stroke trial: a qualitative process evaluation of AVERT. *BMC Med Res Methodol* 2016;16:52. doi: 10.1186/s12874-016-0156-9 [published Online First: 20160510]

42. Robinson K, Allen F, Darby J, et al. Contamination in complex healthcare trials: the falls in care homes (FinCH) study experience. *BMC Med Res Methodol* 2020;20(1):46. doi: 10.1186/s12874-020-00925-z [published Online First: 20200227]

43. Sekhon M, Cartwright M, Francis JJ. Development of a theory-informed questionnaire to assess the acceptability of healthcare interventions. *BMC Health Serv Res* 2022;22(1):279. doi: 10.1186/s12913-022-07577-3 [published Online First: 20220301]

44. Sekhon M, Cartwright M, Francis JJ. Acceptability of healthcare interventions: an overview of reviews and development of a theoretical framework. *BMC Health Serv Res* 2017;17(1):88. doi: 10.1186/s12913-017-2031-8 [published Online First: 20170126]

45. Vernon H, Mior S. The Neck Disability Index: a study of reliability and validity. *J Manipulative Physiol Ther* 1991;14(7):409-15.

46. Bobos P, MacDermid JC, Walton DM, et al. Patient-Reported Outcome Measures Used for Neck Disorders: An Overview of Systematic Reviews. *J Orthop Sports Phys Ther* 2018;48(10):775-88. doi: 10.2519/jospt.2018.8131 [published Online First: 20180622]

47. Modarresi S, Lukacs MJ, Ghodrati M, et al. A Systematic Review and Synthesis of Psychometric Properties of the Numeric Pain Rating Scale and the Visual Analog Scale for Use in People With Neck Pain. *Clin J Pain* 2021;38(2):132-48. doi: 10.1097/AJP.0000000000000999 [published Online First: 20211026]

48. Feng YS, Kohlmann T, Janssen MF, et al. Psychometric properties of the EQ-5D-5L: a systematic review of the literature. *Qual Life Res* 2021;30(3):647-73. doi: 10.1007/s11136-020-02688-y [published Online First: 20201207]

For peer review only - http://bmjopen.bmj.com/site/about/guidelines.xhtml

49. Zigmond AS, Snaith RP. The hospital anxiety and depression scale. *Acta Psychiatr Scand* 1983;67(6):361-70. doi: 10.1111/j.1600-0447.1983.tb09716.x

50. Espie CA, Kyle SD, Hames P, et al. The Sleep Condition Indicator: a clinical screening tool to evaluate insomnia disorder. *BMJ Open* 2014;4(3):e004183. doi: 10.1136/bmjopen-2013-004183 [published Online First: 20140318]

51. Stratford P, Gill C, Westaway M, et al. Assessing disability and change on individual patients: a report of a patient specific measure. *Physiotherapy canada* 1995;47(4):258-63.

52. Maxwell A, Ozmen M, Iezzi A, et al. Deriving population norms for the AQoL-6D and AQoL-8D multi-attribute utility instruments from web-based data. *Qual Life Res* 2016;25(12):3209-19. doi: 10.1007/s11136-016-1337-z [published Online First: 20160625]

53. Richardson J, Iezzi A, Khan MA, et al. Validity and reliability of the Assessment of Quality of Life (AQoL)-8D multi-attribute utility instrument. *Patient* 2014;7(1):85-96. doi: 10.1007/s40271-013-0036-x

54. Jensen MP, Keefe FJ, Lefebvre JC, et al. One- and two-item measures of pain beliefs and coping strategies. *Pain* 2003;104(3):453-69. doi: 10.1016/S0304-3959(03)00076-9

55. Dupuis F, Cherif A, Batcho C, et al. The Tampa Scale of Kinesiophobia: A Systematic Review of Its Psychometric Properties in People With Musculoskeletal Pain. *Clin J Pain* 2023;39(5):236-47. doi: 10.1097/AJP.0000000000001104 [published Online First: 20230501]

56. Nicholas N. Paper presented at the Annual Conference of the British Psychological Society. 1989

57. Withers TM, Garner NJ, Thorley CS, et al. Intervention fidelity assessment: A sub-study of the Norfolk Diabetes Prevention Study (NDPS). *Br J Health Psychol* 2023;28(3):740-52. doi: 10.1111/bjhp.12651 [published Online First: 20230212]

58. Cross R, Greaves CJ, Withall J, et al. Delivery fidelity of the REACT (REtirement in ACTion) physical activity and behaviour maintenance intervention for community dwelling older people with mobility limitations. *BMC Public Health* 2022;22(1):1112. doi: 10.1186/s12889-022-13496-z [published Online First: 20220603]

59. Thabane L, Ma J, Chu R, et al. A tutorial on pilot studies: the what, why and how. *BMC Med Res Methodol* 2010;10(1):1. doi: 10.1186/1471-2288-10-1 [published Online First: 20100106]

60. Gale NK, Heath G, Cameron E, et al. Using the framework method for the analysis of qualitative data in multi-disciplinary health research. *BMC Med Res Methodol* 2013;13:117. doi: 10.1186/1471-2288-13-117 [published Online First: 20130918]

61. Klingberg S, Stalmeijer RE, Varpio L. Using framework analysis methods for qualitative research: AMEE Guide No. 164. *Med Teach* 2024;46(5):603-10. doi: 10.1080/0142159X.2023.2259073 [published Online First: 20230921]

62. Teare MD, Dimairo M, Shephard N, et al. Sample size requirements to estimate key design parameters from external pilot randomised controlled trials: a simulation

For peer review only

849 study. *Trials* 2014;15(1):264. doi: 10.1186/1745-6215-15-264 [published Online First:
850 20140703]

851 63. Higgins J, Green S. Cochrane Handbook for Systematic Reviews of Interventions: The
852 Cochrane Collaboration, 2011.

853 64. Public Health England. Chronic pain in adults 2017, 2017.

854 65. Adebajo A, Shikoh S, Kumar K, et al. Ethnic minority musculoskeletal health.
855 *Rheumatology (Oxford)* 2018;57(2):201-03. doi: 10.1093/rheumatology/kex006
856 [published Online First: 2017/02/17]

857 66. Versus Arthritis. Chronic pain in England: Unseen, unequal, unfair, 2021.

858 67. Staniszewska S, Brett J, Simera I, et al. GRIPP2 reporting checklists: tools to improve
859 reporting of patient and public involvement in research. *Res Involv Engagem*
860 2017;3(1):13. doi: 10.1186/s40900-017-0062-2 [published Online First: 20170802]

861 68. Hu S, Guinan E, Mockler D, et al. Retention rates and reasons for non-retention in
862 exercise oncology trials in the post-treatment phase-a systematic review. *J Cancer*
863 *Surviv* 2025;19(5):1535-43. doi: 10.1007/s11764-024-01569-4 [published Online
864 First: 20240403]

865 69. Watson S, Wong C, Smith T. Factors associated with trial recruitment and retention of
866 people with osteoarthritis: analysis of 215 randomised controlled trials from 2013-
867 2021. *Physiotherapy Review* 2022;26(2):5-19. doi: 10.5114/phr.2022.117578

868 70. Jacques RM, Ahmed R, Harper J, et al. Recruitment, consent and retention of
869 participants in randomised controlled trials: a review of trials published in the National
870 Institute for Health Research (NIHR) Journals Library (1997-2020). *BMJ Open*
871 2022;12(2):e059230. doi: 10.1136/bmjopen-2021-059230 [published Online First:
872 20220214]

873 71. Elfeky A, Gillies K, Gardner H, et al. Non-randomised evaluations of strategies to
874 increase participant retention in randomised controlled trials: a systematic review.
875 *Syst Rev* 2020;9(1):224. doi: 10.1186/s13643-020-01471-x [published Online First:
876 20200929]

877 72. Gillies K, Kearney A, Keenan C, et al. Strategies to improve retention in randomised
878 trials. *Cochrane Database Syst Rev* 2021;3(3):MR000032. doi:
879 10.1002/14651858.MR000032.pub3 [published Online First: 20210306]

880 73. Brueton V, Stenning SP, Stevenson F, et al. Best practice guidance for the use of
881 strategies to improve retention in randomized trials developed from two consensus
882 workshops. *J Clin Epidemiol* 2017;88:122-32. doi: 10.1016/j.jclinepi.2017.05.010
883 [published Online First: 20170522]

884 74. Elfeky A, Treweek S, Hannes K, et al. Using qualitative methods in pilot and feasibility
885 trials to inform recruitment and retention processes in full-scale randomised trials: a
886 qualitative evidence synthesis. *BMJ Open* 2022;12(4):e055521. doi:
887 10.1136/bmjopen-2021-055521 [published Online First: 20220418]

888 75. Hrobjartsson A, Emanuelsson F, Skou Thomsen AS, et al. Bias due to lack of patient
889 blinding in clinical trials. A systematic review of trials randomizing patients to blind

For peer review only - http://bmjopen.bmj.com/site/about/guidelines.xhtml

and nonblind sub-studies. *Int J Epidemiol* 2014;43(4):1272-83. doi: 10.1093/ije/dyu115 [published Online First: 20140530]

76. Armijo-Olivo S, Fuentes J, da Costa BR, et al. Blinding in Physical Therapy Trials and Its Association with Treatment Effects: A Meta-epidemiological Study. *Am J Phys Med Rehabil* 2017;96(1):34-44. doi: 10.1097/PHM.0000000000000521

77. Cook CE, Thigpen CA. Five good reasons to be disappointed with randomized trials. *J Man Manip Ther* 2019;27(2):63-65. doi: 10.1080/10669817.2019.1589697 [published Online First: 20190314]

78. Carroll C, Patterson M, Wood S, et al. A conceptual framework for implementation fidelity. *Implement Sci* 2007;2:40. doi: 10.1186/1748-5908-2-40 [published Online First: 20071130]

79. Toomey E, Hardeman W, Hankonen N, et al. Focusing on fidelity: narrative review and recommendations for improving intervention fidelity within trials of health behaviour change interventions. *Health Psychol Behav Med* 2020;8(1):132-51. doi: 10.1080/21642850.2020.1738935 [published Online First: 20200312]

80. Kunstler BE, Cook JL, Freene N, et al. Physiotherapists use a small number of behaviour change techniques when promoting physical activity: A systematic review comparing experimental and observational studies. *J Sci Med Sport* 2018;21(6):609-15. doi: 10.1016/j.jsams.2017.10.027 [published Online First: 20171207]

81. Magill N, Knight R, McCrone P, et al. A scoping review of the problems and solutions associated with contamination in trials of complex interventions in mental health. *BMC Med Res Methodol* 2019;19(1):4. doi: 10.1186/s12874-018-0646-z [published Online First: 20190107]

82. Gross A, Kay TM, Paquin JP, et al. Exercises for mechanical neck disorders. *Cochrane Database Syst Rev* 2015;1(1):CD004250. doi: 10.1002/14651858.CD004250.pub5 [published Online First: 20150128]

83. Hemming K, Taljaard M, Moerbeek M, et al. Contamination: How much can an individually randomized trial tolerate? *Stat Med* 2021;40(14):3329-51. doi: 10.1002/sim.8958 [published Online First: 20210507]

84. Toomey E, Matthews J, Guerin S, et al. Development of a Feasible Implementation Fidelity Protocol Within a Complex Physical Therapy-Led Self-Management Intervention. *Phys Ther* 2016;96(8):1287-98. doi: 10.2522/ptj.20150446 [published Online First: 20160303]

85. Rathod T, Belcher J, Montgomery AA, et al. Health services changes: is a run-in period necessary before evaluation in randomised clinical trials? *Trials* 2014;15(1):41. doi: 10.1186/1745-6215-15-41 [published Online First: 20140130]

86. Khan N, Abboudi H, Khan MS, et al. Measuring the surgical 'learning curve': methods, variables and competency. *BJU Int* 2014;113(3):504-8. doi: 10.1111/bju.12197 [published Online First: 20130702]

For peer review only

For peer review only - http://bmjopen.bmj.com/site/about/guidelines.xhtml

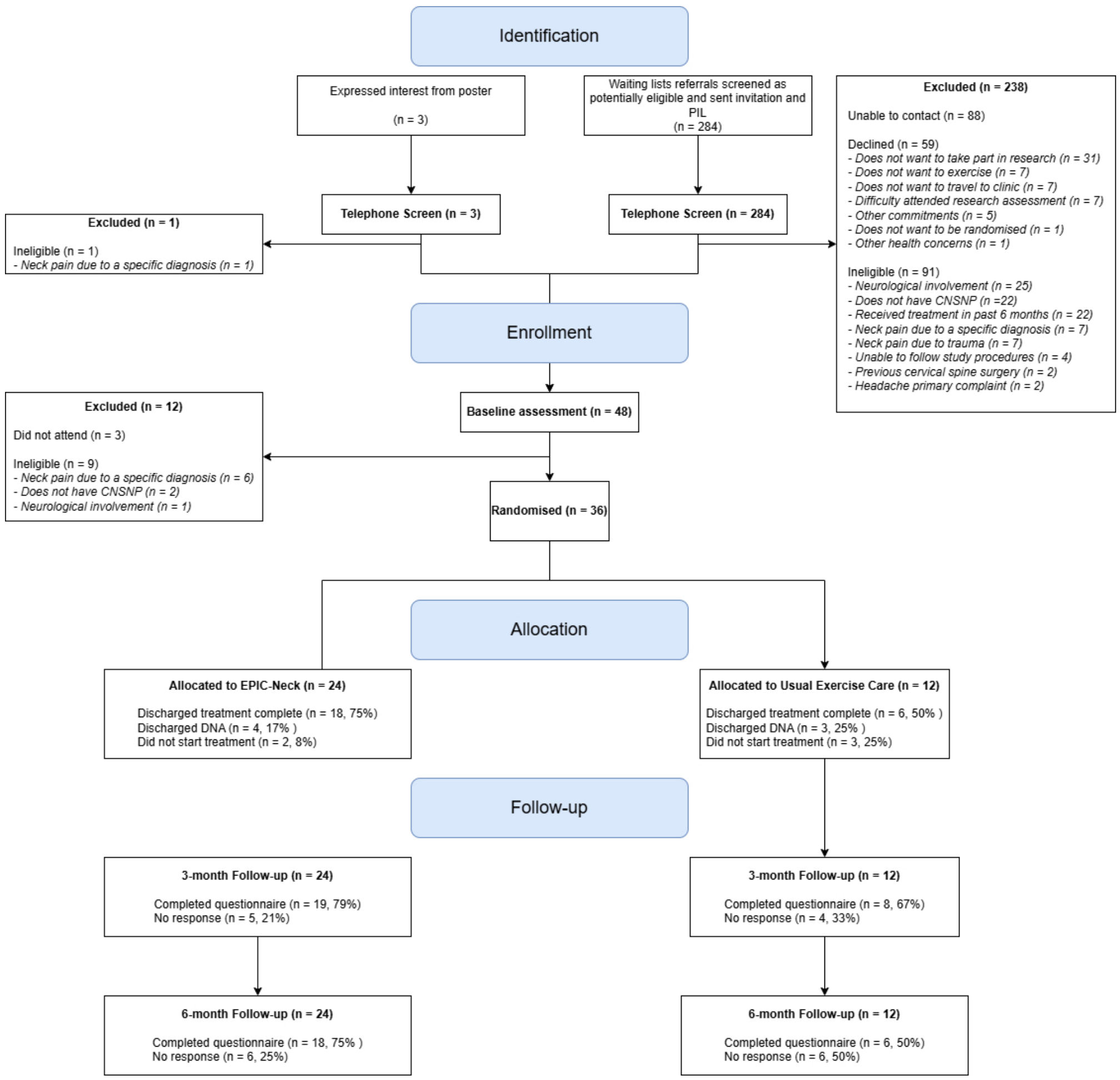

396x381mm (72 x 72 DPI)

BMJ Open

Co-developed neck exercise (EPIC-Neck) versus usual exercise care for people with chronic non-specific neck pain: a UK multicentre randomised feasibility trial

Journal:	*BMJ Open*
Manuscript ID	bmjopen-2026-120571.R1
Article Type:	Original research
Date Submitted by the Author:	21-Jul-2026
Complete List of Authors:	Price, Jonathan; Birmingham Community Healthcare NHS Foundation Trust, Musculoskeletal Physiotherapy Services; Birmingham Community Healthcare NHS Foundation Trust, Research and Innovation; University of Birmingham College of Life and Environmental Sciences, School of Sport, Exercise and Rehabilitation Sciences<br>Rushton, Alison; Western University, School of Physical Therapy<br>Ives, Natalie; University of Birmingham College of Medical and Dental Sciences, Institute of Applied Health Research<br>Bradbury, Marilyn; Birmingham Community Healthcare NHS Foundation Trust, Research and Innovation; University of Birmingham College of Life and Environmental Sciences, School of Sport, Exercise and Rehabilitation Sciences<br>Parmar, Priti ; Birmingham Community Healthcare NHS Foundation Trust, Research and Innovation<br>Greaves, Colin; University of Birmingham College of Life and Environmental Sciences, School of Sport, Exercise and Rehabilitation Sciences
**Primary Subject Heading**:	Rehabilitation medicine
Secondary Subject Heading:	Rehabilitation medicine
Keywords:	Exercise, Self-Management, Feasibility Studies, Behavior, Spine < ORTHOPAEDIC & TRAUMA SURGERY

SCHOLARONE™
MANUSCRIPTS

I, the Submitting Author has the right to grant and does grant on behalf of all authors of the Work (as defined in the below author licence), an exclusive licence and/or a non-exclusive licence for contributions from authors who are: i) UK Crown employees; ii) where BMJ has agreed a CC-BY licence shall apply, and/or iii) in accordance with the terms applicable for US Federal Government officers or employees acting as part of their official duties; on a worldwide, perpetual, irrevocable, royalty-free basis to BMJ Publishing Group Ltd ("BMJ") its licensees and where the relevant Journal is co-owned by BMJ to the co-owners of the Journal, to publish the Work in this journal and any other BMJ products and to exploit all rights, as set out in our licence.

The Submitting Author accepts and understands that any supply made under these terms is made by BMJ to the Submitting Author unless you are acting as an employee on behalf of your employer or a postgraduate student of an affiliated institution which is paying any applicable article publishing charge ("APC") for Open Access articles. Where the Submitting Author wishes to make the Work available on an Open Access basis (and intends to pay the relevant APC), the terms of reuse of such Open Access shall be governed by a Creative Commons licence – details of these licences and which Creative Commons licence will apply to this Work are set out in our licence referred to above.

Other than as permitted in any relevant BMJ Author's Self Archiving Policies, I confirm this Work has not been accepted for publication elsewhere, is not being considered for publication elsewhere and does not duplicate material already published. I confirm all authors consent to publication of this Work and authorise the granting of this licence.

For peer review only

TITLE PAGE

Co-developed neck exercise (EPIC-Neck) versus usual exercise care for people with chronic non-specific neck pain: a UK multicentre randomised feasibility trial

Internal Reference/Short Title: EPIC-Neck

IRAS number: 331102

Trial registration: ISRCTN81746901

AUTHORS

Jonathan Price[1,2,3*] - Jonathan.price2@nhs.net

Alison Rushton[4] - arushto3@uwo.ca

Natalie Ives[5] - n.j.ives@bham.ac.uk

Marilyn Bradbury[2,3] - marilyn.bradbury1@nhs.net

Priti Parmar[2] - priti.parmar2@nhs.net

Colin J Greaves[3] - c.j.greaves@bham.ac.uk

ABSTRACT

Objectives: To assess the feasibility of conducting a definitive randomised controlled trial of the EPIC-Neck programme versus usual exercise care for chronic non-specific neck pain, specifically in relation to recruitment, retention, delivery fidelity, acceptability and contamination.

For peer review only

For peer review only - http://bmjopen.bmj.com/site/about/guidelines.xhtml

Design: Multicentre, two-arm, parallel-group, individually randomised controlled feasibility trial with an embedded process evaluation.

Setting: Three National Health Service musculoskeletal services in England.

Participants: Adults (≥18 years) with chronic non-specific neck pain lasting >3 months.

Interventions: Participants were randomised (2:1) to EPIC-Neck (an individualised, evidence-based, theory-informed, co-designed exercise programme) or usual exercise care. Participants and outcome assessors were not blinded to allocation.

Feasibility objectives: Progression was assessed using predefined green–amber–red criteria across five domains: (1) recruitment rate; (2) retention rate (percentage returning the neck disability index at 6 months); (3) delivery fidelity (audio-recorded appointments scored on a 0-5 Dreyfus competence scale where ≥3 = acceptable); (4) acceptability to participants and physiotherapists (1-5 acceptability questionnaires where ≥4 = acceptable and interviews analysed deductively using framework analysis against the theoretical framework of acceptability); and (5) contamination (presence of EPIC-Neck components in usual care) scored using the same 0-5 Dreyfus competency scale as delivery fidelity.

Results: Between May 2024 and February 2025, 287 individuals were screened and 36 randomised (24 EPIC-Neck, 12 usual care), yielding a mean recruitment rate of 4.5 participants/month (green). Retention at 6 months was 67% (24/36) (red). Among participants who completed their allocated treatment, 6-month retention was 91% (22/24). Delivery fidelity was moderate (mean 2.4/5; amber), with only 14% of participants receiving EPIC-Neck at an acceptable level. Acceptability was generally

For peer review only

For peer review only - http://bmjopen.bmj.com/site/about/guidelines.xhtml

positive though interviews identified addressable concerns around intervention coherence (amber). Contamination was low (mean fidelity score for controls was 1.1/5; green).

Conclusions: EPIC-Neck shows potential for evaluation in a definitive trial, but progression is conditional on future refinement of the intervention and study procedures to optimise retention, delivery fidelity and intervention coherence.

Trial registrations: ISRCTN81746901

Keywords: Neck Pain, Exercise Therapy, Self-Management, Feasibility Studies, Patient Acceptance of Health Care, Intervention Fidelity

Strengths and limitations

- This multicentre randomised feasibility trial used an embedded mixed-methods process evaluation, allowing integration of quantitative and qualitative data from participants and physiotherapists.
- The high proportion of audio-recorded appointments enabled a robust assessment of delivery fidelity and contamination using a structured scoring framework.
- Inclusive recruitment and consent procedures, including multilingual/video materials and interpreter support, were used to reduce barriers to participation.
- Physiotherapists delivered the intervention to relatively few participants, so some fidelity limitations may reflect early learning effects.

For peer review only

For peer review only - http://bmjopen.bmj.com/site/about/guidelines.xhtml

- Fidelity assessments were unblinded, and qualitative interviews were conducted by the intervention developer, creating potential for bias despite mitigation strategies.

Word count: 4131

For peer review only

For peer review only - http://bmjopen.bmj.com/site/about/guidelines.xhtml

BACKGROUND

Neck pain continues to pose a growing global health challenge. The age-standardised number of years lived with disability for neck pain has increased in recent years, rising by 0.12% per year from 2011–2016 and 0.42% per year from 2016–2019, despite advancements in healthcare.[1] Projections suggest this burden will continue to rise, with rates expected to increase from 267 per 100,000 in 2019 to around 360 per 100,000 by 2044.[1] Although all types of neck pain contribute to this burden, chronic non-specific neck pain is particularly impactful because symptoms are associated with greater pain, disability, psychological distress, reduced work capacity and healthcare use than acute neck pain.[2-6] The impact of chronic non-specific neck pain is compounded by recurrent episodes of pain and disability, leading to substantial and repeated healthcare use.[7] Effective treatments are needed to reduce immediate and long-term impact.

Clinical guidelines and multiple systematic reviews support the use of exercise to improve chronic non-specific neck pain and disability.[8-16] However, short-term effects often fall below clinically meaningful thresholds, and the benefits diminish further in the long term.[11 17] Limited adherence may contribute to these outcomes, with at least one-third of individuals not adhering to prescribed programmes and adherence declining noticeably after six weeks.[11 18-21] These factors highlight the need for exercise programmes that maximise effectiveness and support sustained adherence.

To address these limitations, the EPIC-Neck programme (EPIC: Exercise Prescription Improved through Co-design) was co-developed with patients and physiotherapists using Intervention Mapping.[22] EPIC-Neck is an evidence-based,

For peer review only

For peer review only - http://bmjopen.bmj.com/site/about/guidelines.xhtml

theoretically informed exercise programme that integrates behaviour change techniques and a structured exercise prescription framework.[22] It was designed to differ from usual exercise care by combining individualised, physiotherapist-prescribed biopsychosocial exercise with structured behaviour change strategies and patient-facing resources to optimise exercise effects and sustain adherence.[22]

Before evaluating the long-term adherence, clinical effectiveness and cost-effectiveness of EPIC-Neck compared with usual exercise care, key uncertainties that could compromise a definitive trial needed to be addressed.[23] The Medical Research Council recommends that complex interventions, such as EPIC-Neck, undergo feasibility testing before definitive evaluation.[23] For EPIC-Neck, key uncertainties included whether adults with chronic non-specific neck pain could be recruited and retained in an exercise-versus-exercise trial, whether EPIC-Neck was acceptable to patients and physiotherapists, whether key components could be delivered as intended, and whether the intervention remained sufficiently distinct from usual exercise care to avoid contamination between trial arms.

Aims and objectives

Aim: To determine the feasibility of a definitive trial evaluating the clinical and cost-effectiveness of the EPIC-Neck programme compared with usual exercise care for adults with chronic non-specific neck pain.

Primary feasibility objectives, aligned directly with the prespecified traffic-light progression criteria (Table 1), were to:

- Assess mean participant recruitment to the trial.

For peer review only - http://bmjopen.bmj.com/site/about/guidelines.xhtml

- Determine participant retention at 6-month follow-up.
- Evaluate delivery fidelity of key EPIC-Neck components
- Identify the presence of key EPIC-Neck components in usual exercise care (contamination)
- Evaluate the acceptability of EPIC-Neck to patients and physiotherapists

Secondary feasibility objectives were to:

- Describe allocated treatment engagement, including treatment initiation, attendance, total appointment time, treatment duration, and completion.
- Assess the safety of the EPIC-Neck programme.
- Explore the demographics of individuals who do and do not enrol in the trial.
- Assess completeness of outcome measures intended for the planned definitive trial.

For peer review only - http://bmjopen.bmj.com/site/about/guidelines.xhtml

Table 1 - Traffic light progression criteria

	Green	Amber	Red	Feasibility outcomes
**Mean recruitment rate**	≥3 participants per month	1-2 participants per month	≤1 participants per month	**Green:** 4.5 participants per month, 95%CI [3.0, 6.1]
**Retention rate**	≥80%	70-79%	<70%	**Red:** 67%, 95% CI [49, 81]
**Delivery fidelity**	No areas of concern (most items ≥3)	Minor problems (≥ 50% of components between 1.5–3.0)	Major problems (most components < 1.5)	**Amber:** Overall mean score 2.4 (SD 1.3, 95% CI: 1.7, 3.1); 53% of components scored 1.5–3
**Contamination**	No EPIC-Neck components present in usual care	Minor overlap	Major overlap	**Green:** Mean checklist score 1.1/5 (SD 1.0, 95%CI: 0.6, 1.6); 1/17 components observed
**Acceptability**	Mean acceptability score of ≥ 4 and no concerns in interviews	Mean acceptability score 3 and/or minor concerns	Mean acceptability score ≤2 and/or major concerns	**Amber:** Mean 3.9 (SD 1.0, 95%CI: 3.7, 4.1); interview concerns were addressable

Green: No areas of concern
Amber: Minor modifications required before continuing to a definitive trial
Red: Major modifications required before continuing to a definitive trial
Abbreviation: CI: Confidence Interval; SD: Standard Deviation

For peer review only

For peer review only - http://bmjopen.bmj.com/site/about/guidelines.xhtml

METHODS

Reporting

This trial is reported in accordance with the Consolidated Standards of Reporting Trials checklist for pilot/feasibility trials (Supplementary File 1) and a published protocol (Supplementary File 2).[24 25]

Trial design

This was a multicentre, two-arm, parallel-group, individually randomised controlled feasibility trial. Participants were randomised in a 2:1 ratio to either the intervention group (EPIC-Neck programme) or usual exercise care. The feasibility trial evaluated outcomes important for deciding whether and how to progress to a definitive trial, including recruitment, retention, treatment engagement, acceptability, contamination, outcome-measure completion and safety. Embedded within the feasibility trial was a mixed-methods process evaluation, which included a multidimensional fidelity evaluation to understand training, delivery, receipt and enactment of EPIC-Neck and to inform intervention and training refinements. This manuscript reports the feasibility outcomes relevant to progression to a definitive trial; a detailed multidimensional evaluation of fidelity (reporting training, fidelity, delivery fidelity, receipt and enactment) will be reported separately. The trial was registered (ISRCTN81746901) and received ethical approval from South Central - Oxford B Research Ethics Committee (24/SC/0006).

For peer review only - http://bmjopen.bmj.com/site/about/guidelines.xhtml

Participant eligibility, identification and consent procedures

Participants were recruited from National Health Service (NHS) community-based and secondary care musculoskeletal services. Adults (≥18 years) with chronic (≥3 months) non-specific neck pain were eligible. Non-specific neck pain was defined as pain or discomfort in the neck and/or shoulder girdle, with or without referred arm pain, for which no specific cause could be identified.[26 27] Exclusions were recent physiotherapy (within 6 months), previous cervical surgery, traumatic or specific neck pathology (e.g., whiplash, rheumatoid arthritis, osteoarthritis), neurological involvement (e.g., myelopathy or radiculopathy), or inability to follow trial procedures. See protocol for full eligibility criteria.[25]

NHS clinical staff identified potential participants from waiting lists or trial posters and provided a Participant Information Leaflet (PIL). Potential participants completed a telephone eligibility screen and, if eligible, attended a face-to-face baseline assessment with a research physiotherapist. Written informed consent was obtained for the feasibility trial and qualitative interviews, eligibility confirmed, and baseline assessments completed prior to randomisation. Trial materials were available in paper, electronic, and video formats, with audio narration in English or Urdu, and professional interpreters supported screening and consent where required.[28 29]

Randomisation

Randomisation was conducted via a secure web-based system (Sealed Envelope). The sequence was computer-generated using random permuted blocks (sizes 3 and 6) with a 2:1 allocation to EPIC-Neck. A 2:1 allocation ratio in favour of the intervention group was used to ensure sufficient intervention exposure and

For peer review only

For peer review only - http://bmjopen.bmj.com/site/about/guidelines.xhtml

participant feedback to inform feasibility, acceptability and intervention refinement.[30] Allocation was concealed through the automated system, which released the assignment only after baseline data entry by the research physiotherapist.

Blinding

Blinding of treating physiotherapists was not possible due to the nature of the intervention but cross-contamination was minimised by physiotherapists delivering either EPIC-Neck or usual exercise care, but not both. Participants and outcome assessors were not blinded, although blinding procedures will be implemented in the definitive trial.

Physiotherapist consent procedures

Physiotherapists at participating sites provided written informed consent to deliver either EPIC-Neck or usual exercise care and to take part in the process evaluation (audio-recorded appointments/interviews). Physiotherapist characteristics (age, gender, qualifications, years' experience, and postgraduate training) were collected via a self-reported questionnaire prior to training and were not independently verified against certificates or records. Full details are reported elsewhere.[25]

Interventions

EPIC-Neck (Intervention arm)

Full details of the EPIC-Neck programme are published elsewhere.[22] In brief, EPIC-Neck is a biopsychosocial, theory-informed intervention designed to support self-management of chronic neck pain through a home-based exercise programme. Physiotherapists prescribed exercises using a biopsychosocial framework to target

For peer review only

For peer review only - http://bmjopen.bmj.com/site/about/guidelines.xhtml

participants' physical, psychological and social needs (Supplementary File 3, Figure S3-2). A behaviour change facilitation guide supported exercise engagement using techniques such as goal setting, self-monitoring, action planning, social support and self-efficacy enhancement, delivered through patient-centred communication (Supplementary File 3, Figure S3-1).[31 32] EPIC-Neck was delivered face-to-face by trained physiotherapists and supported by paper and digital patient-facing materials (workbook, exercise diary, exercise library, and friends-and-family leaflet). Non-exercise treatments (e.g. postural advice, manual therapy) were provided at the physiotherapist's discretion. Discharge was agreed collaboratively based on goals and self-management ability.

Physiotherapist training

Physiotherapists delivering EPIC-Neck completed a one-day, face-to-face training combining lectures, role play and behavioural skills practice.[33 34] Ongoing support included a training manual, crib sheets, optional booster sessions, and peer/expert support.[33 35] Audio-recordings of intervention delivery were used for fidelity evaluation, but real-time monitoring, feedback or delivery coaching were not provided during the trial.

Usual exercise care (Control arm)

The usual exercise care group received physiotherapy exercises as per the physiotherapists standard practice. Physiotherapists were not given any additional instruction or training. Non-exercise treatments (e.g. postural advice, manual therapy) were provided at the physiotherapist's discretion.

For peer review only - http://bmjopen.bmj.com/site/about/guidelines.xhtml

Outcomes

Feasibility progression criteria

Feasibility of progressing to a definitive trial was assessed across five prespecified domains of uncertainty (recruitment, 6-month retention, delivery fidelity, contamination, and acceptability) using a traffic-light system (green: proceed; amber: proceed with modifications; red: major modifications required) with a priori thresholds (Table 1).[36]

Recruitment

The mean number of participants randomised per month was calculated across the active recruitment period.[37] Thresholds were based on the estimated time required to recruit the anticipated definitive trial sample size of 248 participants across 5 musculoskeletal services.[37] Green indicated recruitment within <18 months, amber indicated 18–30 months, and red indicated >30 months.

Retention

Return of the Neck Disability Index (NDI) at 6 months was used as the retention criterion, selected as an indicator of likely follow-up at the planned 12-month primary endpoint in a definitive trial.[38,39] Thresholds were informed by guidance that ≥80% retention represents low risk of attrition bias and comparable musculoskeletal trials reporting approximately 70% retention; therefore, ≥80% was classified as green and <70% as red.[40-45]

For peer review only

For peer review only - http://bmjopen.bmj.com/site/about/guidelines.xhtml

237 Delivery fidelity

238 All EPIC-Neck appointments were audio-recorded and coded against a pre-defined 17-item checklist of key EPIC-Neck components (Supplementary File 3-Table S3-1).[46 47] Each component was rated using a 0–5 Dreyfus delivery competence scale, where 0 indicated absent delivery, ≥3 acceptable delivery and 5 optimal delivery.[48] Progression thresholds were based on the distribution of checklist scores across components: green indicated most components delivered acceptably (≥3) consistent with previous fidelity research using this approach (≥3),[49 47 50] amber indicated mixed or inconsistent delivery requiring refinement, and red indicated major fidelity concerns.

247 Contamination

248 Presence of EPIC-Neck components within usual exercise care was assessed using audio-recorded usual care appointments, coded against the same 17-item checklist and 0–5 Dreyfus competence scale used for EPIC-Neck delivery fidelity.[51 52] Thresholds reflected the extent to which EPIC-Neck components appeared in usual care: green indicated no or minimal overlap, amber limited overlap, and red substantial overlap suggesting insufficient distinction between trial arms. Final progression judgements were made through discussion within the study team (JP, MB and CG), informed by the checklist scores.

256 Acceptability

257 EPIC-Neck acceptability to participants and physiotherapists was evaluated using a convergent mixed-methods approach informed by the Theoretical Framework of Acceptability (TFA).[53 54] EPIC-Neck participants completed 6-month questionnaires

For peer review only - http://bmjopen.bmj.com/site/about/guidelines.xhtml

covering TFA domains of affective attitude, intervention coherence, perceived effectiveness, opportunity costs and self-efficacy. Items were rated on 1–5 Likert scales and interpreted at face value according to the questionnaire response anchors, where scores of 4–5 indicated positive acceptability, 3 indicated neutrality and 1–2 indicated negative acceptability.[53] Qualitative acceptability was explored through semi-structured interviews with participants (post-discharge) and physiotherapists (end of trial), using patient and public involvement and engagement (PPIE)- informed topic guides mapped to all seven TFA domains. Interviews were conducted by telephone, video call or face-to-face and audio recorded. Progression criteria combined these quantitative thresholds with qualitative findings: green indicated positive acceptability with no substantive interview concerns, amber indicated neutral or mixed acceptability and/or addressable qualitative concerns, and red indicated negative acceptability and/or major qualitative concerns. Final progression judgements were made through discussion between JP and CG, informed by questionnaire scores and qualitative findings. Any disagreements were resolved through discussion with a third study team member.

Allocated treatment engagement

Allocated treatment engagement was described using treatment initiation, attendance, did-not-attend appointments, total appointment time, treatment duration and treatment completion. Treatment initiation was defined as attendance at ≥1 appointment, and treatment completion as collaboratively agreed discharge. Total appointment time was calculated from available audio-recordings, and treatment duration was defined as weeks from initial assessment to final attended appointment.

For peer review only - http://bmjopen.bmj.com/site/about/guidelines.xhtml

For peer review only

Inclusivity

Demographic characteristics of randomised and non-randomised individuals identified from screening logs were compared. Variables included age, gender, ethnicity, first language, interpreter need and deprivation, measured using Index of Multiple Deprivation (IMD) decile. The IMD is a composite measure of area-level deprivation in England, incorporating domains including income, employment, education, health, crime, housing, and living environment, with deciles ranging from 1 (most deprived 10%) to 10 (least deprived 10%).

Feasibility of trial outcome measures

To assess the feasibility of data collection for a future definitive trial, planned trial outcome measures were collected at baseline, 3- and 6-months post-randomisation. Baseline data were collected face-to-face, and follow-up questionnaires were completed by post, with telephone completion and interpreter support available where required. A £20 voucher was offered to participants for returning all three questionnaires. The outcomes were selected based on existing literature, PPIE input and the EPIC-Neck logic model.[22] The planned definitive trial primary outcome is the Neck Disability Index, which is widely used with good psychometric properties and a minimum clinically important difference of 5.5 points.[55 56] Secondary outcome measures include pain (Visual Analogue Scale (VAS));[57] health-related quality of life (EQ-5D-5L);[58] mental well-being (Hospital Anxiety and Depression Scale (HADS));[59] sleep quality (The Sleep Condition Indicator);[60] specific functional or activity limitations (Patient Specific Functional Scale (PSFS)[61] and social and relationships impacts (Relationships and Self-Worth domains of Assessment of Quality-of-Life 8D).[62 63] Candidate mediators were pain beliefs (Single item Survey of Pain Attitudes

For peer review only - http://bmjopen.bmj.com/site/about/guidelines.xhtml

Questionnaire (SOPA));[64] pain related fear (Tampa Scale of Kinesiophobia-13 (TSK-13));[65] pain self-efficacy (Pain self-efficacy questionnaire (PSEQ));[66] night pain (VAS).[57]

Data analysis

Quantitative data were analysed using IBM SPSS v25 and summarised using means (SD), proportions, and 95% confidence intervals as appropriate. For delivery fidelity and contamination, two researchers (JP, MB) independently coded an initial sample of audio-recordings and iteratively calibrated scoring using further recordings until ≥75% of ratings were within one point.[49,67] Remaining recordings were coded by JP. Fidelity and contamination scores were summarised descriptively at component-, participant- and study-level using means (SD). Treatment initiation, completion and discharge following non-attendance were summarised using the number randomised to each group as the denominator. Among participants attending ≥1 appointment, attendance, non-attendance, total appointment time and treatment duration were summarised using means, standard deviations and ranges. Demographic characteristics of randomised and non-randomised individuals were compared using Pearson's chi-square tests ($p < 0.05$). As this was a feasibility trial, planned definitive-trial outcome measures were collected to assess data collection feasibility, completeness, distribution and suitability for a future definitive trial, not to assess clinical effectiveness; therefore, no inferential between-group comparisons were planned or undertaken.[68] Return rates and data completeness were summarised as percentages at each time point, and outcome scores were summarised descriptively using mean (SD) and 95% confidence intervals.

For peer review only

330 Qualitative acceptability data were analysed using deductive framework analysis.[69 70] Interviews were audio-recorded, transcribed verbatim and coded against TFA domains. Data were then summarised using a domain-by-case matrix, enabling comparison within and across cases (NVivo 12 Plus, QSR International). Patterns of acceptability were interpreted within each TFA domain for participants and physiotherapists separately, identifying areas of minor (Amber), major (Red) or no (Green) acceptability concerns. All interviews and qualitative analysis were completed by JP, an experienced physiotherapist and lead developer of the EPIC-Neck programme. Reflexivity was addressed by acknowledging prior professional relationships with some physiotherapists and none with participants. To support analytic rigour and consistency, a subset of domain coding was reviewed within the research team.

342 Quantitative and qualitative acceptability findings were triangulated using a domain-level table, aligning questionnaire and interview findings. Minor, major, or no acceptability concerns were described across data sources for each domain. These domain-level classifications were combined to provide an overall acceptability judgement.

347 Sample size

348 Target recruitment was 45 participants, based on a precision calculation to estimate retention with sufficient accuracy to inform a future definitive trial. Assuming 80% retention in this feasibility trial, approximately 43 participants were required to estimate retention with a 95% confidence interval of ±12%; this was rounded to 45 participants.[44 71] No interim analyses or stopping rules were planned.

For peer review only - http://bmjopen.bmj.com/site/about/guidelines.xhtml

Semi-structured interviews were conducted with a purposive sample of 12 EPIC-Neck participants, sampling for variation in ethnicity, socioeconomic deprivation, and baseline neck disability to reflect potentially under-served groups and diverse pain experiences.[72-74] All physiotherapists delivering the EPIC-Neck programme were invited to be interviewed.

Patient and public involvement and engagement

The trial protocol, intervention development, outcome selection, inclusivity design, trial materials and dissemination were informed by a PPIE group (n = 17). Two PPIE members were part of the Study Management Group. PPIE contributions are reported in more detail in Supplementary File 4 (GRIPP2-SF).[75]

RESULTS

Screening and recruitment

Recruitment took place between 29 May 2024 and 10 February 2025 (Figure 1). Of 287 individuals invited, 36 were randomised (24 EPIC-Neck; 12 usual exercise care), giving an uptake rate of 12.5% (95% CI 8.7–16.4). Recruitment stopped before the planned target of 45 participants was reached because the available study time and funding period ended. At the point recruitment stopped, sufficient data had been collected to evaluate the prespecified feasibility objectives.

Participant characteristics

Participants had a mean age of 45.9 years (range 19–82), with a similar number of males and females (Table 2). Overall, 41.7% were of Asian or Asian British ethnic origin, and 72.2% lived in the most deprived IMD deciles.

For peer review only

375 **Table 2 – Patient participant baseline characteristics**

Demographic characteristic	EPIC-Neck n=24		Usual Exercise Care n=12		Full sample n=36	
	n	%	*n*	%	*n*	%
**Gender**						
Female	11	45.8%	5	41.7%	16	44.4%
Male	13	54.2%	7	58.3%	20	55.6%
**Age**						
18-30	3	12.5%	3	25.0%	6	16.7%
31-40	7	29.2%	1	8.3%	8	22.2%
41-50	3	12.5%	3	25.0%	6	16.7%
51-60	7	29.2%	3	25.0%	10	27.8%
61+	4	16.7%	2	16.7%	6	16.7%
**Ethnicity**						
**Asian or Asian British**	9	37.5%	6	50.0%	15	41.7%
Indian	2	8.3%	1	8.3%	3	8.3%
Pakistani	7	29.2%	5	41.7%	12	33.3%
**Black or Black British**	3	12.5%	2	16.7%	5	13.9%
African	1	4.2%	1	8.3%	2	5.6%
Caribbean	1	4.2%	0	0.0%	1	2.8%
Other	1	4.2%	1	8.3%	2	5.6%
**Mixed**	1	4.2%	1	8.3%	2	5.6%
White & Black Caribbean	1	4.2%	1	8.3%	2	5.6%
**White**	10	41.7%	3	25.0%	13	36.1%
British	9	37.5%	3	25.0%	12	33.3%
Irish	1	4.2%	0	0.0%	1	2.8%
**Other**	1	4.2%	0	0.0%	1	2.8%
Arab	1	4.2%	0	0.0%	1	2.8%
**IMD decile**						
Most deprived (deciles 1-3)	19	79.2%	7	58.3%	26	72.2%
Middle (deciles 4-7)	4	16.7%	5	41.7%	9	25.0%
Least deprived (deciles 8-10)	1	4.2%	0	0.0%	1	2.8%
**Marital status**						
Living with partner	6	25.0%	1	8.3%	7	19.4%
Married/civil union	13	54.2%	5	41.7%	18	50.0%
Separated/divorced	1	4.2%	1	8.3%	2	5.6%
Unmarried (never married)	4	16.7%	5	41.7%	9	25.0%
**Cohabiting status**						
Cohabiting	24	100.0%	10	83.3%	34	94.4%
Living alone	0	0.0%	2	16.7%	2	5.6%
**Neck pain location**						
Left	11	45.8%	9	75.0%	20	55.6%

For peer review only - http://bmjopen.bmj.com/site/about/guidelines.xhtml

Right	11	45.8%	6	50.0%	17	47.2%
Middle	7	29.2%	7	58.3%	14	38.9%
Base of skull	2	8.3%	1	8.3%	3	8.3%

Abbreviations: IMD - Index of multiple deprivation

Feasibility objectives

Mean recruitment rate

Over 8 months, 36 participants were randomised, giving a mean recruitment rate of 4.5 participants per month (95% CI: 3.0, 6.1), which exceeded the "Green" progression criterion (Table 1).

Retention rate

At six months, 67% of participants returned the NDI questionnaire (24/36; 95% CI: 49, 81), meeting the "Red" progression criterion (Table 1). Retention was 75% in the EPIC-Neck group (18/24) and 50% in usual exercise care (6/12) (Figure 1). All participants missing at three months were also missing at six months (9/9). Among those not returning the six-month NDI, 41% (5/12) had not initiated treatment and 41% (5/12) were discharged for non-attendance. Retention among treatment completers (collaborative discharge) across both groups was high (22/24; 91%).

Delivery fidelity

Fidelity was scored for 76/80 EPIC-Neck appointments. Four appointments were excluded due to missing recordings (n=2) or a participant developing neurological symptoms (n=2). The overall mean fidelity score was 2.4 out of 5 (SD 1.3; 95% CI: 1.7, 3.1). Component scores across the 17-item checklist ranged from 0.9 to 3.5,

For peer review only

For peer review only - http://bmjopen.bmj.com/site/about/guidelines.xhtml

with 6/17 components (35%) delivered at an acceptable level (≥3) and 9/17 (53%) scoring 1.5–<3.0 (Table 3). Only 3/22 participants (14%) had a mean score ≥3 (participant means 1.3–3.2). Based on the study-level mean and component-level distribution, delivery fidelity met the Amber progression criterion (Table 1).

For peer review only

For peer review only - http://bmjopen.bmj.com/site/about/guidelines.xhtml

400 **Table 3 – Delivery fidelity scores by component**

	Mean Score (SD)	
**Component**	**EPIC-Neck**	**UEC**
01 - Agree outcome goals	2.0 (1.3)	0.9 (0.9)
02 - Actively involve the patient	3.1 (0.7)	1.2 (0.4)
03 - Improve motivation to perform exercise (PO1) - Enhance perceived importance and safety	2.1 (1.0)	1.4 (0.6)
04 - Improve motivation to perform exercise (PO1) - Improve self-efficacy	3.5 (0.8)	3.0 (1.1)
05 - Improve motivation to perform exercise (PO1) - Engage social support	1.8 (1.1)	0.4 (0.5)
06 - Set a plan to perform exercise (PO1)	2.3 (1.0)	1.0 (0.0)
07 - Encourage self-monitoring	3.3 (0.9)	0.0 (0.0)
08 - Review progress	2.7 (0.5)	1.6 (0.3)
09 - Support problem solving	1.3 (0.7)	0.8 (0.5)
10 - Introduce adapting and progressing (or regression) exercise (PO2)	1.6 (1.1)	0.2 (0.4)
11 - Introduce flare-up exercise (PO3)	1.5 (1.0)	0.1 (0.3)
12 - Introduce general exercise +/- secondary prevention exercise (PO4)	0.9 (0.9)	0.1 (0.3)
13 - Evaluate self-management and discharge suitability	2.0 (1.2)	0.8 (0.9)
14 - Identify short-term outcomes and exercise objectives	3.1 (0.8)	1.0 (0.0)
15 - Home exercise programme – Normalising movement	3.5 (0.7)	2.0 (1.0)
16 - Home exercise programme – Improving tolerance	2.7 (1.6)	2.2 (0.7)
17 - Exercise review	3.0 (1.2)	2.2 (1.1)
	2.4 (1.3)	1.1 (1.0)

401 Abbreviations: PO – Performance Objective; SD – Standard deviation; UEC – Usual Exercise Care

For peer review only - http://bmjopen.bmj.com/site/about/guidelines.xhtml

Contamination

Intervention content was scored for 29/32 usual exercise care appointments. Three appointments were excluded due to missing recordings. The mean checklist score was 1.1 out of 5 (SD 1.0; range 0.6–1.5, 95%CI: 0.6, 1.6), and only 1/17 component ('Improve self-efficacy') was delivered in a manner consistent with the EPIC-Neck protocol (Table 3). This indicates low contamination, meeting the "Green" progression criterion (Table 1).

Acceptability

Eighteen EPIC-Neck participants returned acceptability questionnaires (Table 5). Mean scores for each domain (affective attitude, intervention coherence, perceived effectiveness, and self-efficacy) met the pre-defined ≥4 out of 5 threshold; opportunity costs, reflecting the extent to which doing the exercises interfered with other priorities were neutral (mean 3.3, SD 1.0; 95% CI: 2.8, 3.9). The overall acceptability mean was 3.9 (SD 1.0; 95% CI: 3.7, 4.1).

Table 4 – Interviewed patient and physiotherapist characteristics

	n	%
**Patient participants (n = 12)**		
**Gender**		
Female	4	33.3%
Male	8	66.7%
**Age**		
18-30	1	8.3%
31-40	3	25.0%
41-50	2	16.7%
51-60	3	25.0%
61+	3	25.0%
**Ethnicity**		
**Asian or Asian British**	**3**	**25.0%**
Indian	0	0.0%

For peer review only - http://bmjopen.bmj.com/site/about/guidelines.xhtml

Pakistani	3	25.0%
**Black or Black British**	**2**	**16.7%**
African	0	0.0%
Caribbean	1	8.3%
Other	1	8.3%
**Mixed**	**1**	**8.3%**
White & Black Caribbean	1	8.3%
**White**	**5**	**41.7%**
British	5	41.7%
Irish	0	0.0%
**Other**	**1**	**8.3%**
Arab	1	8.3%
**IMD decile**		
Most deprived (deciles 1-3)	10	83.3%
Middle (deciles 4-7)	2	16.7%
Least deprived (deciles 8-10)	0	0.0%
**Neck pain related disability (NDI)**		
No disability	2	16.7%
Mild disability	5	41.7%
Moderate disability	5	41.7%
Severe disability	0	0.0%
Complete disability	0	0.0%
**Physiotherapist (n = 5)**		
**Gender**		
Female	3	60%
Male	2	40%
**Age**		
20-29	1	20%
30-39	1	20%
40-49	1	20%
50-59	2	40%
**Highest qualification**		
Bachelor's degree	5	100%
**Years experience treating chronic non-specific neck pain**		
0-2	2	40%
3-5	0	0%
6-10	2	40%
11-20	1	20%
**Postgraduate training areas**		
Neck pain assessment and treatment	3	60%
Exercise prescription	3	60%
Behaviour change	2	40%
Psychologically informed practice	1	20%
Patient-centred communication techniques	3	60%

417 Abbreviations: IMD – Index of multiple deprivation

For peer review only - http://bmjopen.bmj.com/site/about/guidelines.xhtml

418 Interviews were completed with 12 participants and 5 physiotherapists (Table 4). Qualitative analysis (Table 5; full details are in Supplementary File 5) indicated that no participant had concerns about intervention effectiveness. However, there were addressable concerns in other TFA domains, particularly intervention coherence, defined here as participants' understanding of the intervention and how it was intended to work. Participants showed limited understanding of the exercise rationale and the intended performance objectives (exercise progression/regression, flare-up management, and general exercise). Physiotherapists reported no concerns regarding their affective attitude or opportunity costs. They did describe addressable concerns across the remaining TFA domains, particularly around intervention coherence and self-efficacy when delivering newer or more complex components.

For peer review only - http://bmjopen.bmj.com/site/about/guidelines.xhtml

Table 5 – Acceptability summary

Domain	Patient Questionnaire* (n = 18) mean (SD) [95%CI]	Patient Interviews (n = 12)	Physiotherapist Interviews (n = 5)	Integrated summary
**Affective attitude**	**Green:** 4.0 (0.8) [3.7, 4.5]	**Amber:** Satisfaction with individualised, targeted approach and associated benefits, though some expressed negative feelings about physical side effects and general exercise; overall, positive outcomes outweighed lack of enjoyment.	**Green:** Enjoyed structured approach and behaviour change techniques	**Amber:** Address patient concerns about physical side effects and attitudes to general exercise
**Burden**	NA	**Amber:** Exercises that were complex or required specific space/positioning increased effort and were often omitted; time burden varied, with integration into daily routines reducing effort.	**Amber:** Cognitive and time burden due to intervention depth, volume and changing practice which improved with exposure.	**Amber:** Address patient burden related to exercise duration and complex exercises, and physiotherapist burden related to intervention depth and change in clinical practice.
**Ethicality**	NA	**Amber:** Alignment with values on individualised, non-pharmacological management, but misalignment for some with values around shared decision-making, dislike of routines, and preference for interpersonal support over written or video resources.	**Amber:** Alignment with individualised biopsychosocial patient centred care beliefs but misaligned for some regarding goal setting and shared decision-making beliefs.	**Amber:** Address patient misalignment with shared decision-making and resource preferences, and physiotherapist misalignment with goal-setting and shared decision-making.
**Intervention coherence**	**Green:** 4.1 (1.1) [3.6, 4.7]	**Red:** Patients understood basic exercise performance but had limited grasp of purpose, benefits, and progression	**Amber:** Coherent with familiar components, but low with new or more complex components	**Red:** Improve patients' understanding of programme rationale, progression strategies, and flare-up

For peer review only - http://bmjopen.bmj.com/site/about/guidelines.xhtml

		strategies; understanding of flare-up management and general exercise plans was inconsistent and rarely aligned with EPIC-Neck standards.	– improved with practice but remained limited	management, and strengthen physiotherapists' understanding of how to deliver newer or more complex components.
**Opportunity costs**	**Amber:** 3.3 (1.0) [2.8, 3.9]	**Amber:** Most reported no trade-offs, but some described minor sacrifices outweighed by benefits, while others de-prioritised exercise in favour of other activities.	**Green:** Minimal trade-offs	**Amber:** Address patient de-prioritisation of exercise
**Perceived effectiveness**	**Green:** 4.1 (1.2) [3.5, 4.7]	**Green:** Consistent benefits in pain, disability and function, with sleep, mental well-being, and relationships improvements noted where limited at baseline	**Amber:** Overall perceived as effective with reservations regarding goal setting, social support and the patient resources	**Amber:** Address physiotherapist concerns about goal setting, social support, and patient resources.
**Self-efficacy**	**Green:** 4.1 (0.9) [3.7, 4.5]	**Amber:** Confident with basic exercises and self-managing flare-ups, but confidence declined with complexity, progression strategies, and general exercise citing fear of harm	**Amber:** Confident with familiar components, but low with new or more complex components – improved with practice but remained limited	**Amber:** Address patient confidence with complex exercises, safety, progression strategies, and general exercise; and physiotherapist confidence with new or complex components.
**Overall acceptability**	**Amber:** 3.9 (1.0) [3.7, 4.1]	-	-	**Amber:** Broadly acceptable with addressable concerns, particularly related to intervention coherence; further refinement and re-testing are required before a definitive trial.

Green: No areas of concern, mean acceptability score of ≥ 4 and no areas of concern identified in qualitative interviews

Amber: Minor problems that require attention before continuing to a definitive trial, mean acceptability score 3 and/or minor problems identified in qualitative interviews

For peer review only - http://bmjopen.bmj.com/site/about/guidelines.xhtml

Red: Major problems that require urgent attention before continuing to a definitive trial, mean acceptability score ≤2 and/or major problems identified in qualitative interviews

Abbreviations: SD – Standard deviation

For peer review only

For peer review only - http://bmjopen.bmj.com/site/about/guidelines.xhtml

Integrated interpretation (Table 5) showed that while questionnaires suggested good patient acceptability across most domains, interviews revealed coherence gaps for both participants and physiotherapists. Collectively, the findings showed that EPIC-Neck was acceptable, but with addressable concerns, meeting the “Amber” progression criterion.

Allocated treatment engagement

Two EPIC-Neck participants (2/24; 8%) and three usual exercise care participants (3/12; 25%) did not initiate treatment. Treatment was completed by 18 EPIC-Neck participants (18/24; 75%) and six usual exercise care participants (6/12; 50%). A further four EPIC-Neck participants (4/24; 17%) and three usual exercise care participants (3/12; 25%) initiated treatment but were discharged following non-attendance in accordance with NHS Trust policy.

Among participants who attended at least one appointment, EPIC-Neck participants attended a mean of 3.6 appointments (SD 1.4, range 1–7) and had a mean of 0.7 DNA appointments (SD 0.8, range 0–3). Usual exercise care participants attended a mean of 3.6 appointments (SD 2.6, range 1–9) and had a mean of 1.1 DNA appointments (SD 1.3, range 0–3). Mean total appointment time was 1 hour 55 minutes (SD 56 minutes, range 27 minutes to 4 hours 7 minutes) in the EPIC-Neck group and 1 hour 30 minutes (SD 52 minutes, range 26 minutes to 2 hours 42 minutes) in the usual exercise care group. Mean treatment duration was 15.9 weeks (SD 8.7, range 0–31) in the EPIC-Neck group and 11.3 weeks (SD 10.0, range 0–29) in the usual exercise care group.

For peer review only

Safety

No exercise-related serious adverse events were reported. One participant experienced a temporary worsening of symptoms (related, non-serious), and another experienced a transient episode of syncope while at work (unrelated, serious).

Inclusivity

No statistically significant differences in age, gender, ethnicity, first language, interpreter need or deprivation were observed between randomised and non-randomised individuals (Table 6).

Table 6 - Demographics randomised vs nonrandomised

**Demographic characteristic**	**Nonrandomised (n=251)**		**Randomised (n=36)**		P-value (from Pearson's Chi-Squared Test)
	n	%	*n*	%	
**Gender**					p = 0.110
Female	147	58.6%	16	44.4%	
Male	104	41.4%	20	55.6%	
**Age**					p = 0.445
18-30	33	13.1%	6	16.7%	
31-40	46	18.3%	8	22.2%	
41-50	54	21.5%	6	16.7%	
51-60	47	18.7%	10	27.8%	
61+	71	28.3%	6	16.7%	
**Ethnicity**					p = 0.549
Asian or Asian British	95	37.8%	11	30.6%	
Black or Black British	16	6.4%	5	13.9%	
Mixed	5	2.0%	1	2.8%	
White	73	29.1%	11	30.6%	
Other	7	2.8%	0	0.0%	
Not known	55	21.9%	8	22.2%	
**First language**					p = 0.264
English	84	33.5%	9	25.0%	
Other	50	19.9%	5	13.9%	
Not known	117	46.6%	22	61.1%	
**Interpreter required**					p = 0.434

For peer review only

For peer review only - http://bmjopen.bmj.com/site/about/guidelines.xhtml

No	227	90.4%	34	94.4%	
Yes	24	9.6%	2	5.6%	
**IMD decile**					p = 0.761
Most deprived (Deciles 1-3)	193	76.9%	27	75.0%	
Middle (Deciles 4-7)	46	18.3%	8	22.2%	
Least deprived (Deciles 8-10)	12	4.8%	1	2.8%	

Note – For consistency, demographic data has been taken from the screening logs – as the demographic data on screening logs can be different to what is self-reported on baseline questionnaires for randomised participants

Abbreviations: IMD – Index of multiple deprivation

Feasibility of trial outcome measures

Summary statistics (means, SDs, 95% CIs) for outcomes and candidate mediators are shown in Table 7. The PSFS was not completed at baseline by two participants who reported no functional limitations. All other measures had 100% baseline return, and 75% and 67% return at 3 and 6 months, respectively. Return rates and item-level completeness are in Supplementary File 3–Table S3-2.

Table 7 - Descriptive summary of planned definitive trial outcome and candidate mediator measures

Outcome Measure	EPIC-Neck			Usual Exercise Care		
	n	Mean (SD)	95%CI	n	Mean (SD)	95%CI
**Planned definitive trial primary outcome measure**						
Neck pain-related disability – NDI (0 = best, 50 = worst)						
Baseline	24	14.13 (7.12)	11.12, 17.13	12	16.58 (8.84)	10.97, 22.20
3-Month	19	14.21 (10.97)	8.92, 19.50	8	16.50 (10.30)	7.89, 25.11
6-Month	18	12.39 (12.79)	6.03, 18.75	6	16.67 (12.47)	3.58, 29.75
**Planned definitive trial secondary outcome measures**						
Neck pain – VAS (0 = best, 10 = worst)						
Baseline	24	5.63 (1.86)	4.84, 6.41	12	5.67 (2.31)	4.20, 7.13
3-Month	19	4.42 (2.87)	3.04, 5.81	8	4.25 (2.55)	2.12, 6.38
6-Month	18	3.50 (2.64)	2.19, 4.81	6	4.00 (2.28)	1.61, 6.39
Functional and activity impact – PSFS (0 = worst, 10 = best)						
Baseline	22	5.09 (1.77)	4.31, 5.88	12	4.50 (2.43)	2.96, 6.04
3-Month	17	5.47 (2.48)	4.20, 6.74	8	4.75 (2.55)	2.62, 6.88
6-Month	16	6.69 (2.65)	5.27, 8.10	6	5.17 (2.93)	2.10, 8.24
Sleep – SCI (0 = worst, 32 = best)						

For peer review only - http://bmjopen.bmj.com/site/about/guidelines.xhtml

Baseline	24	16.50 (6.49)	13.76, 19.24	12	13.83 (6.39)	9.77, 17.90
3-Month	19	16.84 (9.55)	12.24, 21.44	8	13.88 (8.11)	7.09, 20.66
6-Month	18	18.72 (9.89)	13.80, 23.64	6	15.00 (6.60)	8.07, 21.93
Mental wellbeing – HADS (0 = best, 42 = worst)						
Baseline	24	12.46 (7.87)	9.14, 15.78	12	16.00 (10.59)	9.27, 22.73
3-Month	19	14.32 (10.26)	9.37, 19.26	8	16.50 (14.28)	4.56, 28.44
6-Month	18	12.89 (10.36)	7.74, 18.04	6	12.67 (12.34)	-0.28, 25.62
Relationships and social function – AQoL (0 = worst, 100 = best)						
Baseline	24	74.63 (17.21)	67.36, 81.89	12	70.75 (23.69)	55.70, 85.80
3-Month	19	71.42 (20.87)	61.36, 81.48	8	67.13 (27.30)	44.30, 89.95
6-Month	18	75.56 (20.56)	65.33, 85.78	6	68.83 (23.95)	43.70, 93.97
Health related quality of life – EQ-5D-5L (-0.594= worst, 1 = best)						
Baseline	24	0.65 (0.24)	0.54, 0.75	12	0.60 (0.26)	0.43, 0.77
3-Month	19	0.65 (0.32)	0.49, 0.80	8	0.62 (0.22)	0.44, 0.81
6-Month	18	0.65 (0.36)	0.47, 0.83	6	0.63 (0.20)	0.43, 0.84
**Potential measures of mediation**						
Night pain – NPRS (0 = best, 10 = worst)						
Baseline	24	5.58 (2.75)	4.42, 6.74	12	5.42 (2.35)	3.92, 6.91
3-Month	19	3.95 (2.84)	2.58, 5.32	8	4.63 (2.56)	2.48, 6.77
6-Month	18	3.28 (2.87)	1.85, 4.70	6	4.83 (3.13)	1.55, 8.11
Pain self-efficacy – PSEQ (0 = worst, 60 = best)						
Baseline	24	40.29 (12.67)	34.94, 45.64	12	36.33 (14.30)	27.25, 45.42
3-Month	19	44.53 (15.74)	36.94, 52.11	8	39.25 (13.95)	27.59, 50.91
6-Month	18	47.11 (16.05)	39.13, 55.09	6	41.17 (15.82)	24.57, 57.77
Pain beliefs – SOPA (0 = best, 28 = worst)						
Baseline	24	14.71 (3.63)	13.18, 16.24	12	13.50 (4.15)	10.87, 16.13
3-Month	19	13.95 (4.79)	11.64, 16.26	8	10.63 (3.38)	7.80, 13.45
6-Month	18	13.72 (3.83)	11.82, 15.63	6	10.50 (2.43)	7.95, 13.05
Fear of movement – TSK-13 (13 = best, 52 = worst)						
Baseline	24	27.13 (8.46)	23.55, 30.70	12	28.58 (9.64)	22.46, 34.71
3-Month	19	26.53 (9.77)	21.82, 31.23	8	29.50 (10.32)	20.87, 38.13
6-Month	18	24.44 (9.43)	19.75, 29.13	6	31.17 (8.75)	21.98, 40.35

Abbreviations: AQoL - Assessment of Quality-of-Life (relationships and self-worth domains only; HADS - Hospital Anxiety and Depression Scale (Anxiety and depression scores combined); HADS- Anxiety and depression scores combined; NDI – Neck disability index; PSEQ – Pain self-efficacy questionnaire; PSFS – Patient specific functional scale; SCI – Sleep condition indicator; SD – Standard deviation; SOPA – Survey of Pain Attitudes Questionnaire (single item version); TSK - Tampa Scale of Kinesiophobia; VAS – Visual analogue scale

Progression to a definitive trial

Table 1 summarises feasibility findings against the prespecified traffic-light criteria: Green for recruitment and contamination; Amber for delivery fidelity and acceptability; Red for retention. Overall, progression was considered feasible with

For peer review only - http://bmjopen.bmj.com/site/about/guidelines.xhtml

modification, specifically to strengthen retention, delivery fidelity, and intervention coherence.

DISCUSSION

This feasibility trial aimed to determine whether a definitive trial evaluating the clinical and cost-effectiveness of a newly developed, evidence-based, theoretically informed, and individualised programme for chronic non-specific neck pain (EPIC-Neck) is feasible. The feasibility findings were mixed. Recruitment and contamination met Green progression criteria, indicating no major concerns in these domains. However, retention met the Red criterion and delivery fidelity met the Amber criterion, indicating important feasibility concerns that require resolution before a definitive trial. Acceptability to both patients and physiotherapists was broadly positive, but qualitative findings identified addressable concerns within the TFA domain of intervention coherence, particularly participants' understanding of the intervention rationale and physiotherapists' clarity around delivering newer or more complex components.

Recruitment was feasible, but retention posed modifiable challenges

Despite not reaching the estimated sample size, 4–5 participants were randomised per month, demonstrating effective recruitment strategies and exceeded the green progression criteria. In contrast, retention at 6-month was 67%. This is below the ≥80% target associated with lower risk of attrition bias and below the minimum 70% threshold prespecified for progression, raising concerns about whether current retention strategies would be sufficient in a definitive trial..[40-44]

For peer review only

Standard retention strategies were used (linguistic support, pre-paid envelopes, reminders, financial incentives)[43 76 77] and while further optimisation may help (electronic and shorter questionnaires, increased incentives)[43 78] our findings suggest that retention maybe influenced by treatment allocation and engagement, rather than follow-up logistics. Retention differed by treatment allocation (50% usual exercise care vs 75% EPIC-Neck) and none of the participants who did not start treatment or were discharged for non-attendance returned follow-up measures. In contrast, treatment completers across both groups had 91% retention (22/24). Additionally, treatment completion was higher in the EPIC-Neck (75% vs 50% usual exercise care). Taken together, these patterns indicate engagement-related attrition and possible allocation-preference effects within this unblinded feasibility trial, reinforcing the need to consider participant blinding in the definitive exercise-versus-exercise trial to reduce allocation-related differences.[79 80]

Intervention coherence and delivery are suboptimal

Although overall acceptability was broadly positive, there were clear concerns within the intervention coherence domain, particularly among participants. Many had limited understanding of the programme rationale and performance objectives. These coherence deficits align with lower delivery fidelity scores for the corresponding components (mean scores <3), suggesting that limited patient understanding may have been related, at least in part, to suboptimal physiotherapist delivery. Physiotherapists also reported reduced confidence and clarity when delivering newer or more complex EPIC-Neck components.

For peer review only - http://bmjopen.bmj.com/site/about/guidelines.xhtml

Addressing these issues is crucial before progressing to a definitive trial. Poor fidelity can reduce treatment effects,[81] risk misinterpreting null findings [82 83] and threaten internal validity.[84] However, delivery fidelity should not be interpreted as a simple success/failure judgement. Some EPIC-Neck components were delivered acceptably, and evidence from complex behavioural interventions indicates that effectiveness may still be possible despite variable or moderate delivery fidelity, depending on which components are most critical to the intervention mechanism.[46 85 86] So EPIC-Neck may still prove to be effective. Nevertheless, fidelity concerns are particularly important for EPIC-Neck components intended to support long-term self-management, such as flare-up management and long-term exercise, because these may plausibly influence long-term clinical outcomes in a definitive trial. These concerns are, however, modifiable. Consistent with Medical Research Council guidance, EPIC-Neck and its training package can be refined and re-tested, with a focus on strengthening delivery fidelity and intervention coherence before progressing to a definitive evaluation.[23]

EPIC-Neck was acceptable with concerns about intervention coherence and was distinct from usual exercise care

Despite concerns about intervention coherence preventing immediate progression to a definitive trial, EPIC-Neck was broadly acceptable across the remaining domains to both patients and physiotherapists. Additionally, the operationalisation of EPIC-Neck components (e.g., behaviour change strategies, performance objectives) appears different to usual exercise care.[87] This reduces the risk of contamination in a definitive trial diluting between-group effect.[88-90] Overall, EPIC-Neck appears

For peer review only

For peer review only - http://bmjopen.bmj.com/site/about/guidelines.xhtml

558 acceptable and sufficiently distinct from usual exercise care, but requires refinement and further fidelity testing before definitive evaluation.

Future refinement before a definitive trial

Before progression to a definitive trial, key refinements are needed to optimise retention, delivery fidelity and intervention coherence. Retention strategies should address both questionnaire return and treatment engagement, as missing follow-up data appeared linked to treatment non-initiation and non-attendance. Future procedures could consider reducing questionnaire burden (e.g., electronic completion or shorter questionnaires), increasing the financial incentive for completion, and incorporating blinding procedures where feasible.[43 78-80] Additional strategies to optimise delivery and intervention coherence should be designed collaboratively with patients and physiotherapists, and informed by theory and existing evidence. Potential refinements include strengthening physiotherapist training through longer training duration, additional practice, and regular monitoring and feedback.[91-95] Intervention coherence may improve through better delivery, but additional strategies could include methods to check patient understanding during treatment, such as teach-back approaches.[96]

Strengths and limitations

A key strength of this trial is the integration of quantitative and qualitative data, incorporating perspectives from both patients and physiotherapists, enabling a more comprehensive assessment of feasibility. A further strength is that 94% of appointments were audio-recorded providing a robust assessment of delivery fidelity and contamination.[97] Finally, efforts to minimise exclusion based on English

For peer review only - http://bmjopen.bmj.com/site/about/guidelines.xhtml

language and age resulted in a diverse cohort (40% Asian; age range 19–82), enhancing external validity by better reflecting populations attending NHS musculoskeletal services.[29]

The target sample size was not reached, which limits the precision of feasibility estimates and means progression criteria and subgroup patterns should be interpreted cautiously. The 2:1 allocation ratio increased intervention exposure and feedback but resulted in fewer usual exercise care participants, reducing the precision of usual-care estimates and limiting interpretation of between-group descriptive patterns. Participants and outcome assessors were not blinded, which may have introduced performance or detection bias, particularly for self-reported outcomes; blinding of outcome assessment will therefore be incorporated into the definitive trial where feasible. Physiotherapists delivered the EPIC-Neck programme to relatively few patients; therefore, some fidelity issues may be overestimated and could improve with increased exposure and practice.[98 99] Fidelity assessments were undertaken unblinded, which may have influenced scoring despite use of a structured competence checklist. In addition, interviews were conducted by the intervention developer, whose role in EPIC-Neck development may have introduced social desirability and allegiance bias. The use of topic guides, reflective discussions and team-based interpretation helped mitigate this risk, but independent interviewing and analysis would strengthen future evaluations.

CONCLUSION

The EPIC-Neck programme shows potential for evaluation in a definitive trial, but progression is conditional on further refinement. Recruitment and contamination findings were encouraging, and the intervention was broadly acceptable and distinct

For peer review only - http://bmjopen.bmj.com/site/about/guidelines.xhtml

from usual exercise care. However, retention, delivery fidelity and intervention coherence require optimisation before definitive evaluation. Future work should refine the EPIC-Neck programme, training package and trial retention procedures, followed by targeted repeat testing of delivery fidelity, participant and physiotherapist understanding of the intervention and retention before progressing to a full-scale definitive trial.

For peer review only

For peer review only - http://bmjopen.bmj.com/site/about/guidelines.xhtml

LIST OF ABBREVIATIONS

AQoL. Assessment of Quality-of-Life

CI. Confidence Interval

DNA. Did Not Attend

EPIC. Exercise Prescription Improved through Co-design

HADS. Hospital Anxiety and Depression Scale

IMD. Index of Multiple Deprivation

NDI. Neck disability index

NHS. National Health Service

NIH-BCC. National Institutes of Health-Behaviour Change Consortium

PIL. Participant Information Leaflet

PO. Performance Objectives

PPIE. Patient and Public Involvement and Engagement

PSEQ. Pain self-efficacy questionnaire

PSFS. Patient Specific Functional Scale

SCI. Sleep condition indicator

SD. Standard Deviation

SOPA. Survey of Pain Attitudes Questionnaire

For peer review only

For peer review only - http://bmjopen.bmj.com/site/about/guidelines.xhtml

TFA. Theoretical Framework of Acceptability

TSK. Tampa Scale of Kinesiophobia

UEC. Usual Exercise Care

VAS. Visual analogue scale

DECLARATIONS

Ethics approval and consent to participate

The study was registered with ISRCTN81746901 and received ethical approval from South Central - Oxford B Research Ethics Committee (24/SC/0006). Patient participants and physiotherapists gave informed consent to participate in the study before taking part.

Participant consent for publication

Not applicable.

Availability of data and materials

Data are available upon reasonable request. Data sharing requests can be considered via contact with the corresponding author.

Competing interests

The authors declare that they have no competing interests

For peer review only

For peer review only - http://bmjopen.bmj.com/site/about/guidelines.xhtml

Funding

646
647 Jonathan Price, Clinical Doctoral Research Fellow, NIHR302141 is funded by HEE/NIHR for this research project. The views expressed in this publication are those of the author(s) and not necessarily those of the NIHR, NHS or the UK Department of Health and Social Care.

The funders are not involved in trial design, data collection, management, analysis, writing of the report or the decision to submit this report for publication.

The study sponsor is Birmingham Community Healthcare NHS Foundation Trust (BCHC.researchinnovation@nhs.net).

Author Contributions

JP was the chief investigator and conceived and designed the study, was awarded the funding and drafted the manuscript. AR, NI, CG, PP contributed to the study design, provided specific content and edited the trial protocol and manuscript. MB contributed to data analysis. All authors have reviewed and approved the manuscript.

Collaborators

EPIC-Neck feasibility trial collaborators: Apurba Subedi (Birmingham Community Healthcare NHS Foundation Trust), Hayley Alderton (Berkshire Healthcare NHS Foundation Trust), Katherine Yeung (Birmingham Community Healthcare NHS Foundation Trust), Longsun Yau (Birmingham Community Healthcare NHS Foundation Trust), Paul Matthews (Nottingham University Hospitals NHS Trust), Rajvinder Shokkar (Berkshire Healthcare NHS Foundation Trust)

For peer review only - http://bmjopen.bmj.com/site/about/guidelines.xhtml

668 **Acknowledgements**

669 We thank the EPIC-Neck patient and public involvement and engagement group for
670 their contributions.

671 **Author details**

672 [1]Musculoskeletal Physiotherapy Services, Birmingham Community Healthcare NHS
673 Foundation Trust, Birmingham, United Kingdom

674 [2]Research and Innovation, Birmingham Community Healthcare NHS Foundation
675 Trust, Birmingham, United Kingdom

676 [3]School of Sport, Exercise and Rehabilitation Sciences, College of Life and
677 Environmental Sciences, University of Birmingham, Birmingham, United Kingdom

678 [4]School of Physical Therapy, Western University, Ontario, Canada

679 [5]Institute of Applied Health Research, College of Medical and Dental Sciences,
680 University of Birmingham, Birmingham, United Kingdom

681 *Corresponding author

682 Email: Jonathan.price2@nhs.net

683 **ORCID iDs**

684 Jonathan Price: https://orcid.org/0000-0002-5505-1564

685 Alison Rushton: https://orcid.org/0000-0001-8114-7669

686 Natalie Ives: https://orcid.org/0000-0002-1664-7541

For peer review only

Marilyn Bradbury: https://orcid.org/0000-0001-6000-7229

Priti Parmar: https://orcid.org/0000-0003-4344-144X

Colin J Greaves: https://orcid.org/0000-0003-4425-2691

FIGURES

Figure 1 – CONSORT flow diagram. Abbreviations: CNSNP – Chronic non-specific neck pain; DNA – Did not attend; PIL – Participant information leaflet

SUPPLEMENTARY FILES

Supplementary file 1 – CONSORT for pilot and feasibility trials

Supplementary file 2 – Feasibility trial protocol ethics

Supplementary file 3 – Supporting information

Supplementary file 4 – GRIPP2-SF

Supplementary file 5 – Acceptability data

For peer review only

For peer review only - http://bmjopen.bmj.com/site/about/guidelines.xhtml

REFERENCES

1. Xu F, Zhang X, Yang M, et al. Magnitude, temporal trend and inequality in burden of neck pain: an analysis of the Global Burden of Disease Study 2019. *BMC Musculoskelet Disord* 2025;26(1):202. doi: 10.1186/s12891-025-08342-3 [published Online First: 20250227]

2. Myhrvold BL, Irgens P, Robinson HS, et al. Visual trajectory pattern as prognostic factors for neck pain. *Eur J Pain* 2020;24(9):1752-64. doi: 10.1002/ejp.1622 [published Online First: 20200804]

3. Horn ME, Fritz JM. Timing of physical therapy consultation on 1-year healthcare utilization and costs in patients seeking care for neck pain: a retrospective cohort. *BMC Health Serv Res* 2018;18(1):887. doi: 10.1186/s12913-018-3699-0 [published Online First: 20181126]

4. Hey HWD, Lim JXY, Ong JZ, et al. Epidemiology of Neck Pain and Its Impact on Quality-of-Life-A Population-Based, Cross Sectional Study in Singapore. *Spine (Phila Pa 1976)* 2021;46(22):1572-80. doi: 10.1097/BRS.0000000000004071

5. Irgens P, Myhrvold BL, Kongsted A, et al. The clinical course of neck pain: Are trajectory patterns stable over a 1-year period? *Eur J Pain* 2022;26(2):531-42. doi: 10.1002/ejp.1879 [published Online First: 20211103]

6. Hallman DM, Holtermann A, Dencker-Larsen S, et al. Are trajectories of neck-shoulder pain associated with sick leave and work ability in workers? A 1-year prospective study. *BMJ Open* 2019;9(3):e022006. doi: 10.1136/bmjopen-2018-022006 [published Online First: 20190320]

7. Carroll LJ, Hogg-Johnson S, van der Velde G, et al. Course and prognostic factors for neck pain in the general population: results of the Bone and Joint Decade 2000-2010 Task Force on Neck Pain and Its Associated Disorders. *Spine (Phila Pa 1976)* 2008;33(4 Suppl):S75-82. doi: 10.1097/BRS.0b013e31816445be

8. National Institute for Health and Care Excellence. Chronic pain (primary and secondary) in over 16s: assessment of all chronic pain and management of chronic primary pain [Internet]. London: NICE; 2021 [Available from: https://www.nice.org.uk/guidance/ng193.

9. Bier JD, Scholten-Peeters WGM, Staal JB, et al. Clinical Practice Guideline for Physical Therapy Assessment and Treatment in Patients With Nonspecific Neck Pain. *Phys Ther* 2018;98(3):162-71. doi: 10.1093/ptj/pzx118

10. Blanpied PR, Gross AR, Elliott JM, et al. Neck Pain: Revision 2017. *J Orthop Sports Phys Ther* 2017;47(7):A1-A83. doi: 10.2519/jospt.2017.0302

11. Wilhelm MP, Donaldson M, Griswold D, et al. The Effects of Exercise Dosage on Neck-Related Pain and Disability: A Systematic Review With Meta-analysis. *J Orthop Sports Phys Ther* 2020;50(11):607-21. doi: 10.2519/jospt.2020.9155

12. de Zoete RMJ, Brown L, Oliveira K, et al. The effectiveness of general physical exercise for individuals with chronic neck pain: a systematic review of randomised controlled trials. *Eur J Physiother* 2020;22(3):141-47. doi: 10.1080/21679169.2018.1561942

For peer review only - http://bmjopen.bmj.com/site/about/guidelines.xhtml

13. Garzonio S, Arbasetti C, Geri T, et al. Effectiveness of Specific Exercise for Deep Cervical Muscles in Nonspecific Neck Pain: A Systematic Review and Meta-Analysis. *Phys Ther* 2022;102(5):pzac001. doi: 10.1093/ptj/pzac001

14. Martin-Gomez C, Sestelo-Diaz R, Carrillo-Sanjuan V, et al. Motor control using cranio-cervical flexion exercises versus other treatments for non-specific chronic neck pain: A systematic review and meta-analysis. *Musculoskelet Sci Pract* 2019;42:52-59. doi: 10.1016/j.msksp.2019.04.010 [published Online First: 20190420]

15. Li Y, Li S, Jiang J, et al. Effects of yoga on patients with chronic nonspecific neck pain: A PRISMA systematic review and meta-analysis. *Medicine (Baltimore)* 2019;98(8):e14649. doi: 10.1097/MD.0000000000014649

16. Martini JD, Ferreira GE, Xavier de Araujo F. Pilates for neck pain: A systematic review and meta-analysis of randomised controlled trials. *J Bodyw Mov Ther* 2022;31:37-44. doi: 10.1016/j.jbmt.2022.03.011 [published Online First: 20220320]

17. Price J, Rushton A, Tyros V, et al. Expert consensus on the important chronic non-specific neck pain motor control and segmental exercise and dosage variables: An international e-Delphi study. *PLoS One* 2021;16(7):e0253523. doi: 10.1371/journal.pone.0253523 [published Online First: 20210701]

18. McLean SM, Klaber Moffett JA, Sharp DM, et al. A randomised controlled trial comparing graded exercise treatment and usual physiotherapy for patients with non-specific neck pain (the GET UP neck pain trial). *Man Ther* 2013;18(3):199-205. doi: 10.1016/j.math.2012.09.005 [published Online First: 20121022]

19. Villanueva-Ruiz I, Falla D, Saez M, et al. Manual therapy and neck-specific exercise are equally effective for treating non-specific neck pain but only when exercise adherence is maximised: A randomised controlled trial. *Musculoskelet Sci Pract* 2025;77:103319. doi: 10.1016/j.msksp.2025.103319 [published Online First: 20250321]

20. Dalager T, Welch A, O'Leary SP, et al. Clinically Relevant Decreases in Neck/Shoulder Pain Among Office Workers Are Associated With Strength Training Adherence and Exercise Compliance: Explorative Analyses From a Randomized Controlled Trial. *Phys Ther* 2023;103(2):pzac166. doi: 10.1093/ptj/pzac166

21. Zebis MK, Andersen CH, Sundstrup E, et al. Time-wise change in neck pain in response to rehabilitation with specific resistance training: implications for exercise prescription. *PLoS One* 2014;9(4):e93867. doi: 10.1371/journal.pone.0093867 [published Online First: 20140407]

22. Price J, Rushton A, Ives N, et al. Co-development of an evidence-informed, theoretically driven exercise programme for people with chronic non-specific neck pain (the EPIC-Neck programme - "Exercise Prescription Improved through Co-design"). *BMC Musculoskelet Disord* 2025;26(1):689. doi: 10.1186/s12891-025-08918-z [published Online First: 20250718]

23. Skivington K, Matthews L, Simpson SA, et al. A new framework for developing and evaluating complex interventions: update of Medical Research Council guidance. *BMJ* 2021;374:n2061. doi: 10.1136/bmj.n2061 [published Online First: 20210930]

For peer review only - http://bmjopen.bmj.com/site/about/guidelines.xhtml

24. Eldridge SM, Chan CL, Campbell MJ, et al. CONSORT 2010 statement: extension to randomised pilot and feasibility trials. *BMJ* 2016;355:i5239. doi: 10.1136/bmj.i5239 [published Online First: 20161024]

25. Price J, Rushton A, Ives N, et al. Co-designed neck exercise (EPIC-Neck) vs usual exercise care for people with chronic non-specific neck pain: protocol for a randomised feasibility study with process evaluation. *Pilot Feasibility Stud* 2025;11(1):26. doi: 10.1186/s40814-025-01608-6 [published Online First: 20250308]

26. National Institute for Health and Care Excellence. Neck pain - non-specific: Clinical Knowledge Summaries 2023 [Available from: https://cks.nice.org.uk/topics/neck-pain-non-specific/.

27. Tsakitzidis G, Remmen R, Dankaerts W, et al. Non-specific neck pain and evidence-based practice. *European scientific journal* 2013;9(3):1-19.

28. NHS England. Increasing diversity in research participation: A good practice guide for engaging with underrepresented groups [Internet]. London: NHS England; 2023 [Available from: https://www.england.nhs.uk/aac/publication/increasing-diversity-in-research-participation/.

29. National Institute for Health and Care Research. Improving inclusion of under-served groups in clinical research: Guidance from INCLUDE Project [Internet]. London: NIHR; 2022 [Available from: https://www.nihr.ac.uk/documents/improving-inclusion-of-under-served-groups-in-clinical-research-guidance-from-include-project/25435.

30. Thabane L, Lancaster G. A guide to the reporting of protocols of pilot and feasibility trials. *Pilot Feasibility Stud* 2019;5(1):37. doi: 10.1186/s40814-019-0423-8 [published Online First: 20190228]

31. Miller WR, Rollnick S. Motivational Interviewing, Second Edition: Preparing People for Change. 2nd ed. New York, NY: Guilford Publications 2002.

32. Elwyn G, Durand MA, Song J, et al. A three-talk model for shared decision making: multistage consultation process. *BMJ* 2017;359:j4891. doi: 10.1136/bmj.j4891 [published Online First: 20171106]

33. To D, De Carvalho D, Pike A, et al. A qualitative study exploring perceived barriers and enablers to fidelity of training and delivery for an intervention to reduce non-indicated imaging for low back pain. *Chiropr Man Therap* 2023;31(1):6. doi: 10.1186/s12998-023-00480-6 [published Online First: 20230131]

34. Beneciuk JM, George SZ, Greco CM, et al. Targeted interventions to prevent transitioning from acute to chronic low back pain in high-risk patients: development and delivery of a pragmatic training course of psychologically informed physical therapy for the TARGET trial. *Trials* 2019;20(1):256. doi: 10.1186/s13063-019-3350-3 [published Online First: 20190506]

35. Craven K, Holmes J, Powers K, et al. Embedding mentoring to support trial processes and implementation fidelity in a randomised controlled trial of vocational rehabilitation for stroke survivors. *BMC Med Res Methodol* 2021;21(1):203. doi: 10.1186/s12874-021-01382-y [published Online First: 20211003]

36. Mellor K, Albury C, Dutton SJ, et al. Recommendations for progression criteria during external randomised pilot trial design, conduct, analysis and reporting. *Pilot*

For peer review only

For peer review only - http://bmjopen.bmj.com/site/about/guidelines.xhtml

Feasibility Stud 2023;9(1):59. doi: 10.1186/s40814-023-01291-5 [published Online First: 20230415]

37. Price J, Rushton A, Tyros I, et al. Effectiveness and optimal dosage of exercise training for chronic non-specific neck pain: A systematic review with a narrative synthesis. *PLoS One* 2020;15(6):e0234511. doi: 10.1371/journal.pone.0234511 [published Online First: 20200610]

38. Littlewood C, Bateman M, Brown K, et al. A self-managed single exercise programme versus usual physiotherapy treatment for rotator cuff tendinopathy: a randomised controlled trial (the SELF study). *Clin Rehabil* 2016;30(7):686-96. doi: 10.1177/0269215515593784 [published Online First: 20150709]

39. Lamb SE, Williamson EM, Heine PJ, et al. Exercises to improve function of the rheumatoid hand (SARAH): a randomised controlled trial. *Lancet* 2015;385(9966):421-9. doi: 10.1016/S0140-6736(14)60998-3 [published Online First: 20141009]

40. Hu S, Guinan E, Mockler D, et al. Retention rates and reasons for non-retention in exercise oncology trials in the post-treatment phase-a systematic review. *J Cancer Surviv* 2025;19(5):1535-43. doi: 10.1007/s11764-024-01569-4 [published Online First: 20240403]

41. Watson S, Wong C, Smith T. Factors associated with trial recruitment and retention of people with osteoarthritis: analysis of 215 randomised controlled trials from 2013-2021. *Physiother Rev* 2022;26(2):5-19. doi: 10.5114/phr.2022.117578

42. Jacques RM, Ahmed R, Harper J, et al. Recruitment, consent and retention of participants in randomised controlled trials: a review of trials published in the National Institute for Health Research (NIHR) Journals Library (1997-2020). *BMJ Open* 2022;12(2):e059230. doi: 10.1136/bmjopen-2021-059230 [published Online First: 20220214]

43. Elfeky A, Gillies K, Gardner H, et al. Non-randomised evaluations of strategies to increase participant retention in randomised controlled trials: a systematic review. *Syst Rev* 2020;9(1):224. doi: 10.1186/s13643-020-01471-x [published Online First: 20200929]

44. Higgins JPT, Green S. Cochrane Handbook for Systematic Reviews of Interventions. Chichester, UK: The Cochrane Collaboration 2011.

45. Thompson DP, Oldham JA, Woby SR. Does adding cognitive-behavioural physiotherapy to exercise improve outcome in patients with chronic neck pain? A randomised controlled trial. *Physiotherapy* 2016;102(2):170-7. doi: 10.1016/j.physio.2015.04.008 [published Online First: 20150702]

46. Frost J, Wingham J, Britten N, et al. Home-based rehabilitation for heart failure with reduced ejection fraction: mixed methods process evaluation of the REACH-HF multicentre randomised controlled trial. *BMJ Open* 2019;9(8):e026039. doi: 10.1136/bmjopen-2018-026039 [published Online First: 20190802]

47. Thompson TP, Lambert JD, Greaves CJ, et al. Intervention delivery fidelity assessment of a counseling-based intervention for promoting smoking reduction and increasing physical activity. *Health Psychol* 2018;37(7):627-37. doi: 10.1037/hea0000613 [published Online First: 20180430]

For peer review only

For peer review only - http://bmjopen.bmj.com/site/about/guidelines.xhtml

48. Dreyfus SE. The Five-Stage Model of Adult Skill Acquisition. *Bull Sci Technol Soc* 2004;24(3):177-81. doi: 10.1177/0270467604264992

49. Cross R, Greaves CJ, Withall J, et al. Delivery fidelity of the REACT (REtirement in ACTion) physical activity and behaviour maintenance intervention for community dwelling older people with mobility limitations. *BMC Public Health* 2022;22(1):1112. doi: 10.1186/s12889-022-13496-z [published Online First: 20220603]

50. Withers TM, Greaves CJ, Bown MJ, et al. A feasibility study of the CRISP intervention; a cardiovascular risk reduction intervention in patients with an abdominal aortic aneurysm. *NIHR Open Res* 2024;4:34. doi: 10.3310/nihropenres.13596.2 [published Online First: 20240903]

51. Luker JA, Craig LE, Bennett L, et al. Implementing a complex rehabilitation intervention in a stroke trial: a qualitative process evaluation of AVERT. *BMC Med Res Methodol* 2016;16:52. doi: 10.1186/s12874-016-0156-9 [published Online First: 20160510]

52. Robinson K, Allen F, Darby J, et al. Contamination in complex healthcare trials: the falls in care homes (FinCH) study experience. *BMC Med Res Methodol* 2020;20(1):46. doi: 10.1186/s12874-020-00925-z [published Online First: 20200227]

53. Sekhon M, Cartwright M, Francis JJ. Development of a theory-informed questionnaire to assess the acceptability of healthcare interventions. *BMC Health Serv Res* 2022;22(1):279. doi: 10.1186/s12913-022-07577-3 [published Online First: 20220301]

54. Sekhon M, Cartwright M, Francis JJ. Acceptability of healthcare interventions: an overview of reviews and development of a theoretical framework. *BMC Health Serv Res* 2017;17(1):88. doi: 10.1186/s12913-017-2031-8 [published Online First: 20170126]

55. Vernon H, Mior S. The Neck Disability Index: a study of reliability and validity. *J Manipulative Physiol Ther* 1991;14(7):409-15.

56. Bobos P, MacDermid JC, Walton DM, et al. Patient-Reported Outcome Measures Used for Neck Disorders: An Overview of Systematic Reviews. *J Orthop Sports Phys Ther* 2018;48(10):775-88. doi: 10.2519/jospt.2018.8131 [published Online First: 20180622]

57. Modarresi S, Lukacs MJ, Ghodrati M, et al. A Systematic Review and Synthesis of Psychometric Properties of the Numeric Pain Rating Scale and the Visual Analog Scale for Use in People With Neck Pain. *Clin J Pain* 2021;38(2):132-48. doi: 10.1097/AJP.0000000000000999 [published Online First: 20211026]

58. Feng YS, Kohlmann T, Janssen MF, et al. Psychometric properties of the EQ-5D-5L: a systematic review of the literature. *Qual Life Res* 2021;30(3):647-73. doi: 10.1007/s11136-020-02688-y [published Online First: 20201207]

59. Zigmond AS, Snaith RP. The hospital anxiety and depression scale. *Acta Psychiatr Scand* 1983;67(6):361-70. doi: 10.1111/j.1600-0447.1983.tb09716.x

60. Espie CA, Kyle SD, Hames P, et al. The Sleep Condition Indicator: a clinical screening tool to evaluate insomnia disorder. *BMJ Open* 2014;4(3):e004183. doi: 10.1136/bmjopen-2013-004183 [published Online First: 20140318]

For peer review only - http://bmjopen.bmj.com/site/about/guidelines.xhtml

61. Stratford P, Gill C, Westaway M, et al. Assessing disability and change on individual patients: a report of a patient specific measure. *Physiother Can* 1995;47(4):258-63.

62. Maxwell A, Ozmen M, Iezzi A, et al. Deriving population norms for the AQoL-6D and AQoL-8D multi-attribute utility instruments from web-based data. *Qual Life Res* 2016;25(12):3209-19. doi: 10.1007/s11136-016-1337-z [published Online First: 20160625]

63. Richardson J, Iezzi A, Khan MA, et al. Validity and reliability of the Assessment of Quality of Life (AQoL)-8D multi-attribute utility instrument. *Patient* 2014;7(1):85-96. doi: 10.1007/s40271-013-0036-x

64. Jensen MP, Keefe FJ, Lefebvre JC, et al. One- and two-item measures of pain beliefs and coping strategies. *Pain* 2003;104(3):453-69. doi: 10.1016/S0304-3959(03)00076-9

65. Dupuis F, Cherif A, Batcho C, et al. The Tampa Scale of Kinesiophobia: A Systematic Review of Its Psychometric Properties in People With Musculoskeletal Pain. *Clin J Pain* 2023;39(5):236-47. doi: 10.1097/AJP.0000000000001104 [published Online First: 20230501]

66. Nicholas MK. The pain self-efficacy questionnaire: Taking pain into account. *Eur J Pain* 2007;11(2):153-63. doi: 10.1016/j.ejpain.2005.12.008

67. Withers TM, Garner NJ, Thorley CS, et al. Intervention fidelity assessment: A sub-study of the Norfolk Diabetes Prevention Study (NDPS). *Br J Health Psychol* 2023;28(3):740-52. doi: 10.1111/bjhp.12651 [published Online First: 20230212]

68. Thabane L, Ma J, Chu R, et al. A tutorial on pilot studies: the what, why and how. *BMC Med Res Methodol* 2010;10(1):1. doi: 10.1186/1471-2288-10-1 [published Online First: 20100106]

69. Gale NK, Heath G, Cameron E, et al. Using the framework method for the analysis of qualitative data in multi-disciplinary health research. *BMC Med Res Methodol* 2013;13:117. doi: 10.1186/1471-2288-13-117 [published Online First: 20130918]

70. Klingberg S, Stalmeijer RE, Varpio L. Using framework analysis methods for qualitative research: AMEE Guide No. 164. *Med Teach* 2024;46(5):603-10. doi: 10.1080/0142159X.2023.2259073 [published Online First: 20230921]

71. Teare MD, Dimairo M, Shephard N, et al. Sample size requirements to estimate key design parameters from external pilot randomised controlled trials: a simulation study. *Trials* 2014;15(1):264. doi: 10.1186/1745-6215-15-264 [published Online First: 20140703]

72. Public Health England. Chronic pain in adults [Internet]. London: Public Health England; 2017 [Available from: https://assets.publishing.service.gov.uk/media/5fc8c6b78fa8f547585ed7f3/Chronic_Pain_Report.pdf.

73. Adebajo A, Shikoh S, Kumar K, et al. Ethnic minority musculoskeletal health. *Rheumatology (Oxford)* 2018;57(2):201-03. doi: 10.1093/rheumatology/kex006 [published Online First: 2017/02/17]

For peer review only

For peer review only - http://bmjopen.bmj.com/site/about/guidelines.xhtml

74. Versus Arthritis. Chronic pain in England: Unseen, unequal, unfair [Internet]. London: Versus Arthritis; 2021 [Available from: https://www.versusarthritis.org/about-arthritis/data-and-statistics/chronic-pain-in-england/.

75. Staniszewska S, Brett J, Simera I, et al. GRIPP2 reporting checklists: tools to improve reporting of patient and public involvement in research. *Res Involv Engagem* 2017;3(1):13. doi: 10.1186/s40900-017-0062-2 [published Online First: 20170802]

76. Gillies K, Kearney A, Keenan C, et al. Strategies to improve retention in randomised trials. *Cochrane Database Syst Rev* 2021(3):MR000032. doi: 10.1002/14651858.MR000032.pub3 [published Online First: 20210306]

77. Brueton V, Stenning SP, Stevenson F, et al. Best practice guidance for the use of strategies to improve retention in randomized trials developed from two consensus workshops. *J Clin Epidemiol* 2017;88:122-32. doi: 10.1016/j.jclinepi.2017.05.010 [published Online First: 20170522]

78. Elfeky A, Treweek S, Hannes K, et al. Using qualitative methods in pilot and feasibility trials to inform recruitment and retention processes in full-scale randomised trials: a qualitative evidence synthesis. *BMJ Open* 2022;12(4):e055521. doi: 10.1136/bmjopen-2021-055521 [published Online First: 20220418]

79. Hrobjartsson A, Emanuelsson F, Skou Thomsen AS, et al. Bias due to lack of patient blinding in clinical trials. A systematic review of trials randomizing patients to blind and nonblind sub-studies. *Int J Epidemiol* 2014;43(4):1272-83. doi: 10.1093/ije/dyu115 [published Online First: 20140530]

80. Armijo-Olivo S, Fuentes J, da Costa BR, et al. Blinding in Physical Therapy Trials and Its Association with Treatment Effects: A Meta-epidemiological Study. *Am J Phys Med Rehabil* 2017;96(1):34-44. doi: 10.1097/PHM.0000000000000521

81. Cook CE, Thigpen CA. Five good reasons to be disappointed with randomized trials. *J Man Manip Ther* 2019;27(2):63-65. doi: 10.1080/10669817.2019.1589697 [published Online First: 20190314]

82. Carroll C, Patterson M, Wood S, et al. A conceptual framework for implementation fidelity. *Implement Sci* 2007;2:40. doi: 10.1186/1748-5908-2-40 [published Online First: 20071130]

83. Toomey E, Hardeman W, Hankonen N, et al. Focusing on fidelity: narrative review and recommendations for improving intervention fidelity within trials of health behaviour change interventions. *Health Psychol Behav Med* 2020;8(1):132-51. doi: 10.1080/21642850.2020.1738935 [published Online First: 20200312]

84. Borrelli B. The Assessment, Monitoring, and Enhancement of Treatment Fidelity In Public Health Clinical Trials. *J Public Health Dent* 2011;71(Suppl 1):S52-63. doi: 10.1111/j.1752-7325.2011.00233.x

85. Dalal HM, Taylor RS, Jolly K, et al. The effects and costs of home-based rehabilitation for heart failure with reduced ejection fraction: The REACH-HF multicentre randomized controlled trial. *Eur J Prev Cardiol* 2019;26(3):262-72. doi: 10.1177/2047487318806358 [published Online First: 20181010]

For peer review only

For peer review only - http://bmjopen.bmj.com/site/about/guidelines.xhtml

86. Stathi A, Withall J, Greaves CJ, et al. A group-based exercise and behavioural maintenance intervention for adults over 65 years with mobility limitations: the REACT RCT. *Public Health Research* 2022;10(14):1-172. doi: 10.3310/mqbw6832

87. Kunstler BE, Cook JL, Freene N, et al. Physiotherapists use a small number of behaviour change techniques when promoting physical activity: A systematic review comparing experimental and observational studies. *J Sci Med Sport* 2018;21(6):609-15. doi: 10.1016/j.jsams.2017.10.027 [published Online First: 20171207]

88. Magill N, Knight R, McCrone P, et al. A scoping review of the problems and solutions associated with contamination in trials of complex interventions in mental health. *BMC Med Res Methodol* 2019;19(1):4. doi: 10.1186/s12874-018-0646-z [published Online First: 20190107]

89. Gross A, Kay TM, Paquin JP, et al. Exercises for mechanical neck disorders. *Cochrane Database Syst Rev* 2015(1):CD004250. doi: 10.1002/14651858.CD004250.pub5 [published Online First: 20150128]

90. Hemming K, Taljaard M, Moerbeek M, et al. Contamination: How much can an individually randomized trial tolerate? *Stat Med* 2021;40(14):3329-51. doi: 10.1002/sim.8958 [published Online First: 20210507]

91. Simpson P, Holopainen R, Schutze R, et al. Becoming confidently competent: a qualitative investigation of training in cognitive functional therapy for persistent low back pain. *Physiother Theory Pract* 2024;40(4):804-16. doi: 10.1080/09593985.2022.2151333 [published Online First: 20221124]

92. Lawford BJ, Delany C, Bennell KL, et al. Training Physical Therapists in Person-Centered Practice for People With Osteoarthritis: A Qualitative Case Study. *Arthritis Care Res (Hoboken)* 2018;70(4):558-70. doi: 10.1002/acr.23314 [published Online First: 20180226]

93. Simpson P, Holopainen R, Schutze R, et al. Training of Physical Therapists to Deliver Individualized Biopsychosocial Interventions to Treat Musculoskeletal Pain Conditions: A Scoping Review. *Phys Ther* 2021;101(10):pzab188. doi: 10.1093/ptj/pzab188

94. Hatfield TG, Withers TM, Greaves CJ. Systematic review of the effect of training interventions on the skills of health professionals in promoting health behaviour, with meta-analysis of subsequent effects on patient health behaviours. *BMC Health Serv Res* 2020;20(1):593. doi: 10.1186/s12913-020-05420-1 [published Online First: 20200629]

95. Newton C, Singh G, Nolan D, et al. Cognitive Functional Therapy compared with usual physiotherapy care in people with persistent low back pain: a mixed methods feasibility randomised controlled trial in the United Kingdom National Health Service. *Physiotherapy* 2024;123:118-32. doi: 10.1016/j.physio.2024.02.003 [published Online First: 20240212]

96. Shersher V, Haines TP, Sturgiss L, et al. Definitions and use of the teach-back method in healthcare consultations with patients: A systematic review and thematic synthesis. *Patient Educ Couns* 2021;104(1):118-29. doi: 10.1016/j.pec.2020.07.026 [published Online First: 20200801]

For peer review only - http://bmjopen.bmj.com/site/about/guidelines.xhtml

1035 97. Toomey E, Matthews J, Guerin S, et al. Development of a Feasible Implementation
1036 Fidelity Protocol Within a Complex Physical Therapy-Led Self-Management
1037 Intervention. *Phys Ther* 2016;96(8):1287-98. doi: 10.2522/ptj.20150446 [published
1038 Online First: 20160303]

1039 98. Rathod T, Belcher J, Montgomery AA, et al. Health services changes: is a run-in period
1040 necessary before evaluation in randomised clinical trials? *Trials* 2014;15(1):41. doi:
1041 10.1186/1745-6215-15-41 [published Online First: 20140130]

1042 99. Khan N, Abboudi H, Khan MS, et al. Measuring the surgical 'learning curve': methods,
1043 variables and competency. *BJU Int* 2014;113(3):504-8. doi: 10.1111/bju.12197
1044 [published Online First: 20130702]

1045

1046

1047

1048

1049

1050

1051

1052

1053

1054

1055

1056

1057

1058

For peer review only

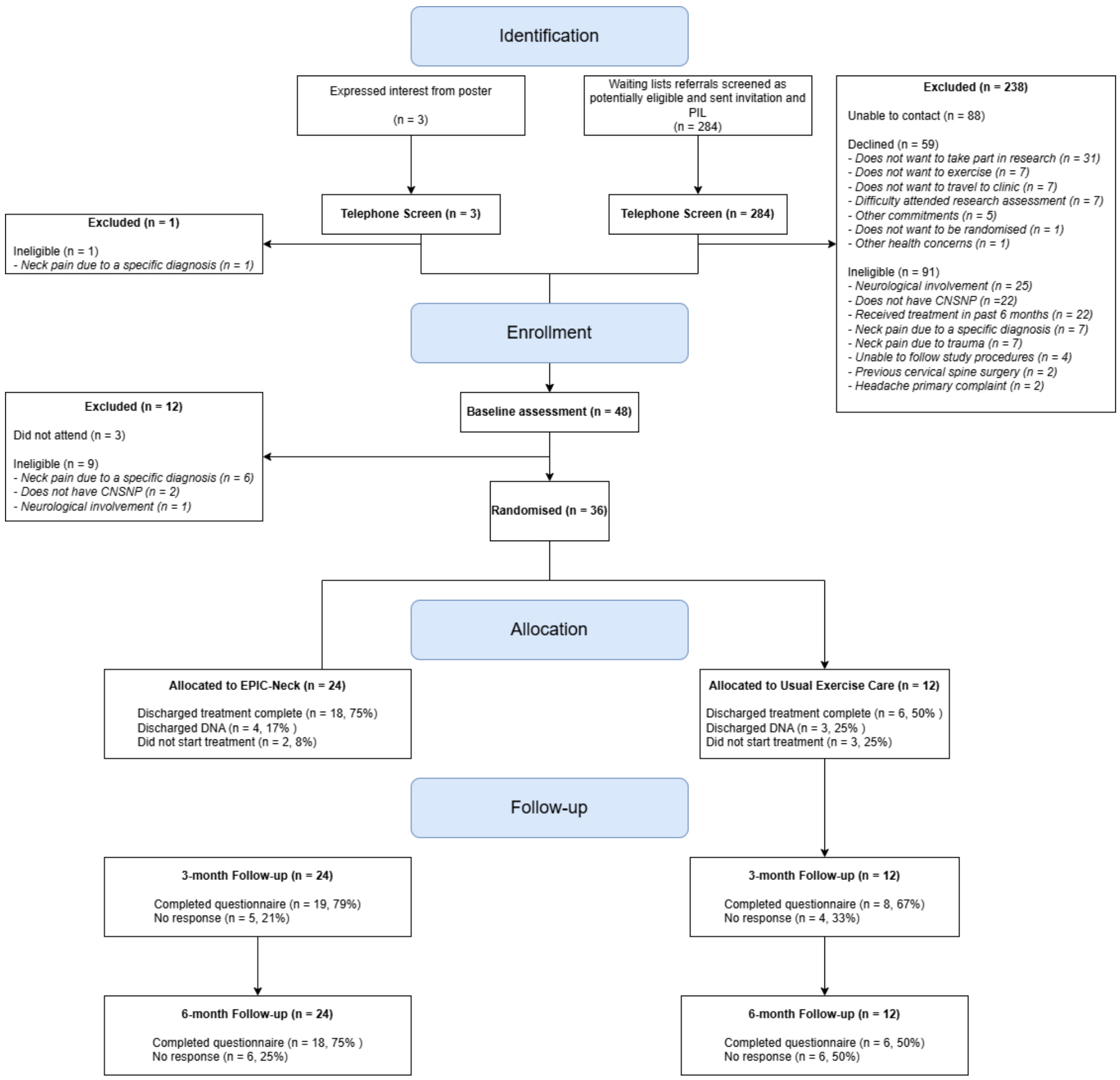

396x381mm (72 x 72 DPI)

BMJ Open

Co-developed neck exercise (EPIC-Neck) versus usual exercise care for people with chronic non-specific neck pain: a UK multicentre randomised feasibility trial

Journal:	*BMJ Open*
Manuscript ID	bmjopen-2026-120571.R2
Article Type:	Original research
Date Submitted by the Author:	11-Aug-2026
Complete List of Authors:	Price, Jonathan; Birmingham Community Healthcare NHS Foundation Trust, Musculoskeletal Physiotherapy Services; Birmingham Community Healthcare NHS Foundation Trust, Research and Innovation; University of Birmingham College of Life and Environmental Sciences, School of Sport, Exercise and Rehabilitation Sciences<br>Rushton, Alison; Western University, School of Physical Therapy<br>Ives, Natalie; University of Birmingham College of Medical and Dental Sciences, Institute of Applied Health Research<br>Bradbury, Marilyn; Birmingham Community Healthcare NHS Foundation Trust, Research and Innovation; University of Birmingham College of Life and Environmental Sciences, School of Sport, Exercise and Rehabilitation Sciences<br>Parmar, Priti ; Birmingham Community Healthcare NHS Foundation Trust, Research and Innovation<br>Greaves, Colin; University of Birmingham College of Life and Environmental Sciences, School of Sport, Exercise and Rehabilitation Sciences
**Primary Subject Heading**:	Rehabilitation medicine
Secondary Subject Heading:	Rehabilitation medicine
Keywords:	Exercise, Self-Management, Feasibility Studies, Behavior, Spine < ORTHOPAEDIC & TRAUMA SURGERY

SCHOLARONE MANUSCRIPTS

I, the Submitting Author has the right to grant and does grant on behalf of all authors of the Work (as defined in the below author licence), an exclusive licence and/or a non-exclusive licence for contributions from authors who are: i) UK Crown employees; ii) where BMJ has agreed a CC-BY licence shall apply, and/or iii) in accordance with the terms applicable for US Federal Government officers or employees acting as part of their official duties; on a worldwide, perpetual, irrevocable, royalty-free basis to BMJ Publishing Group Ltd ("BMJ") its licensees and where the relevant Journal is co-owned by BMJ to the co-owners of the Journal, to publish the Work in this journal and any other BMJ products and to exploit all rights, as set out in our licence.

The Submitting Author accepts and understands that any supply made under these terms is made by BMJ to the Submitting Author unless you are acting as an employee on behalf of your employer or a postgraduate student of an affiliated institution which is paying any applicable article publishing charge ("APC") for Open Access articles. Where the Submitting Author wishes to make the Work available on an Open Access basis (and intends to pay the relevant APC), the terms of reuse of such Open Access shall be governed by a Creative Commons licence – details of these licences and which Creative Commons licence will apply to this Work are set out in our licence referred to above.

Other than as permitted in any relevant BMJ Author's Self Archiving Policies, I confirm this Work has not been accepted for publication elsewhere, is not being considered for publication elsewhere and does not duplicate material already published. I confirm all authors consent to publication of this Work and authorise the granting of this licence.

For peer review only

TITLE PAGE

Co-developed neck exercise (EPIC-Neck) versus usual exercise care for people with chronic non-specific neck pain: a UK multicentre randomised feasibility trial

Internal Reference/Short Title: EPIC-Neck

IRAS number: 331102

Trial registration: ISRCTN81746901

For peer review only

AUTHORS

Jonathan Price[1,2,3*] - Jonathan.price2@nhs.net

Alison Rushton[4] - arushto3@uwo.ca

Natalie Ives[5] - n.j.ives@bham.ac.uk

Marilyn Bradbury[2,3] - marilyn.bradbury1@nhs.net

Priti Parmar[2] - priti.parmar2@nhs.net

Colin J Greaves[3] - c.j.greaves@bham.ac.uk

ABSTRACT

Objectives: To assess the feasibility of conducting a definitive randomised controlled trial of the EPIC-Neck programme versus usual exercise care for chronic non-specific neck pain, specifically in relation to recruitment, retention, delivery fidelity, acceptability and contamination.

For peer review only - http://bmjopen.bmj.com/site/about/guidelines.xhtml

Design: Multicentre, two-arm, parallel-group, individually randomised controlled feasibility trial with an embedded process evaluation.

Setting: Three National Health Service musculoskeletal services in England.

Participants: Adults (≥18 years) with chronic non-specific neck pain lasting >3 months.

Interventions: Participants were randomised (2:1) to EPIC-Neck (an individualised, evidence-based, theory-informed, co-designed exercise programme) or usual exercise care. Participants and outcome assessors were not blinded to allocation.

Feasibility objectives: Progression was assessed using predefined green–amber–red criteria across five domains: (1) recruitment rate; (2) retention rate (percentage returning the neck disability index at 6 months); (3) delivery fidelity (audio-recorded appointments scored on a 0-5 Dreyfus competence scale where ≥3 = acceptable); (4) acceptability to participants and physiotherapists (1-5 acceptability questionnaires where ≥4 = acceptable and interviews analysed deductively using framework analysis against the theoretical framework of acceptability); and (5) contamination (presence of EPIC-Neck components in usual care) scored using the same 0-5 Dreyfus competency scale as delivery fidelity.

Results: Between May 2024 and February 2025, 287 individuals were screened and 36 randomised (24 EPIC-Neck, 12 usual care), yielding a mean recruitment rate of 4.5 participants/month (green). Retention at 6 months was 67% (24/36) (red). Among participants who completed their allocated treatment, 6-month retention was 91% (22/24). Delivery fidelity was moderate (mean 2.4/5; amber), with only 14% of participants receiving EPIC-Neck at an acceptable level. Acceptability was generally

For peer review only

For peer review only - http://bmjopen.bmj.com/site/about/guidelines.xhtml

positive though interviews identified addressable concerns around intervention coherence (amber). Contamination was low (mean fidelity score for controls was 1.1/5; green).

Conclusions: EPIC-Neck shows potential for evaluation in a definitive trial, but progression is conditional on future refinement of the intervention and study procedures to address the key barriers to progression: retention, delivery fidelity and intervention coherence.

Trial registrations: ISRCTN81746901

Keywords: Neck Pain, Exercise Therapy, Self-Management, Feasibility Studies, Patient Acceptance of Health Care, Intervention Fidelity

Strengths and limitations

- This multicentre randomised feasibility trial used an embedded mixed-methods process evaluation, allowing integration of quantitative and qualitative data from participants and physiotherapists.
- The high proportion of audio-recorded appointments enabled a robust assessment of delivery fidelity and contamination using a structured scoring framework.
- Inclusive recruitment and consent procedures, including multilingual/video materials and interpreter support, were used to reduce barriers to participation.
- Physiotherapists delivered the intervention to relatively few participants, so some fidelity limitations may reflect early learning effects.

For peer review only - http://bmjopen.bmj.com/site/about/guidelines.xhtml

- Fidelity assessments were unblinded, and qualitative interviews were conducted by the intervention developer, creating potential for bias despite mitigation strategies.

Word count: 4131

For peer review only

For peer review only - http://bmjopen.bmj.com/site/about/guidelines.xhtml

BACKGROUND

Neck pain continues to pose a growing global health challenge. The age-standardised number of years lived with disability for neck pain has increased in recent years, rising by 0.12% per year from 2011–2016 and 0.42% per year from 2016–2019, despite advancements in healthcare.[1] Although all types of neck pain contribute to this burden, chronic non-specific neck pain is particularly impactful because symptoms are associated with greater pain, disability, psychological distress, reduced work capacity and healthcare use than acute neck pain.[2-6] Effective treatments are needed to reduce immediate and long-term impact.

Clinical guidelines and multiple systematic reviews support the use of exercise to improve chronic non-specific neck pain and disability.[7-15] However, effects are often modest, diminish over time,[10, 16] and may be limited by poor adherence.[10, 17-20] To address these limitations, the EPIC-Neck programme (EPIC: Exercise Prescription Improved through Co-design) was co-developed with patients and physiotherapists using Intervention Mapping.[21] EPIC-Neck differs from usual exercise care by integrating physiotherapist-prescribed exercise, guided by a biopsychosocial prescription framework with structured behaviour change strategies to support exercise effects and adherence.[21]

Before evaluating adherence, clinical effectiveness and cost-effectiveness of EPIC-Neck compared with usual exercise care, key uncertainties that could compromise a definitive trial needed to be addressed.[22] The Medical Research Council recommends that complex interventions, such as EPIC-Neck, undergo feasibility testing before definitive evaluation.[22] For EPIC-Neck, key uncertainties included

For peer review only - http://bmjopen.bmj.com/site/about/guidelines.xhtml

whether adults with chronic non-specific neck pain could be recruited and retained in an exercise-versus-exercise trial, whether EPIC-Neck was acceptable to patients and physiotherapists, whether key components could be delivered as intended, and whether the intervention remained sufficiently distinct from usual exercise care to avoid contamination between trial arms.

Aims and objectives

Aim: To determine the feasibility of a definitive trial evaluating the clinical and cost-effectiveness of EPIC-Neck compared with usual exercise care for adults with chronic non-specific neck pain.

Primary feasibility objectives, aligned directly with the prespecified traffic-light progression criteria (Table 1), were to assess:

- Mean participant recruitment;
- 6-month retention;
- Delivery fidelity of key EPIC-Neck components;
- Presence of key EPIC-Neck components in usual exercise care (contamination);
- Acceptability of EPIC-Neck to patients and physiotherapists.

Secondary feasibility objectives were to:

- Describe treatment engagement, including initiation, attendance, appointment time, treatment duration and completion;
- Assess safety;
- Describe characteristics of individuals who did and did not enrol;

For peer review only

For peer review only - http://bmjopen.bmj.com/site/about/guidelines.xhtml

113 • Assess completeness of planned definitive-trial outcome measures.

For peer review only

For peer review only - http://bmjopen.bmj.com/site/about/guidelines.xhtml

Table 1 - Traffic light progression criteria

	Green	Amber	Red	Feasibility outcomes
**Mean recruitment rate**	≥3 participants per month	1-2 participants per month	≤1 participants per month	**Green:** 4.5 participants per month, 95%CI [3.0, 6.1]
**Retention rate**	≥80%	70-79%	<70%	**Red:** 67%, 95% CI [49, 81]
**Delivery fidelity**	No areas of concern (most items ≥3)	Minor problems (≥ 50% of components between 1.5–3.0)	Major problems (most components < 1.5)	**Amber:** Overall mean score 2.4 (SD 1.3, 95% CI: 1.7, 3.1); 53% of components scored 1.5–3
**Contamination**	No EPIC-Neck components present in usual care	Minor overlap	Major overlap	**Green:** Mean checklist score 1.1/5 (SD 1.0, 95%CI: 0.6, 1.6); 1/17 components observed
**Acceptability**	Mean acceptability score of ≥ 4 and no concerns in interviews	Mean acceptability score 3 and/or minor concerns	Mean acceptability score ≤2 and/or major concerns	**Amber:** Mean 3.9 (SD 1.0, 95%CI: 3.7, 4.1); interview concerns were addressable

Green: No areas of concern
Amber: Minor modifications required before continuing to a definitive trial
Red: Major modifications required before continuing to a definitive trial
Abbreviation: CI: Confidence Interval; SD: Standard Deviation

For peer review only

METHODS

Reporting

This trial is reported in accordance with the CONSORT checklist for pilot/feasibility trials (Supplementary File 1) and the published protocol (Supplementary File 2).[23, 24]

Trial design

This was a multicentre, two-arm, parallel-group, individually randomised controlled feasibility trial. Participants were randomised in a 2:1 ratio to either the intervention group (EPIC-Neck programme) or usual exercise care. The feasibility trial evaluated outcomes important for deciding whether and how to progress to a definitive trial, including recruitment, retention, treatment engagement, acceptability, contamination, outcome-measure completion and safety. Embedded within the feasibility trial was a mixed-methods process evaluation, which included a multidimensional fidelity evaluation to understand EPIC-Neck training, delivery, receipt and enactment and to inform intervention and training refinements. This manuscript reports the feasibility outcomes relevant to progression to a definitive trial; the detailed multidimensional fidelity evaluation is reported separately. The trial was registered (ISRCTN81746901) and received ethical approval from South Central - Oxford B Research Ethics Committee (24/SC/0006).

Participant eligibility, identification and consent procedures

Participants were recruited from National Health Service (NHS) community-based and secondary care musculoskeletal services. Adults (≥18 years) with chronic (≥3 months) non-specific neck pain were eligible. Non-specific neck pain was defined as

For peer review only - http://bmjopen.bmj.com/site/about/guidelines.xhtml

142 pain or discomfort in the neck and/or shoulder girdle, with or without referred arm
143 pain, for which no specific cause could be identified.[25, 26] Exclusions included recent
144 physiotherapy within 6 months, previous cervical surgery, traumatic or specific neck
145 pathology, neurological involvement, or inability to follow trial procedures. Full
146 eligibility criteria are reported in the protocol.[24]

147 NHS clinical staff identified potential participants from waiting lists or trial posters and
148 provided a Participant Information Leaflet (PIL). Potential participants completed
149 telephone eligibility screening and, if eligible, attended a face-to-face baseline
150 assessment with a research physiotherapist. Written informed consent was obtained
151 for the feasibility trial and qualitative interviews, eligibility confirmed, and baseline
152 assessments completed prior to randomisation. Trial materials were available in
153 paper, electronic, and video formats, with English or Urdu audio narration, and
154 professional interpreters supported screening and consent where required.[27, 28]

155 **Randomisation**

156 Randomisation was conducted via a secure web-based system (Sealed Envelope).
157 The sequence was computer-generated using random permuted blocks (sizes 3 and
158 6) with a 2:1 allocation to EPIC-Neck. A 2:1 allocation ratio in favour of the
159 intervention group was used to ensure sufficient intervention exposure and
160 participant feedback to inform feasibility, acceptability and intervention refinement.[29]
161 Allocation was concealed through the automated system, which released the
162 assignment only after baseline data entry by the research physiotherapist.

For peer review only - http://bmjopen.bmj.com/site/about/guidelines.xhtml

Blinding

Blinding of treating physiotherapists was not possible due to the nature of the intervention but cross-contamination was minimised by physiotherapists delivering either EPIC-Neck or usual exercise care, but not both. Participants and outcome assessors were not blinded in this feasibility trial. Blinding procedures will be considered, where appropriate, in the definitive trial.[30]

Physiotherapist consent procedures

Physiotherapists at participating sites provided written informed consent to deliver either EPIC-Neck or usual exercise care and to participate in the process evaluation (audio-recorded appointments/interviews).[24] Characteristics (age, gender, qualifications, years' experience, and postgraduate training) were collected by self-report before training and were not independently verified.

Interventions

EPIC-Neck (Intervention arm)

Full details of the EPIC-Neck programme are published elsewhere.[21] In brief, EPIC-Neck is a biopsychosocial, theory-informed intervention designed to support self-management of chronic neck pain through a home-based exercise programme. Physiotherapists prescribed exercises using a biopsychosocial framework to target participants' physical, psychological and social needs (Supplementary File 3, Figure S3-2). A behaviour change facilitation guide supported exercise engagement using techniques such as goal setting, self-monitoring, action planning, social support and self-efficacy enhancement, delivered through patient-centred communication

For peer review only - http://bmjopen.bmj.com/site/about/guidelines.xhtml

(Supplementary File 3, Figure S3-1).[31, 32] EPIC-Neck was delivered face-to-face by trained physiotherapists and supported by paper and digital patient-facing materials (workbook, exercise diary, exercise library, and friends-and-family leaflet). Non-exercise treatments (e.g. postural advice, manual therapy) were provided at the physiotherapist's discretion. Discharge was agreed collaboratively based on goals and self-management ability.

Physiotherapist training

Physiotherapists delivering EPIC-Neck completed a one-day, face-to-face training combining lectures, role play and behavioural skills practice.[33, 34] Ongoing support included a training manual, crib sheets, optional booster sessions, and peer/expert support.[33, 35] Audio-recordings of intervention delivery were used for fidelity evaluation, but real-time monitoring, feedback or delivery coaching were not provided during the trial.

Usual exercise care (Control arm)

The usual exercise care group received physiotherapy exercises as per the physiotherapists standard practice. Physiotherapists were not given any additional instruction or training. Non-exercise treatments (e.g. postural advice, manual therapy) were provided at the physiotherapist's discretion.

Outcomes

Feasibility progression criteria

Feasibility of progressing to a definitive trial was assessed across five prespecified domains: recruitment, 6-month retention, delivery fidelity, contamination, and

For peer review only - http://bmjopen.bmj.com/site/about/guidelines.xhtml

acceptability. A traffic-light system with a priori thresholds was used to guide progression decision (Table 1).[36]

Recruitment

The mean number of participants randomised per month was calculated across the active recruitment period.[37] Thresholds (Table 1) were based on the estimated time required to recruit the anticipated definitive trial sample size of 248 participants across 5 musculoskeletal services.[37]

Retention

Return of the Neck Disability Index (NDI) at 6 months was used as the retention criterion, selected as an indicator of likely follow-up at the planned 12-month primary endpoint in a definitive trial.[38, 39] Thresholds (Table 1) were informed by guidance that ≥80% retention represents low risk of attrition bias and comparable musculoskeletal trials reporting approximately 70% retention.[40-45]

Delivery fidelity

All EPIC-Neck appointments were audio-recorded and coded against a pre-defined 17-item checklist of key EPIC-Neck components (Supplementary File 3-Table S3-1).[46, 47] Each component was rated using a 0–5 Dreyfus delivery competence scale, where 0 indicated absent delivery, ≥3 acceptable delivery and 5 optimal delivery.[48] Progression thresholds (Table 1) were based on the distribution of checklist scores across components, with ≥3 used as the threshold for acceptable delivery, consistent with previous fidelity research using this approach.[47, 49, 50]

For peer review only

For peer review only - http://bmjopen.bmj.com/site/about/guidelines.xhtml

Contamination

Presence of EPIC-Neck components within usual exercise care was assessed using audio-recorded usual care appointments, coded against the same 17-item checklist and 0–5 Dreyfus competence scale used for EPIC-Neck delivery fidelity.[51, 52] Thresholds (Table 1) reflected the extent to which EPIC-Neck components appeared in usual care and whether this threatened distinction between trial arms. Final progression judgements were made through discussion within the study team (JP, MB and CG), informed by the checklist scores.

Acceptability

EPIC-Neck acceptability to participants and physiotherapists was evaluated using a convergent mixed-methods approach informed by the Theoretical Framework of Acceptability (TFA).[53, 54] EPIC-Neck participants completed 6-month questionnaires covering TFA domains of affective attitude, intervention coherence, perceived effectiveness, opportunity costs and self-efficacy. Items were rated on 1–5 Likert scales and interpreted at face value according to the questionnaire response anchors, where scores of 4–5 indicated positive acceptability, 3 indicated neutrality and 1–2 indicated negative acceptability.[53] Qualitative acceptability was explored through semi-structured interviews with participants (post-discharge) and physiotherapists (end of trial), using patient and public involvement and engagement (PPIE)-informed topic guides mapped to all seven TFA domains. Interviews were conducted by telephone, video call or face-to-face and audio-recorded. Progression criteria (Table 1) combined these quantitative thresholds with qualitative findings. Final progression judgements were made through discussion between JP and CG,

For peer review only

For peer review only - http://bmjopen.bmj.com/site/about/guidelines.xhtml

informed by questionnaire scores and qualitative findings. Any disagreements were resolved through discussion with a third study team member.

Allocated treatment engagement

Allocated treatment engagement was described using initiation, attendance, did-not-attend appointments, total appointment time, treatment duration and treatment completion. Treatment initiation was defined as attendance at ≥1 appointment, and treatment completion as collaboratively agreed discharge. Total appointment time was calculated from available audio-recordings, and treatment duration as weeks from initial assessment to final attended appointment.

For peer review only

Inclusivity

Demographic characteristics of randomised and non-randomised individuals identified from screening logs were compared. Variables included age, gender, ethnicity, first language, interpreter need and deprivation, measured using Index of Multiple Deprivation (IMD) decile. The IMD is a composite measure of area-level deprivation in England, incorporating domains including income, employment, education, health, crime, housing, and living environment, with deciles ranging from 1 (most deprived 10%) to 10 (least deprived 10%).

Feasibility of trial outcome measures

Planned definitive-trial outcome measures were collected at baseline, 3 and 6 months post-randomisation to assess data collection feasibility and completeness. Baseline data were collected face-to-face, and follow-up questionnaires were completed by post, with telephone completion and interpreter support available

For peer review only - http://bmjopen.bmj.com/site/about/guidelines.xhtml

where required. A £20 voucher was offered for returning all three questionnaires. Outcomes were selected based on existing literature, PPIE input and the EPIC-Neck logic model.[21] The planned definitive trial primary outcome was the Neck Disability Index, which is widely used with good psychometric properties and a minimum clinically important difference of 5.5 points.[55, 56] Secondary outcome measures include pain (Visual Analogue Scale (VAS));[57] health-related quality of life (EQ-5D-5L);[58] mental well-being (Hospital Anxiety and Depression Scale (HADS));[59] sleep quality (The Sleep Condition Indicator);[60] specific activity limitations (Patient Specific Functional Scale (PSFS)[61] and social/relationship impact (Relationships and Self-Worth domains of Assessment of Quality-of-Life 8D).[62, 63] Candidate mediators were pain beliefs (Single item Survey of Pain Attitudes Questionnaire (SOPA));[64] pain related fear (Tampa Scale of Kinesiophobia-13 (TSK-13));[65] pain self-efficacy (Pain self-efficacy questionnaire (PSEQ));[66] and night pain (VAS).[57]

Data analysis

Quantitative data were analysed using IBM SPSS v25 and were primarily descriptive, using means, standard deviations (SD), ranges, proportions and 95% confidence intervals (CI), as appropriate. For delivery fidelity and contamination, two researchers (JP, MB) independently coded an initial sample of audio-recordings and iteratively calibrated scoring using further recordings until ≥75% of ratings were within one point.[49, 67] Remaining recordings were coded by JP. Fidelity and contamination scores were summarised descriptively at component-, participant- and study-level. Treatment initiation, completion and discharge following non-attendance were summarised using the number randomised to each group as the denominator. Among participants attending ≥1 appointment, attendance, non-attendance, total

For peer review only - http://bmjopen.bmj.com/site/about/guidelines.xhtml

appointment time and treatment duration were summarised descriptively. Demographic characteristics of randomised and non-randomised individuals were compared using Pearson's chi-square tests ($p < 0.05$). As this was a feasibility trial, no inferential between-group comparisons were planned or undertaken for the planned definitive-trial outcome measures.[68]

Qualitative acceptability data were analysed using deductive framework analysis.[69, 70] Interviews were audio-recorded, transcribed verbatim and coded against TFA domains in NVivo 12 Plus (QSR International). Data were summarised in a domain-by-case matrix to compare patterns within and across cases for participants and physiotherapists separately. Minor (Amber), major (Red) or no (Green) acceptability concerns were identified within each domain. All interviews and qualitative analysis were completed by JP, an experienced physiotherapist and lead developer of the EPIC-Neck programme. Reflexivity was addressed by acknowledging prior professional relationships with some physiotherapists and none with participants. To support analytic rigour and consistency, a subset of domain coding was reviewed within the research team. Quantitative and qualitative acceptability findings were triangulated in a domain-level table, with domain-level acceptability ratings combined to provide an overall judgement.

Sample size

Target recruitment was 45 participants, based on a precision calculation to estimate retention with sufficient accuracy to inform a future definitive trial. Assuming 80% retention in this feasibility trial, approximately 43 participants were required to

For peer review only - http://bmjopen.bmj.com/site/about/guidelines.xhtml

estimate retention with a 95% CI of ±12%; this was rounded to 45 participants.[44, 71] No interim analyses or stopping rules were planned.

Semi-structured interviews were conducted with a purposive sample of 12 EPIC-Neck participants, sampling for variation in ethnicity, socioeconomic deprivation, and baseline neck disability to reflect potentially under-served groups and diverse pain experiences.[72-74] All physiotherapists delivering the EPIC-Neck programme were invited to be interviewed.

Patient and public involvement and engagement

The trial protocol, intervention development, outcome selection, inclusivity design, trial materials and dissemination were informed by a PPIE group (n = 17). Two PPIE members were part of the Study Management Group. PPIE contributions are reported in more detail in Supplementary File 4 (GRIPP2-SF).[75]

RESULTS

Screening and recruitment

Recruitment took place between 29 May 2024 and 10 February 2025 (Figure 1). Of 287 individuals invited, 36 were randomised (24 EPIC-Neck; 12 usual exercise care), giving an uptake rate of 12.5% (95% CI 8.7–16.4). Recruitment stopped before the planned target of 45 participants was reached because the available study time and funding period ended. At the point recruitment stopped, sufficient data had been collected to evaluate the prespecified feasibility objectives.

For peer review only - http://bmjopen.bmj.com/site/about/guidelines.xhtml

339 **Participant characteristics**

340 Participants had a mean age of 45.9 years (range 19–82), with a similar number of
341 males and females (Table 2). Overall, 41.7% were of Asian or Asian British ethnic
342 origin, and 72.2% lived in the most deprived IMD deciles.

For peer review only

For peer review only - http://bmjopen.bmj.com/site/about/guidelines.xhtml

343 **Table 2 – Patient participant baseline characteristics**

Demographic characteristic	EPIC-Neck n=24		Usual Exercise Care n=12		Full sample n=36	
	n	%	*n*	%	*n*	%
**Gender**						
Female	11	45.8%	5	41.7%	16	44.4%
Male	13	54.2%	7	58.3%	20	55.6%
**Age**						
18-30	3	12.5%	3	25.0%	6	16.7%
31-40	7	29.2%	1	8.3%	8	22.2%
41-50	3	12.5%	3	25.0%	6	16.7%
51-60	7	29.2%	3	25.0%	10	27.8%
61+	4	16.7%	2	16.7%	6	16.7%
**Ethnicity**						
**Asian or Asian British**	9	37.5%	6	50.0%	15	41.7%
Indian	2	8.3%	1	8.3%	3	8.3%
Pakistani	7	29.2%	5	41.7%	12	33.3%
**Black or Black British**	3	12.5%	2	16.7%	5	13.9%
African	1	4.2%	1	8.3%	2	5.6%
Caribbean	1	4.2%	0	0.0%	1	2.8%
Other	1	4.2%	1	8.3%	2	5.6%
**Mixed**	1	4.2%	1	8.3%	2	5.6%
White & Black Caribbean	1	4.2%	1	8.3%	2	5.6%
**White**	10	41.7%	3	25.0%	13	36.1%
British	9	37.5%	3	25.0%	12	33.3%
Irish	1	4.2%	0	0.0%	1	2.8%
**Other**	1	4.2%	0	0.0%	1	2.8%
Arab	1	4.2%	0	0.0%	1	2.8%
**IMD decile**						
Most deprived (deciles 1-3)	19	79.2%	7	58.3%	26	72.2%
Middle (deciles 4-7)	4	16.7%	5	41.7%	9	25.0%
Least deprived (deciles 8-10)	1	4.2%	0	0.0%	1	2.8%
**Marital status**						
Living with partner	6	25.0%	1	8.3%	7	19.4%
Married/civil union	13	54.2%	5	41.7%	18	50.0%
Separated/divorced	1	4.2%	1	8.3%	2	5.6%
Unmarried (never married)	4	16.7%	5	41.7%	9	25.0%
**Cohabiting status**						
Cohabiting	24	100.0%	10	83.3%	34	94.4%
Living alone	0	0.0%	2	16.7%	2	5.6%
**Neck pain location**						
Left	11	45.8%	9	75.0%	20	55.6%

For peer review only - http://bmjopen.bmj.com/site/about/guidelines.xhtml

Right	11	45.8%	6	50.0%	17	47.2%
Middle	7	29.2%	7	58.3%	14	38.9%
Base of skull	2	8.3%	1	8.3%	3	8.3%

Abbreviations: IMD - Index of multiple deprivation

Feasibility objectives

Mean recruitment rate

Mean recruitment rate was 4.5 participants per month (95% CI: 3.0, 6.1; 36 participants over 8 months), exceeding the "Green" progression criterion (Table 1).

Retention rate

At six months, 67% of participants returned the NDI questionnaire (24/36; 95% CI: 49, 81), meeting the "Red" criterion (Table 1). Retention was 75% in EPIC-Neck (18/24) and 50% in usual exercise care (6/12) (Figure 1). All 3-month non-responders were also non-responders at 6 months (9/9). Among 6-month non-responders, 41% (5/12) had not initiated treatment and 41% (5/12) were discharged for non-attendance. Retention among treatment completers was high (22/24; 91%).

Delivery fidelity

Fidelity was scored for 76/80 EPIC-Neck appointments; four were excluded due to missing recordings (n=2) or a participant developing neurological symptoms (n=2). Overall mean fidelity was 2.4/5 (SD 1.3; 95% CI: 1.7, 3.1). Component scores ranged from 0.9 to 3.5, with 6/17 components (35%) delivered acceptably (≥3) and 9/17 (53%) scoring 1.5–<3.0 (Table 3). Only 3/22 participants (14%) had a mean score ≥3 (participant means 1.3–3.2). Based on the study-level mean and component-level distribution, delivery fidelity met the Amber progression criterion (Table 1).

For peer review only

Table 3 – Delivery fidelity scores by component

	Mean Score (SD)	
**Component**	**EPIC-Neck**	**UEC**
01 - Agree outcome goals	2.0 (1.3)	0.9 (0.9)
02 - Actively involve the patient	3.1 (0.7)	1.2 (0.4)
03 - Improve motivation to perform exercise (PO1) - Enhance perceived importance and safety	2.1 (1.0)	1.4 (0.6)
04 - Improve motivation to perform exercise (PO1) - Improve self-efficacy	3.5 (0.8)	3.0 (1.1)
05 - Improve motivation to perform exercise (PO1) - Engage social support	1.8 (1.1)	0.4 (0.5)
06 - Set a plan to perform exercise (PO1)	2.3 (1.0)	1.0 (0.0)
07 - Encourage self-monitoring	3.3 (0.9)	0.0 (0.0)
08 - Review progress	2.7 (0.5)	1.6 (0.3)
09 - Support problem solving	1.3 (0.7)	0.8 (0.5)
10 - Introduce adapting and progressing (or regression) exercise (PO2)	1.6 (1.1)	0.2 (0.4)
11 - Introduce flare-up exercise (PO3)	1.5 (1.0)	0.1 (0.3)
12 - Introduce general exercise +/- secondary prevention exercise (PO4)	0.9 (0.9)	0.1 (0.3)
13 - Evaluate self-management and discharge suitability	2.0 (1.2)	0.8 (0.9)
14 - Identify short-term outcomes and exercise objectives	3.1 (0.8)	1.0 (0.0)
15 - Home exercise programme – Normalising movement	3.5 (0.7)	2.0 (1.0)
16 - Home exercise programme – Improving tolerance	2.7 (1.6)	2.2 (0.7)
17 - Exercise review	3.0 (1.2)	2.2 (1.1)
	2.4 (1.3)	1.1 (1.0)

Abbreviations: PO – Performance Objective; SD – Standard deviation; UEC – Usual Exercise Care

For peer review only

For peer review only - http://bmjopen.bmj.com/site/about/guidelines.xhtml

Contamination

Intervention content was scored for 29/32 usual exercise care appointments; three were excluded due to missing recordings. Mean checklist score was 1.1/5 (SD 1.0; range 0.6–1.5, 95% CI: 0.6, 1.6), with 1/17 components ('Improve self-efficacy') delivered in a manner consistent with EPIC-Neck (Table 3). This met the "Green" contamination criterion (Table 1).

Acceptability

Eighteen EPIC-Neck participants returned acceptability questionnaires (Table 4). Mean scores met the pre-defined ≥4/5 threshold for affective attitude, intervention coherence, perceived effectiveness, and self-efficacy. Opportunity costs, reflecting the extent to which doing the exercises interfered with other priorities, were neutral (mean 3.3, SD 1.0; 95% CI: 2.8, 3.9), and overall acceptability was 3.9 (SD 1.0; 95% CI: 3.7, 4.1).

For peer review only

For peer review only - http://bmjopen.bmj.com/site/about/guidelines.xhtml

379 **Table 4 – Acceptability summary**

Domain	Patient Questionnaire (n = 18) mean (SD) [95%CI]	Patient Interviews (n = 12)	Physiotherapist Interviews (n = 5)	Integrated summary
**Affective attitude**	**Green:** 4.0 (0.8) [3.7, 4.5]	**Amber:** Satisfaction with individualised, targeted approach and associated benefits, though some expressed negative feelings about physical side effects and general exercise; overall, positive outcomes outweighed lack of enjoyment.	**Green:** Enjoyed structured approach and behaviour change techniques	**Amber:** Address patient concerns about physical side effects and attitudes to general exercise
**Burden**	NA	**Amber:** Exercises that were complex or required specific space/positioning increased effort and were often omitted; time burden varied, with integration into daily routines reducing effort.	**Amber:** Cognitive and time burden due to intervention depth, volume and changing practice which improved with exposure.	**Amber:** Address patient burden related to exercise duration and complex exercises, and physiotherapist burden related to intervention depth and change in clinical practice.
**Ethicality**	NA	**Amber:** Alignment with values on individualised, non-pharmacological management, but misalignment for some with values around shared decision-making, dislike of routines, and preference for interpersonal support over written or video resources.	**Amber:** Alignment with individualised biopsychosocial patient centred care beliefs but misaligned for some regarding goal setting and shared decision-making beliefs.	**Amber:** Address patient misalignment with shared decision-making and resource preferences, and physiotherapist misalignment with goal-setting and shared decision-making.
**Intervention coherence**	**Green:** 4.1 (1.1) [3.6, 4.7]	**Red:** Patients understood basic exercise performance but had limited grasp of purpose, benefits, and progression strategies; understanding of flare-up management and general exercise plans was inconsistent	**Amber:** Coherent with familiar components, but low with new or more complex components – improved with practice but remained limited	**Red:** Improve patients' understanding of programme rationale, progression strategies, and flare-up management, and strengthen physiotherapists' understanding of how to deliver

For peer review only - http://bmjopen.bmj.com/site/about/guidelines.xhtml

		and rarely aligned with EPIC-Neck standards.		newer or more complex components.
**Opportunity costs**	**Amber:** 3.3 (1.0) [2.8, 3.9]	**Amber:** Most reported no trade-offs, but some described minor sacrifices outweighed by benefits, while others de-prioritised exercise in favour of other activities.	**Green:** Minimal trade-offs	**Amber:** Address patient de-prioritisation of exercise
**Perceived effectiveness**	**Green:** 4.1 (1.2) [3.5, 4.7]	**Green:** Consistent benefits in pain, disability and function, with sleep, mental well-being, and relationships improvements noted where limited at baseline	**Amber:** Overall perceived as effective with reservations regarding goal setting, social support and the patient resources	**Amber:** Address physiotherapist concerns about goal setting, social support, and patient resources.
**Self-efficacy**	**Green:** 4.1 (0.9) [3.7, 4.5]	**Amber:** Confident with basic exercises and self-managing flare-ups, but confidence declined with complexity, progression strategies, and general exercise citing fear of harm	**Amber:** Confident with familiar components, but low with new or more complex components – improved with practice but remained limited	**Amber:** Address patient confidence with complex exercises, safety, progression strategies, and general exercise; and physiotherapist confidence with new or complex components.
**Overall acceptability**	**Amber:** 3.9 (1.0) [3.7, 4.1]	-	-	**Amber:** Broadly acceptable with addressable concerns, particularly related to intervention coherence; further refinement and re-testing are required before a definitive trial.

380 Green: No areas of concern, mean acceptability score of ≥ 4 and no areas of concern identified in qualitative interviews

381 Amber: Minor problems that require attention before continuing to a definitive trial, mean acceptability score 3 and/or minor
382 problems identified in qualitative interviews

383 Red: Major problems that require urgent attention before continuing to a definitive trial, mean acceptability score ≤2 and/or major
384 problems identified in qualitative interviews

385 Abbreviations: SD – Standard deviation

For peer review only - http://bmjopen.bmj.com/site/about/guidelines.xhtml

Interviews were completed with 12 participants and 5 physiotherapists (Table 5). Qualitative analysis (Table 4; full details are in Supplementary File 5) indicated no participant concerns about intervention effectiveness. Addressable concerns were identified in other TFA domains, particularly intervention coherence, defined here as participants' understanding of the intervention and how it was intended to work. Participants showed limited understanding of the exercise rationale and performance objectives (exercise progression/regression, flare-up management, and general exercise). Physiotherapists reported positive affective attitude and minimal opportunity costs but had addressable concerns around coherence and self-efficacy when delivering newer or more complex components.

Table 5 – Interviewed patient and physiotherapist characteristics

	n	%
**Patient participants (n = 12)**		
**Gender**		
Female	4	33.3%
Male	8	66.7%
**Age**		
18-30	1	8.3%
31-40	3	25.0%
41-50	2	16.7%
51-60	3	25.0%
61+	3	25.0%
**Ethnicity**		
**Asian or Asian British**	**3**	**25.0%**
Indian	0	0.0%
Pakistani	3	25.0%
**Black or Black British**	**2**	**16.7%**
African	0	0.0%
Caribbean	1	8.3%
Other	1	8.3%
**Mixed**	**1**	**8.3%**
White & Black Caribbean	1	8.3%
**White**	**5**	**41.7%**
British	5	41.7%
Irish	0	0.0%

For peer review only

For peer review only - http://bmjopen.bmj.com/site/about/guidelines.xhtml

**Other**	**1**	**8.3%**
Arab	1	8.3%
**IMD decile**		
Most deprived (deciles 1-3)	10	83.3%
Middle (deciles 4-7)	2	16.7%
Least deprived (deciles 8-10)	0	0.0%
**Neck pain related disability (NDI)**		
No disability	2	16.7%
Mild disability	5	41.7%
Moderate disability	5	41.7%
Severe disability	0	0.0%
Complete disability	0	0.0%
**Physiotherapist (n = 5)**		
**Gender**		
Female	3	60%
Male	2	40%
**Age**		
20-29	1	20%
30-39	1	20%
40-49	1	20%
50-59	2	40%
**Highest qualification**		
Bachelor's degree	5	100%
**Years experience treating chronic non-specific neck pain**		
0-2	2	40%
3-5	0	0%
6-10	2	40%
11-20	1	20%
**Postgraduate training areas**		
Neck pain assessment and treatment	3	60%
Exercise prescription	3	60%
Behaviour change	2	40%
Psychologically informed practice	1	20%
Patient-centred communication techniques	3	60%

Abbreviations: IMD – Index of multiple deprivation

Integrated interpretation (Table 4) showed that, although questionnaires suggested good patient acceptability across most domains, interviews identified coherence gaps for both participants and physiotherapists. Overall, EPIC-Neck was acceptable, with addressable concerns, meeting the "Amber" progression criterion.

For peer review only - http://bmjopen.bmj.com/site/about/guidelines.xhtml

Allocated treatment engagement

Treatment was not initiated by 2/24 EPIC-Neck participants (8%) and 3/12 usual exercise care participants (25%). Treatment was completed by 18/24 EPIC-Neck participants (75%) and 6/12 usual exercise care participants (50%). A further 4/24 EPIC-Neck participants (17%) and 3/12 usual exercise care participants (25%) initiated treatment but were discharged following non-attendance.

Among participants attending ≥1 appointment, both groups attended a mean of 3.6 appointments: EPIC-Neck, SD 1.4, range 1–7; usual exercise care, SD 2.6, range 1–9. Mean DNA appointments were 0.7 in EPIC-Neck (SD 0.8, range 0–3) and 1.1 in usual exercise care (SD 1.3, range 0–3). Mean total appointment time was 1 hour 55 minutes (SD 56 minutes, range 27 minutes to 4 hours 7 minutes) in the EPIC-Neck group and 1 hour 30 minutes (SD 52 minutes, range 26 minutes to 2 hours 42 minutes) in the usual exercise care group. Mean treatment duration was 15.9 weeks (SD 8.7, range 0–31) in the EPIC-Neck group and 11.3 weeks (SD 10.0, range 0–29) in the usual exercise care group.

Safety

No exercise-related serious adverse events were reported. One participant experienced a temporary worsening of symptoms (related, non-serious), and one experienced transient syncope at work (unrelated, serious).

Inclusivity

No statistically significant differences were observed for any demographic characteristic between randomised and non-randomised individuals (Table 6).

For peer review only - http://bmjopen.bmj.com/site/about/guidelines.xhtml

For peer review only

Table 6 - Demographics randomised vs nonrandomised

**Demographic characteristic**	**Nonrandomised (n=251)**		**Randomised (n=36)**		P-value (from Pearson's Chi-Squared Test)
	n	%	*n*	%	
**Gender**					p = 0.110
Female	147	58.6%	16	44.4%	
Male	104	41.4%	20	55.6%	
**Age**					p = 0.445
18-30	33	13.1%	6	16.7%	
31-40	46	18.3%	8	22.2%	
41-50	54	21.5%	6	16.7%	
51-60	47	18.7%	10	27.8%	
61+	71	28.3%	6	16.7%	
**Ethnicity**					p = 0.549
Asian or Asian British	95	37.8%	11	30.6%	
Black or Black British	16	6.4%	5	13.9%	
Mixed	5	2.0%	1	2.8%	
White	73	29.1%	11	30.6%	
Other	7	2.8%	0	0.0%	
Not known	55	21.9%	8	22.2%	
**First language**					p = 0.264
English	84	33.5%	9	25.0%	
Other	50	19.9%	5	13.9%	
Not known	117	46.6%	22	61.1%	
**Interpreter required**					p = 0.434
No	227	90.4%	34	94.4%	
Yes	24	9.6%	2	5.6%	
**IMD decile**					p = 0.761
Most deprived (Deciles 1-3)	193	76.9%	27	75.0%	
Middle (Deciles 4-7)	46	18.3%	8	22.2%	
Least deprived (Deciles 8-10)	12	4.8%	1	2.8%	

Note: For consistency, demographic data were taken from the screening logs, as these could differ from self-reported baseline questionnaire data for randomised participants.

Abbreviations: IMD – Index of multiple deprivation

For peer review only

Feasibility of trial outcome measures

Summary statistics (means, SDs, 95% CIs) for outcomes and candidate mediators are shown in Table 7. The PSFS was not completed at baseline by two participants who reported no functional limitations. All other measures had 100% baseline return, and 75% and 67% return at 3 and 6 months, respectively. Return rates and item-level completeness are in Supplementary File 3–Table S3-2.

For peer review only

Table 7 - Descriptive summary of planned definitive trial outcome and candidate mediator measures

Outcome Measure	EPIC-Neck			Usual Exercise Care		
	n	Mean (SD)	95%CI	n	Mean (SD)	95%CI
**Planned definitive trial primary outcome measure**						
Neck pain-related disability – NDI (0 = best, 50 = worst)						
Baseline	24	14.13 (7.12)	11.12, 17.13	12	16.58 (8.84)	10.97, 22.20
3-Month	19	14.21 (10.97)	8.92, 19.50	8	16.50 (10.30)	7.89, 25.11
6-Month	18	12.39 (12.79)	6.03, 18.75	6	16.67 (12.47)	3.58, 29.75
**Planned definitive trial secondary outcome measures**						
Neck pain – VAS (0 = best, 10 = worst)						
Baseline	24	5.63 (1.86)	4.84, 6.41	12	5.67 (2.31)	4.20, 7.13
3-Month	19	4.42 (2.87)	3.04, 5.81	8	4.25 (2.55)	2.12, 6.38
6-Month	18	3.50 (2.64)	2.19, 4.81	6	4.00 (2.28)	1.61, 6.39
Functional and activity impact – PSFS (0 = worst, 10 = best)						
Baseline	22	5.09 (1.77)	4.31, 5.88	12	4.50 (2.43)	2.96, 6.04
3-Month	17	5.47 (2.48)	4.20, 6.74	8	4.75 (2.55)	2.62, 6.88
6-Month	16	6.69 (2.65)	5.27, 8.10	6	5.17 (2.93)	2.10, 8.24
Sleep – SCI (0 = worst, 32 = best)						
Baseline	24	16.50 (6.49)	13.76, 19.24	12	13.83 (6.39)	9.77, 17.90
3-Month	19	16.84 (9.55)	12.24, 21.44	8	13.88 (8.11)	7.09, 20.66
6-Month	18	18.72 (9.89)	13.80, 23.64	6	15.00 (6.60)	8.07, 21.93
Mental wellbeing – HADS (0 = best, 42 = worst)						
Baseline	24	12.46 (7.87)	9.14, 15.78	12	16.00 (10.59)	9.27, 22.73
3-Month	19	14.32 (10.26)	9.37, 19.26	8	16.50 (14.28)	4.56, 28.44
6-Month	18	12.89 (10.36)	7.74, 18.04	6	12.67 (12.34)	-0.28, 25.62
Relationships and social function – AQoL (0 = worst, 100 = best)						
Baseline	24	74.63 (17.21)	67.36, 81.89	12	70.75 (23.69)	55.70, 85.80
3-Month	19	71.42 (20.87)	61.36, 81.48	8	67.13 (27.30)	44.30, 89.95
6-Month	18	75.56 (20.56)	65.33, 85.78	6	68.83 (23.95)	43.70, 93.97
Health related quality of life – EQ-5D-5L (-0.594= worst, 1 = best)						
Baseline	24	0.65 (0.24)	0.54, 0.75	12	0.60 (0.26)	0.43, 0.77
3-Month	19	0.65 (0.32)	0.49, 0.80	8	0.62 (0.22)	0.44, 0.81

6-Month	18	0.65 (0.36)	0.47, 0.83	6	0.63 (0.20)	0.43, 0.84
**Potential measures of mediation**						
Night pain – NPRS (0 = best, 10 = worst)						
Baseline	24	5.58 (2.75)	4.42, 6.74	12	5.42 (2.35)	3.92, 6.91
3-Month	19	3.95 (2.84)	2.58, 5.32	8	4.63 (2.56)	2.48, 6.77
6-Month	18	3.28 (2.87)	1.85, 4.70	6	4.83 (3.13)	1.55, 8.11
Pain self-efficacy – PSEQ (0 = worst, 60 = best)						
Baseline	24	40.29 (12.67)	34.94, 45.64	12	36.33 (14.30)	27.25, 45.42
3-Month	19	44.53 (15.74)	36.94, 52.11	8	39.25 (13.95)	27.59, 50.91
6-Month	18	47.11 (16.05)	39.13, 55.09	6	41.17 (15.82)	24.57, 57.77
Pain beliefs – SOPA (0 = best, 28 = worst)						
Baseline	24	14.71 (3.63)	13.18, 16.24	12	13.50 (4.15)	10.87, 16.13
3-Month	19	13.95 (4.79)	11.64, 16.26	8	10.63 (3.38)	7.80, 13.45
6-Month	18	13.72 (3.83)	11.82, 15.63	6	10.50 (2.43)	7.95, 13.05
Fear of movement – TSK-13 (13 = best, 52 = worst)						
Baseline	24	27.13 (8.46)	23.55, 30.70	12	28.58 (9.64)	22.46, 34.71
3-Month	19	26.53 (9.77)	21.82, 31.23	8	29.50 (10.32)	20.87, 38.13
6-Month	18	24.44 (9.43)	19.75, 29.13	6	31.17 (8.75)	21.98, 40.35

Abbreviations: AQoL - Assessment of Quality-of-Life (relationships and self-worth domains only; HADS - Hospital Anxiety and Depression Scale (Anxiety and depression scores combined); NDI – Neck disability index; PSEQ – Pain self-efficacy questionnaire; PSFS – Patient specific functional scale; SCI – Sleep condition indicator; SD – Standard deviation; SOPA – Survey of Pain Attitudes Questionnaire (single item version); TSK - Tampa Scale of Kinesiophobia; VAS – Visual analogue scale

Note: Table 7 reports descriptive statistics only, to assess feasibility of outcome-measure collection, completeness and score distributions for a future definitive trial. No inferential between-group comparisons were planned or undertaken.

Progression to a definitive trial

Table 1 summarises feasibility findings against the prespecified traffic-light criteria: Green for recruitment and contamination; Amber for delivery fidelity and acceptability; Red for retention. Overall, progression was considered feasible with modification, specifically to strengthen retention, delivery fidelity, and intervention coherence.

For peer review only - http://bmjopen.bmj.com/site/about/guidelines.xhtml

DISCUSSION

This feasibility trial aimed to determine whether a definitive trial evaluating the clinical and cost-effectiveness of EPIC-Neck for chronic non-specific neck pain is feasible. The feasibility findings were mixed. Recruitment and contamination met Green progression criteria, indicating no major concerns in these domains. However, retention met the Red criterion and delivery fidelity met the Amber criterion, indicating important feasibility concerns that require resolution before a definitive trial. Acceptability to both patients and physiotherapists was broadly positive, but qualitative findings identified addressable concerns within the TFA domain of intervention coherence, particularly participants' understanding of the intervention rationale and physiotherapists' clarity around delivering newer or more complex components.

Recruitment was feasible, but retention posed modifiable challenges

Despite not reaching the estimated sample size, 4–5 participants were randomised per month, exceeding the green progression criteria. In contrast, 6-month retention was 67%. This is below the ≥80% target associated with lower risk of attrition bias and below the minimum 70% threshold prespecified for progression, raising concerns about whether current retention strategies would be sufficient in a definitive trial.[40-44]

Standard retention strategies were used (linguistic support, pre-paid envelopes, reminders, financial incentives)[43, 76, 77] and while further optimisation may help (electronic and shorter questionnaires, increased incentives)[43, 78] our findings suggest that retention may be influenced by treatment allocation and engagement,

For peer review only - http://bmjopen.bmj.com/site/about/guidelines.xhtml

rather than follow-up logistics. Retention differed by treatment allocation (50% usual exercise care vs 75% EPIC-Neck) and none of the participants who did not start treatment or were discharged for non-attendance returned follow-up measures. In contrast, treatment completers across both groups had 91% retention (22/24). Additionally, treatment completion was higher in EPIC-Neck (75% vs 50% usual exercise care). Taken together, these patterns indicate engagement-related attrition and possible allocation-preference effects within this unblinded feasibility trial, reinforcing the need to consider participant blinding in the definitive exercise-versus-exercise trial to reduce allocation-related differences.[79, 80]

Intervention coherence and delivery are suboptimal

Although overall acceptability was broadly positive, there were clear concerns within the intervention coherence domain, particularly among participants. Many had limited understanding of the programme rationale and performance objectives. These coherence deficits align with lower delivery fidelity scores for the corresponding components (mean scores <3), suggesting that limited patient understanding may have been related, at least in part, to suboptimal physiotherapist delivery. Physiotherapists also reported reduced confidence and clarity when delivering newer or more complex EPIC-Neck components.

Addressing these issues is crucial before progressing to a definitive trial. Poor fidelity can reduce treatment effects,[81] risk misinterpreting null findings [82, 83] and threaten internal validity.[84] However, delivery fidelity should not be interpreted as a simple success/failure judgement. Although only 14% of participants received EPIC-Neck at an acceptable overall fidelity level, 35% of components were delivered acceptably,

For peer review only - http://bmjopen.bmj.com/site/about/guidelines.xhtml

meaning some intended intervention mechanisms may still have been activated. Evidence from complex behavioural interventions also indicates that effectiveness may still be possible despite variable or moderate delivery fidelity, depending on which components are most critical to the intervention mechanism.[46, 85, 86] Nevertheless, fidelity concerns remain substantial because lower-fidelity EPIC-Neck components included those intended to support long-term self-management, such as flare-up management and general exercise, which may plausibly influence long-term clinical outcomes in a definitive trial. These concerns are modifiable. Consistent with Medical Research Council guidance, EPIC-Neck and its training package can be refined and re-tested, with a focus on strengthening delivery fidelity and intervention coherence before progressing to a definitive evaluation.[22]

EPIC-Neck was acceptable with concerns about intervention coherence and was distinct from usual exercise care

Despite concerns about intervention coherence preventing immediate progression to a definitive trial, EPIC-Neck was broadly acceptable across the remaining domains to both patients and physiotherapists. Additionally, the operationalisation of EPIC-Neck components (e.g., behaviour change strategies, performance objectives) appears different from usual exercise care.[87] This reduces the risk of contamination in a definitive trial diluting any between-group effect.[88-90] Overall, EPIC-Neck appears acceptable and sufficiently distinct from usual exercise care, but requires refinement and further fidelity testing before definitive evaluation.

For peer review only

For peer review only - http://bmjopen.bmj.com/site/about/guidelines.xhtml

Future refinement before a definitive trial

Before progression to a definitive trial, key refinements are needed to optimise retention, delivery fidelity and intervention coherence. Retention strategies should address both questionnaire return and treatment engagement, as missing follow-up data appeared linked to treatment non-initiation and non-attendance. Future procedures could consider reducing questionnaire burden (e.g., electronic completion or shorter questionnaires), increasing the financial incentive for completion, and incorporating blinding procedures where feasible.[43, 78-80] Additional strategies to optimise delivery and intervention coherence should be designed collaboratively with patients and physiotherapists, and informed by theory and existing evidence. Potential refinements include strengthening physiotherapist training through longer training duration, additional practice, and regular monitoring and feedback.[91-95] Intervention coherence may improve through better delivery, but additional strategies could include methods to check patient understanding during treatment, such as teach-back approaches.[96]

Strengths and limitations

A key strength of this trial is the integration of quantitative and qualitative data, incorporating perspectives from both patients and physiotherapists, enabling a more comprehensive assessment of feasibility. A further strength is that 94% of appointments were audio-recorded, providing a robust assessment of delivery fidelity and contamination.[97] Finally, efforts to minimise exclusion based on English language and age resulted in a diverse cohort (40% Asian; age range 19–82), enhancing external validity by better reflecting populations attending NHS musculoskeletal services.[28]

For peer review only

For peer review only - http://bmjopen.bmj.com/site/about/guidelines.xhtml

The target sample size was not reached, which limits the precision of feasibility estimates and means progression criteria and subgroup patterns should be interpreted cautiously. The 2:1 allocation ratio increased intervention exposure and feedback but resulted in fewer usual exercise care participants, reducing the precision of usual-care estimates and limiting interpretation of between-group descriptive patterns. Participants and outcome assessors were not blinded, which may have introduced performance or detection bias, particularly for self-reported outcomes; blinding of outcome assessment will therefore be incorporated into the definitive trial where feasible. Physiotherapists delivered EPIC-Neck to relatively few patients; therefore, some fidelity issues may be overestimated and could improve with increased exposure and practice.[98, 99] Fidelity assessments were undertaken unblinded, which may have influenced scoring despite use of a structured competence checklist. In addition, interviews were conducted by the intervention developer, whose role in EPIC-Neck development may have introduced social desirability and allegiance bias. The use of topic guides, reflective discussions and team-based interpretation helped mitigate this risk, but independent interviewing and analysis would strengthen future evaluations.

CONCLUSION

The EPIC-Neck programme shows potential for evaluation in a definitive trial, but progression is conditional on further refinement. Recruitment and contamination findings were encouraging, and the intervention was broadly acceptable and distinct from usual exercise care. However, retention, delivery fidelity and intervention coherence require optimisation before definitive evaluation. Future work should refine the EPIC-Neck programme, training package and trial retention procedures,

For peer review only - http://bmjopen.bmj.com/site/about/guidelines.xhtml

followed by targeted repeat testing of delivery fidelity, participant and physiotherapist understanding of the intervention and retention. Depending on the extent of these refinements, this may require a further feasibility or pilot study before progressing to a full-scale definitive trial.

For peer review only

For peer review only - http://bmjopen.bmj.com/site/about/guidelines.xhtml

LIST OF ABBREVIATIONS

AQoL. Assessment of Quality-of-Life

CI. Confidence Interval

DNA. Did Not Attend

EPIC. Exercise Prescription Improved through Co-design

HADS. Hospital Anxiety and Depression Scale

IMD. Index of Multiple Deprivation

NDI. Neck disability index

NHS. National Health Service

NIH-BCC. National Institutes of Health-Behaviour Change Consortium

PIL. Participant Information Leaflet

PO. Performance Objectives

PPIE. Patient and Public Involvement and Engagement

PSEQ. Pain self-efficacy questionnaire

PSFS. Patient Specific Functional Scale

SCI. Sleep condition indicator

SD. Standard Deviation

SOPA. Survey of Pain Attitudes Questionnaire

For peer review only

TFA. Theoretical Framework of Acceptability

TSK. Tampa Scale of Kinesiophobia

UEC. Usual Exercise Care

VAS. Visual analogue scale

DECLARATIONS

Ethics approval and consent to participate

The study was registered with ISRCTN81746901 and received ethical approval from South Central - Oxford B Research Ethics Committee (24/SC/0006). Patient participants and physiotherapists gave informed consent to participate in the study before taking part.

Participant consent for publication

Not applicable.

Availability of data and materials

Data are available upon reasonable request. Data sharing requests can be considered via contact with the corresponding author.

Competing interests

The authors declare that they have no competing interests

For peer review only

Funding

Jonathan Price, Clinical Doctoral Research Fellow, NIHR302141 is funded by HEE/NIHR for this research project. The views expressed in this publication are those of the author(s) and not necessarily those of the NIHR, NHS or the UK Department of Health and Social Care.

The funders are not involved in trial design, data collection, management, analysis, writing of the report or the decision to submit this report for publication.

The study sponsor is Birmingham Community Healthcare NHS Foundation Trust (BCHC.researchinnovation@nhs.net).

Author Contributions

JP was the chief investigator and conceived and designed the study, was awarded the funding and drafted the manuscript. AR, NI, CG, PP contributed to the study design, provided specific content and edited the trial protocol and manuscript. MB contributed to data analysis. All authors have reviewed and approved the manuscript.

Collaborators

EPIC-Neck feasibility trial collaborators: Apurba Subedi (Birmingham Community Healthcare NHS Foundation Trust), Hayley Alderton (Berkshire Healthcare NHS Foundation Trust), Katherine Yeung (Birmingham Community Healthcare NHS Foundation Trust), Longsun Yau (Birmingham Community Healthcare NHS Foundation Trust), Paul Matthews (Nottingham University Hospitals NHS Trust), Rajvinder Shokkar (Berkshire Healthcare NHS Foundation Trust)

For peer review only

For peer review only - http://bmjopen.bmj.com/site/about/guidelines.xhtml

Acknowledgements

We thank the EPIC-Neck patient and public involvement and engagement group for their contributions.

Author details

[1]Musculoskeletal Physiotherapy Services, Birmingham Community Healthcare NHS Foundation Trust, Birmingham, United Kingdom

[2]Research and Innovation, Birmingham Community Healthcare NHS Foundation Trust, Birmingham, United Kingdom

[3]School of Sport, Exercise and Rehabilitation Sciences, College of Life and Environmental Sciences, University of Birmingham, Birmingham, United Kingdom

[4]School of Physical Therapy, Western University, Ontario, Canada

[5]Institute of Applied Health Research, College of Medical and Dental Sciences, University of Birmingham, Birmingham, United Kingdom

*Corresponding author

Email: Jonathan.price2@nhs.net

ORCID iDs

Jonathan Price: https://orcid.org/0000-0002-5505-1564

Alison Rushton: https://orcid.org/0000-0001-8114-7669

Natalie Ives: https://orcid.org/0000-0002-1664-7541

For peer review only

Marilyn Bradbury: https://orcid.org/0000-0001-6000-7229

Priti Parmar: https://orcid.org/0000-0003-4344-144X

Colin J Greaves: https://orcid.org/0000-0003-4425-2691

FIGURES

Figure 1 – CONSORT flow diagram. Abbreviations: CNSNP – Chronic non-specific neck pain; DNA – Did not attend; PIL – Participant information leaflet

SUPPLEMENTARY FILES

Supplementary file 1 – CONSORT for pilot and feasibility trials

Supplementary file 2 – Feasibility trial protocol ethics

Supplementary file 3 – Supporting information

Supplementary file 4 – GRIPP2-SF

Supplementary file 5 – Acceptability data

For peer review only

For peer review only - http://bmjopen.bmj.com/site/about/guidelines.xhtml

REFERENCES

1. Xu F, Zhang X, Yang M, et al. Magnitude, temporal trend and inequality in burden of neck pain: an analysis of the Global Burden of Disease Study 2019. *BMC Musculoskelet Disord* 2025;26(1):202. doi: 10.1186/s12891-025-08342-3

2. Myhrvold BL, Irgens P, Robinson HS, et al. Visual trajectory pattern as prognostic factors for neck pain. *Eur J Pain* 2020;24(9):1752-64. doi: 10.1002/ejp.1622

3. Horn ME, Fritz JM. Timing of physical therapy consultation on 1-year healthcare utilization and costs in patients seeking care for neck pain: a retrospective cohort. *BMC Health Serv Res* 2018;18(1):887. doi: 10.1186/s12913-018-3699-0

4. Hey HWD, Lim JXY, Ong JZ, et al. Epidemiology of Neck Pain and Its Impact on Quality-of-Life-A Population-Based, Cross Sectional Study in Singapore. *Spine (Phila Pa 1976)* 2021;46(22):1572-80. doi: 10.1097/BRS.0000000000004071

5. Irgens P, Myhrvold BL, Kongsted A, et al. The clinical course of neck pain: Are trajectory patterns stable over a 1-year period? *Eur J Pain* 2022;26(2):531-42. doi: 10.1002/ejp.1879

6. Hallman DM, Holtermann A, Dencker-Larsen S, et al. Are trajectories of neck-shoulder pain associated with sick leave and work ability in workers? A 1-year prospective study. *BMJ Open* 2019;9(3):e022006. doi: 10.1136/bmjopen-2018-022006

7. National Institute for Health and Care Excellence. Chronic pain (primary and secondary) in over 16s: assessment of all chronic pain and management of chronic primary pain [Internet]. London: NICE; 2021 [Available from: https://www.nice.org.uk/guidance/ng193.

8. Bier JD, Scholten-Peeters WGM, Staal JB, et al. Clinical Practice Guideline for Physical Therapy Assessment and Treatment in Patients With Nonspecific Neck Pain. *Phys Ther* 2018;98(3):162-71. doi: 10.1093/ptj/pzx118

9. Blanpied PR, Gross AR, Elliott JM, et al. Neck Pain: Revision 2017. *J Orthop Sports Phys Ther* 2017;47(7):A1-A83. doi: 10.2519/jospt.2017.0302

10. Wilhelm MP, Donaldson M, Griswold D, et al. The Effects of Exercise Dosage on Neck-Related Pain and Disability: A Systematic Review With Meta-analysis. *J Orthop Sports Phys Ther* 2020;50(11):607-21. doi: 10.2519/jospt.2020.9155

11. de Zoete RMJ, Brown L, Oliveira K, et al. The effectiveness of general physical exercise for individuals with chronic neck pain: a systematic review of randomised controlled trials. *Eur J Physiother* 2020;22(3):141-47. doi: 10.1080/21679169.2018.1561942

12. Garzonio S, Arbasetti C, Geri T, et al. Effectiveness of Specific Exercise for Deep Cervical Muscles in Nonspecific Neck Pain: A Systematic Review and Meta-Analysis. *Phys Ther* 2022;102(5):pzac001. doi: 10.1093/ptj/pzac001

13. Martin-Gomez C, Sestelo-Diaz R, Carrillo-Sanjuan V, et al. Motor control using cranio-cervical flexion exercises versus other treatments for non-specific chronic neck pain: A systematic review and meta-analysis. *Musculoskelet Sci Pract* 2019;42:52-59. doi: 10.1016/j.msksp.2019.04.010

For peer review only - http://bmjopen.bmj.com/site/about/guidelines.xhtml

14. Li Y, Li S, Jiang J, et al. Effects of yoga on patients with chronic nonspecific neck pain: A PRISMA systematic review and meta-analysis. *Medicine (Baltimore)* 2019;98(8):e14649. doi: 10.1097/MD.0000000000014649

15. Martini JD, Ferreira GE, Xavier de Araujo F. Pilates for neck pain: A systematic review and meta-analysis of randomised controlled trials. *J Bodyw Mov Ther* 2022;31:37-44. doi: 10.1016/j.jbmt.2022.03.011

16. Price J, Rushton A, Tyros V, et al. Expert consensus on the important chronic non-specific neck pain motor control and segmental exercise and dosage variables: An international e-Delphi study. *PLoS One* 2021;16(7):e0253523. doi: 10.1371/journal.pone.0253523

17. McLean SM, Klaber Moffett JA, Sharp DM, et al. A randomised controlled trial comparing graded exercise treatment and usual physiotherapy for patients with non-specific neck pain (the GET UP neck pain trial). *Man Ther* 2013;18(3):199-205. doi: 10.1016/j.math.2012.09.005

18. Villanueva-Ruiz I, Falla D, Saez M, et al. Manual therapy and neck-specific exercise are equally effective for treating non-specific neck pain but only when exercise adherence is maximised: A randomised controlled trial. *Musculoskelet Sci Pract* 2025;77:103319. doi: 10.1016/j.msksp.2025.103319

19. Dalager T, Welch A, O'Leary SP, et al. Clinically Relevant Decreases in Neck/Shoulder Pain Among Office Workers Are Associated With Strength Training Adherence and Exercise Compliance: Explorative Analyses From a Randomized Controlled Trial. *Phys Ther* 2023;103(2):pzac166. doi: 10.1093/ptj/pzac166

20. Zebis MK, Andersen CH, Sundstrup E, et al. Time-wise change in neck pain in response to rehabilitation with specific resistance training: implications for exercise prescription. *PLoS One* 2014;9(4):e93867. doi: 10.1371/journal.pone.0093867

21. Price J, Rushton A, Ives N, et al. Co-development of an evidence-informed, theoretically driven exercise programme for people with chronic non-specific neck pain (the EPIC-Neck programme - "Exercise Prescription Improved through Co-design"). *BMC Musculoskelet Disord* 2025;26(1):689. doi: 10.1186/s12891-025-08918-z

22. Skivington K, Matthews L, Simpson SA, et al. A new framework for developing and evaluating complex interventions: update of Medical Research Council guidance. *BMJ* 2021;374:n2061. doi: 10.1136/bmj.n2061

23. Eldridge SM, Chan CL, Campbell MJ, et al. CONSORT 2010 statement: extension to randomised pilot and feasibility trials. *BMJ* 2016;355:i5239. doi: 10.1136/bmj.i5239

24. Price J, Rushton A, Ives N, et al. Co-designed neck exercise (EPIC-Neck) vs usual exercise care for people with chronic non-specific neck pain: protocol for a randomised feasibility study with process evaluation. *Pilot Feasibility Stud* 2025;11(1):26. doi: 10.1186/s40814-025-01608-6

25. National Institute for Health and Care Excellence. Neck pain - non-specific: Clinical Knowledge Summaries 2023 [Available from: https://cks.nice.org.uk/topics/neck-pain-non-specific/.

26. Tsakitzidis G, Remmen R, Dankaerts W, et al. Non-specific neck pain and evidence-based practice. *European scientific journal* 2013;9(3):1-19.

For peer review only - http://bmjopen.bmj.com/site/about/guidelines.xhtml

27. NHS England. Increasing diversity in research participation: A good practice guide for engaging with underrepresented groups [Internet]. London: NHS England; 2023 [Available from: https://www.england.nhs.uk/aac/publication/increasing-diversity-in-research-participation/.

28. National Institute for Health and Care Research. Improving inclusion of under-served groups in clinical research: Guidance from INCLUDE Project [Internet]. London: NIHR; 2022 [Available from: https://www.nihr.ac.uk/documents/improving-inclusion-of-under-served-groups-in-clinical-research-guidance-from-include-project/25435.

29. Thabane L, Lancaster G. A guide to the reporting of protocols of pilot and feasibility trials. *Pilot Feasibility Stud* 2019;5(1):37. doi: 10.1186/s40814-019-0423-8

30. Armijo-Olivo S, Fuentes J, Da Costa BR, et al. Blinding in Physical Therapy Trials and Its Association with Treatment Effects. *American Journal of Physical Medicine & Rehabilitation* 2017;96(1):34-44. doi: 10.1097/phm.0000000000000521

31. Miller WR, Rollnick S. Motivational Interviewing, Second Edition: Preparing People for Change. 2nd ed. New York, NY: Guilford Publications 2002.

32. Elwyn G, Durand MA, Song J, et al. A three-talk model for shared decision making: multistage consultation process. *BMJ* 2017;359:j4891. doi: 10.1136/bmj.j4891

33. To D, De Carvalho D, Pike A, et al. A qualitative study exploring perceived barriers and enablers to fidelity of training and delivery for an intervention to reduce non-indicated imaging for low back pain. *Chiropr Man Therap* 2023;31(1):6. doi: 10.1186/s12998-023-00480-6

34. Beneciuk JM, George SZ, Greco CM, et al. Targeted interventions to prevent transitioning from acute to chronic low back pain in high-risk patients: development and delivery of a pragmatic training course of psychologically informed physical therapy for the TARGET trial. *Trials* 2019;20(1):256. doi: 10.1186/s13063-019-3350-3

35. Craven K, Holmes J, Powers K, et al. Embedding mentoring to support trial processes and implementation fidelity in a randomised controlled trial of vocational rehabilitation for stroke survivors. *BMC Med Res Methodol* 2021;21(1):203. doi: 10.1186/s12874-021-01382-y

36. Mellor K, Albury C, Dutton SJ, et al. Recommendations for progression criteria during external randomised pilot trial design, conduct, analysis and reporting. *Pilot Feasibility Stud* 2023;9(1):59. doi: 10.1186/s40814-023-01291-5

37. Price J, Rushton A, Tyros I, et al. Effectiveness and optimal dosage of exercise training for chronic non-specific neck pain: A systematic review with a narrative synthesis. *PLoS One* 2020;15(6):e0234511. doi: 10.1371/journal.pone.0234511

38. Littlewood C, Bateman M, Brown K, et al. A self-managed single exercise programme versus usual physiotherapy treatment for rotator cuff tendinopathy: a randomised controlled trial (the SELF study). *Clin Rehabil* 2016;30(7):686-96. doi: 10.1177/0269215515593784

39. Lamb SE, Williamson EM, Heine PJ, et al. Exercises to improve function of the rheumatoid hand (SARAH): a randomised controlled trial. *Lancet* 2015;385(9966):421-9. doi: 10.1016/S0140-6736(14)60998-3

For peer review only - http://bmjopen.bmj.com/site/about/guidelines.xhtml

787 40. Hu S, Guinan E, Mockler D, et al. Retention rates and reasons for non-retention in
788 exercise oncology trials in the post-treatment phase-a systematic review. *J Cancer*
789 *Surviv* 2025;19(5):1535-43. doi: 10.1007/s11764-024-01569-4

790 41. Watson S, Wong C, Smith T. Factors associated with trial recruitment and retention of
791 people with osteoarthritis: analysis of 215 randomised controlled trials from 2013-
792 2021. *Physiother Rev* 2022;26(2):5-19. doi: 10.5114/phr.2022.117578

793 42. Jacques RM, Ahmed R, Harper J, et al. Recruitment, consent and retention of
794 participants in randomised controlled trials: a review of trials published in the National
795 Institute for Health Research (NIHR) Journals Library (1997-2020). *BMJ Open*
796 2022;12(2):e059230. doi: 10.1136/bmjopen-2021-059230

797 43. Elfeky A, Gillies K, Gardner H, et al. Non-randomised evaluations of strategies to
798 increase participant retention in randomised controlled trials: a systematic review.
799 *Syst Rev* 2020;9(1):224. doi: 10.1186/s13643-020-01471-x

800 44. Higgins JPT, Green S. Cochrane Handbook for Systematic Reviews of Interventions.
801 Chichester, UK: The Cochrane Collaboration 2011.

802 45. Thompson DP, Oldham JA, Woby SR. Does adding cognitive-behavioural physiotherapy
803 to exercise improve outcome in patients with chronic neck pain? A randomised
804 controlled trial. *Physiotherapy* 2016;102(2):170-7. doi: 10.1016/j.physio.2015.04.008

805 46. Frost J, Wingham J, Britten N, et al. Home-based rehabilitation for heart failure with
806 reduced ejection fraction: mixed methods process evaluation of the REACH-HF
807 multicentre randomised controlled trial. *BMJ Open* 2019;9(8):e026039. doi:
808 10.1136/bmjopen-2018-026039

809 47. Thompson TP, Lambert JD, Greaves CJ, et al. Intervention delivery fidelity assessment
810 of a counseling-based intervention for promoting smoking reduction and increasing
811 physical activity. *Health Psychol* 2018;37(7):627-37. doi: 10.1037/hea0000613

812 48. Dreyfus SE. The Five-Stage Model of Adult Skill Acquisition. *Bull Sci Technol Soc*
813 2004;24(3):177-81. doi: 10.1177/0270467604264992

814 49. Cross R, Greaves CJ, Withall J, et al. Delivery fidelity of the REACT (REtirement in
815 ACTion) physical activity and behaviour maintenance intervention for community
816 dwelling older people with mobility limitations. *BMC Public Health* 2022;22(1):1112.
817 doi: 10.1186/s12889-022-13496-z

818 50. Withers TM, Greaves CJ, Bown MJ, et al. A feasibility study of the CRISP intervention; a
819 cardiovascular risk reduction intervention in patients with an abdominal aortic
820 aneurysm. *NIHR Open Res* 2024;4:34. doi: 10.3310/nihropenres.13596.2

821 51. Luker JA, Craig LE, Bennett L, et al. Implementing a complex rehabilitation intervention
822 in a stroke trial: a qualitative process evaluation of AVERT. *BMC Med Res Methodol*
823 2016;16:52. doi: 10.1186/s12874-016-0156-9

824 52. Robinson K, Allen F, Darby J, et al. Contamination in complex healthcare trials: the falls
825 in care homes (FinCH) study experience. *BMC Med Res Methodol* 2020;20(1):46.
826 doi: 10.1186/s12874-020-00925-z

For peer review only

For peer review only - http://bmjopen.bmj.com/site/about/guidelines.xhtml

827 53. Sekhon M, Cartwright M, Francis JJ. Development of a theory-informed questionnaire to assess the acceptability of healthcare interventions. *BMC Health Serv Res* 2022;22(1):279. doi: 10.1186/s12913-022-07577-3

830 54. Sekhon M, Cartwright M, Francis JJ. Acceptability of healthcare interventions: an overview of reviews and development of a theoretical framework. *BMC Health Serv Res* 2017;17(1):88. doi: 10.1186/s12913-017-2031-8

833 55. Vernon H, Mior S. The Neck Disability Index: a study of reliability and validity. *J Manipulative Physiol Ther* 1991;14(7):409-15.

835 56. Bobos P, MacDermid JC, Walton DM, et al. Patient-Reported Outcome Measures Used for Neck Disorders: An Overview of Systematic Reviews. *J Orthop Sports Phys Ther* 2018;48(10):775-88. doi: 10.2519/jospt.2018.8131

838 57. Modarresi S, Lukacs MJ, Ghodrati M, et al. A Systematic Review and Synthesis of Psychometric Properties of the Numeric Pain Rating Scale and the Visual Analog Scale for Use in People With Neck Pain. *Clin J Pain* 2021;38(2):132-48. doi: 10.1097/AJP.0000000000000999

842 58. Feng YS, Kohlmann T, Janssen MF, et al. Psychometric properties of the EQ-5D-5L: a systematic review of the literature. *Qual Life Res* 2021;30(3):647-73. doi: 10.1007/s11136-020-02688-y

845 59. Zigmond AS, Snaith RP. The hospital anxiety and depression scale. *Acta Psychiatr Scand* 1983;67(6):361-70. doi: 10.1111/j.1600-0447.1983.tb09716.x

847 60. Espie CA, Kyle SD, Hames P, et al. The Sleep Condition Indicator: a clinical screening tool to evaluate insomnia disorder. *BMJ Open* 2014;4(3):e004183. doi: 10.1136/bmjopen-2013-004183

850 61. Stratford P, Gill C, Westaway M, et al. Assessing disability and change on individual patients: a report of a patient specific measure. *Physiother Can* 1995;47(4):258-63.

852 62. Maxwell A, Ozmen M, Iezzi A, et al. Deriving population norms for the AQoL-6D and AQoL-8D multi-attribute utility instruments from web-based data. *Qual Life Res* 2016;25(12):3209-19. doi: 10.1007/s11136-016-1337-z

855 63. Richardson J, Iezzi A, Khan MA, et al. Validity and reliability of the Assessment of Quality of Life (AQoL)-8D multi-attribute utility instrument. *Patient* 2014;7(1):85-96. doi: 10.1007/s40271-013-0036-x

858 64. Jensen MP, Keefe FJ, Lefebvre JC, et al. One- and two-item measures of pain beliefs and coping strategies. *Pain* 2003;104(3):453-69. doi: 10.1016/S0304-3959(03)00076-9

861 65. Dupuis F, Cherif A, Batcho C, et al. The Tampa Scale of Kinesiophobia: A Systematic Review of Its Psychometric Properties in People With Musculoskeletal Pain. *Clin J Pain* 2023;39(5):236-47. doi: 10.1097/AJP.0000000000001104

864 66. Nicholas MK. The pain self-efficacy questionnaire: Taking pain into account. *Eur J Pain* 2007;11(2):153-63. doi: 10.1016/j.ejpain.2005.12.008

For peer review only

67. Withers TM, Garner NJ, Thorley CS, et al. Intervention fidelity assessment: A sub-study of the Norfolk Diabetes Prevention Study (NDPS). *Br J Health Psychol* 2023;28(3):740-52. doi: 10.1111/bjhp.12651

68. Thabane L, Ma J, Chu R, et al. A tutorial on pilot studies: the what, why and how. *BMC Med Res Methodol* 2010;10(1):1. doi: 10.1186/1471-2288-10-1

69. Gale NK, Heath G, Cameron E, et al. Using the framework method for the analysis of qualitative data in multi-disciplinary health research. *BMC Med Res Methodol* 2013;13:117. doi: 10.1186/1471-2288-13-117

70. Klingberg S, Stalmeijer RE, Varpio L. Using framework analysis methods for qualitative research: AMEE Guide No. 164. *Med Teach* 2024;46(5):603-10. doi: 10.1080/0142159X.2023.2259073

71. Teare MD, Dimairo M, Shephard N, et al. Sample size requirements to estimate key design parameters from external pilot randomised controlled trials: a simulation study. *Trials* 2014;15(1):264. doi: 10.1186/1745-6215-15-264

72. Public Health England. Chronic pain in adults [Internet]. London: Public Health England; 2017 [Available from: https://assets.publishing.service.gov.uk/media/5fc8c6b78fa8f547585ed7f3/Chronic_Pain_Report.pdf.

73. Adebajo A, Shikoh S, Kumar K, et al. Ethnic minority musculoskeletal health. *Rheumatology (Oxford)* 2018;57(2):201-03. doi: 10.1093/rheumatology/kex006

74. Versus Arthritis. Chronic pain in England: Unseen, unequal, unfair [Internet]. London: Versus Arthritis; 2021 [Available from: https://www.versusarthritis.org/about-arthritis/data-and-statistics/chronic-pain-in-england/.

75. Staniszewska S, Brett J, Simera I, et al. GRIPP2 reporting checklists: tools to improve reporting of patient and public involvement in research. *Res Involv Engagem* 2017;3(1):13. doi: 10.1186/s40900-017-0062-2

76. Gillies K, Kearney A, Keenan C, et al. Strategies to improve retention in randomised trials. *Cochrane Database Syst Rev* 2021(3):MR000032. doi: 10.1002/14651858.MR000032.pub3

77. Brueton V, Stenning SP, Stevenson F, et al. Best practice guidance for the use of strategies to improve retention in randomized trials developed from two consensus workshops. *J Clin Epidemiol* 2017;88:122-32. doi: 10.1016/j.jclinepi.2017.05.010

78. Elfeky A, Treweek S, Hannes K, et al. Using qualitative methods in pilot and feasibility trials to inform recruitment and retention processes in full-scale randomised trials: a qualitative evidence synthesis. *BMJ Open* 2022;12(4):e055521. doi: 10.1136/bmjopen-2021-055521

79. Hrobjartsson A, Emanuelsson F, Skou Thomsen AS, et al. Bias due to lack of patient blinding in clinical trials. A systematic review of trials randomizing patients to blind and nonblind sub-studies. *Int J Epidemiol* 2014;43(4):1272-83. doi: 10.1093/ije/dyu115

For peer review only

For peer review only - http://bmjopen.bmj.com/site/about/guidelines.xhtml

80. Armijo-Olivo S, Fuentes J, da Costa BR, et al. Blinding in Physical Therapy Trials and Its Association with Treatment Effects: A Meta-epidemiological Study. *Am J Phys Med Rehabil* 2017;96(1):34-44. doi: 10.1097/PHM.0000000000000521

81. Cook CE, Thigpen CA. Five good reasons to be disappointed with randomized trials. *J Man Manip Ther* 2019;27(2):63-65. doi: 10.1080/10669817.2019.1589697

82. Carroll C, Patterson M, Wood S, et al. A conceptual framework for implementation fidelity. *Implement Sci* 2007;2:40. doi: 10.1186/1748-5908-2-40

83. Toomey E, Hardeman W, Hankonen N, et al. Focusing on fidelity: narrative review and recommendations for improving intervention fidelity within trials of health behaviour change interventions. *Health Psychol Behav Med* 2020;8(1):132-51. doi: 10.1080/21642850.2020.1738935

84. Borrelli B. The Assessment, Monitoring, and Enhancement of Treatment Fidelity In Public Health Clinical Trials. *J Public Health Dent* 2011;71(Suppl 1):S52-63. doi: 10.1111/j.1752-7325.2011.00233.x

85. Dalal HM, Taylor RS, Jolly K, et al. The effects and costs of home-based rehabilitation for heart failure with reduced ejection fraction: The REACH-HF multicentre randomized controlled trial. *Eur J Prev Cardiol* 2019;26(3):262-72. doi: 10.1177/2047487318806358

86. Stathi A, Withall J, Greaves CJ, et al. A group-based exercise and behavioural maintenance intervention for adults over 65 years with mobility limitations: the REACT RCT. *Public Health Research* 2022;10(14):1-172. doi: 10.3310/mqbw6832

87. Kunstler BE, Cook JL, Freene N, et al. Physiotherapists use a small number of behaviour change techniques when promoting physical activity: A systematic review comparing experimental and observational studies. *J Sci Med Sport* 2018;21(6):609-15. doi: 10.1016/j.jsams.2017.10.027

88. Magill N, Knight R, McCrone P, et al. A scoping review of the problems and solutions associated with contamination in trials of complex interventions in mental health. *BMC Med Res Methodol* 2019;19(1):4. doi: 10.1186/s12874-018-0646-z

89. Gross A, Kay TM, Paquin JP, et al. Exercises for mechanical neck disorders. *Cochrane Database Syst Rev* 2015(1):CD004250. doi: 10.1002/14651858.CD004250.pub5

90. Hemming K, Taljaard M, Moerbeek M, et al. Contamination: How much can an individually randomized trial tolerate? *Stat Med* 2021;40(14):3329-51. doi: 10.1002/sim.8958

91. Simpson P, Holopainen R, Schutze R, et al. Becoming confidently competent: a qualitative investigation of training in cognitive functional therapy for persistent low back pain. *Physiother Theory Pract* 2024;40(4):804-16. doi: 10.1080/09593985.2022.2151333

92. Lawford BJ, Delany C, Bennell KL, et al. Training Physical Therapists in Person-Centered Practice for People With Osteoarthritis: A Qualitative Case Study. *Arthritis Care Res (Hoboken)* 2018;70(4):558-70. doi: 10.1002/acr.23314

93. Simpson P, Holopainen R, Schutze R, et al. Training of Physical Therapists to Deliver Individualized Biopsychosocial Interventions to Treat Musculoskeletal Pain

For peer review only

For peer review only - http://bmjopen.bmj.com/site/about/guidelines.xhtml

Conditions: A Scoping Review. *Phys Ther* 2021;101(10):pzab188. doi: 10.1093/ptj/pzab188

94. Hatfield TG, Withers TM, Greaves CJ. Systematic review of the effect of training interventions on the skills of health professionals in promoting health behaviour, with meta-analysis of subsequent effects on patient health behaviours. *BMC Health Serv Res* 2020;20(1):593. doi: 10.1186/s12913-020-05420-1

95. Newton C, Singh G, Nolan D, et al. Cognitive Functional Therapy compared with usual physiotherapy care in people with persistent low back pain: a mixed methods feasibility randomised controlled trial in the United Kingdom National Health Service. *Physiotherapy* 2024;123:118-32. doi: 10.1016/j.physio.2024.02.003

96. Shersher V, Haines TP, Sturgiss L, et al. Definitions and use of the teach-back method in healthcare consultations with patients: A systematic review and thematic synthesis. *Patient Educ Couns* 2021;104(1):118-29. doi: 10.1016/j.pec.2020.07.026

97. Toomey E, Matthews J, Guerin S, et al. Development of a Feasible Implementation Fidelity Protocol Within a Complex Physical Therapy-Led Self-Management Intervention. *Phys Ther* 2016;96(8):1287-98. doi: 10.2522/ptj.20150446

98. Rathod T, Belcher J, Montgomery AA, et al. Health services changes: is a run-in period necessary before evaluation in randomised clinical trials? *Trials* 2014;15(1):41. doi: 10.1186/1745-6215-15-41

99. Khan N, Abboudi H, Khan MS, et al. Measuring the surgical 'learning curve': methods, variables and competency. *BJU Int* 2014;113(3):504-8. doi: 10.1111/bju.12197

For peer review only

For peer review only - http://bmjopen.bmj.com/site/about/guidelines.xhtml

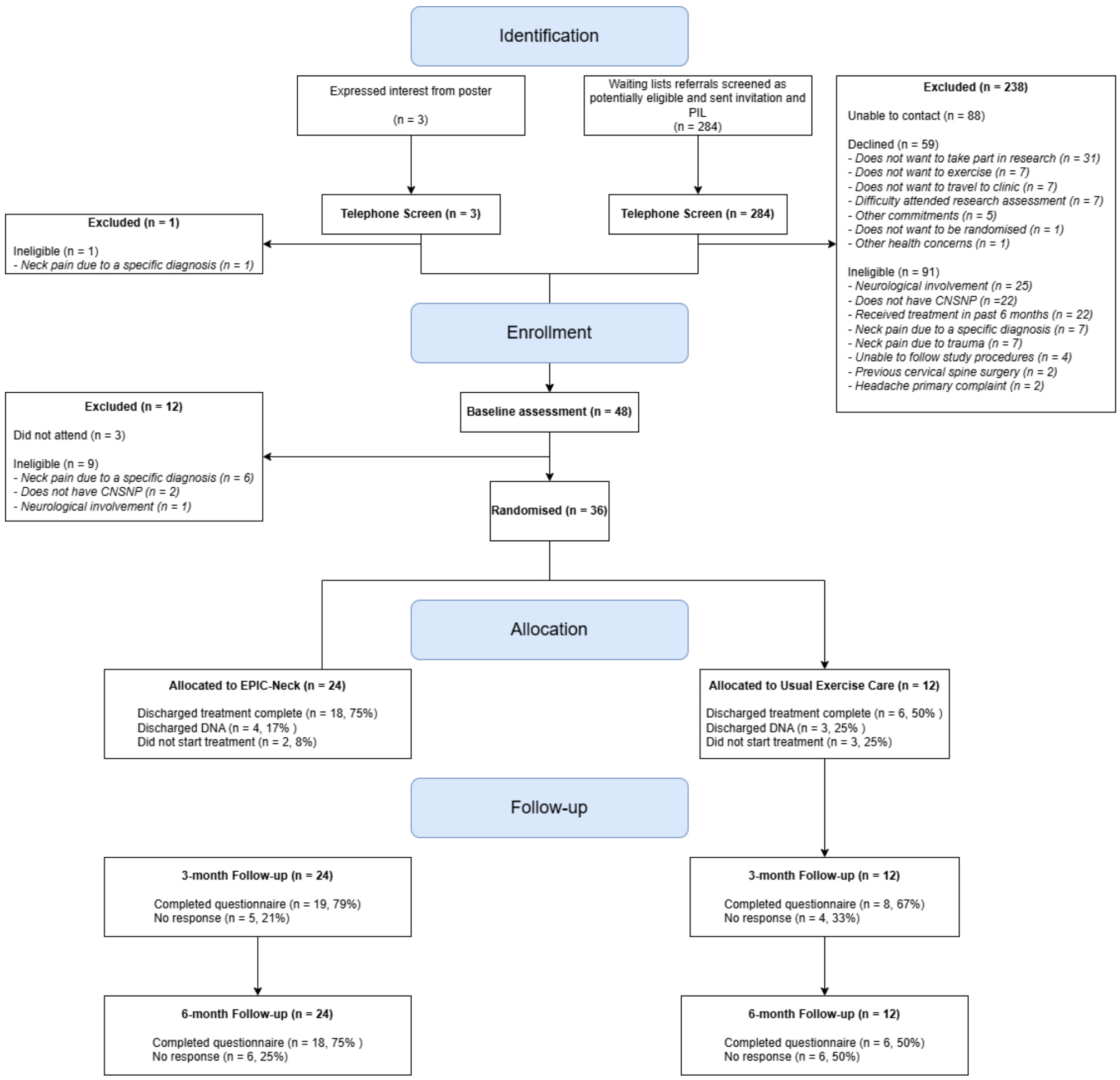

396x381mm (72 x 72 DPI)

BMJ Open

Co-developed neck exercise (EPIC-Neck) versus usual exercise care for people with chronic non-specific neck pain: a UK multicentre randomised feasibility trial

Journal:	*BMJ Open*
Manuscript ID	bmjopen-2026-120571.R3
Article Type:	Original research
Date Submitted by the Author:	10-Sep-2026
Complete List of Authors:	Price, Jonathan; Birmingham Community Healthcare NHS Foundation Trust, Musculoskeletal Physiotherapy Services; Birmingham Community Healthcare NHS Foundation Trust, Research and Innovation; University of Birmingham College of Life and Environmental Sciences, School of Sport, Exercise and Rehabilitation Sciences<br>Rushton, Alison; Western University, School of Physical Therapy<br>Ives, Natalie; University of Birmingham College of Medical and Dental Sciences, Institute of Applied Health Research<br>Bradbury, Marilyn; Birmingham Community Healthcare NHS Foundation Trust, Research and Innovation; University of Birmingham College of Life and Environmental Sciences, School of Sport, Exercise and Rehabilitation Sciences<br>Parmar, Priti ; Birmingham Community Healthcare NHS Foundation Trust, Research and Innovation<br>Greaves, Colin; University of Birmingham College of Life and Environmental Sciences, School of Sport, Exercise and Rehabilitation Sciences
**Primary Subject Heading**:	Rehabilitation medicine
Secondary Subject Heading:	Rehabilitation medicine
Keywords:	Exercise, Self-Management, Feasibility Studies, Behavior, Spine < ORTHOPAEDIC & TRAUMA SURGERY

SCHOLARONE™
MANUSCRIPTS

I, the Submitting Author has the right to grant and does grant on behalf of all authors of the Work (as defined in the below author licence), an exclusive licence and/or a non-exclusive licence for contributions from authors who are: i) UK Crown employees; ii) where BMJ has agreed a CC-BY licence shall apply, and/or iii) in accordance with the terms applicable for US Federal Government officers or employees acting as part of their official duties; on a worldwide, perpetual, irrevocable, royalty-free basis to BMJ Publishing Group Ltd ("BMJ") its licensees and where the relevant Journal is co-owned by BMJ to the co-owners of the Journal, to publish the Work in this journal and any other BMJ products and to exploit all rights, as set out in our licence.

The Submitting Author accepts and understands that any supply made under these terms is made by BMJ to the Submitting Author unless you are acting as an employee on behalf of your employer or a postgraduate student of an affiliated institution which is paying any applicable article publishing charge ("APC") for Open Access articles. Where the Submitting Author wishes to make the Work available on an Open Access basis (and intends to pay the relevant APC), the terms of reuse of such Open Access shall be governed by a Creative Commons licence – details of these licences and which Creative Commons licence will apply to this Work are set out in our licence referred to above.

Other than as permitted in any relevant BMJ Author's Self Archiving Policies, I confirm this Work has not been accepted for publication elsewhere, is not being considered for publication elsewhere and does not duplicate material already published. I confirm all authors consent to publication of this Work and authorise the granting of this licence.

For peer review only

TITLE PAGE

Co-developed neck exercise (EPIC-Neck) versus usual exercise care for people with chronic non-specific neck pain: a UK multicentre randomised feasibility trial

Internal Reference/Short Title: EPIC-Neck

IRAS number: 331102

Trial registration: ISRCTN81746901

AUTHORS

Jonathan Price[1,2,3*] - Jonathan.price2@nhs.net

Alison Rushton[4] - arushto3@uwo.ca

Natalie Ives[5] - n.j.ives@bham.ac.uk

Marilyn Bradbury[2,3] - marilyn.bradbury1@nhs.net

Priti Parmar[2] - priti.parmar2@nhs.net

Colin J Greaves[3] - c.j.greaves@bham.ac.uk

ABSTRACT

Objectives: To assess the feasibility of conducting a definitive randomised controlled trial of the EPIC-Neck programme versus usual exercise care for chronic non-specific neck pain, specifically in relation to recruitment, retention, delivery fidelity, acceptability and contamination.

For peer review only - http://bmjopen.bmj.com/site/about/guidelines.xhtml

For peer review only

Design: Multicentre, two-arm, parallel-group, individually randomised controlled feasibility trial with an embedded process evaluation.

Setting: Three National Health Service musculoskeletal services in England.

Participants: Adults (≥18 years) with chronic non-specific neck pain lasting >3 months.

Interventions: Participants were randomised (2:1) to EPIC-Neck (an individualised, evidence-based, theory-informed, co-designed exercise programme) or usual exercise care. Participants and outcome assessors were not blinded to allocation.

Feasibility objectives: Progression was assessed using predefined green–amber–red criteria across five domains: (1) recruitment rate; (2) retention rate (percentage returning the neck disability index at 6 months); (3) delivery fidelity (audio-recorded appointments scored on a 0-5 Dreyfus competence scale where ≥3 = acceptable); (4) acceptability to participants and physiotherapists (1-5 acceptability questionnaires where ≥4 = acceptable and interviews analysed deductively using framework analysis against the theoretical framework of acceptability); and (5) contamination (presence of EPIC-Neck components in usual care) scored using the same 0-5 Dreyfus competency scale as delivery fidelity.

Results: Between May 2024 and February 2025, 287 individuals were screened and 36 randomised (24 EPIC-Neck, 12 usual care), yielding a mean recruitment rate of 4.5 participants/month (green). Retention at 6 months was 67% (24/36) (red). Among participants who completed their allocated treatment, 6-month retention was 91% (22/24). Delivery fidelity was moderate (mean 2.4/5; amber), with only 14% of participants receiving EPIC-Neck at an acceptable level. Acceptability was generally

For peer review only

For peer review only - http://bmjopen.bmj.com/site/about/guidelines.xhtml

positive though interviews identified addressable concerns around intervention coherence (amber). Contamination was low (mean fidelity score for controls was 1.1/5; green).

Conclusions: EPIC-Neck shows potential for evaluation in a definitive trial, but progression is conditional on future refinement of the intervention and study procedures to address the key barriers to progression: retention, delivery fidelity and intervention coherence.

For peer review only

Trial registrations: ISRCTN81746901

Keywords: Neck Pain, Exercise Therapy, Self-Management, Feasibility Studies, Patient Acceptance of Health Care, Intervention Fidelity

Strengths and limitations

- This multicentre randomised feasibility trial used an embedded mixed-methods process evaluation, allowing integration of quantitative and qualitative data from participants and physiotherapists.
- The high proportion of audio-recorded appointments enabled a robust assessment of delivery fidelity and contamination using a structured scoring framework.
- Inclusive recruitment and consent procedures, including multilingual/video materials and interpreter support, were used to reduce barriers to participation.
- Physiotherapists delivered the intervention to relatively few participants, so some fidelity limitations may reflect early learning effects.

For peer review only - http://bmjopen.bmj.com/site/about/guidelines.xhtml

- Fidelity assessments were unblinded, and qualitative interviews were conducted by the intervention developer, creating potential for bias despite mitigation strategies.

Word count: 4131

For peer review only

For peer review only - http://bmjopen.bmj.com/site/about/guidelines.xhtml

BACKGROUND

Neck pain continues to pose a growing global health challenge. The age-standardised number of years lived with disability for neck pain has increased in recent years, rising by 0.12% per year from 2011–2016 and 0.42% per year from 2016–2019, despite advancements in healthcare.[1] Although all types of neck pain contribute to this burden, chronic non-specific neck pain is particularly impactful because symptoms are associated with greater pain, disability, psychological distress, reduced work capacity and healthcare use than acute neck pain.[2-6] Effective treatments are needed to reduce immediate and long-term impact.

Clinical guidelines and multiple systematic reviews support the use of exercise to improve chronic non-specific neck pain and disability.[7-15] However, effects are often modest, diminish over time,[10, 16] and may be limited by poor adherence.[10, 17-20] To address these limitations, the EPIC-Neck programme (EPIC: Exercise Prescription Improved through Co-design) was co-developed with patients and physiotherapists using Intervention Mapping.[21] EPIC-Neck differs from usual exercise care by integrating physiotherapist-prescribed exercise, guided by a biopsychosocial prescription framework with structured behaviour change strategies to support exercise effects and adherence.[21]

Before evaluating adherence, clinical effectiveness and cost-effectiveness of EPIC-Neck compared with usual exercise care, key uncertainties that could compromise a definitive trial needed to be addressed.[22] The Medical Research Council recommends that complex interventions, such as EPIC-Neck, undergo feasibility testing before definitive evaluation.[22] For EPIC-Neck, key uncertainties included

For peer review only - http://bmjopen.bmj.com/site/about/guidelines.xhtml

whether adults with chronic non-specific neck pain could be recruited and retained in an exercise-versus-exercise trial, whether EPIC-Neck was acceptable to patients and physiotherapists, whether key components could be delivered as intended, and whether the intervention remained sufficiently distinct from usual exercise care to avoid contamination between trial arms.

Aims and objectives

Aim: To determine the feasibility of a definitive trial evaluating the clinical and cost-effectiveness of EPIC-Neck compared with usual exercise care for adults with chronic non-specific neck pain.

Primary feasibility objectives, aligned directly with the prespecified traffic-light progression criteria (Table 1), were to assess:

- Mean participant recruitment;
- 6-month retention;
- Delivery fidelity of key EPIC-Neck components;
- Presence of key EPIC-Neck components in usual exercise care (contamination);
- Acceptability of EPIC-Neck to patients and physiotherapists.

Secondary feasibility objectives were to:

- Describe treatment engagement, including initiation, attendance, appointment time, treatment duration and completion;
- Assess safety;
- Describe characteristics of individuals who did and did not enrol;

For peer review only

- Assess completeness of planned definitive-trial outcome measures.

For peer review only

For peer review only - http://bmjopen.bmj.com/site/about/guidelines.xhtml

114 **Table 1 - Traffic light progression criteria**

	Green	Amber	Red	Feasibility outcomes
**Mean recruitment rate**	≥3 participants per month	1-2 participants per month	≤1 participants per month	**Green:** 4.5 participants per month, 95%CI [3.0, 6.1]
**Retention rate**	≥80%	70-79%	<70%	**Red:** 67%, 95% CI [49, 81]
**Delivery fidelity**	No areas of concern (most items ≥3)	Minor problems (≥ 50% of components between 1.5–3.0)	Major problems (most components < 1.5)	**Amber:** Overall mean score 2.4 (SD 1.3, 95% CI: 1.7, 3.1); 53% of components scored 1.5–3
**Contamination**	No EPIC-Neck components present in usual care	Minor overlap	Major overlap	**Green:** Mean checklist score 1.1/5 (SD 1.0, 95%CI: 0.6, 1.6); 1/17 components observed
**Acceptability**	Mean acceptability score of ≥ 4 and no concerns in interviews	Mean acceptability score 3 and/or minor concerns	Mean acceptability score ≤2 and/or major concerns	**Amber:** Mean 3.9 (SD 1.0, 95%CI: 3.7, 4.1); interview concerns were addressable

115 Green: No areas of concern
116 Amber: Minor modifications required before continuing to a definitive trial
117 Red: Major modifications required before continuing to a definitive trial
118 Abbreviation: CI: Confidence Interval; SD: Standard Deviation
119

For peer review only

METHODS

Reporting

This trial is reported in accordance with the CONSORT checklist for pilot/feasibility trials (Supplementary File 1) and the published protocol (Supplementary File 2).[23, 24]

Trial design

This was a multicentre, two-arm, parallel-group, individually randomised controlled feasibility trial. Participants were randomised in a 2:1 ratio to either the intervention group (EPIC-Neck programme) or usual exercise care. The feasibility trial evaluated outcomes important for deciding whether and how to progress to a definitive trial, including recruitment, retention, treatment engagement, acceptability, contamination, outcome-measure completion and safety. Embedded within the feasibility trial was a mixed-methods process evaluation, which included a multidimensional fidelity evaluation to understand EPIC-Neck training, delivery, receipt and enactment and to inform intervention and training refinements. This manuscript reports the feasibility outcomes relevant to progression to a definitive trial; the detailed multidimensional fidelity evaluation is reported separately. The trial was registered (ISRCTN81746901) and received ethical approval from South Central - Oxford B Research Ethics Committee (24/SC/0006).

Participant eligibility, identification and consent procedures

Participants were recruited from National Health Service (NHS) community-based and secondary care musculoskeletal services. Recruitment was originally planned across two NHS sites as described in the protocol; however, one site withdrew

For peer review only - http://bmjopen.bmj.com/site/about/guidelines.xhtml

during study set-up and two additional NHS musculoskeletal services were subsequently recruited. Adults (≥18 years) with chronic (≥3 months) non-specific neck pain were eligible. Non-specific neck pain was defined as pain or discomfort in the neck and/or shoulder girdle, with or without referred arm pain, for which no specific cause could be identified.[25, 26] Exclusions included recent physiotherapy within 6 months, previous cervical surgery, traumatic or specific neck pathology, neurological involvement, or inability to follow trial procedures. Full eligibility criteria are reported in the protocol.[24]

NHS clinical staff identified potential participants from waiting lists or trial posters and provided a Participant Information Leaflet (PIL). Potential participants completed telephone eligibility screening and, if eligible, attended a face-to-face baseline assessment with a research physiotherapist. Written informed consent was obtained for the feasibility trial and qualitative interviews, eligibility confirmed, and baseline assessments completed prior to randomisation. Trial materials were available in paper, electronic, and video formats, with English or Urdu audio narration, and professional interpreters supported screening and consent where required.[27, 28]

Randomisation

Randomisation was conducted via a secure web-based system (Sealed Envelope). The sequence was computer-generated using random permuted blocks (sizes 3 and 6) with a 2:1 allocation to EPIC-Neck. A 2:1 allocation ratio in favour of the intervention group was used to ensure sufficient intervention exposure and participant feedback to inform feasibility, acceptability and intervention refinement.[29]

For peer review only - http://bmjopen.bmj.com/site/about/guidelines.xhtml

Allocation was concealed through the automated system, which released the assignment only after baseline data entry by the research physiotherapist.

Blinding

Blinding of treating physiotherapists was not possible due to the nature of the intervention but cross-contamination was minimised by physiotherapists delivering either EPIC-Neck or usual exercise care, but not both. Participants and outcome assessors were not blinded in this feasibility trial. Blinding procedures will be considered, where appropriate, in the definitive trial.[30]

Physiotherapist consent procedures

Physiotherapists at participating sites provided written informed consent to deliver either EPIC-Neck or usual exercise care and to participate in the process evaluation (audio-recorded appointments/interviews).[24] Characteristics (age, gender, qualifications, years' experience, and postgraduate training) were collected by self-report before training and were not independently verified.

Interventions

EPIC-Neck (Intervention arm)

Full details of the EPIC-Neck programme are published elsewhere.[21] In brief, EPIC-Neck is a biopsychosocial, theory-informed intervention designed to support self-management of chronic neck pain through a home-based exercise programme. Physiotherapists prescribed exercises using a biopsychosocial framework to target participants' physical, psychological and social needs (Supplementary File 3, Figure S3-2). A behaviour change facilitation guide supported exercise engagement using

For peer review only - http://bmjopen.bmj.com/site/about/guidelines.xhtml

techniques such as goal setting, self-monitoring, action planning, social support and self-efficacy enhancement, delivered through patient-centred communication (Supplementary File 3, Figure S3-1).[31, 32] EPIC-Neck was delivered face-to-face by trained physiotherapists and supported by paper and digital patient-facing materials (workbook, exercise diary, exercise library, and friends-and-family leaflet). Non-exercise treatments (e.g. postural advice, manual therapy) were provided at the physiotherapist's discretion. Discharge was agreed collaboratively based on goals and self-management ability.

Physiotherapist training

Physiotherapists delivering EPIC-Neck completed a one-day, face-to-face training combining lectures, role play and behavioural skills practice.[33, 34] Ongoing support included a training manual, crib sheets, optional booster sessions, and peer/expert support.[33, 35] Audio-recordings of intervention delivery were used for fidelity evaluation, but real-time monitoring, feedback or delivery coaching were not provided during the trial.

Usual exercise care (Control arm)

The usual exercise care group received physiotherapy exercises as per the physiotherapists standard practice. Physiotherapists were not given any additional instruction or training. Non-exercise treatments (e.g. postural advice, manual therapy) were provided at the physiotherapist's discretion.

For peer review only

For peer review only - http://bmjopen.bmj.com/site/about/guidelines.xhtml

Outcomes

Feasibility progression criteria

Feasibility of progressing to a definitive trial was assessed across five prespecified domains: recruitment, 6-month retention, delivery fidelity, contamination, and acceptability. A traffic-light system with a priori thresholds was used to guide progression decision (Table 1).[36]

Recruitment

The mean number of participants randomised per month was calculated across the active recruitment period.[37] Thresholds (Table 1) were based on the estimated time required to recruit the anticipated definitive trial sample size of 248 participants across 5 musculoskeletal services.[37]

Retention

Return of the Neck Disability Index (NDI) at 6 months was used as the retention criterion, selected as an indicator of likely follow-up at the planned 12-month primary endpoint in a definitive trial.[38, 39] Thresholds (Table 1) were informed by guidance that ≥80% retention represents low risk of attrition bias and comparable musculoskeletal trials reporting approximately 70% retention.[40-45]

Delivery fidelity

All EPIC-Neck appointments were audio-recorded and coded against a pre-defined 17-item checklist of key EPIC-Neck components (Supplementary File 3-Table S3-1).[46, 47] Each component was rated using a 0–5 Dreyfus delivery competence scale,

For peer review only

where 0 indicated absent delivery, ≥3 acceptable delivery and 5 optimal delivery.[48] Progression thresholds (Table 1) were based on the distribution of checklist scores across components, with ≥3 used as the threshold for acceptable delivery, consistent with previous fidelity research using this approach.[47, 49, 50]

Contamination

Presence of EPIC-Neck components within usual exercise care was assessed using audio-recorded usual care appointments, coded against the same 17-item checklist and 0–5 Dreyfus competence scale used for EPIC-Neck delivery fidelity.[51, 52] Thresholds (Table 1) reflected the extent to which EPIC-Neck components appeared in usual care and whether this threatened distinction between trial arms. Final progression judgements were made through discussion within the study team (JP, MB and CG), informed by the checklist scores. Interviews with usual-care physiotherapists were undertaken to further characterise usual exercise care and will be reported elsewhere.

Acceptability

EPIC-Neck acceptability to participants and physiotherapists was evaluated using a convergent mixed-methods approach informed by the Theoretical Framework of Acceptability (TFA).[53, 54] EPIC-Neck participants completed 3 and 6-month questionnaires covering TFA domains of affective attitude, intervention coherence, perceived effectiveness, opportunity costs and self-efficacy. Items were rated on 1–5 Likert scales and interpreted at face value according to the questionnaire response anchors, where scores of 4–5 indicated positive acceptability, 3 indicated neutrality and 1–2 indicated negative acceptability.[53] Qualitative acceptability was explored

For peer review only

For peer review only - http://bmjopen.bmj.com/site/about/guidelines.xhtml

through semi-structured interviews with participants (post-discharge) and physiotherapists (end of trial), using patient and public involvement and engagement (PPIE)-informed topic guides mapped to all seven TFA domains. Interviews were conducted by telephone, video call or face-to-face and audio-recorded. Progression criteria (Table 1) combined these quantitative thresholds with qualitative findings. Final progression judgements were made through discussion between JP and CG, informed by questionnaire scores and qualitative findings. Any disagreements were resolved through discussion with a third study team member.

Allocated treatment engagement

Allocated treatment engagement was described using initiation, attendance, did-not-attend appointments, total appointment time, treatment duration and treatment completion. Treatment initiation was defined as attendance at ≥1 appointment, and treatment completion as collaboratively agreed discharge. Total appointment time was calculated from available audio-recordings, and treatment duration as weeks from initial assessment to final attended appointment.

Inclusivity

Demographic characteristics of randomised and non-randomised individuals identified from screening logs were compared. Variables included age, gender, ethnicity, first language, interpreter need and deprivation, measured using Index of Multiple Deprivation (IMD) decile. The IMD is a composite measure of area-level deprivation in England, incorporating domains including income, employment, education, health, crime, housing, and living environment, with deciles ranging from 1 (most deprived 10%) to 10 (least deprived 10%).

For peer review only

For peer review only - http://bmjopen.bmj.com/site/about/guidelines.xhtml

Feasibility of trial outcome measures

Planned definitive-trial outcome measures were collected at baseline, 3 and 6 months post-randomisation to assess data collection feasibility and completeness. Baseline data were collected face-to-face, and follow-up questionnaires were completed by post, with telephone completion and interpreter support available where required. A £20 voucher was offered for returning all three questionnaires. Outcomes were selected based on existing literature, PPIE input and the EPIC-Neck logic model.[21] The planned definitive trial primary outcome was the Neck Disability Index, which is widely used with good psychometric properties and a minimum clinically important difference of 5.5 points.[55, 56] Secondary outcome measures include pain (Visual Analogue Scale (VAS));[57] health-related quality of life (EQ-5D-5L);[58] mental well-being (Hospital Anxiety and Depression Scale (HADS));[59] sleep quality (The Sleep Condition Indicator);[60] specific activity limitations (Patient Specific Functional Scale (PSFS)[61] and social/relationship impact (Relationships and Self-Worth domains of Assessment of Quality-of-Life 8D).[62, 63] Candidate mediators were pain beliefs (Single item Survey of Pain Attitudes Questionnaire (SOPA));[64] pain related fear (Tampa Scale of Kinesiophobia-13 (TSK-13));[65] pain self-efficacy (Pain self-efficacy questionnaire (PSEQ));[66] and night pain (VAS).[57]

For peer review only

Data analysis

Quantitative data were analysed using IBM SPSS v25 and were primarily descriptive, using means, standard deviations (SD), ranges, proportions and 95% confidence intervals (CI), as appropriate. For delivery fidelity and contamination, two researchers (JP, MB) independently coded an initial sample of audio-recordings and iteratively calibrated scoring using further recordings until ≥75% of ratings were

For peer review only - http://bmjopen.bmj.com/site/about/guidelines.xhtml

within one point.[49, 67] Remaining recordings were coded by JP. Fidelity and contamination scores were summarised descriptively at component-, participant- and study-level. Treatment initiation, completion and discharge following non-attendance were summarised using the number randomised to each group as the denominator. Among participants attending ≥1 appointment, attendance, non-attendance, total appointment time and treatment duration were summarised descriptively.

Demographic characteristics of randomised and non-randomised individuals were compared using Pearson's chi-square tests ($p < 0.05$). As this was a feasibility trial, no inferential between-group comparisons were planned or undertaken for the planned definitive-trial outcome measures.[68]

Qualitative acceptability data were analysed using deductive framework analysis.[69, 70] Interviews were audio-recorded, transcribed verbatim and coded against TFA domains in NVivo 12 Plus (QSR International). Data were summarised in a domain-by-case matrix to compare patterns within and across cases for participants and physiotherapists separately. Minor (Amber), major (Red) or no (Green) acceptability concerns were identified within each domain. All interviews and qualitative analysis were completed by JP, an experienced physiotherapist and lead developer of the EPIC-Neck programme. Reflexivity was addressed by acknowledging prior professional relationships with some physiotherapists and none with participants. To support analytic rigour and consistency, a subset of domain coding was reviewed within the research team. Quantitative and qualitative acceptability findings were triangulated in a domain-level table, with domain-level acceptability ratings combined to provide an overall judgement.

For peer review only - http://bmjopen.bmj.com/site/about/guidelines.xhtml

Sample size

Target recruitment was 45 participants, based on a precision calculation to estimate retention with sufficient accuracy to inform a future definitive trial. Assuming 80% retention in this feasibility trial, approximately 43 participants were required to estimate retention with a 95% CI of ±12%; this was rounded to 45 participants.[44, 71] No interim analyses or stopping rules were planned.

Semi-structured interviews were conducted with a purposive sample of 12 EPIC-Neck participants, sampling for variation in ethnicity, socioeconomic deprivation, and baseline neck disability to reflect potentially under-served groups and diverse pain experiences.[72-74] All physiotherapists delivering the EPIC-Neck programme were invited to be interviewed.

Patient and public involvement and engagement

The trial protocol, intervention development, outcome selection, inclusivity design, trial materials and dissemination were informed by a PPIE group (n = 17). Two PPIE members were part of the Study Management Group. PPIE contributions are reported in more detail in Supplementary File 4 (GRIPP2-SF).[75]

RESULTS

Screening and recruitment

Recruitment took place between 29 May 2024 and 10 February 2025 (Figure 1). Of 287 individuals invited, 36 were randomised (24 EPIC-Neck; 12 usual exercise care), giving an uptake rate of 12.5% (95% CI 8.7–16.4). Recruitment stopped before the planned target of 45 participants was reached because the available study time and

For peer review only - http://bmjopen.bmj.com/site/about/guidelines.xhtml

funding period ended. At the point recruitment stopped, sufficient data had been collected to evaluate the prespecified feasibility objectives.

Participant characteristics

Participants had a mean age of 45.9 years (range 19–82), with a similar number of males and females (Table 2). Overall, 41.7% were of Asian or Asian British ethnic origin, and 72.2% lived in the most deprived IMD deciles.

For peer review only

For peer review only - http://bmjopen.bmj.com/site/about/guidelines.xhtml

Table 2 – Patient participant baseline characteristics

Demographic characteristic	EPIC-Neck n=24		Usual Exercise Care n=12		Full sample n=36	
	n	%	*n*	%	*n*	%
**Gender**						
Female	11	45.8%	5	41.7%	16	44.4%
Male	13	54.2%	7	58.3%	20	55.6%
**Age**						
18-30	3	12.5%	3	25.0%	6	16.7%
31-40	7	29.2%	1	8.3%	8	22.2%
41-50	3	12.5%	3	25.0%	6	16.7%
51-60	7	29.2%	3	25.0%	10	27.8%
61+	4	16.7%	2	16.7%	6	16.7%
**Ethnicity**						
**Asian or Asian British**	9	37.5%	6	50.0%	15	41.7%
Indian	2	8.3%	1	8.3%	3	8.3%
Pakistani	7	29.2%	5	41.7%	12	33.3%
**Black or Black British**	3	12.5%	2	16.7%	5	13.9%
African	1	4.2%	1	8.3%	2	5.6%
Caribbean	1	4.2%	0	0.0%	1	2.8%
Other	1	4.2%	1	8.3%	2	5.6%
**Mixed**	1	4.2%	1	8.3%	2	5.6%
White & Black Caribbean	1	4.2%	1	8.3%	2	5.6%
**White**	10	41.7%	3	25.0%	13	36.1%
British	9	37.5%	3	25.0%	12	33.3%
Irish	1	4.2%	0	0.0%	1	2.8%
**Other**	1	4.2%	0	0.0%	1	2.8%
Arab	1	4.2%	0	0.0%	1	2.8%
**IMD decile**						
Most deprived (deciles 1-3)	19	79.2%	7	58.3%	26	72.2%
Middle (deciles 4-7)	4	16.7%	5	41.7%	9	25.0%
Least deprived (deciles 8-10)	1	4.2%	0	0.0%	1	2.8%
**Marital status**						
Living with partner	6	25.0%	1	8.3%	7	19.4%
Married/civil union	13	54.2%	5	41.7%	18	50.0%
Separated/divorced	1	4.2%	1	8.3%	2	5.6%
Unmarried (never married)	4	16.7%	5	41.7%	9	25.0%
**Cohabiting status**						
Cohabiting	24	100.0%	10	83.3%	34	94.4%
Living alone	0	0.0%	2	16.7%	2	5.6%
**Neck pain location**						
Left	11	45.8%	9	75.0%	20	55.6%

For peer review only

For peer review only - http://bmjopen.bmj.com/site/about/guidelines.xhtml

Right	11	45.8%	6	50.0%	17	47.2%
Middle	7	29.2%	7	58.3%	14	38.9%
Base of skull	2	8.3%	1	8.3%	3	8.3%

Abbreviations: IMD - Index of multiple deprivation

Feasibility objectives

Mean recruitment rate

Mean recruitment rate was 4.5 participants per month (95% CI: 3.0, 6.1; 36 participants over 8 months), exceeding the “Green” progression criterion (Table 1).

Retention rate

At six months, 67% of participants returned the NDI questionnaire (24/36; 95% CI: 49, 81), meeting the “Red” criterion (Table 1). Retention was 75% in EPIC-Neck (18/24) and 50% in usual exercise care (6/12) (Figure 1). All 3-month non-responders were also non-responders at 6 months (9/9). Among 6-month non-responders, 41% (5/12) had not initiated treatment and 41% (5/12) were discharged for non-attendance. Retention among treatment completers was high (22/24; 91%).

Delivery fidelity

Fidelity was scored for 76/80 EPIC-Neck appointments; four were excluded due to missing recordings (n=2) or a participant developing neurological symptoms (n=2). Overall mean fidelity was 2.4/5 (SD 1.3; 95% CI: 1.7, 3.1). Component scores ranged from 0.9 to 3.5, with 6/17 components (35%) delivered acceptably (≥3) and 9/17 (53%) scoring 1.5–<3.0 (Table 3). Only 3/22 participants (14%) had a mean score ≥3 (participant means 1.3–3.2). Based on the study-level mean and component-level distribution, delivery fidelity met the Amber progression criterion (Table 1).

For peer review only

For peer review only - http://bmjopen.bmj.com/site/about/guidelines.xhtml

Table 3 – Delivery fidelity scores by component

	Mean Score (SD)	
**Component**	**EPIC-Neck**	**UEC**
01 - Agree outcome goals	2.0 (1.3)	0.9 (0.9)
02 - Actively involve the patient	3.1 (0.7)	1.2 (0.4)
03 - Improve motivation to perform exercise (PO1) - Enhance perceived importance and safety	2.1 (1.0)	1.4 (0.6)
04 - Improve motivation to perform exercise (PO1) - Improve self-efficacy	3.5 (0.8)	3.0 (1.1)
05 - Improve motivation to perform exercise (PO1) - Engage social support	1.8 (1.1)	0.4 (0.5)
06 - Set a plan to perform exercise (PO1)	2.3 (1.0)	1.0 (0.0)
07 - Encourage self-monitoring	3.3 (0.9)	0.0 (0.0)
08 - Review progress	2.7 (0.5)	1.6 (0.3)
09 - Support problem solving	1.3 (0.7)	0.8 (0.5)
10 - Introduce adapting and progressing (or regression) exercise (PO2)	1.6 (1.1)	0.2 (0.4)
11 - Introduce flare-up exercise (PO3)	1.5 (1.0)	0.1 (0.3)
12 - Introduce general exercise +/- secondary prevention exercise (PO4)	0.9 (0.9)	0.1 (0.3)
13 - Evaluate self-management and discharge suitability	2.0 (1.2)	0.8 (0.9)
14 - Identify short-term outcomes and exercise objectives	3.1 (0.8)	1.0 (0.0)
15 - Home exercise programme – Normalising movement	3.5 (0.7)	2.0 (1.0)
16 - Home exercise programme – Improving tolerance	2.7 (1.6)	2.2 (0.7)
17 - Exercise review	3.0 (1.2)	2.2 (1.1)
	2.4 (1.3)	1.1 (1.0)

Abbreviations: PO – Performance Objective; SD – Standard deviation; UEC – Usual Exercise Care

For peer review only

Contamination

Intervention content was scored for 29/32 usual exercise care appointments; three were excluded due to missing recordings. Mean checklist score was 1.1/5 (SD 1.0; range 0.6–1.5, 95% CI: 0.6, 1.6), with 1/17 components (‘Improve self-efficacy’) delivered in a manner consistent with EPIC-Neck (Table 3). This met the “Green” contamination criterion (Table 1).

Acceptability

Acceptability findings were similar at 3 and 6 months, therefore only the 6-month findings are considered in the integrated acceptability assessment. Both 3- and 6-month data are available in Supplementary File 5. Eighteen EPIC-Neck participants returned acceptability questionnaires (Table 4). Mean scores met the pre-defined ≥4/5 threshold for affective attitude, intervention coherence, perceived effectiveness, and self-efficacy. Opportunity costs, reflecting the extent to which doing the exercises interfered with other priorities, were neutral (mean 3.3, SD 1.0; 95% CI: 2.8, 3.9), and overall acceptability was 3.9 (SD 1.0; 95% CI: 3.7, 4.1).

For peer review only

For peer review only - http://bmjopen.bmj.com/site/about/guidelines.xhtml

386 **Table 4 – Acceptability summary**

Domain	Patient Questionnaire (n = 18) mean (SD) [95%CI]	Patient Interviews (n = 12)	Physiotherapist Interviews (n = 5)	Integrated summary
**Affective attitude**	**Green:** 4.1 (0.8) [3.7, 4.5]	**Amber:** Satisfaction with individualised, targeted approach and associated benefits, though some expressed negative feelings about physical side effects and general exercise; overall, positive outcomes outweighed lack of enjoyment.	**Green:** Enjoyed structured approach and behaviour change techniques	**Amber:** Address patient concerns about physical side effects and attitudes to general exercise
**Burden**	NA	**Amber:** Exercises that were complex or required specific space/positioning increased effort and were often omitted; time burden varied, with integration into daily routines reducing effort.	**Amber:** Cognitive and time burden due to intervention depth, volume and changing practice which improved with exposure.	**Amber:** Address patient burden related to exercise duration and complex exercises, and physiotherapist burden related to intervention depth and change in clinical practice.
**Ethicality**	NA	**Amber:** Alignment with values on individualised, non-pharmacological management, but misalignment for some with values around shared decision-making, dislike of routines, and preference for interpersonal support over written or video resources.	**Amber:** Alignment with individualised biopsychosocial patient centred care beliefs but misaligned for some regarding goal setting and shared decision-making beliefs.	**Amber:** Address patient misalignment with shared decision-making and resource preferences, and physiotherapist misalignment with goal-setting and shared decision-making.
**Intervention coherence**	**Green:** 4.1 (1.1) [3.6, 4.7]	**Red:** Patients understood basic exercise performance but had limited grasp of purpose, benefits, and progression strategies; understanding of flare-up management and general exercise plans was inconsistent	**Amber:** Coherent with familiar components, but low with new or more complex components – improved with practice but remained limited	**Red:** Improve patients' understanding of programme rationale, progression strategies, and flare-up management, and strengthen physiotherapists' understanding of how to deliver

For peer review only - http://bmjopen.bmj.com/site/about/guidelines.xhtml

		and rarely aligned with EPIC-Neck standards.		newer or more complex components.
**Opportunity costs**	**Amber:** 3.3 (1.0) [2.8, 3.9]	**Amber:** Most reported no trade-offs, but some described minor sacrifices outweighed by benefits, while others de-prioritised exercise in favour of other activities.	**Green:** Minimal trade-offs	**Amber:** Address patient de-prioritisation of exercise
**Perceived effectiveness**	**Green:** 4.1 (1.2) [3.5, 4.7]	**Green:** Consistent benefits in pain, disability and function, with sleep, mental well-being, and relationships improvements noted where limited at baseline	**Amber:** Overall perceived as effective with reservations regarding goal setting, social support and the patient resources	**Amber:** Address physiotherapist concerns about goal setting, social support, and patient resources.
**Self-efficacy**	**Green:** 4.1 (0.9) [3.7, 4.5]	**Amber:** Confident with basic exercises and self-managing flare-ups, but confidence declined with complexity, progression strategies, and general exercise citing fear of harm	**Amber:** Confident with familiar components, but low with new or more complex components – improved with practice but remained limited	**Amber:** Address patient confidence with complex exercises, safety, progression strategies, and general exercise; and physiotherapist confidence with new or complex components.
**Overall acceptability**	**Amber:** 3.9 (1.0) [3.7, 4.1]	-	-	**Amber:** Broadly acceptable with addressable concerns, particularly related to intervention coherence; further refinement and re-testing are required before a definitive trial.

387 Green: No areas of concern, mean acceptability score of ≥ 4 and no areas of concern identified in qualitative interviews

388 Amber: Minor problems that require attention before continuing to a definitive trial, mean acceptability score 3 and/or minor
389 problems identified in qualitative interviews

390 Red: Major problems that require urgent attention before continuing to a definitive trial, mean acceptability score ≤2 and/or major
391 problems identified in qualitative interviews

392 Abbreviations: SD – Standard deviation

For peer review only - http://bmjopen.bmj.com/site/about/guidelines.xhtml

393 Interviews were completed with 12 participants and 5 physiotherapists (Table 5).
394 Qualitative analysis (Table 4; full details are in Supplementary File 5) indicated no
395 participant concerns about intervention effectiveness. Addressable concerns were
396 identified in other TFA domains, particularly intervention coherence, defined here as
397 participants' understanding of the intervention and how it was intended to work.
398 Participants showed limited understanding of the exercise rationale and performance
399 objectives (exercise progression/regression, flare-up management, and general
400 exercise). Physiotherapists reported positive affective attitude and minimal
401 opportunity costs but had addressable concerns around coherence and self-efficacy
402 when delivering newer or more complex components.

403 **Table 5 – Interviewed patient and physiotherapist characteristics**

	n	%
**Patient participants (n = 12)**		
**Gender**		
Female	4	33.3%
Male	8	66.7%
**Age**		
18-30	1	8.3%
31-40	3	25.0%
41-50	2	16.7%
51-60	3	25.0%
61+	3	25.0%
**Ethnicity**		
**Asian or Asian British**	**3**	**25.0%**
Indian	0	0.0%
Pakistani	3	25.0%
**Black or Black British**	**2**	**16.7%**
African	0	0.0%
Caribbean	1	8.3%
Other	1	8.3%
**Mixed**	**1**	**8.3%**
White & Black Caribbean	1	8.3%
**White**	**5**	**41.7%**
British	5	41.7%
Irish	0	0.0%

For peer review only - http://bmjopen.bmj.com/site/about/guidelines.xhtml

**Other**	**1**	**8.3%**
Arab	1	8.3%
**IMD decile**		
Most deprived (deciles 1-3)	10	83.3%
Middle (deciles 4-7)	2	16.7%
Least deprived (deciles 8-10)	0	0.0%
**Neck pain related disability (NDI)**		
No disability	2	16.7%
Mild disability	5	41.7%
Moderate disability	5	41.7%
Severe disability	0	0.0%
Complete disability	0	0.0%
**Physiotherapist (n = 5)**		
**Gender**		
Female	3	60%
Male	2	40%
**Age**		
20-29	1	20%
30-39	1	20%
40-49	1	20%
50-59	2	40%
**Highest qualification**		
Bachelor's degree	5	100%
**Years experience treating chronic non-specific neck pain**		
0-2	2	40%
3-5	0	0%
6-10	2	40%
11-20	1	20%
**Postgraduate training areas**		
Neck pain assessment and treatment	3	60%
Exercise prescription	3	60%
Behaviour change	2	40%
Psychologically informed practice	1	20%
Patient-centred communication techniques	3	60%

Abbreviations: IMD – Index of multiple deprivation

Integrated interpretation (Table 4) showed that, although questionnaires suggested good patient acceptability across most domains, interviews identified coherence gaps for both participants and physiotherapists. Although intervention coherence was identified as a substantial concern, the overall acceptability judgement reflected findings across all TFA domains rather than any single domain. Overall, EPIC-Neck

For peer review only - http://bmjopen.bmj.com/site/about/guidelines.xhtml

was acceptable, with addressable concerns, meeting the “Amber” progression criterion.

Allocated treatment engagement

Treatment was not initiated by 2/24 EPIC-Neck participants (8%) and 3/12 usual exercise care participants (25%). Treatment was completed by 18/24 EPIC-Neck participants (75%) and 6/12 usual exercise care participants (50%). A further 4/24 EPIC-Neck participants (17%) and 3/12 usual exercise care participants (25%) initiated treatment but were discharged following non-attendance.

Among participants attending ≥1 appointment, both groups attended a mean of 3.6 appointments: EPIC-Neck, SD 1.4, range 1–7; usual exercise care, SD 2.6, range 1–9. Mean DNA appointments were 0.7 in EPIC-Neck (SD 0.8, range 0–3) and 1.1 in usual exercise care (SD 1.3, range 0–3). Mean total appointment time was 1 hour 55 minutes (SD 56 minutes, range 27 minutes to 4 hours 7 minutes) in the EPIC-Neck group and 1 hour 30 minutes (SD 52 minutes, range 26 minutes to 2 hours 42 minutes) in the usual exercise care group. Mean treatment duration was 15.9 weeks (SD 8.7, range 0–31) in the EPIC-Neck group and 11.3 weeks (SD 10.0, range 0–29) in the usual exercise care group.

Safety

No exercise-related serious adverse events were reported. One participant experienced a temporary worsening of symptoms (related, non-serious), and one experienced transient syncope at work (unrelated, serious).

For peer review only - http://bmjopen.bmj.com/site/about/guidelines.xhtml

431 Inclusivity

432 No statistically significant differences were observed for any demographic
433 characteristic between randomised and non-randomised individuals (Table 6).

434 **Table 6 - Demographics randomised vs nonrandomised**

Demographic characteristic	Nonrandomised (n=251)		Randomised (n=36)		P-value (from Pearson's Chi-Squared Test)
	n	%	*n*	%	
**Gender**					p = 0.110
Female	147	58.6%	16	44.4%	
Male	104	41.4%	20	55.6%	
**Age**					p = 0.445
18-30	33	13.1%	6	16.7%	
31-40	46	18.3%	8	22.2%	
41-50	54	21.5%	6	16.7%	
51-60	47	18.7%	10	27.8%	
61+	71	28.3%	6	16.7%	
**Ethnicity**					p = 0.549
Asian or Asian British	95	37.8%	11	30.6%	
Black or Black British	16	6.4%	5	13.9%	
Mixed	5	2.0%	1	2.8%	
White	73	29.1%	11	30.6%	
Other	7	2.8%	0	0.0%	
Not known	55	21.9%	8	22.2%	
**First language**					p = 0.264
English	84	33.5%	9	25.0%	
Other	50	19.9%	5	13.9%	
Not known	117	46.6%	22	61.1%	
**Interpreter required**					p = 0.434
No	227	90.4%	34	94.4%	
Yes	24	9.6%	2	5.6%	
**IMD decile**					p = 0.761
Most deprived (Deciles 1-3)	193	76.9%	27	75.0%	
Middle (Deciles 4-7)	46	18.3%	8	22.2%	
Least deprived (Deciles 8-10)	12	4.8%	1	2.8%	

For peer review only - http://bmjopen.bmj.com/site/about/guidelines.xhtml

435 Note: For consistency, demographic data were taken from the screening logs, as
436 these could differ from self-reported baseline questionnaire data for randomised
437 participants.

438 Abbreviations: IMD – Index of multiple deprivation

439 Feasibility of trial outcome measures

440 Summary statistics (means, SDs, 95% CIs) for outcomes and candidate mediators
441 are shown in Table 7. The PSFS was not completed at baseline by two participants
442 who reported no functional limitations. All other measures had 100% baseline return,
443 and 75% and 67% return at 3 and 6 months, respectively. Return rates and
444 item-level completeness are in Supplementary File 3–Table S3-2.

445 **Table 7 - Descriptive summary of planned definitive trial outcome and**
446 **candidate mediator measures**

Outcome Measure	EPIC-Neck			Usual Exercise Care		
	n	Mean (SD)	95%CI	n	Mean (SD)	95%CI
**Planned definitive trial primary outcome measure**						
Neck pain-related disability – NDI (0 = best, 50 = worst)						
Baseline	24	14.13 (7.12)	11.12, 17.13	12	16.58 (8.84)	10.97, 22.20
3-Month	19	14.21 (10.97)	8.92, 19.50	8	16.50 (10.30)	7.89, 25.11
6-Month	18	12.39 (12.79)	6.03, 18.75	6	16.67 (12.47)	3.58, 29.75
**Planned definitive trial secondary outcome measures**						
Neck pain – VAS (0 = best, 10 = worst)						
Baseline	24	5.63 (1.86)	4.84, 6.41	12	5.67 (2.31)	4.20, 7.13
3-Month	19	4.42 (2.87)	3.04, 5.81	8	4.25 (2.55)	2.12, 6.38
6-Month	18	3.50 (2.64)	2.19, 4.81	6	4.00 (2.28)	1.61, 6.39
Functional and activity impact – PSFS (0 = worst, 10 = best)						
Baseline	22	5.09 (1.77)	4.31, 5.88	12	4.50 (2.43)	2.96, 6.04
3-Month	17	5.47 (2.48)	4.20, 6.74	8	4.75 (2.55)	2.62, 6.88
6-Month	16	6.69 (2.65)	5.27, 8.10	6	5.17 (2.93)	2.10, 8.24
Sleep – SCI (0 = worst, 32 = best)						
Baseline	24	16.50 (6.49)	13.76, 19.24	12	13.83 (6.39)	9.77, 17.90
3-Month	19	16.84 (9.55)	12.24, 21.44	8	13.88 (8.11)	7.09, 20.66
6-Month	18	18.72 (9.89)	13.80, 23.64	6	15.00 (6.60)	8.07, 21.93
Mental wellbeing – HADS (0 = best, 42 = worst)						
Baseline	24	12.46 (7.87)	9.14, 15.78	12	16.00 (10.59)	9.27, 22.73
3-Month	19	14.32 (10.26)	9.37, 19.26	8	16.50 (14.28)	4.56, 28.44
6-Month	18	12.89 (10.36)	7.74, 18.04	6	12.67 (12.34)	-0.28, 25.62
Relationships and social function – AQoL (0 = worst, 100 = best)						
Baseline	24	74.63 (17.21)	67.36, 81.89	12	70.75 (23.69)	55.70, 85.80

For peer review only - http://bmjopen.bmj.com/site/about/guidelines.xhtml

3-Month	19	71.42 (20.87)	61.36, 81.48	8	67.13 (27.30)	44.30, 89.95
6-Month	18	75.56 (20.56)	65.33, 85.78	6	68.83 (23.95)	43.70, 93.97
Health related quality of life – EQ-5D-5L (-0.594= worst, 1 = best)						
Baseline	24	0.65 (0.24)	0.54, 0.75	12	0.60 (0.26)	0.43, 0.77
3-Month	19	0.65 (0.32)	0.49, 0.80	8	0.62 (0.22)	0.44, 0.81
6-Month	18	0.65 (0.36)	0.47, 0.83	6	0.63 (0.20)	0.43, 0.84
**Potential measures of mediation**						
Night pain – NPRS (0 = best, 10 = worst)						
Baseline	24	5.58 (2.75)	4.42, 6.74	12	5.42 (2.35)	3.92, 6.91
3-Month	19	3.95 (2.84)	2.58, 5.32	8	4.63 (2.56)	2.48, 6.77
6-Month	18	3.28 (2.87)	1.85, 4.70	6	4.83 (3.13)	1.55, 8.11
Pain self-efficacy – PSEQ (0 = worst, 60 = best)						
Baseline	24	40.29 (12.67)	34.94, 45.64	12	36.33 (14.30)	27.25, 45.42
3-Month	19	44.53 (15.74)	36.94, 52.11	8	39.25 (13.95)	27.59, 50.91
6-Month	18	47.11 (16.05)	39.13, 55.09	6	41.17 (15.82)	24.57, 57.77
Pain beliefs – SOPA (0 = best, 28 = worst)						
Baseline	24	14.71 (3.63)	13.18, 16.24	12	13.50 (4.15)	10.87, 16.13
3-Month	19	13.95 (4.79)	11.64, 16.26	8	10.63 (3.38)	7.80, 13.45
6-Month	18	13.72 (3.83)	11.82, 15.63	6	10.50 (2.43)	7.95, 13.05
Fear of movement – TSK-13 (13 = best, 52 = worst)						
Baseline	24	27.13 (8.46)	23.55, 30.70	12	28.58 (9.64)	22.46, 34.71
3-Month	19	26.53 (9.77)	21.82, 31.23	8	29.50 (10.32)	20.87, 38.13
6-Month	18	24.44 (9.43)	19.75, 29.13	6	31.17 (8.75)	21.98, 40.35

Abbreviations: AQoL - Assessment of Quality-of-Life (relationships and self-worth domains only; HADS - Hospital Anxiety and Depression Scale (Anxiety and depression scores combined); NDI – Neck disability index; PSEQ – Pain self-efficacy questionnaire; PSFS – Patient specific functional scale; SCI – Sleep condition indicator; SD – Standard deviation; SOPA – Survey of Pain Attitudes Questionnaire (single item version); TSK - Tampa Scale of Kinesiophobia; VAS – Visual analogue scale

Note: Table 7 reports descriptive statistics only, to assess feasibility of outcome-measure collection, completeness and score distributions for a future definitive trial. No inferential between-group comparisons were planned or undertaken.

Progression to a definitive trial

Table 1 summarises feasibility findings against the prespecified traffic-light criteria: Green for recruitment and contamination; Amber for delivery fidelity and acceptability; Red for retention. Overall, progression was considered feasible with modification, specifically to strengthen retention, delivery fidelity, and intervention coherence.

For peer review only - http://bmjopen.bmj.com/site/about/guidelines.xhtml

DISCUSSION

This feasibility trial aimed to determine whether a definitive trial evaluating the clinical and cost-effectiveness of EPIC-Neck for chronic non-specific neck pain is feasible. The feasibility findings were mixed. Recruitment and contamination met Green progression criteria, indicating no major concerns in these domains. However, retention met the Red criterion and delivery fidelity met the Amber criterion, indicating important feasibility concerns that require resolution before a definitive trial. Acceptability to both patients and physiotherapists was broadly positive, but qualitative findings identified addressable concerns within the TFA domain of intervention coherence, particularly participants' understanding of the intervention rationale and physiotherapists' clarity around delivering newer or more complex components.

Recruitment was feasible, but retention posed modifiable challenges

Despite not reaching the estimated sample size, 4–5 participants were randomised per month, exceeding the green progression criteria. In contrast, 6-month retention was 67%. This is below the ≥80% target associated with lower risk of attrition bias and below the minimum 70% threshold prespecified for progression, raising concerns about whether current retention strategies would be sufficient in a definitive trial.[40-44]

Standard retention strategies were used (linguistic support, pre-paid envelopes, reminders, financial incentives)[43, 76, 77] and while further optimisation may help (electronic and shorter questionnaires, increased incentives)[43, 78] our findings suggest that retention may be influenced by treatment allocation and engagement,

For peer review only - http://bmjopen.bmj.com/site/about/guidelines.xhtml

rather than follow-up logistics. Retention differed by treatment allocation (50% usual exercise care vs 75% EPIC-Neck) and none of the participants who did not start treatment or were discharged for non-attendance returned follow-up measures. In contrast, treatment completers across both groups had 91% retention (22/24). Additionally, treatment completion was higher in EPIC-Neck (75% vs 50% usual exercise care). Taken together, these patterns indicate engagement-related attrition and possible allocation-preference effects within this unblinded feasibility trial, reinforcing the need to consider participant blinding in the definitive exercise-versus-exercise trial to reduce allocation-related differences.[79, 80]

Intervention coherence and delivery are suboptimal

Although overall acceptability was broadly positive, there were clear concerns within the intervention coherence domain, particularly among participants. Many had limited understanding of the programme rationale and performance objectives. These coherence deficits align with lower delivery fidelity scores for the corresponding components (mean scores <3), suggesting that limited patient understanding may have been related, at least in part, to suboptimal physiotherapist delivery. Physiotherapists also reported reduced confidence and clarity when delivering newer or more complex EPIC-Neck components.

Addressing these issues is crucial before progressing to a definitive trial. Poor fidelity can reduce treatment effects,[81] risk misinterpreting null findings [82, 83] and threaten internal validity.[84] However, delivery fidelity should not be interpreted as a simple success/failure judgement. Although only 14% of participants received EPIC-Neck at an acceptable overall fidelity level, 35% of components were delivered acceptably,

For peer review only

For peer review only - http://bmjopen.bmj.com/site/about/guidelines.xhtml

meaning some intended intervention mechanisms may still have been activated. Evidence from complex behavioural interventions also indicates that effectiveness may still be possible despite variable or moderate delivery fidelity, depending on which components are most critical to the intervention mechanism.[46, 85, 86] Nevertheless, fidelity concerns remain substantial because lower-fidelity EPIC-Neck components included those intended to support long-term self-management, such as flare-up management and general exercise, which may plausibly influence long-term clinical outcomes in a definitive trial. These concerns are modifiable. Consistent with Medical Research Council guidance, EPIC-Neck and its training package can be refined and re-tested, with a focus on strengthening delivery fidelity and intervention coherence before progressing to a definitive evaluation.[22]

EPIC-Neck was acceptable with concerns about intervention coherence and was distinct from usual exercise care

Despite concerns about intervention coherence preventing immediate progression to a definitive trial, EPIC-Neck was broadly acceptable across the remaining domains to both patients and physiotherapists. Additionally, the operationalisation of EPIC-Neck components (e.g., behaviour change strategies, performance objectives) appears different from usual exercise care.[87] This reduces the risk of contamination in a definitive trial diluting any between-group effect.[88-90] Overall, EPIC-Neck appears acceptable and sufficiently distinct from usual exercise care, but requires refinement and further fidelity testing before definitive evaluation.

For peer review only - http://bmjopen.bmj.com/site/about/guidelines.xhtml

Future refinement before a definitive trial

Before progression to a definitive trial, key refinements are needed to optimise retention, delivery fidelity and intervention coherence. Retention strategies should address both questionnaire return and treatment engagement, as missing follow-up data appeared linked to treatment non-initiation and non-attendance. Future procedures could consider reducing questionnaire burden (e.g., electronic completion or shorter questionnaires), increasing the financial incentive for completion, and incorporating blinding procedures where feasible.[43, 78-80] Additional strategies to optimise delivery and intervention coherence should be designed collaboratively with patients and physiotherapists, and informed by theory and existing evidence. Potential refinements include strengthening physiotherapist training through longer training duration, additional practice, and regular monitoring and feedback.[91-95] Intervention coherence may improve through better delivery, but additional strategies could include methods to check patient understanding during treatment, such as teach-back approaches.[96]

Strengths and limitations

A key strength of this trial is the integration of quantitative and qualitative data, incorporating perspectives from both patients and physiotherapists, enabling a more comprehensive assessment of feasibility. A further strength is that 94% of appointments were audio-recorded, providing a robust assessment of delivery fidelity and contamination.[97] Finally, efforts to minimise exclusion based on English language and age resulted in a diverse cohort (40% Asian; age range 19–82), enhancing external validity by better reflecting populations attending NHS musculoskeletal services.[28]

For peer review only - http://bmjopen.bmj.com/site/about/guidelines.xhtml

555 The target sample size was not reached, which limits the precision of feasibility
556 estimates and means progression criteria and subgroup patterns should be
557 interpreted cautiously. The 2:1 allocation ratio increased intervention exposure and
558 feedback but resulted in fewer usual exercise care participants, reducing the
559 precision of usual-care estimates and limiting interpretation of between-group
560 descriptive patterns. Participants and outcome assessors were not blinded, which
561 may have introduced performance or detection bias, particularly for self-reported
562 outcomes; blinding of outcome assessment will therefore be incorporated into the
563 definitive trial where feasible. Physiotherapists delivered EPIC-Neck to relatively few
564 patients; therefore, some fidelity issues may be overestimated and could improve
565 with increased exposure and practice.[98, 99] Fidelity assessments were undertaken
566 unblinded, which may have influenced scoring despite use of a structured
567 competence checklist. In addition, interviews were conducted by the intervention
568 developer, whose role in EPIC-Neck development may have introduced social
569 desirability and allegiance bias. The use of topic guides, reflective discussions and
570 team-based interpretation helped mitigate this risk, but independent interviewing and
571 analysis would strengthen future evaluations.

572 CONCLUSION

573 The EPIC-Neck programme shows potential for evaluation in a definitive trial, but
574 progression is conditional on further refinement. Recruitment and contamination
575 findings were encouraging, and the intervention was broadly acceptable and distinct
576 from usual exercise care. However, retention, delivery fidelity and intervention
577 coherence require optimisation before definitive evaluation. Future work should
578 refine the EPIC-Neck programme, training package and trial retention procedures,

For peer review only - http://bmjopen.bmj.com/site/about/guidelines.xhtml

followed by targeted repeat testing of delivery fidelity, participant and physiotherapist understanding of the intervention and retention. Depending on the extent of these refinements, this may require a further feasibility or pilot study before progressing to a full-scale definitive trial.

For peer review only

For peer review only - http://bmjopen.bmj.com/site/about/guidelines.xhtml

LIST OF ABBREVIATIONS

AQoL. Assessment of Quality-of-Life

CI. Confidence Interval

DNA. Did Not Attend

EPIC. Exercise Prescription Improved through Co-design

HADS. Hospital Anxiety and Depression Scale

IMD. Index of Multiple Deprivation

NDI. Neck disability index

NHS. National Health Service

NIH-BCC. National Institutes of Health-Behaviour Change Consortium

PIL. Participant Information Leaflet

PO. Performance Objectives

PPIE. Patient and Public Involvement and Engagement

PSEQ. Pain self-efficacy questionnaire

PSFS. Patient Specific Functional Scale

SCI. Sleep condition indicator

SD. Standard Deviation

SOPA. Survey of Pain Attitudes Questionnaire

For peer review only

TFA. Theoretical Framework of Acceptability

TSK. Tampa Scale of Kinesiophobia

UEC. Usual Exercise Care

VAS. Visual analogue scale

DECLARATIONS

Ethics approval and consent to participate

The study was registered with ISRCTN81746901 and received ethical approval from South Central - Oxford B Research Ethics Committee (24/SC/0006). Patient participants and physiotherapists gave informed consent to participate in the study before taking part.

Participant consent for publication

Not applicable.

Availability of data and materials

Data are available upon reasonable request. Data sharing requests can be considered via contact with the corresponding author.

Competing interests

The authors declare that they have no competing interests

For peer review only

Funding

Jonathan Price, Clinical Doctoral Research Fellow, NIHR302141 is funded by HEE/NIHR for this research project. The views expressed in this publication are those of the author(s) and not necessarily those of the NIHR, NHS or the UK Department of Health and Social Care.

The funders are not involved in trial design, data collection, management, analysis, writing of the report or the decision to submit this report for publication.

The study sponsor is Birmingham Community Healthcare NHS Foundation Trust (BCHC.researchinnovation@nhs.net).

For peer review only

Author Contributions

JP was the chief investigator and conceived and designed the study, was awarded the funding and drafted the manuscript. AR, NI, CG, PP contributed to the study design, provided specific content and edited the trial protocol and manuscript. MB contributed to data analysis. All authors have reviewed and approved the manuscript.

JP is the guarantor and accepts full responsibility for the overall content, the conduct of the study, access to the data and the decision to submit for publication.

Collaborators

EPIC-Neck feasibility trial collaborators: Apurba Subedi (Birmingham Community Healthcare NHS Foundation Trust), Hayley Alderton (Berkshire Healthcare NHS Foundation Trust), Katherine Yeung (Birmingham Community Healthcare NHS Foundation Trust), Longsun Yau (Birmingham Community Healthcare NHS

For peer review only - http://bmjopen.bmj.com/site/about/guidelines.xhtml

Foundation Trust), Paul Matthews (Nottingham University Hospitals NHS Trust), Rajvinder Shokkar (Berkshire Healthcare NHS Foundation Trust)

Acknowledgements

We thank the EPIC-Neck patient and public involvement and engagement group for their contributions.

Author details

[1]Musculoskeletal Physiotherapy Services, Birmingham Community Healthcare NHS Foundation Trust, Birmingham, United Kingdom

[2]Research and Innovation, Birmingham Community Healthcare NHS Foundation Trust, Birmingham, United Kingdom

[3]School of Sport, Exercise and Rehabilitation Sciences, College of Life and Environmental Sciences, University of Birmingham, Birmingham, United Kingdom

[4]School of Physical Therapy, Western University, Ontario, Canada

[5]Institute of Applied Health Research, College of Medical and Dental Sciences, University of Birmingham, Birmingham, United Kingdom

*Corresponding author

Email: Jonathan.price2@nhs.net

ORCID iDs

Jonathan Price: https://orcid.org/0000-0002-5505-1564

For peer review only

Alison Rushton: https://orcid.org/0000-0001-8114-7669

Natalie Ives: https://orcid.org/0000-0002-1664-7541

Marilyn Bradbury: https://orcid.org/0000-0001-6000-7229

Priti Parmar: https://orcid.org/0000-0003-4344-144X

Colin J Greaves: https://orcid.org/0000-0003-4425-2691

FIGURES

Figure 1 – CONSORT flow diagram. Abbreviations: CNSNP – Chronic non-specific neck pain; DNA – Did not attend; PIL – Participant information leaflet

SUPPLEMENTARY FILES

Supplementary file 1 – CONSORT for pilot and feasibility trials

Supplementary file 2 – Feasibility trial protocol ethics

Supplementary file 3 – Supporting information

Supplementary file 4 – GRIPP2-SF

Supplementary file 5 – Acceptability data

For peer review only

REFERENCES

1. Xu F, Zhang X, Yang M, et al. Magnitude, temporal trend and inequality in burden of neck pain: an analysis of the Global Burden of Disease Study 2019. *BMC Musculoskelet Disord* 2025;26(1):202. doi: 10.1186/s12891-025-08342-3

2. Myhrvold BL, Irgens P, Robinson HS, et al. Visual trajectory pattern as prognostic factors for neck pain. *Eur J Pain* 2020;24(9):1752-64. doi: 10.1002/ejp.1622

3. Horn ME, Fritz JM. Timing of physical therapy consultation on 1-year healthcare utilization and costs in patients seeking care for neck pain: a retrospective cohort. *BMC Health Serv Res* 2018;18(1):887. doi: 10.1186/s12913-018-3699-0

4. Hey HWD, Lim JXY, Ong JZ, et al. Epidemiology of Neck Pain and Its Impact on Quality-of-Life-A Population-Based, Cross Sectional Study in Singapore. *Spine (Phila Pa 1976)* 2021;46(22):1572-80. doi: 10.1097/BRS.0000000000004071

5. Irgens P, Myhrvold BL, Kongsted A, et al. The clinical course of neck pain: Are trajectory patterns stable over a 1-year period? *Eur J Pain* 2022;26(2):531-42. doi: 10.1002/ejp.1879

6. Hallman DM, Holtermann A, Dencker-Larsen S, et al. Are trajectories of neck-shoulder pain associated with sick leave and work ability in workers? A 1-year prospective study. *BMJ Open* 2019;9(3):e022006. doi: 10.1136/bmjopen-2018-022006

7. National Institute for Health and Care Excellence. Chronic pain (primary and secondary) in over 16s: assessment of all chronic pain and management of chronic primary pain [Internet]. London: NICE; 2021 [Available from: https://www.nice.org.uk/guidance/ng193.

8. Bier JD, Scholten-Peeters WGM, Staal JB, et al. Clinical Practice Guideline for Physical Therapy Assessment and Treatment in Patients With Nonspecific Neck Pain. *Phys Ther* 2018;98(3):162-71. doi: 10.1093/ptj/pzx118

9. Blanpied PR, Gross AR, Elliott JM, et al. Neck Pain: Revision 2017. *J Orthop Sports Phys Ther* 2017;47(7):A1-A83. doi: 10.2519/jospt.2017.0302

10. Wilhelm MP, Donaldson M, Griswold D, et al. The Effects of Exercise Dosage on Neck-Related Pain and Disability: A Systematic Review With Meta-analysis. *J Orthop Sports Phys Ther* 2020;50(11):607-21. doi: 10.2519/jospt.2020.9155

11. de Zoete RMJ, Brown L, Oliveira K, et al. The effectiveness of general physical exercise for individuals with chronic neck pain: a systematic review of randomised controlled trials. *Eur J Physiother* 2020;22(3):141-47. doi: 10.1080/21679169.2018.1561942

12. Garzonio S, Arbasetti C, Geri T, et al. Effectiveness of Specific Exercise for Deep Cervical Muscles in Nonspecific Neck Pain: A Systematic Review and Meta-Analysis. *Phys Ther* 2022;102(5):pzac001. doi: 10.1093/ptj/pzac001

13. Martin-Gomez C, Sestelo-Diaz R, Carrillo-Sanjuan V, et al. Motor control using cranio-cervical flexion exercises versus other treatments for non-specific chronic neck pain: A systematic review and meta-analysis. *Musculoskelet Sci Pract* 2019;42:52-59. doi: 10.1016/j.msksp.2019.04.010

For peer review only - http://bmjopen.bmj.com/site/about/guidelines.xhtml

14. Li Y, Li S, Jiang J, et al. Effects of yoga on patients with chronic nonspecific neck pain: A PRISMA systematic review and meta-analysis. *Medicine (Baltimore)* 2019;98(8):e14649. doi: 10.1097/MD.0000000000014649

15. Martini JD, Ferreira GE, Xavier de Araujo F. Pilates for neck pain: A systematic review and meta-analysis of randomised controlled trials. *J Bodyw Mov Ther* 2022;31:37-44. doi: 10.1016/j.jbmt.2022.03.011

16. Price J, Rushton A, Tyros V, et al. Expert consensus on the important chronic non-specific neck pain motor control and segmental exercise and dosage variables: An international e-Delphi study. *PLoS One* 2021;16(7):e0253523. doi: 10.1371/journal.pone.0253523

17. McLean SM, Klaber Moffett JA, Sharp DM, et al. A randomised controlled trial comparing graded exercise treatment and usual physiotherapy for patients with non-specific neck pain (the GET UP neck pain trial). *Man Ther* 2013;18(3):199-205. doi: 10.1016/j.math.2012.09.005

18. Villanueva-Ruiz I, Falla D, Saez M, et al. Manual therapy and neck-specific exercise are equally effective for treating non-specific neck pain but only when exercise adherence is maximised: A randomised controlled trial. *Musculoskelet Sci Pract* 2025;77:103319. doi: 10.1016/j.msksp.2025.103319

19. Dalager T, Welch A, O'Leary SP, et al. Clinically Relevant Decreases in Neck/Shoulder Pain Among Office Workers Are Associated With Strength Training Adherence and Exercise Compliance: Explorative Analyses From a Randomized Controlled Trial. *Phys Ther* 2023;103(2):pzac166. doi: 10.1093/ptj/pzac166

20. Zebis MK, Andersen CH, Sundstrup E, et al. Time-wise change in neck pain in response to rehabilitation with specific resistance training: implications for exercise prescription. *PLoS One* 2014;9(4):e93867. doi: 10.1371/journal.pone.0093867

21. Price J, Rushton A, Ives N, et al. Co-development of an evidence-informed, theoretically driven exercise programme for people with chronic non-specific neck pain (the EPIC-Neck programme - "Exercise Prescription Improved through Co-design"). *BMC Musculoskelet Disord* 2025;26(1):689. doi: 10.1186/s12891-025-08918-z

22. Skivington K, Matthews L, Simpson SA, et al. A new framework for developing and evaluating complex interventions: update of Medical Research Council guidance. *BMJ* 2021;374:n2061. doi: 10.1136/bmj.n2061

23. Eldridge SM, Chan CL, Campbell MJ, et al. CONSORT 2010 statement: extension to randomised pilot and feasibility trials. *BMJ* 2016;355:i5239. doi: 10.1136/bmj.i5239

24. Price J, Rushton A, Ives N, et al. Co-designed neck exercise (EPIC-Neck) vs usual exercise care for people with chronic non-specific neck pain: protocol for a randomised feasibility study with process evaluation. *Pilot Feasibility Stud* 2025;11(1):26. doi: 10.1186/s40814-025-01608-6

25. National Institute for Health and Care Excellence. Neck pain - non-specific: Clinical Knowledge Summaries 2023 [Available from: https://cks.nice.org.uk/topics/neck-pain-non-specific/.

26. Tsakitzidis G, Remmen R, Dankaerts W, et al. Non-specific neck pain and evidence-based practice. *European scientific journal* 2013;9(3):1-19.

For peer review only - http://bmjopen.bmj.com/site/about/guidelines.xhtml

27. NHS England. Increasing diversity in research participation: A good practice guide for engaging with underrepresented groups [Internet]. London: NHS England; 2023 [Available from: https://www.england.nhs.uk/aac/publication/increasing-diversity-in-research-participation/.

28. National Institute for Health and Care Research. Improving inclusion of under-served groups in clinical research: Guidance from INCLUDE Project [Internet]. London: NIHR; 2022 [Available from: https://www.nihr.ac.uk/documents/improving-inclusion-of-under-served-groups-in-clinical-research-guidance-from-include-project/25435.

29. Thabane L, Lancaster G. A guide to the reporting of protocols of pilot and feasibility trials. *Pilot Feasibility Stud* 2019;5(1):37. doi: 10.1186/s40814-019-0423-8

30. Armijo-Olivo S, Fuentes J, Da Costa BR, et al. Blinding in Physical Therapy Trials and Its Association with Treatment Effects. *American Journal of Physical Medicine & Rehabilitation* 2017;96(1):34-44. doi: 10.1097/phm.0000000000000521

31. Miller WR, Rollnick S. Motivational Interviewing, Second Edition: Preparing People for Change. 2nd ed. New York, NY: Guilford Publications 2002.

32. Elwyn G, Durand MA, Song J, et al. A three-talk model for shared decision making: multistage consultation process. *BMJ* 2017;359:j4891. doi: 10.1136/bmj.j4891

33. To D, De Carvalho D, Pike A, et al. A qualitative study exploring perceived barriers and enablers to fidelity of training and delivery for an intervention to reduce non-indicated imaging for low back pain. *Chiropr Man Therap* 2023;31(1):6. doi: 10.1186/s12998-023-00480-6

34. Beneciuk JM, George SZ, Greco CM, et al. Targeted interventions to prevent transitioning from acute to chronic low back pain in high-risk patients: development and delivery of a pragmatic training course of psychologically informed physical therapy for the TARGET trial. *Trials* 2019;20(1):256. doi: 10.1186/s13063-019-3350-3

35. Craven K, Holmes J, Powers K, et al. Embedding mentoring to support trial processes and implementation fidelity in a randomised controlled trial of vocational rehabilitation for stroke survivors. *BMC Med Res Methodol* 2021;21(1):203. doi: 10.1186/s12874-021-01382-y

36. Mellor K, Albury C, Dutton SJ, et al. Recommendations for progression criteria during external randomised pilot trial design, conduct, analysis and reporting. *Pilot Feasibility Stud* 2023;9(1):59. doi: 10.1186/s40814-023-01291-5

37. Price J, Rushton A, Tyros I, et al. Effectiveness and optimal dosage of exercise training for chronic non-specific neck pain: A systematic review with a narrative synthesis. *PLoS One* 2020;15(6):e0234511. doi: 10.1371/journal.pone.0234511

38. Littlewood C, Bateman M, Brown K, et al. A self-managed single exercise programme versus usual physiotherapy treatment for rotator cuff tendinopathy: a randomised controlled trial (the SELF study). *Clin Rehabil* 2016;30(7):686-96. doi: 10.1177/0269215515593784

39. Lamb SE, Williamson EM, Heine PJ, et al. Exercises to improve function of the rheumatoid hand (SARAH): a randomised controlled trial. *Lancet* 2015;385(9966):421-9. doi: 10.1016/S0140-6736(14)60998-3

For peer review only - http://bmjopen.bmj.com/site/about/guidelines.xhtml

799 40. Hu S, Guinan E, Mockler D, et al. Retention rates and reasons for non-retention in
800 exercise oncology trials in the post-treatment phase-a systematic review. *J Cancer*
801 *Surviv* 2025;19(5):1535-43. doi: 10.1007/s11764-024-01569-4

802 41. Watson S, Wong C, Smith T. Factors associated with trial recruitment and retention of
803 people with osteoarthritis: analysis of 215 randomised controlled trials from 2013-
804 2021. *Physiother Rev* 2022;26(2):5-19. doi: 10.5114/phr.2022.117578

805 42. Jacques RM, Ahmed R, Harper J, et al. Recruitment, consent and retention of
806 participants in randomised controlled trials: a review of trials published in the National
807 Institute for Health Research (NIHR) Journals Library (1997-2020). *BMJ Open*
808 2022;12(2):e059230. doi: 10.1136/bmjopen-2021-059230

809 43. Elfeky A, Gillies K, Gardner H, et al. Non-randomised evaluations of strategies to
810 increase participant retention in randomised controlled trials: a systematic review.
811 *Syst Rev* 2020;9(1):224. doi: 10.1186/s13643-020-01471-x

812 44. Higgins JPT, Green S. Cochrane Handbook for Systematic Reviews of Interventions.
813 Chichester, UK: The Cochrane Collaboration 2011.

814 45. Thompson DP, Oldham JA, Woby SR. Does adding cognitive-behavioural physiotherapy
815 to exercise improve outcome in patients with chronic neck pain? A randomised
816 controlled trial. *Physiotherapy* 2016;102(2):170-7. doi: 10.1016/j.physio.2015.04.008

817 46. Frost J, Wingham J, Britten N, et al. Home-based rehabilitation for heart failure with
818 reduced ejection fraction: mixed methods process evaluation of the REACH-HF
819 multicentre randomised controlled trial. *BMJ Open* 2019;9(8):e026039. doi:
820 10.1136/bmjopen-2018-026039

821 47. Thompson TP, Lambert JD, Greaves CJ, et al. Intervention delivery fidelity assessment
822 of a counseling-based intervention for promoting smoking reduction and increasing
823 physical activity. *Health Psychol* 2018;37(7):627-37. doi: 10.1037/hea0000613

824 48. Dreyfus SE. The Five-Stage Model of Adult Skill Acquisition. *Bull Sci Technol Soc*
825 2004;24(3):177-81. doi: 10.1177/0270467604264992

826 49. Cross R, Greaves CJ, Withall J, et al. Delivery fidelity of the REACT (REtirement in
827 ACTion) physical activity and behaviour maintenance intervention for community
828 dwelling older people with mobility limitations. *BMC Public Health* 2022;22(1):1112.
829 doi: 10.1186/s12889-022-13496-z

830 50. Withers TM, Greaves CJ, Bown MJ, et al. A feasibility study of the CRISP intervention; a
831 cardiovascular risk reduction intervention in patients with an abdominal aortic
832 aneurysm. *NIHR Open Res* 2024;4:34. doi: 10.3310/nihropenres.13596.2

833 51. Luker JA, Craig LE, Bennett L, et al. Implementing a complex rehabilitation intervention
834 in a stroke trial: a qualitative process evaluation of AVERT. *BMC Med Res Methodol*
835 2016;16:52. doi: 10.1186/s12874-016-0156-9

836 52. Robinson K, Allen F, Darby J, et al. Contamination in complex healthcare trials: the falls
837 in care homes (FinCH) study experience. *BMC Med Res Methodol* 2020;20(1):46.
838 doi: 10.1186/s12874-020-00925-z

For peer review only - http://bmjopen.bmj.com/site/about/guidelines.xhtml

53. Sekhon M, Cartwright M, Francis JJ. Development of a theory-informed questionnaire to assess the acceptability of healthcare interventions. *BMC Health Serv Res* 2022;22(1):279. doi: 10.1186/s12913-022-07577-3

54. Sekhon M, Cartwright M, Francis JJ. Acceptability of healthcare interventions: an overview of reviews and development of a theoretical framework. *BMC Health Serv Res* 2017;17(1):88. doi: 10.1186/s12913-017-2031-8

55. Vernon H, Mior S. The Neck Disability Index: a study of reliability and validity. *J Manipulative Physiol Ther* 1991;14(7):409-15.

56. Bobos P, MacDermid JC, Walton DM, et al. Patient-Reported Outcome Measures Used for Neck Disorders: An Overview of Systematic Reviews. *J Orthop Sports Phys Ther* 2018;48(10):775-88. doi: 10.2519/jospt.2018.8131

57. Modarresi S, Lukacs MJ, Ghodrati M, et al. A Systematic Review and Synthesis of Psychometric Properties of the Numeric Pain Rating Scale and the Visual Analog Scale for Use in People With Neck Pain. *Clin J Pain* 2021;38(2):132-48. doi: 10.1097/AJP.0000000000000999

58. Feng YS, Kohlmann T, Janssen MF, et al. Psychometric properties of the EQ-5D-5L: a systematic review of the literature. *Qual Life Res* 2021;30(3):647-73. doi: 10.1007/s11136-020-02688-y

59. Zigmond AS, Snaith RP. The hospital anxiety and depression scale. *Acta Psychiatr Scand* 1983;67(6):361-70. doi: 10.1111/j.1600-0447.1983.tb09716.x

60. Espie CA, Kyle SD, Hames P, et al. The Sleep Condition Indicator: a clinical screening tool to evaluate insomnia disorder. *BMJ Open* 2014;4(3):e004183. doi: 10.1136/bmjopen-2013-004183

61. Stratford P, Gill C, Westaway M, et al. Assessing disability and change on individual patients: a report of a patient specific measure. *Physiother Can* 1995;47(4):258-63.

62. Maxwell A, Ozmen M, Iezzi A, et al. Deriving population norms for the AQoL-6D and AQoL-8D multi-attribute utility instruments from web-based data. *Qual Life Res* 2016;25(12):3209-19. doi: 10.1007/s11136-016-1337-z

63. Richardson J, Iezzi A, Khan MA, et al. Validity and reliability of the Assessment of Quality of Life (AQoL)-8D multi-attribute utility instrument. *Patient* 2014;7(1):85-96. doi: 10.1007/s40271-013-0036-x

64. Jensen MP, Keefe FJ, Lefebvre JC, et al. One- and two-item measures of pain beliefs and coping strategies. *Pain* 2003;104(3):453-69. doi: 10.1016/S0304-3959(03)00076-9

65. Dupuis F, Cherif A, Batcho C, et al. The Tampa Scale of Kinesiophobia: A Systematic Review of Its Psychometric Properties in People With Musculoskeletal Pain. *Clin J Pain* 2023;39(5):236-47. doi: 10.1097/AJP.0000000000001104

66. Nicholas MK. The pain self-efficacy questionnaire: Taking pain into account. *Eur J Pain* 2007;11(2):153-63. doi: 10.1016/j.ejpain.2005.12.008

For peer review only

For peer review only - http://bmjopen.bmj.com/site/about/guidelines.xhtml

878 67. Withers TM, Garner NJ, Thorley CS, et al. Intervention fidelity assessment: A sub-study
879 of the Norfolk Diabetes Prevention Study (NDPS). *Br J Health Psychol*
880 2023;28(3):740-52. doi: 10.1111/bjhp.12651

881 68. Thabane L, Ma J, Chu R, et al. A tutorial on pilot studies: the what, why and how. *BMC*
882 *Med Res Methodol* 2010;10(1):1. doi: 10.1186/1471-2288-10-1

883 69. Gale NK, Heath G, Cameron E, et al. Using the framework method for the analysis of
884 qualitative data in multi-disciplinary health research. *BMC Med Res Methodol*
885 2013;13:117. doi: 10.1186/1471-2288-13-117

886 70. Klingberg S, Stalmeijer RE, Varpio L. Using framework analysis methods for qualitative
887 research: AMEE Guide No. 164. *Med Teach* 2024;46(5):603-10. doi:
888 10.1080/0142159X.2023.2259073

889 71. Teare MD, Dimairo M, Shephard N, et al. Sample size requirements to estimate key
890 design parameters from external pilot randomised controlled trials: a simulation
891 study. *Trials* 2014;15(1):264. doi: 10.1186/1745-6215-15-264

892 72. Public Health England. Chronic pain in adults [Internet]. London: Public Health England;
893 2017 [Available from:
894 https://assets.publishing.service.gov.uk/media/5fc8c6b78fa8f547585ed7f3/Chronic_P
895 ain_Report.pdf.

896 73. Adebajo A, Shikoh S, Kumar K, et al. Ethnic minority musculoskeletal health.
897 *Rheumatology (Oxford)* 2018;57(2):201-03. doi: 10.1093/rheumatology/kex006

898 74. Versus Arthritis. Chronic pain in England: Unseen, unequal, unfair [Internet]. London:
899 Versus Arthritis; 2021 [Available from: https://www.versusarthritis.org/about-
900 arthritis/data-and-statistics/chronic-pain-in-england/.

901 75. Staniszewska S, Brett J, Simera I, et al. GRIPP2 reporting checklists: tools to improve
902 reporting of patient and public involvement in research. *Res Involv Engagem*
903 2017;3(1):13. doi: 10.1186/s40900-017-0062-2

904 76. Gillies K, Kearney A, Keenan C, et al. Strategies to improve retention in randomised
905 trials. *Cochrane Database Syst Rev* 2021(3):MR000032. doi:
906 10.1002/14651858.MR000032.pub3

907 77. Brueton V, Stenning SP, Stevenson F, et al. Best practice guidance for the use of
908 strategies to improve retention in randomized trials developed from two consensus
909 workshops. *J Clin Epidemiol* 2017;88:122-32. doi: 10.1016/j.jclinepi.2017.05.010

910 78. Elfeky A, Treweek S, Hannes K, et al. Using qualitative methods in pilot and feasibility
911 trials to inform recruitment and retention processes in full-scale randomised trials: a
912 qualitative evidence synthesis. *BMJ Open* 2022;12(4):e055521. doi:
913 10.1136/bmjopen-2021-055521

914 79. Hrobjartsson A, Emanuelsson F, Skou Thomsen AS, et al. Bias due to lack of patient
915 blinding in clinical trials. A systematic review of trials randomizing patients to blind
916 and nonblind sub-studies. *Int J Epidemiol* 2014;43(4):1272-83. doi:
917 10.1093/ije/dyu115

For peer review only - http://bmjopen.bmj.com/site/about/guidelines.xhtml

80. Armijo-Olivo S, Fuentes J, da Costa BR, et al. Blinding in Physical Therapy Trials and Its Association with Treatment Effects: A Meta-epidemiological Study. *Am J Phys Med Rehabil* 2017;96(1):34-44. doi: 10.1097/PHM.0000000000000521

81. Cook CE, Thigpen CA. Five good reasons to be disappointed with randomized trials. *J Man Manip Ther* 2019;27(2):63-65. doi: 10.1080/10669817.2019.1589697

82. Carroll C, Patterson M, Wood S, et al. A conceptual framework for implementation fidelity. *Implement Sci* 2007;2:40. doi: 10.1186/1748-5908-2-40

83. Toomey E, Hardeman W, Hankonen N, et al. Focusing on fidelity: narrative review and recommendations for improving intervention fidelity within trials of health behaviour change interventions. *Health Psychol Behav Med* 2020;8(1):132-51. doi: 10.1080/21642850.2020.1738935

84. Borrelli B. The Assessment, Monitoring, and Enhancement of Treatment Fidelity In Public Health Clinical Trials. *J Public Health Dent* 2011;71(Suppl 1):S52-63. doi: 10.1111/j.1752-7325.2011.00233.x

85. Dalal HM, Taylor RS, Jolly K, et al. The effects and costs of home-based rehabilitation for heart failure with reduced ejection fraction: The REACH-HF multicentre randomized controlled trial. *Eur J Prev Cardiol* 2019;26(3):262-72. doi: 10.1177/2047487318806358

86. Stathi A, Withall J, Greaves CJ, et al. A group-based exercise and behavioural maintenance intervention for adults over 65 years with mobility limitations: the REACT RCT. *Public Health Research* 2022;10(14):1-172. doi: 10.3310/mqbw6832

87. Kunstler BE, Cook JL, Freene N, et al. Physiotherapists use a small number of behaviour change techniques when promoting physical activity: A systematic review comparing experimental and observational studies. *J Sci Med Sport* 2018;21(6):609-15. doi: 10.1016/j.jsams.2017.10.027

88. Magill N, Knight R, McCrone P, et al. A scoping review of the problems and solutions associated with contamination in trials of complex interventions in mental health. *BMC Med Res Methodol* 2019;19(1):4. doi: 10.1186/s12874-018-0646-z

89. Gross A, Kay TM, Paquin JP, et al. Exercises for mechanical neck disorders. *Cochrane Database Syst Rev* 2015(1):CD004250. doi: 10.1002/14651858.CD004250.pub5

90. Hemming K, Taljaard M, Moerbeek M, et al. Contamination: How much can an individually randomized trial tolerate? *Stat Med* 2021;40(14):3329-51. doi: 10.1002/sim.8958

91. Simpson P, Holopainen R, Schutze R, et al. Becoming confidently competent: a qualitative investigation of training in cognitive functional therapy for persistent low back pain. *Physiother Theory Pract* 2024;40(4):804-16. doi: 10.1080/09593985.2022.2151333

92. Lawford BJ, Delany C, Bennell KL, et al. Training Physical Therapists in Person-Centered Practice for People With Osteoarthritis: A Qualitative Case Study. *Arthritis Care Res (Hoboken)* 2018;70(4):558-70. doi: 10.1002/acr.23314

93. Simpson P, Holopainen R, Schutze R, et al. Training of Physical Therapists to Deliver Individualized Biopsychosocial Interventions to Treat Musculoskeletal Pain

For peer review only

For peer review only - http://bmjopen.bmj.com/site/about/guidelines.xhtml

Conditions: A Scoping Review. *Phys Ther* 2021;101(10):pzab188. doi: 10.1093/ptj/pzab188

94. Hatfield TG, Withers TM, Greaves CJ. Systematic review of the effect of training interventions on the skills of health professionals in promoting health behaviour, with meta-analysis of subsequent effects on patient health behaviours. *BMC Health Serv Res* 2020;20(1):593. doi: 10.1186/s12913-020-05420-1

95. Newton C, Singh G, Nolan D, et al. Cognitive Functional Therapy compared with usual physiotherapy care in people with persistent low back pain: a mixed methods feasibility randomised controlled trial in the United Kingdom National Health Service. *Physiotherapy* 2024;123:118-32. doi: 10.1016/j.physio.2024.02.003

96. Shersher V, Haines TP, Sturgiss L, et al. Definitions and use of the teach-back method in healthcare consultations with patients: A systematic review and thematic synthesis. *Patient Educ Couns* 2021;104(1):118-29. doi: 10.1016/j.pec.2020.07.026

97. Toomey E, Matthews J, Guerin S, et al. Development of a Feasible Implementation Fidelity Protocol Within a Complex Physical Therapy-Led Self-Management Intervention. *Phys Ther* 2016;96(8):1287-98. doi: 10.2522/ptj.20150446

98. Rathod T, Belcher J, Montgomery AA, et al. Health services changes: is a run-in period necessary before evaluation in randomised clinical trials? *Trials* 2014;15(1):41. doi: 10.1186/1745-6215-15-41

99. Khan N, Abboudi H, Khan MS, et al. Measuring the surgical 'learning curve': methods, variables and competency. *BJU Int* 2014;113(3):504-8. doi: 10.1111/bju.12197

For peer review only

For peer review only - http://bmjopen.bmj.com/site/about/guidelines.xhtml

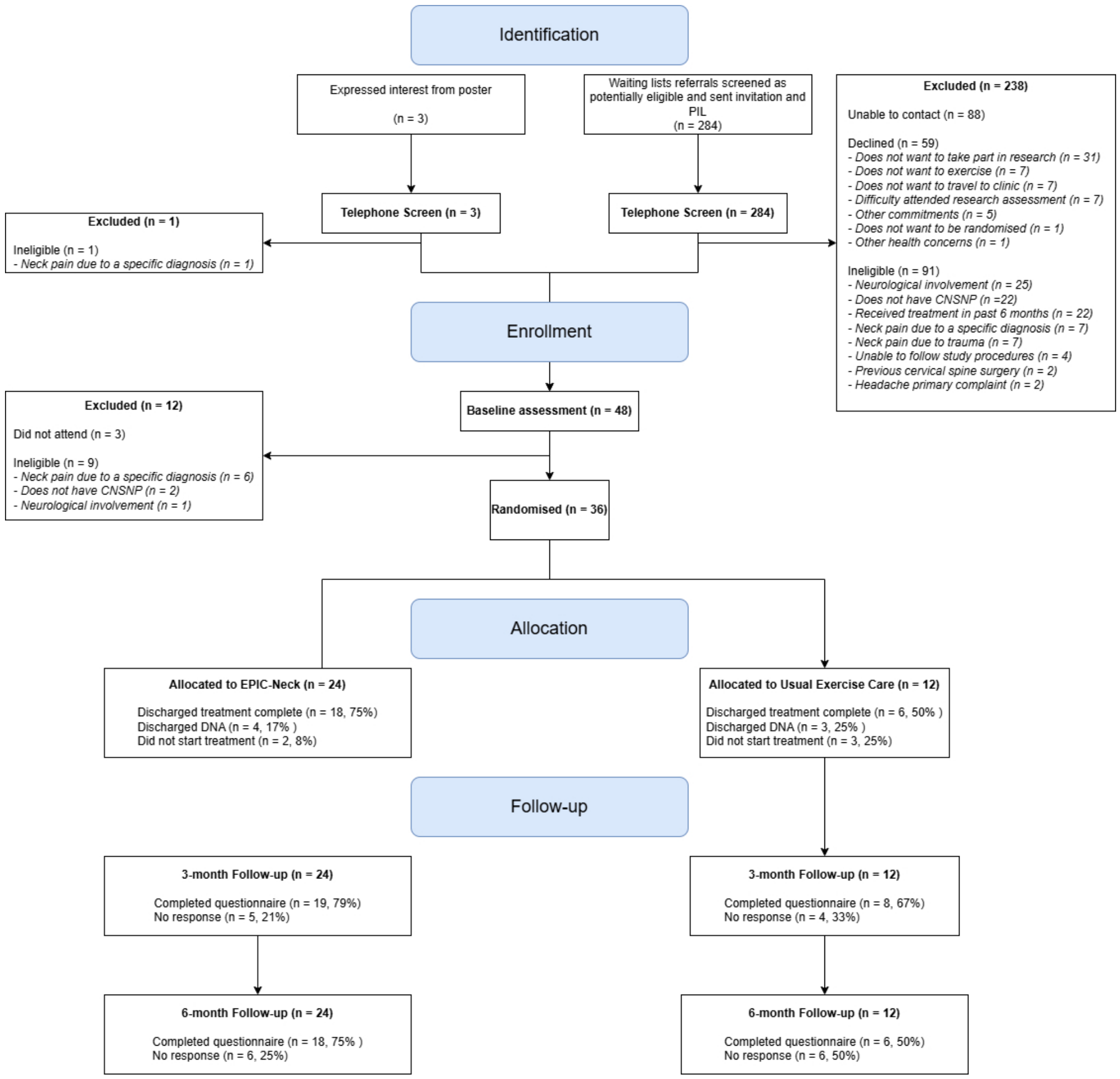

396x381mm (72 x 72 DPI)
