# Supplementary material for: Co-developed neck exercise (EPIC-Neck) versus usual exercise care for people with chronic non-specific neck pain: a UK multicentre randomised feasibility trial
Source: BMJ Open. 2026 Oct 20;16(10):e120571. doi: 10.1136/bmjopen-2026-120571 (PMC13711528; doi:10.1136/bmjopen-2026-120571)
Supplement: Supplementary file 7 [file bmjopen-2026-120571.reviewer_comments.pdf]

PEER REVIEW HISTORY

BMJ Open publishes all reviews undertaken for accepted manuscripts. Reviewers are asked to complete a checklist review form and are provided with free text boxes to elaborate on their assessment. These free text comments are reproduced below.

ARTICLE DETAILS

Title (Provisional)

Co-developed neck exercise (EPIC-Neck) versus usual exercise care for people with chronic non-specific neck pain: a UK multicentre randomised feasibility trial

Authors

Price, Jonathan; Rushton, Alison; Ives, Natalie; Bradbury, Marilyn; Parmar, Priti; Greaves, Colin J

VERSION 1 - REVIEW

**Reviewer**	**1**
**Name**	**Bagherian, Sajad**
**Affiliation**	**Shahrekord University**
**Date**	**20-May-2026**
**COI**	**None**

The study addresses an important question and uses an appropriate feasibility design. That said, the overall message of the paper appears somewhat more positive than the data seem to support. While recruitment was successful and contamination appears low, retention at 6 months was suboptimal and intervention fidelity was not yet strong. These findings suggest promise, but also indicate that meaningful refinement is still needed before progression to a definitive trial can be fully justified.

Abstract

The abstract is clear and well-structured overall, but the conclusion should be phrased more cautiously. In particular, the statement that progression to a definitive trial is feasible may overstate the findings, given that retention was low and only a small proportion of participants received the intervention at an acceptable fidelity level. The abstract would benefit from a more balanced summary of the feasibility signals, making it clearer that progression appears possible only with important modifications. The acceptability findings are also somewhat mixed, as concerns about intervention coherence were identified, and this nuance should be better reflected in the concluding statements.

BACKGROUND

The topic is well chosen and the rationale for evaluating a co-developed intervention for chronic non-specific neck pain is understandable. However, the introduction would benefit from a sharper articulation of the specific research gap and a clearer explanation of why this intervention is sufficiently distinct from existing exercise-based care approaches to warrant feasibility testing in its current form. If novelty is emphasized, this should be framed carefully and supported precisely. It would also help to state the feasibility objectives more explicitly and avoid any implication that the study is intended to demonstrate effectiveness.

Methods

The use of a multicentre randomised feasibility design with embedded process evaluation is appropriate and a clear strength of the study. The progression criteria are also useful. However, the manuscript should ensure that these criteria are fully justified and consistently interpreted throughout. In particular, more clarity may be needed around how acceptable fidelity was defined and why the selected thresholds were chosen. The implications of the 2:1 allocation ratio should also be briefly justified. Given that participants and outcome assessors were not blinded, the discussion of potential bias should be sufficiently transparent. In addition, the methods should make clear how therapist training, support, and monitoring were handled, especially since fidelity emerged as an important issue.

Results

The results are informative, but the interpretation should remain closely aligned with the actual feasibility findings. Recruitment appears to have been successful, which is encouraging. However, retention at 6 months was below an ideal level for a future definitive trial, and this should be presented as a significant issue rather than a minor limitation. Similarly, the fidelity findings appear central to the feasibility question and should be given greater emphasis. The fact that only a small proportion of participants received the intervention at an acceptable level suggests that the intervention may not yet be sufficiently deliverable in routine practice in its current form. The findings on acceptability are useful, but they should be reported with more nuance, as positive responses seem to coexist with concerns about intervention coherence.

Discussion

The discussion is likely to benefit from a more cautious tone overall. The manuscript should avoid giving the impression that the feasibility trial was broadly successful when, in fact, the results appear mixed across key progression domains (green for recruitment/contamination, amber for fidelity/acceptability, red for retention). Recruitment and contamination findings are encouraging, but retention and fidelity are major feasibility concerns and should be discussed as such.

The qualitative findings seem especially important here, as they may help explain why fidelity and coherence were not stronger. It would strengthen the paper if the discussion more clearly outlined specific, actionable modifications to the intervention, training package,

and trial procedures that will be tested before any future definitive trial is undertaken. A vague statement that "refinement is needed" is insufficient.

Additionally, the discussion should more transparently address the risk of allegiance bias and the implications of the unmet sample size.

Conclusion

The conclusion should be moderated. At present, the data seem to support conditional feasibility rather than clear readiness for a definitive trial. It would be more accurate to conclude that the intervention shows potential, but requires further refinement, improved delivery fidelity, and better participant retention before definitive evaluation. A slightly more cautious and proportionate conclusion would better reflect the study findings. In fact, given the red retention signal and very low proportion of acceptable fidelity deliveries, the authors should consider whether a second feasibility or pilot study (with a revised intervention and training package) is needed before proceeding to a full-scale definitive trial.

**Reviewer**	**2**
**Name**	**Saadat, Maryam**
**Affiliation**	**Ahvaz Jundishapur University of Medical Sciences**
**Date**	**22-May-2026**
**COI**	**None**

Abstract

• Please spell out the full term for the acronym EPIC Neck at its first occurrence in the manuscript

• The stated objective is too general and does not fully reflect the feasibility components typically required in a pilot study. I suggest revising the objective to more clearly specify which feasibility domains will be evaluated

• Set keywords based on MESH

Introduction

• Include a reference for line 76

• The Introduction does not sufficiently define the knowledge void that the study intends to fill. Please clarify it

Method

• The manuscript does not clearly describe the inclusion criteria for participants with non specific neck pain

• "The manuscript states that 45 participants were targeted to estimate retention assuming 80% retention with a 95% CI ±12%. However, the method used to derive this sample size is not described

Results

• The authors refer to 'IMD decile'. Please briefly define this term for readers who may not be familiar with this specific UK-based socioeconomic index

• Regarding the postgraduate training areas reported, could the authors clarify the method of data collection? Specifically, were these qualifications verified through official certificates/records, or were they based on self-reported information from the physiotherapists? Please specify the method used to validate these training credentials

Discussion

• The statement regarding refinement of the intervention is too general. Based on the reported deficiencies in patient understanding and physiotherapist confidence, more explicit and actionable strategies are needed. Please clearly outline how the intervention and training package will be modified to address these issues before proceeding to a definitive trial.

**Reviewer**	**3**
**Name**	**Hernández-Beltrán, Víctor**
**Affiliation**	**Universidad de Extremadura**
**Date**	**30-May-2026**
**COI**	**None**

After reading and reviewing the article, I have detected several issues. Why did you submit two articles? It is incomprehensible.

A lot of unnecessary information, the article is too long, and the results are not significant; only descriptive analyses were carried out. No chi-squared value has been shown. What is the aim of Table 7? What are the authors comparing to? No analysis statistic value was added. What analysis did they use?

From my point of view, the authors have to clarify the information, reorder the section, and explain briefly and clearly all the stages of the study.

In this line, if the authors reorder the structure and submit the original article, it can be reviewed again. But, in the present form, it is impossible to revise. It is too long, and they mix two articles.

VERSION 1 - AUTHOR RESPONSE

Reviewer 1

Section	Feedback	Response	Updated Text
General	The study addresses an important question and uses an appropriate feasibility design. That said, the overall message of the paper appears somewhat more positive than the data seem to support. While recruitment was successful and contamination appears low, retention at 6 months was suboptimal and intervention fidelity was not yet strong. These findings suggest promise, but also indicate that meaningful refinement is still needed before progression to a definitive trial can be fully justified.	Thank you for this helpful overall assessment. We agree that the manuscript should more clearly reflect the mixed feasibility profile. We have therefore revised the abstract, discussion and conclusion to present the findings more cautiously and proportionately. We address your specific points in detail below.	-
Title/Abstract	The abstract is clear and well-structured overall, but the conclusion should be phrased more cautiously. In particular, the statement that progression to a definitive trial is feasible may overstate the findings, given that retention was low and only a small proportion of participants received the intervention at an acceptable fidelity level. The abstract would benefit from a more balanced summary of the feasibility signals, making it clearer that progression appears possible only with important modifications. The acceptability findings are also somewhat mixed, as concerns about intervention coherence were identified, and this	Thank you. The conclusion has now been moderated to better reflect the conditional nature of progression.	**Original (Abstract):**<br>Conclusions: Progression to a definitive trial is feasible with modifications to improve retention and strengthen delivery fidelity and patient understanding of key EPIC-Neck components. Further intervention refinement and repeat fidelity evaluation are recommended prior to definitive testing.<br><br>**Updated:**<br>Conclusions: EPIC-Neck shows potential for evaluation in a definitive trial, but progression is conditional on future refinement of the intervention and study procedures to optimise retention, delivery fidelity and intervention coherence.

Section	Feedback	Response	Updated Text
	nuance should be better reflected in the concluding statements.		
Background	The topic is well chosen and the rationale for evaluating a co-developed intervention for chronic non-specific neck pain is understandable. However, the introduction would benefit from a sharper articulation of the specific research gap and a clearer explanation of why this intervention is sufficiently distinct from existing exercise-based care approaches to warrant feasibility testing in its current form. If novelty is emphasized, this should be framed carefully and supported precisely. It would also help to state the feasibility objectives more explicitly and avoid any implication that the study is intended to demonstrate effectiveness.	Thank you for these comments. We have revised the Introduction to clarify that EPIC-Neck was designed to differ from usual exercise care by combining individualised biopsychosocial exercise prescription, structured behaviour change strategies and patient-facing resources. We have also made the feasibility objectives more explicit, including to address uncertainties about recruitment and retention in an exercise-versus-exercise trial, acceptability, delivery fidelity, and whether EPIC-Neck remained sufficiently distinct from usual exercise care to avoid contamination.	**Original (Background):**<br>To address these limitations, the EPIC-Neck programme was co-developed with patients and physiotherapists using Intervention Mapping 22. It is an evidence-based, theoretically informed, individualised programme that integrates behaviour change techniques and a structured exercise prescription framework to maximise clinical effectiveness and adherence 22.<br><br>Prior to evaluating the long-term clinical and cost-effectiveness of the EPIC-Neck programme, the Medical Research Council recommends complex interventions undergo feasibility testing to address uncertainties that could compromise a definitive trial 23. For the EPIC-Neck programme, key uncertainties included its acceptability to patients and physiotherapists (which may influence adherence and engagement), delivery fidelity (which could dilute treatment effects), and the risk of contamination between intervention and control conditions.<br><br>**Updated:**<br>To address these limitations, the EPIC-Neck programme (EPIC: Exercise Prescription Improved through Co-design) was co-developed with patients and physiotherapists using Intervention Mapping.22 EPIC-Neck is an evidence-based, theoretically informed exercise programme that integrates behaviour change techniques and

Section	Feedback	Response	Updated Text
			a structured exercise prescription framework.22 It was designed to differ from usual exercise care by combining individualised, physiotherapist-prescribed biopsychosocial exercise with structured behaviour change strategies and patient-facing resources to optimise exercise effects and sustain adherence.22<br><br>Before evaluating the long-term adherence, clinical effectiveness and cost-effectiveness of EPIC-Neck compared with usual exercise care, key uncertainties that could compromise a definitive trial needed to be addressed.23 The Medical Research Council recommends that complex interventions, such as EPIC-Neck, undergo feasibility testing before definitive evaluation.23 For EPIC-Neck, key uncertainties included whether adults with chronic non-specific neck pain could be recruited and retained in an exercise-versus-exercise trial, whether EPIC-Neck was acceptable to patients and physiotherapists, whether key components could be delivered as intended, and whether the intervention remained sufficiently distinct from usual exercise care to avoid contamination between trial arms.
Methods	The use of a multicentre randomised feasibility design with embedded process evaluation is appropriate and a clear strength of the study. The progression criteria are also useful. However, the manuscript should ensure that these criteria are fully justified and consistently interpreted throughout. In particular, more clarity may	Thank you for this comment. We have added a brief rationale under each progression-criteria heading.	**Updated (Methods):**<br>Recruitment<br>Thresholds were based on the estimated time required to recruit the anticipated definitive trial sample size of 248 participants across 5 musculoskeletal services.37 Green indicated recruitment within <18 months, amber indicated 18–30 months, and red indicated >30 months.<br><br>Retention

Section	Feedback	Response	Updated Text
	be needed around how acceptable fidelity was defined and why the selected thresholds were chosen.		Thresholds were informed by guidance that ≥80% retention represents low risk of attrition bias and comparable musculoskeletal trials reporting approximately 70% retention; therefore, ≥80% was classified as green and <70% as red.40-45<br><br>Delivery fidelity<br>Progression thresholds were based on the distribution of checklist scores across components: green indicated most components delivered acceptably consistent with previous fidelity research using this approach (≥3), 49 47 50 amber indicated mixed or inconsistent delivery requiring refinement, and red indicated major fidelity concerns.<br><br>Contamination<br>Thresholds reflected the extent to which EPIC-Neck components appeared in usual care: green indicated no or minimal overlap, amber limited overlap, and red substantial overlap suggesting insufficient distinction between trial arms. Final progression judgements were made through discussion within the study team (JP, MB and CG), informed by the checklist scores.<br><br>Acceptability<br>Items were rated on 1–5 Likert scales and interpreted at face value according to the questionnaire response anchors, where scores of 4–5 indicated positive acceptability, 3 indicated neutrality and 1–2 indicated negative acceptability.53 ….<br>Progression criteria combined these quantitative thresholds with qualitative findings: green indicated positive acceptability

Section	Feedback	Response	Updated Text
			with no substantive interview concerns, amber indicated neutral or mixed acceptability and/or addressable qualitative concerns, and red indicated negative acceptability and/or major qualitative concerns. Final progression judgements were made through discussion between JP and CG, informed by questionnaire scores and qualitative findings. Any disagreements were resolved through discussion with a third study team member.
Methods	The implications of the 2:1 allocation ratio should also be briefly justified.	Thank you for this comment. We have added a brief justification for the 2:1 allocation ratio and acknowledged it as a limitation. This ratio was used to increase the number of participants receiving EPIC-Neck, thereby ensuring sufficient intervention exposure and feedback to inform feasibility, acceptability and intervention refinement.	**Updated (Methods - Randomisation):**<br>A 2:1 allocation ratio in favour of the intervention group was used to ensure sufficient intervention exposure and participant feedback to inform feasibility, acceptability and intervention refinement.30<br><br>**Updated (Discussion - Limitations):**<br>The 2:1 allocation ratio increased intervention exposure and feedback but resulted in fewer usual exercise care participants, reducing the precision of usual-care estimates and limiting interpretation of between-group descriptive patterns.
Methods	Given that participants and outcome assessors were not blinded, the discussion of potential bias should be sufficiently transparent.	Thank you for this comment. We agree that the potential for bias due to lack of participant and outcome assessor blinding should	**Updated (Discussion - Limitations):**<br>Participants and outcome assessors were not blinded, which may have introduced performance or detection bias, particularly for self-reported outcomes; blinding of outcome assessment will therefore be

Section	Feedback	Response	Updated Text
		be acknowledged more explicitly. Although the Methods section already states that participants and outcome assessors were not blinded, we have added this as a limitation and clarified that blinding procedures will be implemented in a definitive trial.	incorporated into the definitive trial where feasible.
Methods	In addition, the methods should make clear how therapist training, support, and monitoring were handled, especially since fidelity emerged as an important issue.	Thank you for this comment. We have clarified how physiotherapist training and support were provided, and distinguished these from fidelity evaluation. Audio-recordings were used to assess delivery fidelity, but real-time monitoring, feedback or delivery coaching were not provided during the trial.	**Original (Methods - Physiotherapist training):** Physiotherapists delivering EPIC-Neck completed a one-day, face-to-face training combining lectures, role play and behavioural skills practice 33 34. Ongoing support included a training manual, crib sheets, optional booster sessions, and peer/expert support 33 35.<br><br>**Updated:** Physiotherapists delivering EPIC-Neck completed a one-day, face-to-face training combining lectures, role play and behavioural skills practice.33 34 Ongoing support included a training manual, crib sheets, optional booster sessions, and peer/expert support.33 35 Audio-recordings of intervention delivery were used for fidelity evaluation, but real-time monitoring, feedback or delivery coaching were not provided during the trial.
Results/ Discussion	The results are informative, but the interpretation should remain closely aligned with the actual feasibility findings.	Thank you for this comment.<br><br>We agree that interpretation	**Original (Discussion):** The findings show that a definitive trial is feasible but there are important concerns relating to participant retention, delivery

Section	Feedback	Response	Updated Text
	Recruitment appears to have been successful, which is encouraging.<br><br>However, retention at 6 months was below an ideal level for a future definitive trial, and this should be presented as a significant issue rather than a minor limitation.<br><br>Similarly, the fidelity findings appear central to the feasibility question and should be given greater emphasis. The fact that only a small proportion of participants received the intervention at an acceptable level suggests that the intervention may not yet be sufficiently deliverable in routine practice in its current form.<br><br>The findings on acceptability are useful, but they should be reported with more nuance, as positive responses seem to coexist with concerns about intervention coherence.<br><br>The discussion is likely to benefit from a more cautious tone overall. The manuscript should avoid giving the impression that the feasibility trial was broadly successful when, in fact, the results appear mixed across key progression domains (green for recruitment/contamination, amber for fidelity/acceptability, red for retention). Recruitment	should remain closely aligned with the feasibility findings. The Results section already reports the progression criteria reached for recruitment, retention, delivery fidelity, contamination and acceptability, including that recruitment met the Green criterion, retention met the Red criterion, delivery fidelity met the Amber criterion, and acceptability met the Amber criterion. To avoid over-interpreting within the Results, we have retained the descriptive presentation there, but revised the Discussion to give greater emphasis to the implications of below-target retention and delivery fidelity for progression to a definitive trial.<br><br>We have revised the Discussion	fidelity and intervention coherence. However, the EPIC-Neck programme is broadly acceptable and demonstrably different from usual exercise care.<br><br>**Updated:**<br>The feasibility findings were mixed. Recruitment and contamination met Green progression criteria, indicating no major concerns in these domains. However, retention met the Red criterion and delivery fidelity met the Amber criterion, indicating important feasibility concerns that require resolution before a definitive trial. Acceptability to both patients and physiotherapists was broadly positive, but qualitative findings identified addressable concerns within the TFA domain of intervention coherence, particularly participants' understanding of the intervention rationale and physiotherapists' clarity around delivering newer or more complex components.<br><br>**Original (Discussion – recruitment was feasible, but retention posed modifiable challenges):**<br>This is below the ≥80% needed to minimize attritions bias and below rates typically observed in clinical trials, raising concerns over retention strategies if carried forward to a definitive trial 40-44.<br><br>**Updated:**<br>This is below the ≥80% target associated with lower risk of attrition bias and below the minimum 70% threshold prespecified for progression, raising concerns about whether current retention strategies would

Section	Feedback	Response	Updated Text
	and contamination findings are encouraging, but retention and fidelity are major feasibility concerns and should be discussed as such.<br>The qualitative findings seem especially important here, as they may help explain why fidelity and coherence were not stronger.	to provide a more balanced interpretation, giving greater emphasis to retention and delivery fidelity as important feasibility concerns, and to the qualitative findings explaining intervention coherence issues.	be sufficient in a definitive trial.40-44<br><br>**Original (Discussion - Intervention coherence and delivery are suboptimal):**<br>Addressing these issues is crucial before progressing to a definitive trial. Poor fidelity can reduce treatment effects 81, risk misinterpreting null findings 82 83 and threaten internal validity 84. This is particularly important where fidelity concerns relate to intervention components intended to support long-term self-management (such as the EPIC-Neck performance objectives) given that the primary outcome of a definitive trial would be long-term clinical outcomes. These concerns are, however, modifiable. Consistent with Medical Research Council guidance, EPIC-Neck and its training package can be refined and re-tested, with a focus on strengthening delivery fidelity and intervention coherence before progressing to a definitive evaluation 23.<br><br>**Updated:**<br>Addressing these issues is crucial before progressing to a definitive trial. Poor fidelity can reduce treatment effects,81 risk misinterpreting null findings 82 83 and threaten internal validity.84 However, delivery fidelity should not be interpreted as a simple success/failure judgement. Some EPIC-Neck components were delivered acceptably, and evidence from complex behavioural interventions indicates that effectiveness may still be possible despite variable or moderate delivery fidelity, depending on which components

Section	Feedback	Response	Updated Text
			are most critical to the intervention mechanism.46 85 86 So EPIC-Neck may still prove to be effective. Nevertheless, fidelity concerns are particularly important for EPIC-Neck components intended to support long-term self-management, such as flare-up management and long-term exercise, because these may plausibly influence long-term clinical outcomes in a definitive trial. These concerns are, however, modifiable. Consistent with Medical Research Council guidance, EPIC-Neck and its training package can be refined and re-tested, with a focus on strengthening delivery fidelity and intervention coherence before progressing to a definitive evaluation.23<br><br>**Original (Discussion - EPIC-Neck was acceptable with concerns about intervention coherence and was distinct from usual exercise care):**<br>These coherence deficits align with the lower delivery fidelity scores for the corresponding components (mean scores <3), suggesting that limited patient understanding was at least partly driven by suboptimal physiotherapist delivery.<br><br>**Updated:**<br>These coherence deficits align with lower delivery fidelity scores for the corresponding components (mean scores <3), suggesting that limited patient understanding may have been related, at least in part, to suboptimal physiotherapist delivery.<br><br>**Original (Discussion):**

Section	Feedback	Response	Updated Text
			EPIC-Neck is broadly acceptable and distinct from usual exercise care<br><br>**Updated:**<br>EPIC-Neck was acceptable with concerns about intervention coherence and was distinct from usual exercise care<br><br>**Original (Discussion - EPIC-Neck was acceptable with concerns about intervention coherence and was distinct from usual exercise care):**<br>Overall, EPIC-Neck is a novel and acceptable intervention, warranting refinement and further fidelity testing before definitive evaluation.<br><br>**Updated:**<br>Overall, EPIC-Neck appears acceptable and sufficiently distinct from usual exercise care, but requires refinement and further fidelity testing before definitive evaluation.
Discussion	It would strengthen the paper if the discussion more clearly outlined specific, actionable modifications to the intervention, training package, and trial procedures that will be tested before any future definitive trial is undertaken. A vague statement that "refinement is needed" is insufficient.	Thank you for this comment. We have added a new "Future refinement before a definitive trial" section outlining candidate refinements to trial procedures, intervention delivery, physiotherapist training and intervention coherence. These are presented as proposed areas for further development	**Updated (Discussion):**<br>Future refinement before a definitive trial<br>Before progression to a definitive trial, key refinements are needed to optimise retention, delivery fidelity and intervention coherence. Retention strategies should address both questionnaire return and treatment engagement, as missing follow-up data appeared linked to treatment non-initiation and non-attendance. Future procedures could consider reducing questionnaire burden (e.g., electronic completion or shorter questionnaires), increasing the financial incentive for completion, and incorporating blinding procedures where feasible.43 78-80 Additional

Section	Feedback	Response	Updated Text
		with patients, physiotherapists and stakeholders, rather than as fixed modifications.	strategies to optimise delivery and intervention coherence should be designed collaboratively with patients and physiotherapists, and informed by theory and existing evidence. Potential refinements include strengthening physiotherapist training through longer training duration, additional practice, and regular monitoring and feedback.88-92 Intervention coherence may improve through better delivery, but additional strategies could include methods to check patient understanding during treatment, such as teach-back approaches.93
Discussion	Additionally, the discussion should more transparently address the risk of allegiance bias and the implications of the unmet sample size.	Thank you for this comment. We have revised the limitations section to more explicitly acknowledge the potential for allegiance bias, and that the trial did not reach the target sample size.	**Original (Discussion – Limitations):**<br>This trial also has limitations. Physiotherapists delivered the EPIC-Neck programme to relatively few patients; therefore, some fidelity issues may be overestimated and could improve with increased exposure and practice 95 96. Fidelity assessments were undertaken unblinded, which may have influenced scoring despite use of a structured competence checklist. In addition, interviews were conducted by the intervention developer, raising the possibility of social-desirability bias, although the use of a topic guide and reflexive discussions helped mitigate this risk.<br><br>**Updated:**<br>This trial also has limitations. The target sample size was not reached, which limits the precision of feasibility estimates and means progression criteria and subgroup patterns should be interpreted cautiously. Participants and outcome assessors were not blinded,

Section	Feedback	Response	Updated Text
			which may have introduced performance or detection bias, particularly for self-reported outcomes; blinding of outcome assessment will therefore be incorporated into the definitive trial where feasible. Physiotherapists delivered the EPIC-Neck programme to relatively few patients; therefore, some fidelity issues may be overestimated and could improve with increased exposure and practice.95 96 Fidelity assessments were undertaken unblinded, which may have influenced scoring despite use of a structured competence checklist. In addition, interviews were conducted by the intervention developer, whose role in EPIC-Neck development may have introduced social desirability and allegiance bias. The use of topic guides, reflective discussions and team-based interpretation helped mitigate this risk, but independent interviewing and analysis would strengthen future evaluations.
Conclusion	The conclusion should be moderated. At present, the data seem to support conditional feasibility rather than clear readiness for a definitive trial. It would be more accurate to conclude that the intervention shows potential, but requires further refinement, improved delivery fidelity, and better participant retention before definitive evaluation. A slightly more cautious and proportionate conclusion would better reflect the study findings. In fact, given the red retention signal and very low	Thank you. The conclusion has been moderated to make it clear that progression is conditional.	**Original (Conclusion):**<br>Progression to a definitive trial evaluating the clinical and cost-effectiveness of EPIC-Neck compared to usual exercise care is feasible, with modification to improve participant retention, delivery fidelity and intervention coherence. The EPIC-Neck intervention was broadly acceptable and different from usual exercise care. Future research should refine the EPIC-Neck programme and re-assess fidelity and retention rates prior to progressing to a definitive trial.<br><br>**Updated:**<br>The EPIC-Neck programme shows potential for evaluation in a

Section	Feedback	Response	Updated Text
	proportion of acceptable fidelity deliveries, the authors should consider whether a second feasibility or pilot study (with a revised intervention and training package) is needed before proceeding to a full-scale definitive trial.		definitive trial, but progression is conditional on further refinement. Recruitment and contamination findings were encouraging, and the intervention was broadly acceptable and distinct from usual exercise care. However, retention, delivery fidelity and intervention coherence require optimisation before definitive evaluation. Future work should refine the EPIC-Neck programme, training package and trial retention procedures, followed by targeted repeat testing of delivery fidelity, participant and physiotherapist understanding of the intervention and retention before progressing to a full-scale definitive trial.

Reviewer 2

Section	Feedback	Response	Updated Text
General	Please spell out the full term for the acronym EPIC Neck at its first occurrence in the manuscript	Thank you. We have written out the full intervention name at first mention in the main manuscript and added the abbreviation to the abbreviations list.	**Original (Background):**<br>To address these limitations, the EPIC-Neck programme was co-developed with patients and physiotherapists using Intervention Mapping.22<br><br>**Updated:**<br>To address these limitations, the EPIC-Neck programme (EPIC: Exercise Prescription Improved through Co-design) was co-developed with patients and physiotherapists using Intervention Mapping.22
Title/ Abstract	The stated objective is too general and does not fully reflect the feasibility components typically required in a pilot study. I suggest revising the objective to more clearly specify which feasibility	Thank you. We have revised the objective wording to make this more explicit.	**Original (Aims and objectives):**<br>Objectives: To assess the feasibility of conducting a definitive randomised controlled trial evaluating the clinical and cost-effectiveness of the EPIC-Neck programme compared with usual exercise care for people with chronic non-specific neck pain.<br><br>**Updated:**

Section	Feedback	Response	Updated Text
	domains will be evaluated		Objectives: To assess the feasibility of conducting a definitive randomised controlled trial of the EPIC-Neck programme versus usual exercise care for chronic non-specific neck pain, specifically in relation to recruitment, retention, delivery fidelity, acceptability and contamination.
Title/ Abstract	Set keywords based on MESH	Thank you. The keywords have been revised to align with MeSH descriptors. As no suitable MeSH heading was identified for intervention fidelity, this term has been retained.	**Original:**<br>Neck pain, exercise, self-management, behaviour change, feasibility trial, acceptability, fidelity, complex intervention development<br><br>**Updated:**<br>Neck Pain, Exercise Therapy, Self-Management, Feasibility Studies, Patient Acceptance of Health Care, Intervention Fidelity
Background	The Introduction does not sufficiently define the knowledge void that the study intends to fill. Please clarify it	Thank you for these comments. We have made the feasibility uncertainties more explicit, including recruitment and retention in an exercise-versus-exercise trial, acceptability, delivery fidelity, and whether EPIC-Neck remained sufficiently distinct from usual exercise care to avoid contamination.	**Original (Background):**<br>To address these limitations, the EPIC-Neck programme was co-developed with patients and physiotherapists using Intervention Mapping 22. It is an evidence-based, theoretically informed, individualised programme that integrates behaviour change techniques and a structured exercise prescription framework to maximise clinical effectiveness and adherence 22.<br><br>Prior to evaluating the long-term clinical and cost-effectiveness of the EPIC-Neck programme, the Medical Research Council recommends complex interventions undergo feasibility testing to address uncertainties that could compromise a definitive trial 23. For the EPIC-Neck programme, key uncertainties included its acceptability to patients and physiotherapists (which may influence adherence and engagement), delivery fidelity (which could dilute treatment effects), and the risk of contamination between intervention and control conditions.<br><br>**Updated:**

Section	Feedback	Response	Updated Text
			To address these limitations, the EPIC-Neck programme (EPIC: Exercise Prescription Improved through Co-design) was co-developed with patients and physiotherapists using Intervention Mapping.22 EPIC-Neck is an evidence-based, theoretically informed exercise programme that integrates behaviour change techniques and a structured exercise prescription framework.22 It was designed to differ from usual exercise care by combining individualised, physiotherapist-prescribed biopsychosocial exercise with structured behaviour change strategies and patient-facing resources to optimise exercise effects and sustain adherence.22<br><br>Before evaluating the long-term adherence, clinical effectiveness and cost-effectiveness of EPIC-Neck compared with usual exercise care, key uncertainties that could compromise a definitive trial needed to be addressed.23 The Medical Research Council recommends that complex interventions, such as EPIC-Neck, undergo feasibility testing before definitive evaluation.23 For EPIC-Neck, key uncertainties included whether adults with chronic non-specific neck pain could be recruited and retained in an exercise-versus-exercise trial, whether EPIC-Neck was acceptable to patients and physiotherapists, whether key components could be delivered as intended, and whether the intervention remained sufficiently distinct from usual exercise care to avoid contamination between trial arms.
Background	Include a reference for line 76	Thank you. We have removed the claim relating to the proportion of cases observed in clinical practice, as this was based on unpublished local service	**Original (Background):**<br>Although all types of neck pain contribute to this burden, chronic non-specific neck pain is particularly impactful, representing around 60% of all neck pain cases seen in clinical practice and associated with higher pain, greater disability, and reduced work capacity compared with acute neck pain 2-6.<br><br>**Updated:**

Section	Feedback	Response	Updated Text
		data. We have revised the sentence to focus on the documented clinical impact of chronic non-specific neck pain and retained the supporting references.	Although all types of neck pain contribute to this burden, chronic non-specific neck pain is particularly impactful because symptoms are associated with greater pain, disability, psychological distress, reduced work capacity and healthcare use than acute neck pain.2-6
Methods	The manuscript does not clearly describe the inclusion criteria for participants with non specific neck pain	Thank you. We have clarified the inclusion and exclusion criteria, while retaining reference to the published protocol for full details.	**Original (Methods – Participant eligibility):**<br>Adults (≥18 years) with chronic non-specific neck pain were eligible. Non-specific neck pain was defined as pain or discomfort in the neck and/or shoulder girdle, with or without referred arm pain, for which no specific cause could be identified.26 Exclusions were recent physiotherapy, previous cervical surgery, traumatic or specific neck pathology, neurological involvement, or inability to follow trial procedures. See protocol for full eligibility criteria.25<br><br>**Updated:**<br>Adults (≥18 years) with chronic (≥3 months) non-specific neck pain were eligible. Exclusions were recent physiotherapy (within 6 months), previous cervical surgery, traumatic or specific neck pathology (e.g., whiplash, rheumatoid arthritis, osteoarthritis), neurological involvement (e.g., myelopathy or radiculopathy), or inability to follow trial procedures. See protocol for full eligibility criteria.25
Methods	The manuscript states that 45 participants were targeted to estimate retention assuming 80% retention with a 95% CI ±12%. However, the method used to derive this sample size is not described	Thank you. We have clarified that the sample size was determined using a precision calculation to estimate retention in the	**Original (Methods – Sample size):**<br>Target recruitment was 45 participants, to allow estimation of retention rates, assuming 80% retention with 95% CI ±12% 62 63. No interim analyses or stopping rules were planned.<br><br>**Updated:**<br>Target recruitment was 45 participants, based on a precision calculation to estimate retention with sufficient accuracy

Section	Feedback	Response	Updated Text
		future definitive trial.	to inform a future definitive trial. Assuming 80% retention in this feasibility trial, approximately 43 participants were required to estimate retention with a 95% confidence interval of ±12%; this was rounded to 45 participants.62 63 No interim analyses or stopping rules were planned.
Methods	The authors refer to 'IMD decile'. Please briefly define this term for readers who may not be familiar with this specific UK-based socioeconomic index	Thank you. We have now defined IMD as the Index of Multiple Deprivation and clarified its meaning when first reported.	**Updated (Methods – Inclusivity):**<br>The IMD is a composite measure of area-level deprivation in England, incorporating domains including income, employment, education, health, crime, housing, and living environment, with deciles ranging from 1 (most deprived 10%) to 10 (least deprived 10%).
Methods	Regarding the postgraduate training areas reported, could the authors clarify the method of data collection? Specifically, were these qualifications verified through official certificates/records, or were they based on self-reported information from the physiotherapists? Please specify the method used to validate these training credentials	Thank you. We have clarified that physiotherapist postgraduate training areas were collected as self-reported background characteristics and were not independently verified against certificates or records.	**Original (Methods – Physiotherapist consent procedures):**<br>Physiotherapists at participating sites provided written informed consent to deliver either EPIC-Neck or usual exercise care and to take part in the process evaluation (audio-recorded appointments/interviews). Full details are reported elsewhere.25<br><br>**Updated:**<br>Physiotherapists at participating sites provided written informed consent to deliver either EPIC-Neck or usual exercise care and to take part in the process evaluation (audio-recorded appointments/interviews). Physiotherapist characteristics (age, gender, qualifications, years' experience, and postgraduate training) were collected via a self-reported questionnaire prior to training and were not independently verified against certificates or records. Full details are reported elsewhere.25
Discussion	The statement regarding refinement of the intervention is too general. Based on the reported deficiencies in patient understanding and	Thank you for this comment. We have added a new "Future refinement before a definitive trial" section outlining candidate	**Updated (Discussion):**<br>Future refinement before a definitive trial<br>Before progression to a definitive trial, key refinements are needed to optimise retention, delivery fidelity and intervention coherence. Retention strategies should address both questionnaire return and treatment engagement, as missing follow-up data appeared linked to treatment non-

Section	Feedback	Response	Updated Text
	physiotherapist confidence, more explicit and actionable strategies are needed. Please clearly outline how the intervention and training package will be modified to address these issues before proceeding to a definitive trial.	refinements to trial procedures, intervention delivery, physiotherapist training and intervention coherence. These are presented as proposed areas for further development with patients, physiotherapists and stakeholders, rather than as fixed modifications.	initiation and non-attendance. Future procedures could consider reducing questionnaire burden (e.g., electronic completion or shorter questionnaires), increasing incentives, and incorporating blinding procedures where feasible.43 78-80 Strategies to optimise delivery and intervention coherence should be designed collaboratively with patients and physiotherapists, and informed by theory and existing evidence. Potential refinements include strengthening physiotherapist training through longer training duration, additional practice, and regular monitoring and feedback.88-92 Intervention coherence may improve through better delivery, but additional strategies could include methods to check patient understanding during treatment, such as teach-back approaches.93

Reviewer 3

Section	Feedback	Response	Updated Text
General	After reading and reviewing the article, I have detected several issues. Why did you submit two articles? It is incomprehensible.	Thank you for this comment. We recognise that the relationship between the feasibility trial and the embedded fidelity evaluation required clearer signposting. The two manuscripts address related but distinct research questions. The present manuscript reports the trial-level feasibility findings relevant to progression to a definitive trial, including recruitment, retention, treatment	**Original (Methods - Trial design):**<br>This was a multicentre, two-arm, parallel-group, individually randomised controlled feasibility trial with an embedded convergent mixed methods process evaluation. Participants were randomised in a 2:1 ratio, to either the intervention group (EPIC-Neck programme) or usual exercise care.<br><br>**Updated:**<br>This was a multicentre, two-arm, parallel-group, individually randomised controlled feasibility trial. Participants were randomised in a 2:1 ratio to either the intervention group (EPIC-Neck programme) or usual exercise care. The feasibility trial evaluated outcomes important for deciding whether and how to progress to a definitive trial, including recruitment, retention, treatment engagement, acceptability, contamination, outcome-measure completion and safety. Embedded within the feasibility trial was a mixed-methods process evaluation, which included a multidimensional fidelity evaluation to understand training, delivery, receipt and

Section	Feedback	Response	Updated Text
		engagement, acceptability, contamination, outcome-measure completion and safety. The companion manuscript reports the detailed multidimensional fidelity evaluation, including training, delivery, receipt and enactment of EPIC-Neck. A single paper reporting all of the dimensions of fidelity in detail as well as the other metrics of feasibility would have been much too long. We have revised the Trial design section to make this distinction clearer.	enactment of EPIC-Neck and to inform intervention and training refinements. This manuscript reports the feasibility outcomes relevant to progression to a definitive trial; a detailed multidimensional evaluation of fidelity (reporting training fidelity, delivery fidelity, receipt and enactment) will be reported separately.
General	A lot of unnecessary information, the article is too long	Thank you for this comment. We have reviewed the manuscript for conciseness and removed or shortened text where possible, particularly in the intervention description, methods and results sections. Some methodological detail has been retained or added in response to other reviewers'	-

Section	Feedback	Response	Updated Text
		requests or where needed to clarify feasibility criteria, progression decisions and the relationship between this manuscript and the separate fidelity evaluation.	
Results	The results are not significant; only descriptive analyses were carried out. No chi-squared value has been shown.	Thank you for this comment. This study was designed and reported as a randomised feasibility trial rather than an effectiveness trial. Analyses were therefore primarily descriptive and focused on feasibility outcomes and prespecified progression criteria, rather than formal hypothesis testing. Pearson's chi-squared tests were undertaken only for the assessment of sample inclusivity and are reported in Table 6. In line with recommendations and usual practice for feasibility studies, no inferential analyses were planned for the main feasibility outcomes or planned	The data analysis section has been clarified to state that no inferential between-group comparisons were planned or undertaken for the main feasibility outcomes or planned definitive trial outcome measures. See below for details.

Section	Feedback	Response	Updated Text
		definitive-trial outcome measures.	
Results	What is the aim of Table 7? What are the authors comparing to? No analysis statistic value was added. What analysis did they use?	Thank you for this comment. Table 7 presents descriptive summaries of the planned definitive-trial outcome measures and candidate mediator measures at each timepoint for both groups. The aim is to assess feasibility of outcome data collection, completeness, score distributions and suitability for a future definitive trial, not to formally compare groups or assess clinical effectiveness. In line with feasibility trial guidance and the prespecified analysis plan, no inferential statistical analyses were planned or undertaken for these outcomes. We have updated the Data analysis section and Table 7 title to make this clearer.	**Original (Methods - Data analysis):**<br>No between-group comparisons or subgroup/adjusted analyses were undertaken 68.<br><br>**Updated:**<br>As this was a feasibility trial, planned definitive-trial outcome measures were collected to assess data collection feasibility, completeness, distribution and suitability for a future definitive trial, not to assess clinical effectiveness; therefore, no inferential between-group comparisons were planned or undertaken.68 Return rates and data completeness were summarised as percentages at each time point, and outcome scores were summarised descriptively using mean (SD) and 95% confidence intervals.<br><br>**Original (Results - Table 7):**<br>Table 7 - Outcome measure data collection<br><br>**Updated:**<br>Table 7 - Descriptive summary of planned definitive trial outcome and candidate mediator measures
General	From my point of view, the authors have to clarify the	Thank you for this feedback. We have revised the	**Original (Methods – Trial design):**<br>This was a multicentre, two-arm, parallel-group, individually randomised controlled

Section	Feedback	Response	Updated Text
	information, reorder the section, and explain briefly and clearly all the stages of the study. In this line, if the authors reorder the structure and submit the original article, it can be reviewed again. But, in the present form, it is impossible to revise. It is too long, and they mix two articles.	manuscript to improve signposting and clarify the relationship between the feasibility trial, its embedded process evaluation and separate multidimensional fidelity manuscript. The Trial design section now explains that this manuscript reports feasibility outcomes relevant to progression to a definitive trial, while the detailed multidimensional fidelity evaluation is reported separately. We have also reviewed the manuscript structure and shortened sections where possible to improve clarity and reduce overlap.	feasibility trial with an embedded convergent mixed methods process evaluation. Participants were randomised in a 2:1 ratio, to either the intervention group (EPIC-Neck programme) or usual exercise care.<br><br>**Updated:**<br>This was a multicentre, two-arm, parallel-group, individually randomised controlled feasibility trial. Participants were randomised in a 2:1 ratio to either the intervention group (EPIC-Neck programme) or usual exercise care. The feasibility trial evaluated outcomes important for deciding whether and how to progress to a definitive trial, including recruitment, retention, treatment engagement, acceptability, contamination, outcome-measure completion and safety. Embedded within the feasibility trial was a mixed-methods process evaluation, which included a multidimensional fidelity evaluation to understand training, delivery, receipt and enactment of EPIC-Neck and to inform intervention and training refinements. This manuscript reports the feasibility outcomes relevant to progression to a definitive trial; a detailed multidimensional evaluation of fidelity (reporting training, fidelity, delivery fidelity, receipt and enactment) will be reported separately.

VERSION 2 - REVIEW

Reviewer 1

Name Bagherian, Sajad

Affiliation Shahrekord University

Date 23-Jul-2026

COI

The authors have responded comprehensively to the previous round of reviews and have substantially improved the manuscript. The revisions to the abstract, introduction, discussion and conclusion have appropriately moderated the interpretation of findings, and the new "Future refinement" section provides specific actionable recommendations. The study remains a well-conducted and transparently reported feasibility trial with a robust embedded process evaluation. However, several issues still require attention. The manuscript's length remains excessive, with considerable duplication across sections. The relationship between this feasibility trial and the separate fidelity manuscript needs clearer signposting. Some quantitative results are presented without sufficient context for interpretation (e.g., the completeness of outcome measures in Table 7). Additionally, the authors should clarify whether any changes were made to the trial procedures following the interim PPIE feedback mentioned in the response table. With these relatively minor revisions, the manuscript will be suitable for publication and will make a valuable contribution to the feasibility trial literature.

Abstract

The revised abstract is much improved and now appropriately reflects the conditional nature of progression. The authors have moderated the conclusion to state that "EPIC-Neck shows potential" and that "progression is conditional on future refinement." This is a significant improvement. One minor concern remains: the abstract states that "acceptability was generally positive though interviews identified addressable concerns around intervention coherence," which is accurate, but the authors might consider briefly noting that the retention rate (67%) and delivery fidelity (2.4/5) were the primary barriers to progression, as this would provide a more balanced summary of the mixed feasibility profile.

Introduction

The revised introduction is clearer and now more explicitly states the feasibility uncertainties that the study was designed to address. The authors have clarified that EPIC-Neck was designed to differ from usual exercise care by combining individualised biopsychosocial exercise prescription with structured behaviour change strategies and patient-facing resources. One concern is that the introduction remains somewhat lengthy, with some repetition around the limitations of existing exercise programmes. The authors could consider condensing the background on exercise effectiveness and adherence to focus more sharply on the specific gap that EPIC-Neck addresses. This would improve conciseness without losing key contextual information.

Materials and Methods

The methods are now more clearly reported, with the addition of brief justifications for the progression criteria and the 2:1 allocation ratio. The authors have clarified the distinction between the feasibility trial and the separate fidelity evaluation, which addresses a concern

raised by a previous reviewer. However, the methods section remains quite long. The authors should consider whether some detailed intervention descriptions could be moved to supplementary materials to improve readability. One issue: the authors state that "Participants and outcome assessors were not blinded" but later suggest that "blinding procedures will be implemented in the definitive trial." The authors should clarify whether it is feasible to blind participants in an exercise-versus-exercise trial, and if so, how this would be achieved.

Results

The results are presented clearly and the revised tables are helpful. However, several issues affect interpretation. The authors report that "retention among treatment completers across both groups was high (22/24; 91%)," which is an important finding, but they do not report how many participants completed treatment in each group (this is reported only in the text). A table showing treatment engagement by group would improve clarity. The authors also report that "All participants missing at three months were also missing at six months (9/9)," which suggests that participants who dropped out did so early. The authors should discuss whether this pattern is informative for retention strategies. The Table 7 summary of planned definitive trial outcomes is descriptive, as appropriate for a feasibility trial, but the authors could more explicitly state that no inferential tests were performed.

Discussion

The revised discussion is substantially improved and now more accurately reflects the mixed feasibility profile. The authors have appropriately given greater emphasis to the implications of below-target retention and delivery fidelity. The new "Future refinement" section is a welcome addition and provides specific, actionable modifications. One concern is that the discussion of delivery fidelity might be slightly overoptimistic. The authors note that "evidence from complex behavioural interventions indicates that effectiveness may still be possible despite variable or moderate delivery fidelity," which is accurate, but they might also acknowledge that the very low proportion of participants receiving acceptable fidelity (only 14%) is a substantial concern that requires significant intervention redesign before a definitive trial.

Limitations

The limitations section has been strengthened and now appropriately acknowledges the unmet sample size, lack of blinding, potential for allegiance bias, and the limited number of patients per physiotherapist. One addition would strengthen this section further: the authors should note that the predominantly Asian and deprived sample, while reflecting the local population, may limit generalisability to other UK regions or international settings. The authors might also acknowledge that the PPIE group, while diverse, may not fully represent the views of patients who declined to participate or those who dropped out. This would provide a more complete picture of the study's generalisability.

Conclusion

The revised conclusion is appropriately cautious and now clearly states that "progression is conditional on further refinement." This is a significant improvement over the previous version. The authors appropriately note that "recruitment and contamination findings were encouraging" while "retention, delivery fidelity and intervention coherence require optimisation." The conclusion could be strengthened by more explicitly stating that the authors recommend a second feasibility or pilot study with the refined intervention and procedures before proceeding to a full-scale definitive trial, rather than simply "targeted repeat testing." This would provide clearer guidance for readers and future researchers.

VERSION 2 - AUTHOR RESPONSE

	Section	Comments	Response	Updated Text
1.1	General	The authors have responded comprehensively to the previous round of reviews and have substantially improved the manuscript. The revisions to the abstract, introduction, discussion and conclusion have appropriately moderated the interpretation of findings, and the new "Future refinement" section provides specific actionable recommendations. The study remains a well-conducted and transparently reported feasibility trial with a robust embedded process evaluation. However, several issues still require	Thank you for the positive and constructive feedback. We are pleased that the revisions have improved the manuscript and that the study is now considered a well-conducted and transparently reported feasibility trial. We have addressed the remaining points below, and the additional revisions have helped further improve the clarity, balance and readability of the manuscript.	-

		attention. With these relatively minor revisions, the manuscript will be suitable for publication and will make a valuable contribution to the feasibility trial literature.		
1.2	General	The manuscript's length remains excessive, with considerable duplication across sections.	Thank you for this comment. We have reviewed the manuscript again for duplication and concision. We have shortened sections where possible, particularly the Introduction and Methods, while retaining essential methodological detail required to interpret the feasibility outcomes, progression criteria and embedded process evaluation.	-
1.3	Abstract	The revised abstract is much improved and now appropriately reflects the conditional nature of progression. The authors have moderated the conclusion to state that "EPIC-Neck shows potential" and that "progression is conditional on future refinement." This is a significant improvement.<br><br>One minor concern remains:	Thank you for this positive feedback. We agree that the abstract conclusion should make the main barriers to progression explicit. Although retention and delivery fidelity are already reported in the abstract Results, we have revised the Conclusion to clarify that progression is conditional on addressing the key barriers identified in the feasibility trial: retention, delivery fidelity and	**Original (Abstract):**<br><br>EPIC-Neck shows potential for evaluation in a definitive trial, but progression is conditional on future refinement of the intervention and study procedures to optimise retention, delivery fidelity and intervention coherence.<br><br>**Updated:**<br><br>EPIC-Neck shows potential for evaluation in a definitive trial, but progression is conditional on future refinement of the intervention and study

		the abstract states that "acceptability was generally positive though interviews identified addressable concerns around intervention coherence," which is accurate, but the authors might consider briefly noting that the retention rate (67%) and delivery fidelity (2.4/5) were the primary barriers to progression, as this would provide a more balanced summary of the mixed feasibility profile.	intervention coherence.	procedures to address the key barriers to progression: retention, delivery fidelity and intervention coherence.
1.4	Introduction	The revised introduction is clearer and now more explicitly states the feasibility uncertainties that the study was designed to address. The authors have clarified that EPIC-Neck was designed to differ from usual exercise care by combining individualised biopsychosocial exercise prescription with structured behaviour change strategies and patient-facing resources.	Thank you for this comment. We have condensed the Introduction to reduce repetition around exercise effectiveness and adherence, and to focus more directly on the specific gap addressed by EPIC-Neck.	See “Introduction” in the main manuscript

		One concern is that the introduction remains somewhat lengthy, with some repetition around the limitations of existing exercise programmes. The authors could consider condensing the background on exercise effectiveness and adherence to focus more sharply on the specific gap that EPIC-Neck addresses. This would improve conciseness without losing key contextual information.		
1.5	Methods	The methods are now more clearly reported, with the addition of brief justifications for the progression criteria and the 2:1 allocation ratio. The authors have clarified the distinction between the feasibility trial and the separate fidelity evaluation, which addresses a concern raised by a previous reviewer.	Thank you for this comment. We have retained concise signposting in the Trial design section to clarify that this manuscript reports feasibility outcomes relevant to progression to a definitive trial, while the detailed multidimensional fidelity evaluation is reported separately. As both manuscripts are currently under review, we have not added a formal cross-reference at this stage; if both are accepted, we will work with the	**Original (Methods, Trial Design):**<br>This was a multicentre, two-arm, parallel-group, individually randomised controlled feasibility trial. Participants were randomised in a 2:1 ratio to either the intervention group (EPIC-Neck programme) or usual exercise care. The feasibility trial evaluated outcomes important for deciding whether and how to progress to a definitive trial, including recruitment, retention, treatment engagement, acceptability, contamination, outcome-measure completion and safety. Embedded within the feasibility trial was a
1.6	Methods	The relationship between this		

		feasibility trial and the separate fidelity manuscript needs clearer signposting.	editorial team to add appropriate cross-references during production.	mixed-methods process evaluation, which included a multidimensional fidelity evaluation to understand training, delivery, receipt and enactment of EPIC-Neck and to inform intervention and training refinements. This manuscript reports the feasibility outcomes relevant to progression to a definitive trial; a detailed multidimensional evaluation of fidelity (reporting training, fidelity, delivery fidelity, receipt and enactment) will be reported separately.<br><br>**Updated:**<br>This was a multicentre, two-arm, parallel-group, individually randomised controlled feasibility trial. Participants were randomised in a 2:1 ratio to either the intervention group (EPIC-Neck programme) or usual exercise care. The feasibility trial evaluated outcomes important for deciding whether and how to progress to a definitive trial, including recruitment, retention, treatment engagement, acceptability, contamination, outcome-measure completion and safety. Embedded within the feasibility trial was a mixed-methods process evaluation, which included a multidimensional fidelity evaluation to understand EPIC-Neck training, delivery, receipt and enactment and to inform intervention and training

				refinements. This manuscript reports the feasibility outcomes relevant to progression to a definitive trial; the detailed multidimensional fidelity evaluation is reported separately.
1.7	Methods	However, the methods section remains quite long. The authors should consider whether some detailed intervention descriptions could be moved to supplementary materials to improve readability.	Thank you for this comment. We have reviewed the Methods section again and reduced text where possible. We have retained the brief intervention description because it is essential for interpreting the feasibility outcomes, particularly delivery fidelity, acceptability and contamination, and because more detailed information is already signposted to the published intervention development paper and supplementary materials. We have therefore not moved this short description to supplementary materials.	-
1.8	Methods	One issue: the authors state that "Participants and outcome assessors were not blinded" but later suggest that "blinding procedures will be implemented in the definitive trial." The authors should clarify whether it is feasible to blind	Thank you for this comment. We agree that full participant blinding may be challenging in an exercise-versus-exercise trial, and that the previous wording was too definitive. We have revised the statement to clarify that blinding procedures will be considered where	**Original (Methods, Blinding):**<br>Participants and outcome assessors were not blinded, although blinding procedures will be implemented in the definitive trial.<br><br>**Updated:**<br>Participants and outcome assessors were not blinded in this feasibility trial. Blinding procedures will be considered, where

		participants in an exercise-versus-exercise trial, and if so, how this would be achieved.	appropriate in the definitive trial, rather than implying that all forms of blinding will necessarily be feasible.	appropriate, in the definitive trial.30
1.9	Methods	Additionally, the authors should clarify whether any changes were made to the trial procedures following the interim PPIE feedback mentioned in the response table.	Thank you for this comment. The interim PPIE feedback referred to pragmatic recruitment communication refinements rather than formal changes to the trial design, intervention, progression criteria or outcome assessment procedures. Specifically, when initial recruitment uptake was low, the PPIE group suggested using NHS NoReply SMS messages as an additional route to support participant invitation and engagement. We have clarified this in Supplementary File 4 (GRIPP2-SF).	**Original (Supplementary File 4):**<br>Provided suggestions to improve recruitment processes when initial uptake was low<br><br>**Updated:**<br>Provided suggestions to improve recruitment processes when initial uptake was low, including the use of NHS NoReply SMS messages as an additional route to support participant invitation and engagement.
2.0	Results	The results are presented clearly and the revised tables are helpful. However, several issues affect interpretation. The authors report that "retention among treatment completers across both groups was high (22/24; 91%)," which is an important finding, but they do not	Thank you for this comment. Treatment completion by allocation group is already reported in both the CONSORT flow diagram and the allocated treatment engagement section of the Results: 18/24 EPIC-Neck participants and 6/12 usual exercise care participants completed treatment. In the	No changes were made in response to this comment.

		report how many participants completed treatment in each group (this is reported only in the text). A table showing treatment engagement by group would improve clarity.	interests of concision, and given the reviewer's broader concern about manuscript length, we have not added an additional table duplicating these data.	
2.1	Results	The authors also report that "All participants missing at three months were also missing at six months (9/9)," which suggests that participants who dropped out did so early. The authors should discuss whether this pattern is informative for retention strategies.	Thank you for this comment. We agree that the overlap between 3-month and 6-month non-response is informative and suggests that retention issues may emerge early. The Discussion already addresses this by noting that future retention strategies should focus on both questionnaire return and treatment engagement, particularly because missing follow-up data appeared linked to treatment non-initiation and non-attendance. To avoid adding further length to an already detailed Discussion, we have not expanded this further.	No changes were made in response to this comment.
2.2	Results	Some quantitative results are presented without sufficient context for interpretation (e.g., the completeness of outcome measures in Table 7).	Thank you for this comment. To improve clarity without expanding the Results section, we have added a footnote to Table 7 stating that the table presents descriptive statistics only, to	**Table 7**<br><br>Note: Table 7 reports descriptive statistics only, to assess feasibility of outcome-measure collection, completeness and score distributions for a future definitive trial. No inferential between-group

		The Table 7 summary of planned definitive trial outcomes is descriptive, as appropriate for a feasibility trial, but the authors could more explicitly state that no inferential tests were performed.	assess feasibility of outcome-measure collection, completeness and score distributions for a future definitive trial, and that no inferential between-group comparisons were planned or undertaken.	comparisons were planned or undertaken.
2.3	Discussion	The revised discussion is substantially improved and now more accurately reflects the mixed feasibility profile. The authors have appropriately given greater emphasis to the implications of below-target retention and delivery fidelity. The new "Future refinement" section is a welcome addition and provides specific, actionable modifications.<br><br>One concern is that the discussion of delivery fidelity might be slightly overoptimistic. The authors note that "evidence from complex behavioural interventions indicates that effectiveness may still be possible despite variable or moderate delivery fidelity," which is	Thank you for this comment. We agree that the low proportion of participants receiving EPIC-Neck at an acceptable overall fidelity level should be acknowledged more explicitly, while also avoiding a simple success/failure interpretation of fidelity. We have revised the Discussion to state that although only 14% of participants received EPIC-Neck at an acceptable overall fidelity level, 35% of components were delivered acceptably, meaning some intended intervention mechanisms may still have been activated. We have also clarified that fidelity concerns remain substantial because lower-fidelity components included those intended to support long-term self-management, such	**Original (Discussion):**<br><br>Addressing these issues is crucial before progressing to a definitive trial. Poor fidelity can reduce treatment effects,81 risk misinterpreting null findings 82 83 and threaten internal validity.84 However, delivery fidelity should not be interpreted as a simple success/failure judgement. Some EPIC-Neck components were delivered acceptably, and evidence from complex behavioural interventions indicates that effectiveness may still be possible despite variable or moderate delivery fidelity, depending on which components are most critical to the intervention mechanism.46 85 86 So EPIC-Neck may still prove to be effective. Nevertheless, fidelity concerns are particularly important for EPIC-Neck components intended to support long-term self-management, such as flare-up management and long-term exercise, because these may plausibly influence long-term clinical outcomes in a

		accurate, but they might also acknowledge that the very low proportion of participants receiving acceptable fidelity (only 14%) is a substantial concern that requires significant intervention redesign before a definitive trial.	as flare-up management and general exercise, which may plausibly influence long-term clinical outcomes in a definitive trial.	definitive trial. These concerns are, however, modifiable. Consistent with Medical Research Council guidance, EPIC-Neck and its training package can be refined and re-tested, with a focus on strengthening delivery fidelity and intervention coherence before progressing to a definitive evaluation.22<br><br>**Updated:**<br>Addressing these issues is crucial before progressing to a definitive trial. Poor fidelity can reduce treatment effects,81 risk misinterpreting null findings 82 83 and threaten internal validity.84 However, delivery fidelity should not be interpreted as a simple success/failure judgement. Although only 14% of participants received EPIC-Neck at an acceptable overall fidelity level, 35% of components were delivered acceptably, meaning some intended intervention mechanisms may still have been activated. Evidence from complex behavioural interventions also indicates that effectiveness may still be possible despite variable or moderate delivery fidelity, depending on which components are most critical to the intervention mechanism.46 85 86 Nevertheless, fidelity concerns remain substantial because lower-fidelity EPIC-Neck components included those intended to support long-term self-

				management, such as flare-up management and general exercise, which may plausibly influence long-term clinical outcomes in a definitive trial. These concerns are, however, modifiable. Consistent with Medical Research Council guidance, EPIC-Neck and its training package can be refined and re-tested, with a focus on strengthening delivery fidelity and intervention coherence before progressing to a definitive evaluation.22
2.4	Limitations	The limitations section has been strengthened and now appropriately acknowledges the unmet sample size, lack of blinding, potential for allegiance bias, and the limited number of patients per physiotherapist.<br><br>One addition would strengthen this section further: the authors should note that the predominantly Asian and deprived sample, while reflecting the local population, may limit generalisability to other UK regions or international settings.	Thank you for this comment. We agree that transferability should be interpreted in context. However, we consider the ethnically diverse and socioeconomically deprived sample to be an important strength of this feasibility study, as it reflects the local NHS services involved and demonstrates the feasibility of inclusive recruitment procedures. As this was a feasibility trial, it was not designed to generate generalisable effectiveness estimates. We have therefore retained this as a strength rather than reframing it as a limitation.	No changes were made in response to this comment.
2.5	Limitations	The authors might also acknowledge	Thank you for this comment. We agree	No changes were made in response to this comment.

		that the PPIE group, while diverse, may not fully represent the views of patients who declined to participate or those who dropped out. This would provide a more complete picture of the study's generalisability.	that PPIE contributors may not fully represent all potential participants, particularly individuals who declined participation or disengaged during the study. This is already acknowledged in Supplementary File 4 (GRIPP2-SF), where we note that the PPIE group were highly engaged and motivated and may not fully represent patients who struggle with engagement. Given the reviewer's broader concern about manuscript length, we have not expanded the main manuscript further.	
2.6	Conclusion	The revised conclusion is appropriately cautious and now clearly states that "progression is conditional on further refinement." This is a significant improvement over the previous version. The authors appropriately note that "recruitment and contamination findings were encouraging" while "retention, delivery fidelity and intervention	Thank you for this comment. We have avoided stating that a full second feasibility or pilot study is definitely required, as the appropriate next step should be proportionate to the extent of intervention, training and trial-procedure refinements. We have therefore revised the conclusion to state that targeted repeat testing of delivery fidelity, participant and physiotherapist understanding of the	**Original (Conclusion):**<br>Future work should refine the EPIC-Neck programme, training package and trial retention procedures, followed by targeted repeat testing of delivery fidelity, participant and physiotherapist understanding of the intervention and retention before progressing to a full-scale definitive trial.<br><br>**Updated:**<br>Future work should refine the EPIC-Neck programme, training package and trial retention procedures, followed by targeted repeat testing of delivery fidelity, participant and

		coherence require optimisation."<br><br>The conclusion could be strengthened by more explicitly stating that the authors recommend a second feasibility or pilot study with the refined intervention and procedures before proceeding to a full-scale definitive trial, rather than simply "targeted repeat testing." This would provide clearer guidance for readers and future researchers.	intervention, and retention is needed, and that this may require a further feasibility or pilot study depending on the extent of refinements.	physiotherapist understanding of the intervention and retention. Depending on the extent of these refinements, this may require a further feasibility or pilot study before progressing to a full-scale definitive trial.

VERSION 3 - REVIEW

**Reviewer**	**1**
**Name**	**Bagherian, Sajad**
**Affiliation**	**Shahrekord University**
**Date**	**01-Sep-2026**
**COI**	

Thank you for the careful revisions. The manuscript is substantially improved and is generally well designed and clearly reported. The objectives, methodology, results, and conclusions are appropriate for a feasibility study.

Before publication, I recommend a few minor clarifications:

1. Please clarify the discrepancy between the **two sites described in the protocol** and the **three NHS services reported in the manuscript**.

2. The protocol planned acceptability assessment at **3 and 6 months**, whereas the manuscript reports the 6-month assessment. Please explain whether the 3-month assessment was conducted and, if not, why.

3. Please clarify whether the planned **usual-care physiotherapist interviews** were completed and, if so, where these findings are reported.

4. Please briefly clarify how the overall **acceptability rating** was determined given the reported concerns regarding intervention coherence.

These are minor reporting/clarification issues and do not undermine the overall quality of the study. Addressing them would improve transparency and consistency before publication.

VERSION 3 - AUTHOR RESPONSE

	Comments	Response	Updated Text
1	Please clarify the discrepancy between the **two sites described in the protocol** and the **three NHS services reported in the manuscript**.	We thank the reviewer for identifying this discrepancy. The protocol was written when recruitment was planned across two NHS sites. During study set-up, one site withdrew and two additional NHS musculoskeletal services were recruited. Recruitment was therefore conducted across three NHS musculoskeletal services. The manuscript has been amended to clarify this.	**Methods (Participant eligibility, identification and consent procedures)**<br><br>Recruitment was originally planned across two NHS sites as described in the protocol; however, one site withdrew during study set-up and two additional NHS musculoskeletal services were subsequently recruited.
2	The protocol planned acceptability assessment at **3 and 6 months**, whereas the manuscript reports the 6-month assessment. Please explain	Acceptability questionnaires were collected at both 3 and 6 months as specified in the protocol. However, because findings were highly consistent across timepoints and word-count constraints required prioritisation of key feasibility findings, only the 6-month results are reported in the main body of the manuscript. The manuscript has been amended to clarify this and the full questionnaire findings have been added to Supplementary File 5.<br><br>**EPIC-Neck participant acceptability questionnaire findings at 3 and 6 months**	**Methods (Outcomes - Accpetability)**<br><br>Original:<br><br>EPIC-Neck participants completed 6-month questionnaires covering TFA

<table>
<tr>
<td></td>
<td>whether the 3-month assessment was conducted and, if not, why.</td>
<td>
<table>
<tr><th></th><th>3-months</th><th></th><th></th><th>6-months</th><th></th><th></th></tr>
<tr><th>Domain</th><th>n</th><th>mean</th><th>SD</th><th>n</th><th>mean</th><th>SD</th></tr>
<tr><td>Affective attitude</td><td>19</td><td>4.00</td><td>0.82</td><td>18</td><td>4.06</td><td>0.80</td></tr>
<tr><td>Intervention coherence</td><td>19</td><td>4.16</td><td>0.76</td><td>18</td><td>4.11</td><td>1.08</td></tr>
<tr><td>Opportunity costs</td><td>19</td><td>3.26</td><td>1.28</td><td>18</td><td>3.33</td><td>1.03</td></tr>
<tr><td>Perceived effectiveness</td><td>19</td><td>3.95</td><td>1.03</td><td>18</td><td>4.06</td><td>1.21</td></tr>
<tr><td>Self-efficacy</td><td>19</td><td>3.63</td><td>1.00</td><td>18</td><td>4.09</td><td>0.90</td></tr>
<tr><td></td><td></td><td></td><td></td><td></td><td></td><td></td></tr>
<tr><td>Overall</td><td>19</td><td>3.80</td><td>1.02</td><td>18</td><td>3.93</td><td>1.04</td></tr>
</table>

Note: Overall acceptability was calculated as the mean across the five questionnaire domains.
Abbreviations: SD – Standard deviation
</td>
<td>domains of affective attitude, intervention coherence, perceived effectiveness, opportunity costs and self-efficacy.

Updated:

EPIC-Neck participants completed 3 and 6-month questionnaires covering TFA domains of affective attitude, intervention coherence, perceived effectiveness, opportunity costs and self-efficacy.

Results (Acceptability)

Updated: Acceptability findings were similar at 3 and 6 months, therefore only the 6-month findings are considered in the integrated acceptability assessment. Both 3 and 6 month data are available in Supplementary File 5.</td>
</tr>
<tr>
<td>3</td>
<td>Please clarify whether the planned **usual-care physiotherapist interviews** were</td>
<td>Interviews with physiotherapists delivering usual exercise care were completed. As contamination was minimal and the interviews were conducted primarily to characterise usual exercise care rather than address progression-related feasibility outcomes, they are not reported in this manuscript and will be presented separately.</td>
<td>Methods (Contamination)

Interviews with usual-care physiotherapists were undertaken to</td>
</tr>
</table>

	completed and, if so, where these findings are reported.		further characterise usual exercise care and will be reported elsewhere.
4	Please briefly clarify how the overall **acceptability rating** was determined given the reported concerns regarding intervention coherence.	We thank the reviewer for this comment. The manuscript already describes that overall acceptability was determined through triangulation of quantitative and qualitative findings across all TFA domains. To improve clarity, we have added a brief explanatory sentence emphasising that the overall judgement reflected all domains collectively rather than intervention coherence alone.	**Results (Acceptability)**<br>Original:<br>Integrated interpretation (Table 4) showed that, although questionnaires suggested good patient acceptability across most domains, interviews identified coherence gaps for both participants and physiotherapists. Overall, EPIC-Neck was acceptable, with addressable concerns, meeting the “Amber” progression criterion.<br><br>Updated:<br>Integrated interpretation (Table 4) showed that, although questionnaires suggested good patient acceptability across most domains, interviews identified coherence gaps for both participants and physiotherapists. Although

			intervention coherence was identified as a substantial concern, the overall acceptability judgement reflected findings across all TFA domains rather than any single domain. Overall, EPIC-Neck was acceptable, with addressable concerns, meeting the “Amber” progression criterion.
